# Comprehensive intra-host infection kinetics reveals high arbo-orthoflavivirus transmission potential by neglected vector species, *Aedes scutellaris*

**DOI:** 10.1371/journal.pntd.0012530

**Published:** 2025-05-06

**Authors:** Yudthana Samung, Jutharat Pengon, Chatpong Pethrak, Phonchanan Pakparnich, Saranya Thaiudomsup, Kittitat Suksirisawat, Manop Saeung, Anon Phayakkaphon, Songpol Eiamsam-ang, Thipruethai Phanitchat, Channarong Sartsanga, Tararat Jantra, Patchara Sriwichai, Natapong Jupatanakul

**Affiliations:** 1 Department of Medical Entomology, Faculty of Tropical Medicine, Mahidol University, Bangkok, Thailand; 2 National Center for Genetic Engineering and Biotechnology (BIOTEC), Pathum Thani, Thailand; 3 Department of Entomology, Faculty of Agriculture, Kasetsart University, Bangkok, Thailand; Medizinische Universitat Wien, AUSTRIA

## Abstract

**Background:**

Dengue virus (DENV) and Zika virus (ZIKV) are primarily transmitted by *Aedes* mosquitoes. As most studies on vector competence have focused on *Aedes aegypti* and *Aedes albopictus* while neglecting other *Aedes* species, it is possible that the transmission risks might be underestimated. It is necessary to examine additional species that could potentially serve as competent vectors. This is particularly important considering the potential expansion of their geographical range due to climate change or species-specific vector reduction interventions.

**Methodology/Principal Findings:**

In this study, we examined the infection kinetics and transmission potential of *Aedes scutellaris* from Thailand, comparing to *Ae. aegypti* and *Ae. albopictus*. Our findings demonstrated that *Ae. scutellaris* and *Ae. albopictus* had lower rates of midgut infection compared to *Ae. aegypti* due to smaller blood meal sizes during feeding. However, once the infection has established *Ae. scutellaris* exhibited efficient replication of ZIKV and DENV1–4 in the midguts, secondary organs, and salivary glands. Notably, *Ae. scutellaris* had a low salivary gland escape barrier, with comparable transmissibility as *Ae. aegypti* when inoculated with the same viral load.

**Conclusion:**

This study highlights the potential of *Ae. scutellaris* as a vector for DENV and ZIKV and emphasizes the importance of considering neglected mosquito species in arbovirus transmission and surveillance efforts.

## Author summary

Dengue and Zika are caused by arthropod-borne orthoflaviviruses, and spread primarily through the bite of infected *Aedes* mosquitoes. Most research on DENV and ZIKV transmission has primarily focused on *Aedes aegypti* and *Aedes albopictus* while other *Aedes* species are overlooked, thus the epidemiology of transmission might be underestimated. With climate change together with species-specific mosquito population reduction interventions these neglected *Aedes* species could become increasingly important in sustaining virus transmission. In this study, we examined *Aedes scutellaris*, a mosquito species that co-habitats with *Ae. aegypti* and *Ae. albopictus*, to assess its ability to transmit DENV and ZIKV using a combination of blood feeding and intrathoracic injection methods. Our findings show that although *Ae. scutellaris* had lower initial infection rates due to smaller blood meals, DENV and ZIKV were able to replicate and transmit at levels comparable to *Ae. aegypti* when exposed to similar virus loads. This highlights the need to study a broader range of species to improve virus control and outbreak prevention strategies.

## 1 Introduction

Dengue and Zika infections are both caused by arthropod-borne orthoflaviviruses (arbo-orthoflaviviruses) belonging to the orthoflavivirus family. Dengue virus is responsible for approximately 100–400 million infections worldwide each year [1,2], with severe cases leading to dengue hemorrhagic fever and dengue shock syndrome. Zika virus, on the other hand, gained global attention in 2015 due to rapid expansion across the tropical and subtropical countries in the Americas and more importantly its association with neurological symptoms and congenital malformations in newborns [3,4]. Both viruses are primarily transmitted through the bite of infected *Aedes* mosquitoes. To date, most research on DENV and ZIKV transmission gives primary focus to *Ae. aegypti* as the main vector and *Ae. albopictus* as a secondary one, while other mosquito species are largely overlooked. However, with climate change, there’s potential for arbovirus vectors to spread geographically [5–7], including neglected mosquito species posing an unknown risk of virus transmission. Additionally, several species-specific intervention such as gene drive and *Wolbachia* might cause significant changes in population structure of the primary vectors thus providing ecological gap for the neglected vectors [8,9].

The Scutellaris group of *Aedes* consists of more than 46 species [10] with geographical range that originally covers the Southeast Asia, South Pacific, and Northern Australia [11–13]. The major species of this subgroup is *Ae. albopictus*, which has been one of the most invasive mosquitoes globally. *Ae. scutellari*s [14], also belongs to this subgroup with geographic range that covers Papua New Guinea, Tonga, Southeast Asia, the South Pacific, Australia, and central Thailand [12,14,15], has been considered a potential carrier of the dengue virus in Papua New Guinea and suggested to be responsible for major DENV transmission in the Pacific islands [12,13]. An early study demonstrated an ability of *Ae. scutellaris* to transmit DENV2 in human volunteers but the volunteers were bitten by more than 50 bites of infectious mosquitoes and more importantly it did not compare transmissibility of *Ae. scutellaris* and primary vector such as *Ae. aegypti* [16]. Despite the suggested role of *Ae. scutellaris* in DENV transmission, only one study has made a comparative analysis of vector competence regarding DENV2 infection in *Ae. scutellaris* and the primary vector *Ae. aegypti* [12]. The study found that *Ae. scutellaris* is a moderately efficient DENV2 vector, with salivary gland infection to those of *Ae. aegypti*. However, the study only included DENV2 without evaluation of the virus’s ability to escape from the salivary gland into the mosquito’s saliva as the infective stage.

In this present study, we conducted detailed analyses of DENV1–4 and ZIKV infection kinetics to determine the vector competence of *Ae. scutellaris*, and compared it to Thai laboratory colonies of *Ae. aegypti* and *Ae. albopictus*. Through a combination of artificial membrane feeding and intrathoracic injection, we demonstrated that *Ae. scutellaris* can transmit ZIKV and DENVs at a level similar to *Ae. aegypti*, especially inoculated with the same virus load.

## 2 Materials and methods

### 2.1 Ethic statement

This study was carried out in accordance with the Faculty of Tropical Medicine- animal Care and Use Committee (FTM-ACUC), Mahidol University, Bangkok and the BIOTEC Committee for Care and Use of laboratory animals (BIOTEC-IACUC). Mosquito collection and maintenance of the field colony was processed followed by the approved protocol of FTM – ACUC 008/2023 and BT-Animal 05/2564. Mice were used only for mosquito rearing as a blood source, according to the approved protocol (BT-Animal 05/2564). Mosquito infection assays were performed followed the approved protocol (BT-Animal 05/2564). Human erythrocytes were used for an infection by artificial membrane feeding. Blood was collected from human volunteer following a protocol approved by National Science and Technology Development Agency Institutional Review Board (NIRB-052–2563). Written consent has been obtained from volunteers prior to blood withdrawal by medical technologists.

### 2.2 *Aedes scutellaris* colonization

**Fig 1:**
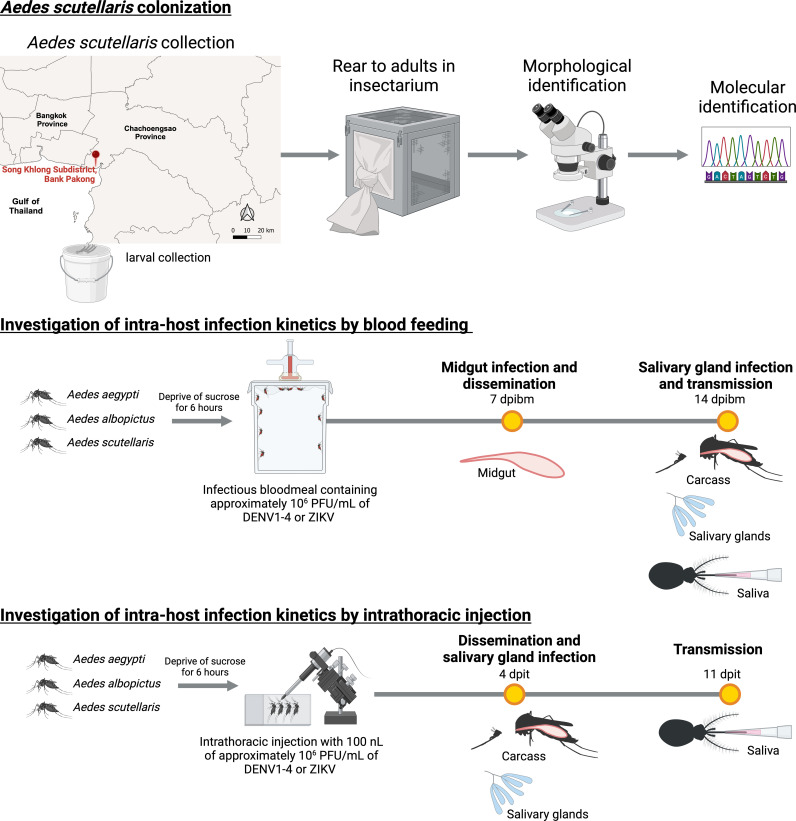
Overview of the *Ae. scutellaris* colonization and arbovirus infection kinetics investigation. *Ae. scutellaris* aquatic stages were collected from the coastal area at Song Khlong Subdistrict, Bank Pakong District, Chachoengsao Province. The map was generated using QGIS software (version 3.22.7). The Thailand base map was obtained from the Humanitarian data exchange (https://data.humdata.org/dataset/cod-ab-tha) under the Creative Commons Attribution for Intergovernmental Organizations (CC BY-IGO) license. Larvae were reared to adults in insectarium and confirmed as *Ae. scutellaris* using morphological and molecular methods. The intra-host infection kinetics of *Ae. scutellaris*, *Ae. aegypti*, and *Ae. albopictus* were investigated by blood feeding or intrathoracic injection. For blood feeding, 6 Log_10_ PFU/mL of ZIKV or DENV1-4 were offered artificial membrane feeding. The midgut infection was measured by plaque assay at 7 dpibm while the dissemination, salivary gland infection and transmissibility were measured at 14 dpibm. The intrathoracic injection was used to determine arbo-orthoflavivirus transmissibility bypassing midgut infection barrier. Individual mosquito was injected with approximately 100 PFU of virus (100 nL of 6 Log_10_ PFU/mL stock). The dissemination and salivary gland infection were measured at 4 dpit while the transmissibility was measured at 11 dpit. The image was created in BioRender (https://BioRender.com/p28g278).

*Ae. scutellaris* subgroup larvae were collected from the coastal area at Song Khlong Subdistrict, Bang Pakong District, Chachoengsao Province between November 2022 to January 2023 (**Fig 1**). A total of approximately 1600 *Aedes spp.* larvae were collected from the field sites. These larvae were subsequently transferred to an insectarium at Faculty of Tropical Medicine, Mahidol university and reared to adults. Individual emerging of *Ae. scutellaris* identified by morphological observation [17] were pooled to establish a field-derived *Ae. scutellaris* colony. Molecular identification of *Ae. scutellaris* was confirmed by PCR based DNA barcode of COI as previously described (S1 File) [18]. Low passage *Ae. scutellaris* (less than six generations) were transferred to BIOTEC’s insectary and used for infection experiments.

### 2.3 Mosquito colonies and maintenance

The recently colonized *Ae. scutellaris* and laboratory strains of *Ae. aegypti* and *Ae. albopictus* were used for all the infection studies. The *Ae. aegypti* DMSC and *Ae. albopictus* TH Laboratory strains were originally obtained from the Department of Medical Sciences, Ministry of Public Health, Thailand. These strains have been maintained in our laboratory for over 30 passages (DMSC) and 20 passages (TH). The DMSC strain has demonstrated the highest transmissibility among all the laboratory and field strains currently maintained in our lab (S1 Fig), highlighting its suitability as a reference for studying vector competence. In contrast, the TH strain exhibits transmissibility levels comparable to other field-collected *Ae. albopictus* strains from Thailand, providing a valuable model for comparison in studies of arbovirus transmission.

Mosquitoes were maintained in BIOTEC’s insectary at 27 °C with 80% humidity and a 12-hours day/night, 30-minutes dusk/dawn lighting cycle. The larvae were fed on powdered fish food (Tetra Bits). Adults were fed on 10% sucrose solution ad libitum. To obtain the eggs for colony maintenance, mosquitoes were allowed to feed on ICR mice anesthetized with 2% Avertin (2,2,2-Tribromoethanol, Sigma, T48402).

### 2.4 Virus propagation and titration

The SV0010/15 Thai ZIKV isolate and the contemporary DENV 1–4 panel from BEI Resources consisting of Dengue Virus Type 1, UIS 998 (NR-49713), Dengue Virus Type 2, US/BID-V594/2006 (NR-43280), Dengue Virus Type 3, US/BID-V1043/2006 (NR-43282), and Dengue Virus Type 4, UIS 497 (NR-49724) were used in the infection studies. All the viruses were propagated using the *Ae. albopictus* cell line C6/36 (ATCC CRL-1660) as previously described [19]. Briefly, after the cells were cultured to 80% confluency in T-75 cm^2^ flasks, supernatant was removed and replaced with the virus stocks at the MOI of 0.1 in 5 mL L-15 medium without supplements for 2 hours. After virus incubation, supernatant was removed, and replaced with 2% FBS L-15 medium then further incubated at 28 °C. The supernatant was collected at 6–7 days post inoculation then supplemented with FBS to final concentration of 20% and stored at −80 °C until further use.

Virus titers was determined by plaque assay in BHK-21 cells following the previously published protocol [19]. Briefly, 100 µL of virus suspension was added to BHK-21 cells seeded in 24-well plate at 80% confluency. Inoculated plates were then gently rocked at room temperature for 15 minutes before an incubation at 37 °C, 5% CO_2_ for 45 minutes. After incubation, 1 mL of overlay medium (1% methylcellulose (Sigma, M0512) in MEM supplemented with 2% FBS and 1X Pen/Strep) was added to each well then further incubated at 37 °C, 5% CO_2_ for 6 days. The plates were then fixed and stained with 0.5% crystal violet (Sigma, C6158) in 1:1 Methanol/Acetone fixative for 1 hour at room temperature. Stained plates were then washed under running tap water and air dried before plaque counting.

### 2.5 Mosquito infection by artificial membrane feeding

Mosquitoes were orally challenged with ZIKV and DENVs using the Hemotek artificial membrane feeding system. Briefly, fifty 7-day-old female mosquitoes were deprived of sucrose solution for six hours before offering with an artificial infectious blood meal containing 40% human erythrocytes (washed twice with 1 volume of RPMI medium) and virus stock diluted to approximately 6 Log_10_ PFU/mL with L-15 supplemented with 10% FBS and 1X Pen/Strep at 37 °C for 30 minutes. The measured exact feeding titers for each virus were 6.26-6.54 log_10_ PFU/mL for DENV1, 6.32-6.38 log_10_ PFU/mL for DENV2, 6.40-6.41 log_10_ PFU/mL for DENV3, 6.08-6.34 log_10_ PFU/mL for DENV4, and 6.32-6.56 log_10_ PFU/mL for ZIKV. After feeding, mosquitoes were then anesthetized in a refrigerator for 15 minutes, and engorged females were sorted on ice. Blood fed mosquitoes were maintained in waxed paper cups with 10% sucrose solution in a climate-controlled chamber under controlled conditions of 28 °C ± 1 °C, a 12-hour light-dark cycle, and 70% relative humidity before tissue collection.

### 2.6 Estimation of blood meal size

The blood meal size of the blood engorged mosquitoes was estimated by quantifying the amount of heme. Recently blood engorged mosquitoes (approximately 30 minutes after offering the blood meal) were collected in 100 µL sterile Milli-Q water and stored at -80 °C until further analysis. The blood meal used in each blood feeding experiment was also stored at -80 °C for use as a standard. To measure heme amount, the stored mosquito samples were thawed and homogenized with 0.5 mm glass beads using Bullet Blender Tissue Homogenizer (NextAdvance). Supernatant of the homogenized samples was then collected after centrifugation at 8,000 xg, 4 °C for 2 minutes. Amount of heme in the supernatant was then measured using Heme assay kit (Sigma, MAK316) following the manufacturer’s protocol. Briefly, 50 µL of supernatant was added to 96-well plate containing 200 µL of heme assay reagent, incubated at room temperature for 5 minutes, and the absorbance was measured at 400 nm. Fifty microliter of Milli-Q water was used as a blank control. For the standard curve, six adult female mosquitoes were homogenized in 600 µL of Milli-Q water. The resulting homogenate was divided into six aliquots, each supplemented with 0, 1, 2, 3, 4, or 5 µL of blood meal. A 50 µL aliquot of each standard sample was then added to a 96-well plate containing 200 µL of heme assay reagent. The absorbance was measured, and a linear standard curve was generated. Blood meal size for each mosquito was calculated by interpolating the absorbance values from the standard curve.

### 2.7 Mosquito infection by intrathoracic injection

The intrathoracic inoculation was conducted by injected 100 nL of virus stock into the thorax of cold-anesthetized 4- to 7-day-old female mosquitoes using a nanoliter injector (Nanoject III; Drummond Scientific). The measured titers used for injection for each virus were 6.23-6.40 log_10_ PFU/mL for DENV1, 6.11-6.34 log_10_ PFU/mL for DENV2, 5.85-6.40 log_10_ PFU/mL for DENV3, 6.11-6.41 log_10_ PFU/mL for DENV4, and 6.36-6.48 log_10_ PFU/mL for ZIKV. Injected mosquitoes were then maintained on 10% sucrose solution at a condition as mentioned above.

### 2.8 Mosquito dissection and salivation assay

Mosquitoes were cold anesthetized in refrigerator for 15 minutes before surface sterilization in 70% ethanol for 1 minute followed by twice PBS washes. Mosquitoes were then individually dissected in drops of 1X PBS. Midguts, carcasses, and salivary glands were collected in 150 µL of MEM supplemented with 10% FBS and 1X Pen/Strep and stored at -80 °C for titration with plaque assay as described above.

Mosquito saliva was collected according to a previously published protocol [19]. Briefly, mosquitoes were paralyzed with triethylamine before inserting the proboscis into a pipette tip containing 20 µL of MEM supplemented with 10% FBS and 1X Pen/Strep. After 45 minutes of salivation, the medium in the tips were mixed with 180 µL of MEM supplemented with 2% FBS and 1X Antibiotics/Antimycotics (penicillin/streptomycin/amphotericin B) and immediately titrated by plaque assay mosquito.

### 2.9 Detection of *Wolbachia* by *wsp* gene detection

The presence of *Wolbachia* was detected by PCR of *wsp* gene following a previously published protocol [20]. Briefly, total DNA was extracted from whole mosquito sample using Quick DNA Mini Prep (Zymo Research, D3024) following manufacturer’s protocol. The PCR detection was conducted using 20 ng of DNA template in Luna Universal qPCR master mix (New England Biolabs, M3003S) with 0.1 µM of wsp-81F (5’-TGG TCC AAT AAG TGA TGA AGA AAC) and wsp-691R primers (5’-AAA AAT TAA ACG CTA CTC CA). The thermal cycling condition was: Initial denaturation of 98 °C for 30 sec, followed by 35 cycles of denaturation at 98 °C for 20 sec, annealing at 45 °C for 20 sec, and extension at 68 °C for 45 sec, followed by final extension at 68 °C for 5 min. Genomic DNA of *Ae. albopictus* and *Culex quinquefasciatus* were used as positive controls.

### 2.10 Data analysis

The Factor Analysis of Mixed Data (FAMD) [21] was used to identify relationship between infection prevalence/median from each infection experiment and mosquito species, virus, and tissue type. FAMD was conducted using FactoMineR package in R [22].

Statistical analyses in this study were conducted using the rstatix package (version 0.7.1) [23] in R (version 4.3.0). Multiple comparison was conducted using Kruskal-Wallis followed by Dunn’s posthoc test. Graphs were generated using the ggpubr package (version 0.6.0) [24] in R.

## 3 Results

*Ae. scutellaris* was successfully colonized and validated using molecular identification with COI gene (S1 File). Additionally, testing for the presence of *Wolbachia* revealed that this colonized population of *Ae. scutellaris* was free of *Wolbachia* (S2 Fig). In order to determine the vector competence of *Ae. scutellaris* for dengue virus serotypes 1–4 (DENV1–4) and Zika virus (ZIKV), we conducted a study to evaluate the intra-host infection kinetics of these viruses in a recently colonized *Ae. scutellaris* population. We compared the infection levels of *Ae. scutellaris* with those of laboratory colonies of *Ae. aegypti* and *Ae. albopictus* with high arbo-orthoflavivirus transmission efficiency. The mosquitoes were fed a blood meal containing approximately 6 Log_10_ PFU/mL of each virus. We assessed the extent of midgut infection 7 days post the infectious blood meal (dpibm), and then determined the dissemination, salivary gland infection, and transmissibility 14 dpibm (**Fig 1**). Additionally, due to a low engorgement rate of *Ae. scutellaris* from artificial membrane feeding, we also infected the mosquitoes by intrathoracic inoculation to determine vector competence when the mosquitoes were infected with the same amount of inoculating viruses. Virus replication in the body and salivary gland infection were determined at 4 days post intrathoracic injection (dpit), and transmissibility was determined at 11 dpit (**Fig 1**). The infection titers of all infected mosquitoes are provided in S1 Table, and the corresponding summary statistics are presented in S2 Table.

### 3.1 *Aedes scutellaris* exhibits a lower prevalence of midgut infection following artificial membrane feeding, but has the lowest salivary gland escape barrier compares to the other two *Aedes* species

**Fig 2:**
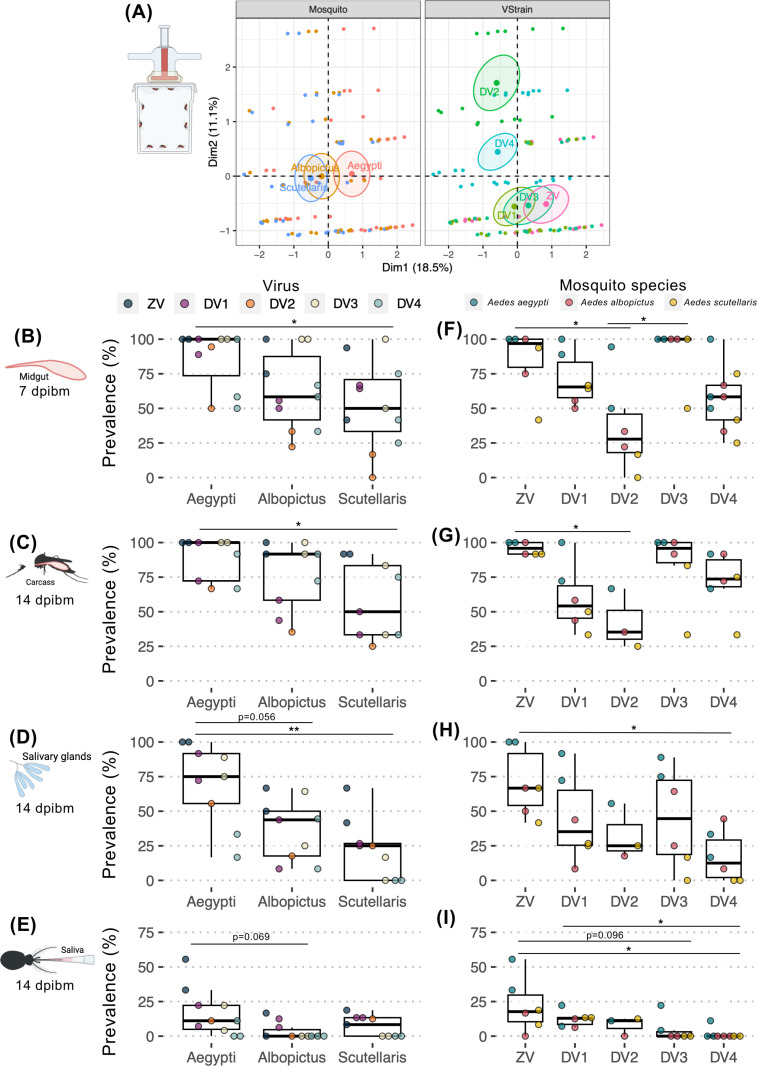
Comparison of ZIKV and DENV1-4 infection prevalence in different infection barriers of *Ae. aegypti*, *Ae. albopictus*, and *Ae. scutellaris* following artificial membrane feeding. (A) Factor Analysis of Mixed Data (FAMD) was used to visualize the relationships between infection prevalence results from each blood feeding experiment on transformed coordinates. Each dot represents infection prevalence data from an individual experiment. The information in the analysis included infection prevalence, mosquito species (Mosquito), and virus (VStrain). Clustering was based on qualitative coordinates (mosquito species, tissue, and virus), with each color indicating individuals belonging to a specific feature. Cluster distances reflect correlations among variables, and confidence ellipses highlight potential relationships. Abbreviation: Dim, dimension. Box and scatter plot comparing infection prevalence in the (B, F) midgut at 7 dpibm, (C, G) carcass at 14 dpibm, (D, H) salivary glands at 14 dpibm, and (E, I) saliva at 14 dpibm. Each dot represents infection prevalence from each blood feeding experiment. The boxes in the plots represent the interquartile range, and the whiskers indicate the range of maximum and minimum values, excluding outliers (less than Q1 - 1.5 X IQR, or more than Q3 + 1.5 X IQR). Statistical analyses were performed using the Kruskal-Wallis followed by Dunn’s post hoc test, with significance levels indicated as *p* < 0.05 (*) and *p* < 0.01 (**). Non-statistically significant results with p ≤ 0.1 were reported as numerical values. All infection experiments were conducted in two replicates, with a minimum of 8 mosquitoes per replicate, except for the carcass, salivary gland, and saliva samples of DENV2, which were derived from a single replicate due to unforeseen accidents during the experimental procedure. The image was created in BioRender (https://BioRender.com/p28g278).

The arbo-orthoflavivirus susceptibility and transmissibility of each *Aedes* species was compared as demonstrated by infection prevalence through each infection barrier (percent of mosquito with infectious virus specific body compartment in total blood fed mosquitoes). Factor analysis of mixed data (FAMD) was used to investigate the relationship between infection prevalence patterns and key determinants, including and mosquito species, and virus. We found that the prevalence of infection of *Ae. scutellaris* was similar to *Ae. albopictus*, both of which differ from *Ae. aegypti* (Fig 2A). To compare overall arbo-orthoflaviviruses susceptibility of each mosquito, we next compared infection prevalence by grouping the data according to mosquito species (Fig 2B-E).

**Fig 3:**
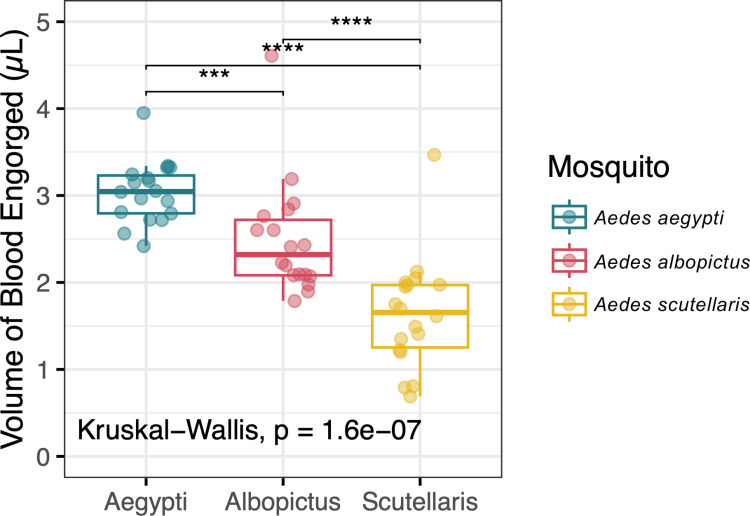
Volume of blood meal engorged by *Ae. aegypti*, *Ae. albopictus*, and *Ae. scutellaris.* Box and scatter plot illustrating the blood meal size of the three *Aedes* species, with each data point representing an individual mosquito. Estimation of blood meal size was conducted by measuring the amount of heme in individual mosquito after blood engorgement. The scatter plot represents the estimated blood meal volume for each individual mosquito from all samples. The boxes in the plots represent the interquartile range, and the whiskers indicate the range of maximum and minimum values, excluding outliers (less than Q1 - 1.5 X IQR, or more than Q3 + 1.5 X IQR). Statistical analyses were performed using the Kruskal-Wallis followed by Dunn’s post hoc test, with significance levels indicated as *p* < 0.001 (***) and *p* < 0.0001 (****). Blood feeding conducted in two replicates, with a minimum of 8 mosquitoes per replicate.

The midgut overall arbo-orthoflaviviruses (ZIKV and DENV1–4) infection prevalence were the lowest in *Ae. scutellaris* followed by *Ae. albopictus,* and lastly *Ae. aegypti* with the median midgut infection prevalence of 50%, 58.3%, and 100%, respectively (**Fig 2B).** Given that the size of blood meal directly influences the inoculation size, it was possible that the observed differences in establishment of midgut infection were due to the differences in blood meal size. Therefore, we determined blood meal size by quantifying amount of heme in the blood engorged mosquitoes. We found that *Ae. aegypti*, *Ae. albopictus*, and *Ae. scutellaris* ingested 3.0 ± 0.4, 2.5 ± 0.7, and 1.6 ± 0.6 µL of blood, respectively **(Fig 3**). With the largest blood meal size among the three *Aedes* species, it was not surprising that *Ae. aegypti* had the highest midgut infection prevalence at 7 dpibm. Interestingly, despite *Ae. scutellaris* having a significantly smaller blood meal size than *Ae. albopictus*, the midgut infection prevalence was similar, suggesting a more permissive midguts of *Ae. scutellaris* compared to *Ae. albopictus*.

In salivary glands, the median infection prevalence of arbo-orthoflaviviruses after infectious artificial blood meal differed significantly among mosquito species, with *Ae. aegypti* exhibiting the highest prevalence (75%), followed by *Ae. albopictus* (43.75%) and *Ae. scutellaris* (25%) (Fig 2C-D). Interestingly, despite *Ae. scutellaris* having a threefold lower prevalence of infection in salivary glands compared to *Ae. aegypti*, no statistically significant difference was observed in virus transmissibility between these two species (11.1% vs. 8.3%, respectively; Fig 2E). This observation indicates that *Ae. scutellaris* possesses a lower salivary gland escape barrier compared to *Ae. aegypti*. Additionally, *Ae. albopictus* had a median prevalence of 0% in saliva despite almost twofold higher prevalence of salivary gland infection, further supporting that *Ae. scutellaris* has a lower salivary gland escape barrier compared to other more extensively studied *Aedes* species.

### 3.2 Tissue-specific infection prevalence of arbo-orthoflaviviruses was driven by viral genetics

To investigate how infection prevalence varies by virus strain, we performed FAMD clustering based on virus-specific data. This analysis revealed that ZIKV, DENV1, and DENV3 clustered together, whereas DENV2 and DENV4 formed distinct groups (Fig 2A).

We next compared the overall infectivity of each virus by grouping infection prevalence data according to virus strain (Fig 2F-I). Across all three *Aedes* species, ZIKV and DENV3 exhibited the highest midgut infection prevalence with the median midgut infection prevalence of 96.9% and 100%, respectively (Fig 2F). In contrast, DENV1, DENV2, and DENV4 showed midgut infection prevalences of 65.5%, 27.8%, and 58.3%, respectively, indicating that ZIKV and DENV3 are particularly efficient at establishing primary midgut infection, while DENV2 is the least efficient.

In subsequent infection barriers, ZIKV consistently demonstrated the highest body, salivary gland, and saliva prevalence across all three *Aedes* species (Fig 2F-I). Among the DENVs, although DENV3 had the highest midgut infection, dissemination, and salivary gland infection, DENV1 salivary gland infection rates increased to levels comparable to DENV3, and surpassed DENV3 transmission rates. Interestingly, while DENV2 had the lowest midgut infection prevalence, the virus had low barrier during subsequent infection steps eventually resulting in similar transmissibility to DENV1 (Fig 2F-I). Conversely, despite DENV3 exhibiting the highest midgut infection prevalence, it was less efficient at overcoming subsequent infection barriers (Fig 2F-H), ranking among the least transmissible viruses (Fig 2I). Likewise, DENV4 also exhibited strong barrier during both salivary gland infection and escape (Fig 2H-I). Overall, these results indicate that tissue-specific infectivity phenotypes are largely determined by viral genetics, as the observed patterns for ZIKV and the DENVs remained consistent across all three *Aedes* species.

### 3.3 Tissues of all three *Aedes* species supported high level of arbo-orthoflavivirus replication

**Fig 4:**
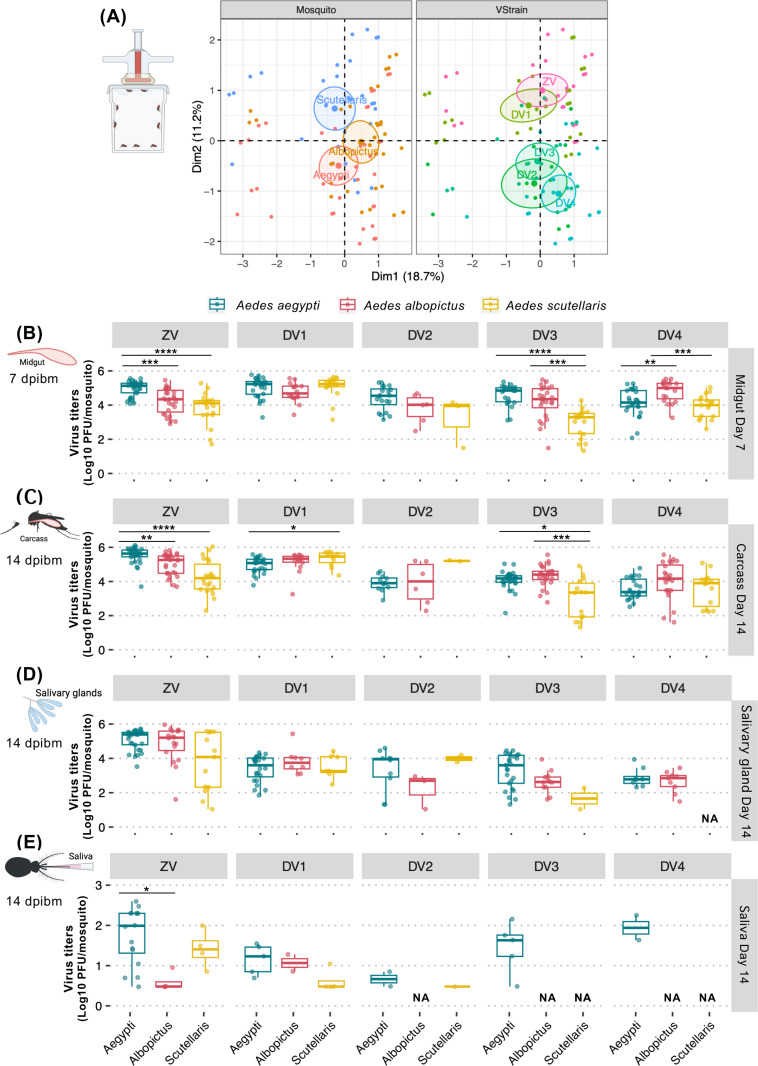
Comparison of ZIKV and DENV1-4 titers in different infection barriers of *Ae. aegypti*, *Ae. albopictus*, and *Ae. scutellaris.* (A) FAMD factor maps to visualize variance between median infection titers from each blood feeding experiment on the transformed coordinates. Each dot represents median infection value from all the infection experiments. The information in the analysis included median infection titers, mosquito species (Mosquito), and virus (VStrain). The clustering is based on qualitative coordinates (mosquito species, tissue and virus), and each color represents individuals in a specific feature. Cluster distance represents the correlation among variables. The confidence ellipses indicate their potential relationships. Abbreviation: Dim, dimension. Box and scatter plot comparing infection titers of individual mosquito in the (B) midguts at 7 dpibm, (C) carcasses at 14 dpibm, (D) salivary glands at 14 dpibm, and (E) saliva at 14 dpibm. Each dot represents virus titers of individual mosquito. The boxes in the plots represent the interquartile range, and the whiskers indicate the range of maximum and minimum values, excluding outliers (less than Q1 - 1.5 X IQR, or more than Q3 + 1.5 X IQR). Only data from infected mosquitoes were used for analyses to allow for the comparison of infection levels in the infected mosquitoes. Groups with no positive virus in the samples were indicated with NA (not available). All the infection experiments were done in two replicates (minimum 8 mosquitoes per replicate) except for the dissemination, salivary gland infection and transmission of DENV2 that have only one replicate due to accidents during experimental procedure. Statistical analyses were conducted using Kruskal-Wallis test followed by post-hoc test with Holm–Bonferroni adjustment, *: p < 0.05, **: p < 0.01, ***: *p* < 0.001, and ****: *p* < 0.0001. The image was created in BioRender (https://BioRender.com/p28g278).

In addition to the infection prevalence, we also investigated how well the tissues of *Ae. scutellaris* support arbo-orthoflaviviruses replication (**Fig 4**). Because higher viral replication in insect vectors may increases transmission risk, we compared infectious virus levels in the body compartments of infected mosquitoes. The FAMD map suggested that the median virus titers in the infected *Ae. aegypti* was more similar to *Ae. albopictus* than *Ae. scutellaris*, which was different from the FAMD map of the infection prevalence that demonstrate more similarity between *Ae. albopictus* and *Ae. scutellaris* (**Fig 2A).** The differences between the FAMD map of infection prevalence and titers was also observed among the virus cluster. While the infection prevalence of DENV1, DENV3, and ZIKV were clustered together, only ZIKV and DENV1 were clustered together and the DENV3 became more similar to DENV2 and DENV4 **(Fig 4A**).

Further comparison of the midgut infection revealed that titers of ZIKV and DENV3 in infected *Ae. scutellaris* were significantly lower than in *Ae. aegypti*, but not for DENV1, DENV2, and DENV4 (**Fig 4B**). These findings indicate that *Ae. scutellaris* midguts can support similar levels of DENV1, DENV2, and DENV4 during the late stage of midgut infection, despite a smaller starting inoculum from a smaller blood meal size. Similarly, midgut titers of ZIKV in *Ae. albopictus* was significantly lower than *Ae. aegypti* while those of DENVs were similar among these two *Aedes* species or even higher than *Ae. aegypti* in the case of DENV4 (**Fig 4B**).

In the next infection barrier, the pattern of infection intensity (virus titers) in the carcasses were similar to those of the midgut infection levels, with *Ae. scutellaris* displaying comparable DENV2 and DENV4 titers, as well as lower ZIKV and DENV3 titers compared to *Ae. aegypti* (**Fig 4C**). Interestingly, DENV1 titers in the carcasses of *Ae. scutellaris* were significantly higher than those in *Ae. aegypti*, suggesting a higher level of DENV1 replication in *Ae. scutellaris* during the late stage of infection. In *Ae. albopictus*, the carcass titers of DENV1–4 were not different from *Ae. aegypti* but those of ZIKV were significantly lower.

The titers of each virus in the infected salivary glands were not statistically different among the three *Aedes* species (**Fig 4D**), indicating that these viruses can efficiently replicate in the salivary glands of *Ae. scutellaris* once infection is established. However, it should be noted that lack of significance can also possibly be due to the low number of infected salivary glands. Indeed, none of the *Ae. scutellaris* salivary glands were infected by DENV4.

### 3.4 Saliva viral loads of arbo-orthoflaviviruses in *Aedes* species following artificial membrane feeding were influenced by both viral and vector genetics

Among mosquitoes with detectable virus in saliva, *Ae. aegypti* exhibited comparable ZIKV titers to *Ae. scutellaris* but significantly higher titers than *Ae. albopictus* (**Fig 4E**) despite similar salivary gland viral loads across all three species. This suggests that *Ae. albopictus* possesses a strong salivary gland escape barrier, limiting the release of infectious virus into saliva compared to *Ae. aegypti* and *Ae. scutellaris*.

Among DENV serotypes, DENV1 titers in saliva were comparable across all three mosquito species, while DENV2 titers in *Ae. aegypti* were similar to those in *Ae. scutellaris*. Comparisons of DENV3 and DENV4 saliva titers between *Ae. aegypti* and the other two species were not possible due to the absence of virus-positive saliva samples in *Ae. albopictus* and *Ae. scutellaris*.

When comparing different DENV serotypes, although the differences were not statistically significant due to the low sample size, we observed trends suggesting variation in the ability of viruses to overcome the salivary gland escape barrier. While salivary gland viral titers were comparable across all DENV serotypes, DENV4 exhibited higher saliva titers, whereas DENV2 had lower saliva titers compared to DENV1 and DENV3. Given the limited number of virus-positive saliva samples, caution is required when interpreting these findings.

### 3.5 *Aedes scutellaris* is as robust as *Aedes aegypti* in arbo-orthoflavivirus transmission when inoculated with similar virus titers

Since *Ae. scutellaris* took a significantly smaller blood meal from the artificial membrane feeding (Fig 3), it is possible that the smaller starting inoculating virus affect the differential transmissibility phenotype among three *Aedes* species. Such effect from different amount of inoculating virus on transmissibility has been demonstrated, even with the same virus and mosquito strain in our previous detailed ZIKV infection kinetics study [19]. It is possible that the recently colonized *Ae. scutellaris* has not adapted to the laboratory environment, leading to an underestimation of its blood meal size during artificial membrane feeding. In natural settings, these mosquitoes may consume larger blood meals than what was observed in the laboratory, potentially resulting in an underestimation of their transmissibility. To overcome this limitation, we infected ZIKV or DENVs in the three *Aedes* species by intrathoracic injection to ensure equal virus inoculation. Here, each individual mosquitoes were injected with 100 nL of approximately 6 Log_10_ PFU/mL of virus (approximately 100 PFU). The body and salivary gland titers were measured at 4 dpit and transmission was evaluated by collecting saliva at 11 dpit.

**Fig 5:**
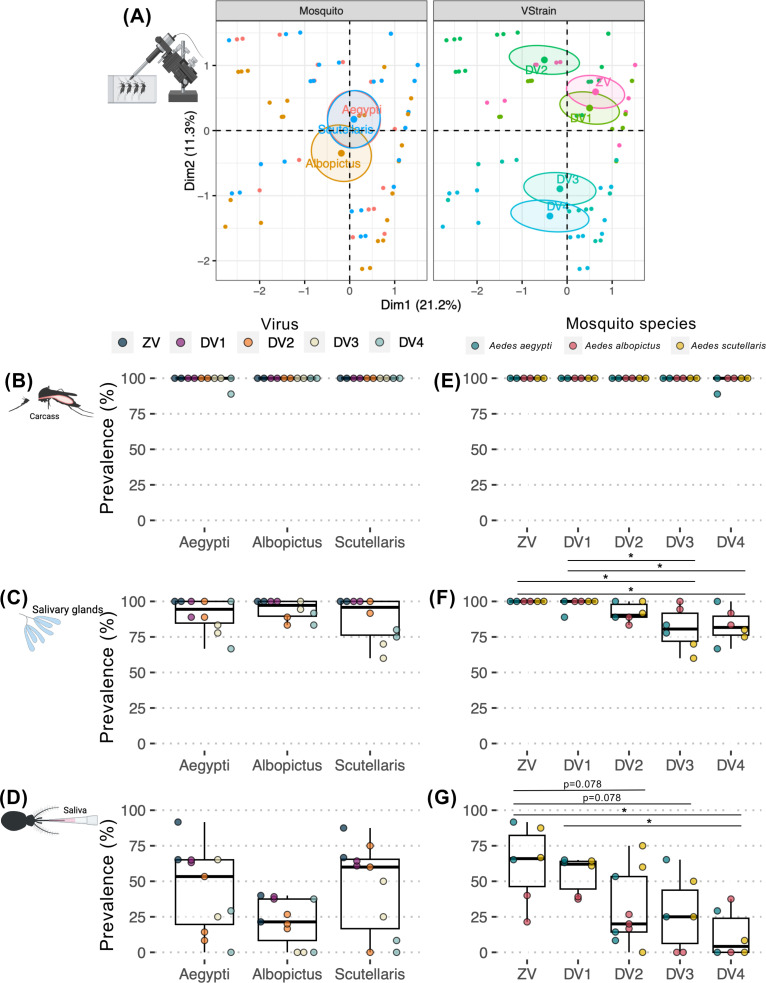
Comparison of ZIKV and DENV1-4 infection prevalence in different infection barriers of *Ae. aegypti*, *Ae. albopictus*, and *Ae. scutellaris* after intrathoracic injection. (A) FAMD factor maps to visualize variance between prevalence from each intrathoracic injection experiment on the transformed coordinates. Each dot represents prevalence from all the infection experiments. The information in the analysis included median infection prevalence, mosquito species (Mosquito), and virus (VStrain). The clustering is based on qualitative coordinates (mosquito species, tissue and virus), and each color represents individuals in a specific feature. Cluster distance represents the correlation among variables. The confidence ellipses indicate their potential relationships. Abbreviation: Dim, dimension. Box and scatter plot comparing infection prevalence of individual mosquito in the (B) carcasses at 4 dpit, (C) salivary glands at 4 dpit, and (D) saliva at 11 dpit, grouping by mosquito species and virus. Each dot represents infection prevalence from each intrathoracic injection experiment. The boxes in the plots represent the interquartile range, and the whiskers indicate the range of maximum and minimum values, excluding outliers (less than Q1 - 1.5 X IQR, or more than Q3 + 1.5 X IQR). Statistical analysis comparing infection level was conducted using Kruskal-Wallis followed by Dunn’s post-hoc test in R. *: p < 0.05. Non-statistically significant results with p ≤ 0.1 were reported as numerical values. All the infection experiments were done in at least two replicates (minimum 12 mosquitoes per replicate). The image was created in BioRender (https://BioRender.com/p28g278).

The FAMD analysis of the infection prevalence data following intrathoracic injection revealed that the clusters of *Ae. scutellaris* and *Ae. aegypti* were almost identical suggesting similar infection prevalence patterns between the two mosquitoes (Fig 5A). In contrast to the blood feeding experiment, which demonstrated a lower infection and transmissibility of *Ae. scutellaris* compared to *Ae. aegypti*, we found that the infection prevalence of arbo-orthoflaviviruses of these two *Aedes* species were comparable throughout the mosquito transmission cycle (Fig 5B-D). While *Ae. albopictus* had similar infection prevalence in the body and salivary glands to the other two *Aedes* species, viral prevalence in saliva was significantly lower. Specifically, only 21.4% of *Ae. albopictus* had detectable virus in saliva, compared to 53.3% in *Ae. aegypti* and 60% in *Ae. scutellaris* (Fig 5D). This demonstrated a stronger salivary gland escape barrier in *Ae. albopictus* than the other two *Aedes* species.

When comparing infection prevalence between viruses following intrathoracic injection, ZIKV was consistently the most infectious virus across all tissues examined (Fig 5E-G). Among DENV serotypes, while DENV3 had the highest infection prevalence in the carcass and salivary glands following blood feeding, DENV1 became the most infectious DENV following intrathoracic injection, followed by DENV2, then DENV3 and DENV4 in that order (Fig 5E-F). At the transmission step, the infection prevalence ranking followed the order of DENV1 > DENV2 ~ DENV3 > DENV4, with DENV4 exhibiting the lowest prevalence in saliva. These findings align with those from the blood-feeding experiments, confirming that DENV3 and DENV4 exhibit lower infectivity during virus propagation and tissue invasion. This highlights serotype-specific differences in intra-host viral dynamics, which may influence vector competence and transmission efficiency across different *Aedes* species.

**Fig 6:**
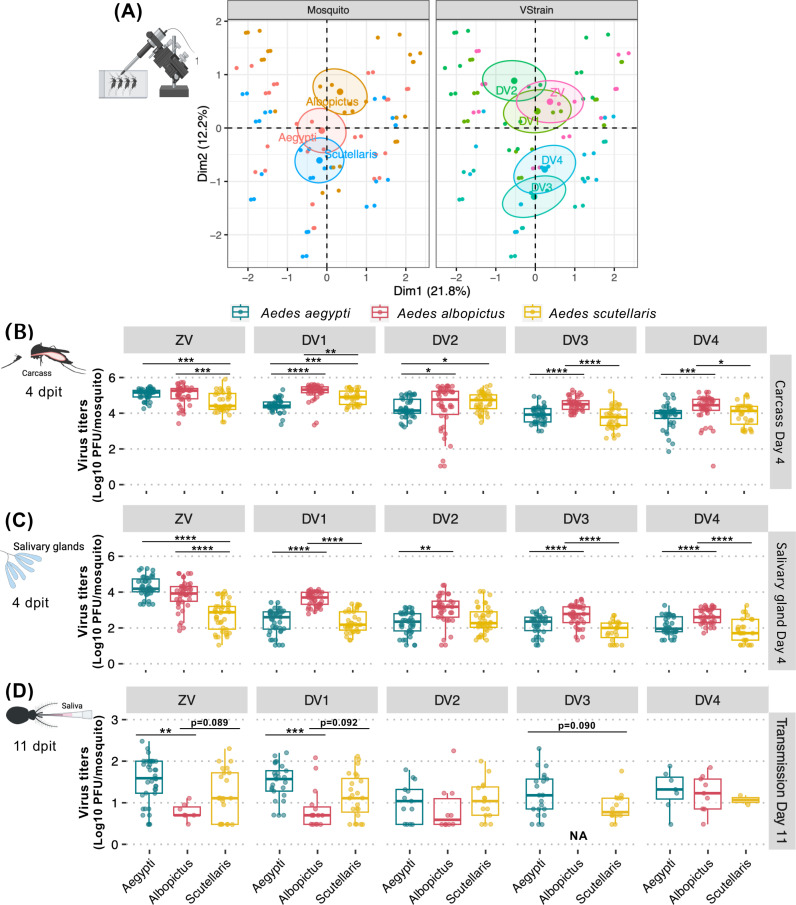
Comparison of ZIKV and DENV1-4 titers in different infection barriers of *Ae. aegypti*, *Ae. albopictus*, and *Ae. scutellaris* after intrathoracic injection (A) FAMD factor maps to visualize variance between median infection titers from intrathoracic injection experiment on the transformed coordinates. Each dot represents median infection value from all the infection experiments. The information in the analysis included median infection titers, mosquito species (Mosquito), and virus (VStrain). The clustering is based on qualitative coordinates (mosquito species, tissue and virus), and each color represents individuals in a specific feature. Cluster distance represents the correlation among variables. The confidence ellipses indicate their potential relationships. Abbreviation: Dim, dimension. Box and scatter plot comparing infection titers of individual mosquito in the (B) carcasses at 4 dpit, (C) salivary glands at 4 dpit, and (D) saliva at 11 dpit, grouping by mosquito species and virus. Each dot represents infection titers from each intrathoracic injection experiment. The boxes in the plots represent the interquartile range, and the whiskers indicate the range of maximum and minimum values, excluding outliers (less than Q1 - 1.5 X IQR, or more than Q3 + 1.5 X IQR). Statistical analysis comparing infection level was conducted using Kruskal-Wallis followed by Dunn’s post-hoc test in R. *: p < 0.05, **: p < 0.01, ***: *p* < 0.001, and ****: *p* < 0.0001. All the infection experiments were done in at least two replicates (minimum 12 mosquitoes per replicate). The image was created in BioRender (https://BioRender.com/p28g278).

Unlike the infection prevalence, the FAMD analysis of median titers revealed distinct patterns of infection titers among the three *Aedes* species (Fig 6A). When comparing infection titers to *Ae. aegypti*, we found that *Ae. scutellaris* supported lower levels of ZIKV replication in the body and salivary glands but exhibited similar ZIKV titers in the saliva (Fig 6B-D). This suggests that *Ae. scutellaris* has a low ZIKV salivary gland escape barrier, allowing efficient virus release into saliva despite lower overall viral loads in other tissues.

For DENV, *Ae. scutellaris* exhibited similar levels of virus propagation following intrathoracic inoculation in the body compared to *Ae. aegypti*, ultimately resulting in comparable saliva virus titers (Fig 6B-D). In contrast, *Ae. albopictus* demonstrated the most robust DENV replication in the body and salivary glands (Fig 6B-C), but its saliva titers varied by serotype (Fig 6D). Specifically, *Ae. albopictus* exhibited lower DENV1 saliva titers compared to the other two species, while having comparable titers for DENV2 and DENV4 (Fig 6D). Due to the absence of virus-positive samples in *Ae. albopictus* saliva for DENV3, we were unable to directly compare its saliva titers to those of *Ae. aegypti* and *Ae. scutellaris* (Fig 6D).

## 4 Discussion

To evaluate the potential role of *Ae. scutellaris* in DENV and ZIKV transmission, we compared the intra-host infection kinetics of these viruses in a recently colonized *Ae. scutellaris* with those of laboratory colonies of *Ae. aegypti* and *Ae. albopictus*, which are known to have high arbo-orthoflavivirus transmissibility. In fact, the laboratory *Ae. aegypti* strain was the most transmissible mosquito we have in our laboratory. The comparison to transmissibility of *Ae. scutellaris* to this highly transmissible strain thus highlight the transmission potential of *Ae. scutellaris*. Our research provides extensive insights into the vector competence of *Ae. scutellaris* for dengue and Zika viruses in relation to the more studied *Ae. aegypti* and *Ae. albopictus*, offering a deeper understanding of the potential role this mosquito species plays in the spread of the viruses.

Our findings suggest that blood meal size, which determines the initial inoculum size, may influence the establishment of midgut infection, a critical barrier for arbovirus transmission. Among the three species, *Ae. aegypti* exhibited the highest midgut infection prevalence, likely correlated with its larger blood meal size. However, we cannot exclude the possibility that *Ae. scutellaris* midguts are intrinsically less susceptible to arbo-orthoflaviviruses, regardless of inoculum size. Future experiments normalizing the viral dose across species by accounting for differences in blood meal volume would help elucidate the relative contributions of inoculum size versus midgut susceptibility to these differences. Interestingly, despite *Ae. scutellaris* acquiring significantly smaller blood meals compared to *Ae. albopictus*, the midgut infection prevalence was similar, suggesting that *Ae. scutellaris* midguts may be more permissive than those of *Ae. albopictus*. The midgut infection comprises of multiple steps including primary infection of virus from the blood meal to midgut epithelial cells, the replication of viruses in the primary infected cells and the spread of virus to other cells (secondary infection) [19]. Although our study did not pinpoint the specific step of midgut infection that constitutes a bottleneck for *Ae. scutellaris* and *Ae. albopictus*, the observation that midgut virus titers eventually reached comparable levels across the three species at later time points suggests that the primary barrier lies during the initial stages of the establishment of midgut infection of the virus in the blood meal.

Although not surprising, our study provided experimental evidence on the effect of blood meal size on vector competence. The size of blood meal determines the amount of virus initiating midgut infection, which directly determine the infection prevalence. In engorged mosquitoes, the smaller blood meal size decreased the number of virus initiating midgut infection, which has previously been demonstrated to influence the level of persistent infection across the tissues [19,25]. It is interesting to note that the comparative blood meal size between mosquito species and population has rarely been investigated despite its relevance to vector competence and vectorial capacity. In addition to vector competence, the differences in blood meal size may also determine the fecundity of female mosquitoes, thereby affecting mosquito population density. Our observations incite further investigation on the host and environmental factors determining blood meal size and the effect of blood meal size on mosquito biology.

Following the midgut infection, the infection prevalences in carcasses and salivary glands among the three *Aedes* species followed the same patterns as that in the midguts, with *Ae. aegypti* being the most permissible while *Ae. scutellaris* being the least permissible mosquitoes. These findings indicate that midgut infection serves as a critical bottleneck in the arbovirus transmission cycle, as successful viral establishment in the midgut is a key determinant for subsequent steps of transmission cycle.

The most concerning phenotype of *Ae. scutellaris* is its ability to support ZIKV and DENV replication and its low barrier for crossing tissue boundaries. In intrathoracic injection experiments, *Ae. scutellaris* demonstrated comparable transmissibility to *Ae. aegypti* despite having lower ZIKV titers in the salivary glands. Similarly, for DENV serotypes, *Ae. scutellaris* exhibited salivary gland and saliva viral titers comparable to those of *Ae. aegypti*, suggesting that it possesses a permissive transmission profile across multiple arboviruses.

While our study demonstrates the less permissive *Ae. albopictus* midgut for the establishment of midgut infection, there have been contradicting results regarding ZIKV and DENV establishment of midgut infection phenotype between *Ae. albopictus* and *Ae. aegypti*. Some studies found that *Ae. albopictus* had superior ability to support establishment of midgut infection to *Ae. aegypti* [26] while the others showed similar establishment of midgut infection [27,28] or the inferior midgut infection [29–32]. These contradicting results demonstrate varying degrees of midgut permissiveness of different mosquito populations from the same species. Regardless of the midgut infectivity, most of these studies demonstrated similar or superior propagation of ZIKV and DENV in the tissues of *Ae. albopictus.* However, despite the high level of virus titers in the tissues, the salivary gland escape barrier is likely a major limitation for ZIKV and DENV transmission by *Ae. albopictus*.

In addition to comparing vector competence phenotypes within mosquito species, we also examined the intra-host infection kinetics of ZIKV and DENV1–4 by standardizing blood meal or intrathoracic injection titers. Our findings reveal striking differences in the infection kinetics within the midgut, carcass, and salivary glands among the viruses studied, suggesting tissue-specific infection phenotypes for each virus. Specifically, ZIKV was the most infectious and transmissible virus compared to the DENVs. The virus was very efficient in establishment of midgut infection, propagation in secondary tissues and crossing intra-host infection boundaries, which eventually leads to the highest transmissibility. Despite a high transmissibility of ZIKV, the number of reported ZIKV cases in Thailand has been only a fraction of DENV cases suggesting that there might be other factors that limit ZIKV outbreak such as herd immunity in human populations [33] or the interaction with other co-circulating viruses [34]. Another explanation is that ZIKV surveillance is much more limited than DENVs thus the reported number did not reflect a real number of infections. Among DENVs, the establishment of midgut infection was the highest in DENV3 followed by DENV1, DENV4 then DENV2. Our results were different from a previous comprehensive intra-host infection kinetics by Novelo et al. [25], which showed that DENV1 and DENV2 had higher rates of midgut infection than DENV3 and DENV4 in terms of the establishment of midgut infection. Interestingly, while DENV3 efficiently established midgut infection, its replication rate within the mosquito may be lower during subsequent steps of infection. This is suggested by the gradual reduction in differences between DENV3 and DENV1/DENV2 infection levels in the carcasses and salivary glands. In addition to DENV3, DENV4 also showed low replication rate in the midguts and salivary glands, which was similar to what previously observed [25]. The differences in the infection pattern observed between our study and previous studies highlighted the influence of vector and virus genotypes on the infection phenotypes [35,36].

It should be noted that, for practical reasons, our study conducted the infection experiments with only one strain per virus. Except for ZIKV that we used a local strain, the DENVs used in this study were the contemporary DENV panel freely available from BEI Resources consisting of DENV1 UIS 998, DENV2 US/BID-V594/2006, DENV3 US/BID-V1043/2006, and DENV4 UIS 497. The reason why we opted for these DENVs was to allow comparison between our results and other infection studies in the future. Due to limited number of virus genotype used, it is possible that there may be specific interactions between mosquito and virus genotypes that influence infection outcome. Future works investigating the transmissibility of local mosquitoes and virus strains coupled with evaluation of vector population density in Zika and dengue endemic areas will provide a more comprehensive understanding on the role of each mosquito species in local virus transmission. Thailand offers a unique setting to conduct such future investigation due to an availability of weekly dengue case surveillance to district levels as well as research infrastructure.

The significance of our research is underscored by the potential of *Ae. scutellaris* in arbovirus circulation and outbreaks, especially considering changing climate patterns. Most existing studies on the geographical distribution of *Ae. scutellaris* are over a decade old [12,14,15], yet they similarly identified its high arbovirus transmission potential, consistent with our findings. Given this and the evolving environmental landscape, there is a critical need for updated investigations into its current distribution and possible variations in vector competence to better assess its role in future arbovirus dynamics. In addition to *Ae. scutellaris*, it is crucial to explore the vector competence of other members within the Scutellaris subgroup. While several studies have investigated the vector competence of *Ae. polynesiensis*, identifying its role as a vector for DENV, CHIKV, and ZIKV in the Pacific region [11,37], the potential involvement of other members in arbovirus transmission remains unestablished. Recognizing and understanding the role of this neglected vector species contributes not only to our understanding of disease transmission but also informs the development of effective vector control strategies.

## Supporting information

S1 File Sequencing and Blastn results of Cytochrome Oxidase I (COI) from colonized *Aedes scutellaris.* (DOCX)

S1 Table Virus titers of all samples in our infection study. (CSV)

S2 Table Summary statistics of infection titers for each experimental group. (XLSX)

S3 Table Summary of experimental design and sample size. (XLSX)

S4 Table Summary statistics of infection prevalence, mean, median, and standard deviation for each replicate. (XLSX)

S1 Fig ZIKV transmissibility of Thai *Ae. aegypti* and *Ae. albopictus* populations. Box and scatter plot comparing virus titers in saliva at 14 dpibm. Each dot represents titers in saliva of each individual mosquito. The boxes in the plots represent the interquartile range, and the whiskers indicate the range of maximum and minimum values, excluding outliers (less than Q1 - 1.5 X IQR, or more than Q3 + 1.5 X IQR). The *Ae. aegypti* strains consist of two laboratory strains: DMSC and LVP and three field strains: NAK, CSP and BTY. The *Ae. albopictus* strains consist of one laboratory strain TH and one field strain SNS. The data were summarized from one blood feeding experiment with at least 30 mosquitoes per group. Statistical analysis comparing infection level was conducted using Kruskal-Wallis followed by Dunn’s post-hoc test for *Ae. aegypti* and Wilcoxon rank sum test for *Ae. albopictus*. *: p < 0.05.
(TIFF)

S2 Fig *Wolbachia* detection in *Ae. scutellaris.* The presence of *Wolbachia* was detected by PCR of *wsp* gene. None of the *Ae. scutellaris* samples were infected by *Wolbachia* while the *wsp* gene was detected in *Ae. albopictus* and *Culex quinquefasciatus* used as positive controls.
(TIFF)
