# Supplementary material for: Comprehensive intra-host infection kinetics reveals high arbo-orthoflavivirus transmission potential by neglected vector species, Aedes scutellaris
Source: PLoS Negl Trop Dis. 2025 May 6;19(5):e0012530. doi: 10.1371/journal.pntd.0012530 (PMC12080922; doi:10.1371/journal.pntd.0012530)
Supplement: S1 File — (DOCX) [file pntd.0012530.s001.docx]

**Scutellaris COI sequencing result**

AAGCCTATTTTTGGAGTATGATCGGGGAAGTCGGAACTTCATTAAGAGTTTTAATTCGAACTGAACTTAGTCACCCTGGAATATTTATCGGAAATGATCAAATTTATAATGTAATTCGTTACTAGCTCATGCATTTATTATAATTTTTTTTATAGTAATACCTATTATAATTGGAGGATTTGGAAATTGGTTAGTCCCATTAATATTAGGTGCTCCTGATATAGCTTTCCCTCGAATAAATAATATAAGTTTTTGAATACTTCCTCCCTCTTTAACTCTTTTAATTTCTAGATCTATAGTAGAAAATGGAGCAGGAACAGGTTGAACTGTATACCCTCCTCTTTCTTCTGGAACTGCTCATGCAGGAGCTTCTGTAGATTTAGCTATTTTTTCTCTTCATTTAGCAGGAATTTCTTCTATTTTAGGAGCAGTAAATTTTATTACAACTGTAATTAATATACGATCAACTGGAATTACACTTGATCGTTTACCTTTATTTGTCTGATCTGTAATTATTACAGCTATTTTATTACTTCTTTCATTACCAGTTTTAGCAGGAGCTATTACTATATTATTAACTGATCGAAATTTTAATACATCATTTTTTGACCCAATTGGAGGAGGTGACCCTATTCTTTATCAACATCTATTTTGATTTTTTGGACATCGCAAAGATTATAAAAAAT

**Scutellaris COI Blastn results**

RID: DAFB0S7A013

Job Title:Benchling Query: Scu_CS1-Lep-F1

Program: BLASTN

Database: nt Nucleotide collection (nt)

Query #1: Query ID: lcl|Query_6193891 Length: 686

Sequences producing significant alignments:

Scientific Common Max Total Query E Per. Acc.

Description Name Name Taxid Score Score cover Value Ident Len Accession

Aedes scutellaris isolate NC-061 cytochrome oxidase subunit 1... Aedes scutel... NA 373850 1197 1197 95% 0.0 99.54 688 MN733757.1

Aedes scutellaris isolate NC-033 cytochrome oxidase subunit 1... Aedes scutel... NA 373850 1197 1197 95% 0.0 99.54 699 MN733755.1

Aedes scutellaris isolate NC-072 cytochrome oxidase subunit 1... Aedes scutel... NA 373850 1197 1197 95% 0.0 99.54 707 MN733754.1

Aedes scutellaris isolate NC-035 cytochrome oxidase subunit 1... Aedes scutel... NA 373850 1194 1194 95% 0.0 99.39 689 MN733756.1

Aedes scutellaris isolate NC-099 cytochrome oxidase subunit 1... Aedes scutel... NA 373850 1186 1186 95% 0.0 99.24 715 MN733758.1

Aedes malayensis voucher Aedes_malayensis_S45_102A cytochrome ... Aedes malaye... NA 1424567 1182 1182 95% 0.0 99.39 677 MW321935.1

Aedes scutellaris isolate scu1 cytochrome oxidase subunit 1... Aedes scutel... NA 373850 1177 1177 94% 0.0 99.54 658 KP843372.1

Aedes malayensis voucher Aedes_malayensis_KR41_L4_4 cytochrome... Aedes malaye... NA 1424567 1177 1177 95% 0.0 99.24 677 MW321934.1

Aedes malayensis voucher DNUS-WCP5 cytochrome c oxidase subuni... Aedes malaye... NA 1424567 1171 1171 94% 0.0 99.38 656 KY420768.1

Aedes malayensis voucher DNUS-Sentosa1 cytochrome c oxidase... Aedes malaye... NA 1424567 1171 1171 94% 0.0 99.38 657 KY420767.1

Aedes malayensis voucher DNUS-WCP10 cytochrome c oxidase subun... Aedes malaye... NA 1424567 1171 1171 94% 0.0 99.38 658 KY420726.1

Aedes malayensis voucher DNUS-Clementi9 cytochrome c oxidase... Aedes malaye... NA 1424567 1171 1171 94% 0.0 99.38 658 KY420725.1

Aedes malayensis voucher Aedes_malayensis_S39_109A cytochrome ... Aedes malaye... NA 1424567 1171 1171 95% 0.0 99.08 677 MW321936.1

Aedes malayensis voucher DNUS-Sentosa3 cytochrome c oxidase... Aedes malaye... NA 1424567 1166 1166 94% 0.0 99.23 658 KY420776.1

Aedes malayensis voucher DNUS-ECP1-3 cytochrome c oxidase... Aedes malaye... NA 1424567 1166 1166 94% 0.0 99.23 658 KY420770.1

Aedes albopictus isolate AB1S1_04B cytochrome oxidase subunit ... Aedes albopi... Asian tiger ... 7160 1164 1164 93% 0.0 99.53 639 KM613084.1

Aedes malayensis voucher DNUS-WCP4 cytochrome c oxidase subuni... Aedes malaye... NA 1424567 1162 1162 94% 0.0 99.23 661 KY420724.1

Aedes malayensis voucher DNUS-Clementi1 cytochrome c oxidase... Aedes malaye... NA 1424567 1160 1160 94% 0.0 99.07 657 KY420775.1

Aedes malayensis voucher DNUS-Sentosa8 cytochrome c oxidase... Aedes malaye... NA 1424567 1160 1160 94% 0.0 99.07 658 KY420772.1

Aedes albopictus isolate AB1S5_inB cytochrome oxidase subunit ... Aedes albopi... Asian tiger ... 7160 1158 1158 93% 0.0 99.38 639 KM613085.1

Aedes albopictus isolate AB1_04B cytochrome oxidase subunit 1... Aedes albopi... Asian tiger ... 7160 1158 1158 93% 0.0 99.38 639 KM613083.1

Aedes scutellaris isolate scu2 cytochrome oxidase subunit 1... Aedes scutel... NA 373850 1149 1149 94% 0.0 98.77 658 KP843373.1

Aedes malayensis isolate NN519 cytochrome c oxidase subunit 1... Aedes malaye... NA 1424567 1144 1144 94% 0.0 98.61 658 MG921173.1

Aedes malayensis voucher DNUS-WCP8 cytochrome c oxidase subuni... Aedes malaye... NA 1424567 1140 1140 92% 0.0 99.06 635 KY420813.1

Aedes albopictus isolate AA004CK cytochrome oxidase subunit 1... Aedes albopi... Asian tiger ... 7160 1136 1136 93% 0.0 98.75 639 KM613121.1

Aedes malayensis voucher DNUS-Sentosa4 cytochrome c oxidase... Aedes malaye... NA 1424567 1133 1133 90% 0.0 99.52 621 KY420769.1

Aedes scutellaris voucher AS107 cytochrome c oxidase subunit I... Aedes scutel... NA 373850 1125 1125 90% 0.0 99.52 618 MW664823.1

Aedes scutellaris voucher AS072 cytochrome c oxidase subunit I... Aedes scutel... NA 373850 1125 1125 90% 0.0 99.52 618 MW664822.1

Aedes scutellaris voucher AS105 cytochrome c oxidase subunit I... Aedes scutel... NA 373850 1125 1125 90% 0.0 99.52 618 MW664821.1

Aedes scutellaris voucher AS038 cytochrome c oxidase subunit I... Aedes scutel... NA 373850 1125 1125 90% 0.0 99.52 618 MW664820.1

Aedes scutellaris voucher AS034 cytochrome c oxidase subunit I... Aedes scutel... NA 373850 1125 1125 90% 0.0 99.52 618 MW664819.1

Aedes scutellaris voucher AS032 cytochrome c oxidase subunit I... Aedes scutel... NA 373850 1125 1125 90% 0.0 99.52 618 MW664818.1

Aedes scutellaris voucher AS112 cytochrome c oxidase subunit I... Aedes scutel... NA 373850 1125 1125 90% 0.0 99.52 618 MW664817.1

Aedes scutellaris voucher AS111 cytochrome c oxidase subunit I... Aedes scutel... NA 373850 1125 1125 90% 0.0 99.52 618 MW664816.1

Aedes scutellaris voucher AS110 cytochrome c oxidase subunit I... Aedes scutel... NA 373850 1125 1125 90% 0.0 99.52 618 MW664815.1

Aedes scutellaris voucher AS029 cytochrome c oxidase subunit I... Aedes scutel... NA 373850 1125 1125 90% 0.0 99.52 618 MW664814.1

Aedes malayensis voucher LA081 cytochrome c oxidase subunit I... Aedes malaye... NA 1424567 1092 1092 90% 0.0 98.55 618 MW664763.1

Aedes malayensis voucher LA080 cytochrome c oxidase subunit I... Aedes malaye... NA 1424567 1092 1092 90% 0.0 98.55 618 MW664762.1

Aedes riversi mitochondrial COI gene for cytochrome oxidase... Aedes riversi NA 373849 1055 1055 94% 0.0 96.14 658 AB738098.1

Aedes riversi mitochondrial COI gene for cytochrome oxidase... Aedes riversi NA 373849 1050 1050 94% 0.0 95.99 658 AB738253.1

Aedes riversi mitochondrial COI gene for cytochrome oxidase... Aedes riversi NA 373849 1044 1044 93% 0.0 95.97 650 LC054394.1

Aedes riversi mitochondrial COI gene for cytochrome oxidase... Aedes riversi NA 373849 1044 1044 93% 0.0 95.97 650 LC054391.1

Aedes riversi mitochondrial COI gene for cytochrome oxidase... Aedes riversi NA 373849 1044 1044 94% 0.0 95.83 658 AB738283.1

Aedes riversi mitochondrial COI gene for cytochrome oxidase... Aedes riversi NA 373849 1044 1044 94% 0.0 95.83 658 AB738131.1

Aedes riversi mitochondrial COI gene for cytochrome oxidase... Aedes riversi NA 373849 1044 1044 94% 0.0 95.83 658 AB738126.1

Aedes riversi mitochondrial COI gene for cytochrome oxidase... Aedes riversi NA 373849 1038 1038 93% 0.0 95.81 650 LC054393.1

Aedes riversi mitochondrial COI gene for cytochrome oxidase... Aedes riversi NA 373849 1038 1038 93% 0.0 95.81 650 LC054392.1

Aedes riversi mitochondrial COI gene for cytochrome oxidase... Aedes riversi NA 373849 1038 1038 93% 0.0 95.81 650 LC054390.1

Aedes riversi mitochondrial COI gene for cytochrome oxidase... Aedes riversi NA 373849 1038 1038 94% 0.0 95.68 658 AB738307.1

Aedes riversi mitochondrial COI gene for cytochrome oxidase... Aedes riversi NA 373849 1038 1038 94% 0.0 95.68 658 AB738193.1

Aedes riversi mitochondrial COI gene for cytochrome oxidase... Aedes riversi NA 373849 1038 1038 94% 0.0 95.68 658 AB738123.1

Aedes riversi mitochondrial COI gene for cytochrome oxidase... Aedes riversi NA 373849 1038 1038 94% 0.0 95.69 658 AB738116.1

Aedes riversi mitochondrial COI gene for cytochrome oxidase... Aedes riversi NA 373849 1022 1022 94% 0.0 95.22 658 AB738295.1

Aedes daitensis mitochondrial COI gene for cytochrome oxidase... Aedes daitensis NA 742472 1022 1022 94% 0.0 95.22 658 AB738096.1

Culicidae sp. sc_02090 cytochrome oxidase subunit 1 (COI) gene... Culicidae sp... NA 1900197 961 961 94% 0.0 93.52 658 KX052479.1

Toxorhynchites amboinensis voucher ICBRIRI-0002 cytochrome c... Toxorhynchit... NA 46208 959 959 96% 0.0 92.90 669 JQ235743.1

Culicidae sp. sc_01327 cytochrome oxidase subunit 1 (COI) gene... Culicidae sp... NA 1900193 955 955 94% 0.0 93.36 658 KX052485.1

Culicidae sp. sc_05594 cytochrome oxidase subunit 1 (COI) gene... Culicidae sp... NA 1900212 955 955 94% 0.0 93.36 658 KX052482.1

Culicidae sp. sc_02856 cytochrome oxidase subunit 1 (COI) gene... Culicidae sp... NA 1900202 946 946 93% 0.0 93.31 653 KX052491.1

Culicidae sp. sc_05401 cytochrome oxidase subunit 1 (COI) gene... Culicidae sp... NA 1900210 942 942 93% 0.0 93.29 651 KX052486.1

Culicidae sp. sc_06676 cytochrome oxidase subunit 1 (COI) gene... Culicidae sp... NA 1900217 941 941 93% 0.0 93.16 653 KX052506.1

Culicidae sp. sc_00010 cytochrome oxidase subunit 1 (COI) gene... Culicidae sp... NA 1900190 933 933 92% 0.0 93.23 647 KX052496.1

Aedes polynesiensis voucher WA068 cytochrome c oxidase subunit... Aedes polyne... NA 188700 920 920 90% 0.0 93.55 618 MW664787.1

Aedes polynesiensis voucher WA063 cytochrome c oxidase subunit... Aedes polyne... NA 188700 920 920 90% 0.0 93.55 618 MW664783.1

Aedes polynesiensis voucher WA062 cytochrome c oxidase subunit... Aedes polyne... NA 188700 920 920 90% 0.0 93.55 618 MW664782.1

Aedes polynesiensis voucher WA042 cytochrome c oxidase subunit... Aedes polyne... NA 188700 920 920 90% 0.0 93.55 618 MW664775.1

Aedes polynesiensis voucher WA067 cytochrome c oxidase subunit... Aedes polyne... NA 188700 915 915 90% 0.0 93.39 618 MW664786.1

Aedes polynesiensis voucher WA065 cytochrome c oxidase subunit... Aedes polyne... NA 188700 915 915 90% 0.0 93.39 618 MW664785.1

Aedes polynesiensis voucher WA055 cytochrome c oxidase subunit... Aedes polyne... NA 188700 915 915 90% 0.0 93.39 618 MW664780.1

Aedes polynesiensis voucher WA041 cytochrome c oxidase subunit... Aedes polyne... NA 188700 915 915 90% 0.0 93.39 618 MW664774.1

Aedes polynesiensis voucher WA038 cytochrome c oxidase subunit... Aedes polyne... NA 188700 915 915 90% 0.0 93.39 618 MW664773.1

Aedes polynesiensis voucher WA037 cytochrome c oxidase subunit... Aedes polyne... NA 188700 915 915 90% 0.0 93.39 618 MW664772.1

Aedes polynesiensis voucher WA036 cytochrome c oxidase subunit... Aedes polyne... NA 188700 915 915 90% 0.0 93.39 618 MW664771.1

Aedes pseudoscutellaris voucher FI328 cytochrome c oxidase... Aedes pseudo... NA 316597 909 909 90% 0.0 93.23 618 MW664801.1

Aedes pseudoscutellaris voucher FI006 cytochrome c oxidase... Aedes pseudo... NA 316597 909 909 90% 0.0 93.23 618 MW664798.1

Aedes polynesiensis voucher WA069 cytochrome c oxidase subunit... Aedes polyne... NA 188700 909 909 90% 0.0 93.23 618 MW664788.1

Aedes polynesiensis voucher WA064 cytochrome c oxidase subunit... Aedes polyne... NA 188700 909 909 90% 0.0 93.23 618 MW664784.1

Aedes polynesiensis voucher WA060 cytochrome c oxidase subunit... Aedes polyne... NA 188700 909 909 90% 0.0 93.23 618 MW664781.1

Aedes polynesiensis voucher WA054 cytochrome c oxidase subunit... Aedes polyne... NA 188700 909 909 90% 0.0 93.23 618 MW664779.1

Aedes polynesiensis voucher WA053 cytochrome c oxidase subunit... Aedes polyne... NA 188700 909 909 90% 0.0 93.23 618 MW664778.1

Aedes polynesiensis voucher WA048 cytochrome c oxidase subunit... Aedes polyne... NA 188700 909 909 90% 0.0 93.23 618 MW664777.1

Aedes polynesiensis voucher WA046 cytochrome c oxidase subunit... Aedes polyne... NA 188700 909 909 90% 0.0 93.23 618 MW664776.1

Aedes polynesiensis voucher WA034 cytochrome c oxidase subunit... Aedes polyne... NA 188700 909 909 90% 0.0 93.23 618 MW664770.1

Aedes polynesiensis voucher WA033 cytochrome c oxidase subunit... Aedes polyne... NA 188700 909 909 90% 0.0 93.23 618 MW664769.1

Aedes polynesiensis voucher WA029 cytochrome c oxidase subunit... Aedes polyne... NA 188700 909 909 90% 0.0 93.23 618 MW664768.1

Aedes polynesiensis voucher WA027 cytochrome c oxidase subunit... Aedes polyne... NA 188700 909 909 90% 0.0 93.23 618 MW664767.1

Aedes polynesiensis voucher WA026 cytochrome c oxidase subunit... Aedes polyne... NA 188700 909 909 90% 0.0 93.23 618 MW664766.1

Aedes polynesiensis voucher WA025 cytochrome c oxidase subunit... Aedes polyne... NA 188700 909 909 90% 0.0 93.23 618 MW664765.1

Aedes polynesiensis voucher WA024 cytochrome c oxidase subunit... Aedes polyne... NA 188700 909 909 90% 0.0 93.23 618 MW664764.1

Aedes pseudoscutellaris voucher FI272 cytochrome c oxidase... Aedes pseudo... NA 316597 904 904 90% 0.0 93.06 618 MW664812.1

Aedes pseudoscutellaris voucher FI271 cytochrome c oxidase... Aedes pseudo... NA 316597 904 904 90% 0.0 93.06 618 MW664811.1

Aedes pseudoscutellaris voucher FI205 cytochrome c oxidase... Aedes pseudo... NA 316597 904 904 90% 0.0 93.06 618 MW664810.1

Aedes pseudoscutellaris voucher FI314 cytochrome c oxidase... Aedes pseudo... NA 316597 904 904 90% 0.0 93.06 618 MW664809.1

Aedes pseudoscutellaris voucher FI163 cytochrome c oxidase... Aedes pseudo... NA 316597 904 904 90% 0.0 93.06 618 MW664808.1

Aedes pseudoscutellaris voucher FI013 cytochrome c oxidase... Aedes pseudo... NA 316597 904 904 90% 0.0 93.06 618 MW664807.1

Aedes pseudoscutellaris voucher FI221 cytochrome c oxidase... Aedes pseudo... NA 316597 904 904 90% 0.0 93.06 618 MW664806.1

Aedes pseudoscutellaris voucher FI220 cytochrome c oxidase... Aedes pseudo... NA 316597 904 904 90% 0.0 93.06 618 MW664805.1

Aedes pseudoscutellaris voucher FI012 cytochrome c oxidase... Aedes pseudo... NA 316597 904 904 90% 0.0 93.06 618 MW664804.1

Aedes pseudoscutellaris voucher FI010 cytochrome c oxidase... Aedes pseudo... NA 316597 904 904 90% 0.0 93.06 618 MW664803.1

Aedes pseudoscutellaris voucher FI219 cytochrome c oxidase... Aedes pseudo... NA 316597 904 904 90% 0.0 93.06 618 MW664802.1

Alignments:

>Aedes scutellaris isolate NC-061 cytochrome oxidase subunit 1 (COI) gene, partial cds; mitochondrial

Sequence ID: MN733757.1 Length: 688

Range 1: 16 to 672

Score:1197 bits(648), Expect:0.0,

Identities:656/659(99%), Gaps:3/659(0%), Strand: Plus/Plus

Query 6 TATTTTTGGAGTATGATCGGGGA-AGTCGGAACTTCATTAAGAGTTTTAATTCGAACTGA 64

||||||||||||||||||||||| ||||||||||||||||||||||||||||||||||||

Sbjct 16 TATTTTTGGAGTATGATCGGGGATAGTCGGAACTTCATTAAGAGTTTTAATTCGAACTGA 75

Query 65 ACTTAGTCACCCTGGAATATTTATCGGAAATGATCAAATTTATAATGTAATTCGTTACTA 124

|||||||||||||||||||||||||||||||||||||||||||||||||||| ||||||

Sbjct 76 ACTTAGTCACCCTGGAATATTTATCGGAAATGATCAAATTTATAATGTAATT-GTTACT- 133

Query 125 GCTCATGCATTTATTATAAttttttttATAGTAATACCTATTATAATTGGAGGATTTGGA 184

||||||||||||||||||||||||||||||||||||||||||||||||||||||||||||

Sbjct 134 GCTCATGCATTTATTATAATTTTTTTTATAGTAATACCTATTATAATTGGAGGATTTGGA 193

Query 185 AATTGGTTAGTCCCATTAATATTAGGTGCTCCTGATATAGCTTTCCCTCGAATAAATAAT 244

||||||||||||||||||||||||||||||||||||||||||||||||||||||||||||

Sbjct 194 AATTGGTTAGTCCCATTAATATTAGGTGCTCCTGATATAGCTTTCCCTCGAATAAATAAT 253

Query 245 ATAAGTTTTTGAATACTTCCTCCCTCTTTAACTCTTTTAATTTCTAGATCTATAGTAGAA 304

||||||||||||||||||||||||||||||||||||||||||||||||||||||||||||

Sbjct 254 ATAAGTTTTTGAATACTTCCTCCCTCTTTAACTCTTTTAATTTCTAGATCTATAGTAGAA 313

Query 305 AATGGAGCAGGAACAGGTTGAACTGTATACCCTCCTCTTTCTTCTGGAACTGCTCATGCA 364

||||||||||||||||||||||||||||||||||||||||||||||||||||||||||||

Sbjct 314 AATGGAGCAGGAACAGGTTGAACTGTATACCCTCCTCTTTCTTCTGGAACTGCTCATGCA 373

Query 365 GGAGCTTCTGTAGATTTAGCTATTTTTTCTCTTCATTTAGCAGGAATTTCTTCTATTTTA 424

||||||||||||||||||||||||||||||||||||||||||||||||||||||||||||

Sbjct 374 GGAGCTTCTGTAGATTTAGCTATTTTTTCTCTTCATTTAGCAGGAATTTCTTCTATTTTA 433

Query 425 GGAGCAGTAAATTTTATTACAACTGTAATTAATATACGATCAACTGGAATTACACTTGAT 484

||||||||||||||||||||||||||||||||||||||||||||||||||||||||||||

Sbjct 434 GGAGCAGTAAATTTTATTACAACTGTAATTAATATACGATCAACTGGAATTACACTTGAT 493

Query 485 CGTTTACCTTTATTTGTCTGATCTGTAATTATTACAGCTATTTTATTACTTCTTTCATTA 544

||||||||||||||||||||||||||||||||||||||||||||||||||||||||||||

Sbjct 494 CGTTTACCTTTATTTGTCTGATCTGTAATTATTACAGCTATTTTATTACTTCTTTCATTA 553

Query 545 CCAGTTTTAGCAGGAGCTATTACTATATTATTAACTGATCGAAATTTTAATACATCATTT 604

||||||||||||||||||||||||||||||||||||||||||||||||||||||||||||

Sbjct 554 CCAGTTTTAGCAGGAGCTATTACTATATTATTAACTGATCGAAATTTTAATACATCATTT 613

Query 605 TTTGACCCAATTGGAGGAGGTGACCCTATTCTTTATCAACATCTATTTTGATTTTTTGG 663

|||||||||||||||||||||||||||||||||||||||||||||||||||||||||||

Sbjct 614 TTTGACCCAATTGGAGGAGGTGACCCTATTCTTTATCAACATCTATTTTGATTTTTTGG 672

>Aedes scutellaris isolate NC-033 cytochrome oxidase subunit 1 (COI) gene, partial cds; mitochondrial

Sequence ID: MN733755.1 Length: 699

Range 1: 28 to 684

Score:1197 bits(648), Expect:0.0,

Identities:656/659(99%), Gaps:3/659(0%), Strand: Plus/Plus

Query 6 TATTTTTGGAGTATGATCGGGGA-AGTCGGAACTTCATTAAGAGTTTTAATTCGAACTGA 64

||||||||||||||||||||||| ||||||||||||||||||||||||||||||||||||

Sbjct 28 TATTTTTGGAGTATGATCGGGGATAGTCGGAACTTCATTAAGAGTTTTAATTCGAACTGA 87

Query 65 ACTTAGTCACCCTGGAATATTTATCGGAAATGATCAAATTTATAATGTAATTCGTTACTA 124

|||||||||||||||||||||||||||||||||||||||||||||||||||| ||||||

Sbjct 88 ACTTAGTCACCCTGGAATATTTATCGGAAATGATCAAATTTATAATGTAATT-GTTACT- 145

Query 125 GCTCATGCATTTATTATAAttttttttATAGTAATACCTATTATAATTGGAGGATTTGGA 184

||||||||||||||||||||||||||||||||||||||||||||||||||||||||||||

Sbjct 146 GCTCATGCATTTATTATAATTTTTTTTATAGTAATACCTATTATAATTGGAGGATTTGGA 205

Query 185 AATTGGTTAGTCCCATTAATATTAGGTGCTCCTGATATAGCTTTCCCTCGAATAAATAAT 244

||||||||||||||||||||||||||||||||||||||||||||||||||||||||||||

Sbjct 206 AATTGGTTAGTCCCATTAATATTAGGTGCTCCTGATATAGCTTTCCCTCGAATAAATAAT 265

Query 245 ATAAGTTTTTGAATACTTCCTCCCTCTTTAACTCTTTTAATTTCTAGATCTATAGTAGAA 304

||||||||||||||||||||||||||||||||||||||||||||||||||||||||||||

Sbjct 266 ATAAGTTTTTGAATACTTCCTCCCTCTTTAACTCTTTTAATTTCTAGATCTATAGTAGAA 325

Query 305 AATGGAGCAGGAACAGGTTGAACTGTATACCCTCCTCTTTCTTCTGGAACTGCTCATGCA 364

||||||||||||||||||||||||||||||||||||||||||||||||||||||||||||

Sbjct 326 AATGGAGCAGGAACAGGTTGAACTGTATACCCTCCTCTTTCTTCTGGAACTGCTCATGCA 385

Query 365 GGAGCTTCTGTAGATTTAGCTATTTTTTCTCTTCATTTAGCAGGAATTTCTTCTATTTTA 424

||||||||||||||||||||||||||||||||||||||||||||||||||||||||||||

Sbjct 386 GGAGCTTCTGTAGATTTAGCTATTTTTTCTCTTCATTTAGCAGGAATTTCTTCTATTTTA 445

Query 425 GGAGCAGTAAATTTTATTACAACTGTAATTAATATACGATCAACTGGAATTACACTTGAT 484

||||||||||||||||||||||||||||||||||||||||||||||||||||||||||||

Sbjct 446 GGAGCAGTAAATTTTATTACAACTGTAATTAATATACGATCAACTGGAATTACACTTGAT 505

Query 485 CGTTTACCTTTATTTGTCTGATCTGTAATTATTACAGCTATTTTATTACTTCTTTCATTA 544

||||||||||||||||||||||||||||||||||||||||||||||||||||||||||||

Sbjct 506 CGTTTACCTTTATTTGTCTGATCTGTAATTATTACAGCTATTTTATTACTTCTTTCATTA 565

Query 545 CCAGTTTTAGCAGGAGCTATTACTATATTATTAACTGATCGAAATTTTAATACATCATTT 604

||||||||||||||||||||||||||||||||||||||||||||||||||||||||||||

Sbjct 566 CCAGTTTTAGCAGGAGCTATTACTATATTATTAACTGATCGAAATTTTAATACATCATTT 625

Query 605 TTTGACCCAATTGGAGGAGGTGACCCTATTCTTTATCAACATCTATTTTGATTTTTTGG 663

|||||||||||||||||||||||||||||||||||||||||||||||||||||||||||

Sbjct 626 TTTGACCCAATTGGAGGAGGTGACCCTATTCTTTATCAACATCTATTTTGATTTTTTGG 684

>Aedes scutellaris isolate NC-072 cytochrome oxidase subunit 1 (COI) gene, partial cds; mitochondrial

Sequence ID: MN733754.1 Length: 707

Range 1: 44 to 700

Score:1197 bits(648), Expect:0.0,

Identities:656/659(99%), Gaps:3/659(0%), Strand: Plus/Plus

Query 6 TATTTTTGGAGTATGATCGGGGA-AGTCGGAACTTCATTAAGAGTTTTAATTCGAACTGA 64

||||||||||||||||||||||| ||||||||||||||||||||||||||||||||||||

Sbjct 44 TATTTTTGGAGTATGATCGGGGATAGTCGGAACTTCATTAAGAGTTTTAATTCGAACTGA 103

Query 65 ACTTAGTCACCCTGGAATATTTATCGGAAATGATCAAATTTATAATGTAATTCGTTACTA 124

|||||||||||||||||||||||||||||||||||||||||||||||||||| ||||||

Sbjct 104 ACTTAGTCACCCTGGAATATTTATCGGAAATGATCAAATTTATAATGTAATT-GTTACT- 161

Query 125 GCTCATGCATTTATTATAAttttttttATAGTAATACCTATTATAATTGGAGGATTTGGA 184

||||||||||||||||||||||||||||||||||||||||||||||||||||||||||||

Sbjct 162 GCTCATGCATTTATTATAATTTTTTTTATAGTAATACCTATTATAATTGGAGGATTTGGA 221

Query 185 AATTGGTTAGTCCCATTAATATTAGGTGCTCCTGATATAGCTTTCCCTCGAATAAATAAT 244

||||||||||||||||||||||||||||||||||||||||||||||||||||||||||||

Sbjct 222 AATTGGTTAGTCCCATTAATATTAGGTGCTCCTGATATAGCTTTCCCTCGAATAAATAAT 281

Query 245 ATAAGTTTTTGAATACTTCCTCCCTCTTTAACTCTTTTAATTTCTAGATCTATAGTAGAA 304

||||||||||||||||||||||||||||||||||||||||||||||||||||||||||||

Sbjct 282 ATAAGTTTTTGAATACTTCCTCCCTCTTTAACTCTTTTAATTTCTAGATCTATAGTAGAA 341

Query 305 AATGGAGCAGGAACAGGTTGAACTGTATACCCTCCTCTTTCTTCTGGAACTGCTCATGCA 364

||||||||||||||||||||||||||||||||||||||||||||||||||||||||||||

Sbjct 342 AATGGAGCAGGAACAGGTTGAACTGTATACCCTCCTCTTTCTTCTGGAACTGCTCATGCA 401

Query 365 GGAGCTTCTGTAGATTTAGCTATTTTTTCTCTTCATTTAGCAGGAATTTCTTCTATTTTA 424

||||||||||||||||||||||||||||||||||||||||||||||||||||||||||||

Sbjct 402 GGAGCTTCTGTAGATTTAGCTATTTTTTCTCTTCATTTAGCAGGAATTTCTTCTATTTTA 461

Query 425 GGAGCAGTAAATTTTATTACAACTGTAATTAATATACGATCAACTGGAATTACACTTGAT 484

||||||||||||||||||||||||||||||||||||||||||||||||||||||||||||

Sbjct 462 GGAGCAGTAAATTTTATTACAACTGTAATTAATATACGATCAACTGGAATTACACTTGAT 521

Query 485 CGTTTACCTTTATTTGTCTGATCTGTAATTATTACAGCTATTTTATTACTTCTTTCATTA 544

||||||||||||||||||||||||||||||||||||||||||||||||||||||||||||

Sbjct 522 CGTTTACCTTTATTTGTCTGATCTGTAATTATTACAGCTATTTTATTACTTCTTTCATTA 581

Query 545 CCAGTTTTAGCAGGAGCTATTACTATATTATTAACTGATCGAAATTTTAATACATCATTT 604

||||||||||||||||||||||||||||||||||||||||||||||||||||||||||||

Sbjct 582 CCAGTTTTAGCAGGAGCTATTACTATATTATTAACTGATCGAAATTTTAATACATCATTT 641

Query 605 TTTGACCCAATTGGAGGAGGTGACCCTATTCTTTATCAACATCTATTTTGATTTTTTGG 663

|||||||||||||||||||||||||||||||||||||||||||||||||||||||||||

Sbjct 642 TTTGACCCAATTGGAGGAGGTGACCCTATTCTTTATCAACATCTATTTTGATTTTTTGG 700

>Aedes scutellaris isolate NC-035 cytochrome oxidase subunit 1 (COI) gene, partial cds; mitochondrial

Sequence ID: MN733756.1 Length: 689

Range 1: 25 to 682

Score:1194 bits(646), Expect:0.0,

Identities:656/660(99%), Gaps:4/660(0%), Strand: Plus/Plus

Query 6 TATTTTTGGAGTATGATCGGGGA-AGTCGGAACTTCATTAAGAGTTTTAATTCGAACTGA 64

||||||||||||||||||||||| ||||||||||||||||||||||||||||||||||||

Sbjct 25 TATTTTTGGAGTATGATCGGGGATAGTCGGAACTTCATTAAGAGTTTTAATTCGAACTGA 84

Query 65 ACTTAGTCACCCTGGAATATTTATCGGAAATGATCAAATTTATAATGTAATTCGTTACTA 124

|||||||||||||||||||||||||||||||||||||||||||||||||||| ||||||

Sbjct 85 ACTTAGTCACCCTGGAATATTTATCGGAAATGATCAAATTTATAATGTAATT-GTTACT- 142

Query 125 GCTCATGCATTTATTATAAttttttttATAGTAATACCTATTATAATTGGAGGATTTGGA 184

||||||||||||||||||||||||||||||||||||||||||||||||||||||||||||

Sbjct 143 GCTCATGCATTTATTATAATTTTTTTTATAGTAATACCTATTATAATTGGAGGATTTGGA 202

Query 185 AATTGGTTAGTCCCATTAATATTAGGTGCTCCTGATATAGCTTTCCCTCGAATAAATAAT 244

||||||||||||||||||||||||||||||||||||||||||||||||||||||||||||

Sbjct 203 AATTGGTTAGTCCCATTAATATTAGGTGCTCCTGATATAGCTTTCCCTCGAATAAATAAT 262

Query 245 ATAAGTTTTTGAATACTTCCTCCCTCTTTAACTCTTTTAATTTCTAGATCTATAGTAGAA 304

||||||||||||||||||||||||||||||||||||||||||||||||||||||||||||

Sbjct 263 ATAAGTTTTTGAATACTTCCTCCCTCTTTAACTCTTTTAATTTCTAGATCTATAGTAGAA 322

Query 305 AATGGAGCAGGAACAGGTTGAACTGTATACCCTCCTCTTTCTTCTGGAACTGCTCATGCA 364

||||||||||||||||||||||||||||||||||||||||||||||||||||||||||||

Sbjct 323 AATGGAGCAGGAACAGGTTGAACTGTATACCCTCCTCTTTCTTCTGGAACTGCTCATGCA 382

Query 365 GGAGCTTCTGTAGATTTAGCTATTTTTTCTCTTCATTTAGCAGGAATTTCTTCTATTTTA 424

||||||||||||||||||||||||||||||||||||||||||||||||||||||||||||

Sbjct 383 GGAGCTTCTGTAGATTTAGCTATTTTTTCTCTTCATTTAGCAGGAATTTCTTCTATTTTA 442

Query 425 GGAGCAGTAAATTTTATTACAACTGTAATTAATATACGATCAACTGGAATTACACTTGAT 484

||||||||||||||||||||||||||||||||||||||||||||||||||||||||||||

Sbjct 443 GGAGCAGTAAATTTTATTACAACTGTAATTAATATACGATCAACTGGAATTACACTTGAT 502

Query 485 CGTTTACCTTTATTTGTCTGATCTGTAATTATTACAGCTATTTTATTACTTCTTTCATTA 544

||||||||||||||||||||||||||||||||||||||||||||||||||||||||||||

Sbjct 503 CGTTTACCTTTATTTGTCTGATCTGTAATTATTACAGCTATTTTATTACTTCTTTCATTA 562

Query 545 CCAGTTTTAGCAGGAGCTATTACTATATTATTAACTGATCGAAATTTTAAT-ACATCATT 603

||||||||||||||||||||||||||||||||||||||||||||||||||| ||||||||

Sbjct 563 CCAGTTTTAGCAGGAGCTATTACTATATTATTAACTGATCGAAATTTTAATAACATCATT 622

Query 604 TTTTGACCCAATTGGAGGAGGTGACCCTATTCTTTATCAACATCTATTTTGATTTTTTGG 663

||||||||||||||||||||||||||||||||||||||||||||||||||||||||||||

Sbjct 623 TTTTGACCCAATTGGAGGAGGTGACCCTATTCTTTATCAACATCTATTTTGATTTTTTGG 682

>Aedes scutellaris isolate NC-099 cytochrome oxidase subunit 1 (COI) gene, partial cds; mitochondrial

Sequence ID: MN733758.1 Length: 715

Range 1: 41 to 697

Score:1186 bits(642), Expect:0.0,

Identities:654/659(99%), Gaps:3/659(0%), Strand: Plus/Plus

Query 6 TATTTTTGGAGTATGATCGGGGA-AGTCGGAACTTCATTAAGAGTTTTAATTCGAACTGA 64

||||||||||||||||||||||| ||||||||||||||||||||||||||||||||||||

Sbjct 41 TATTTTTGGAGTATGATCGGGGATAGTCGGAACTTCATTAAGAGTTTTAATTCGAACTGA 100

Query 65 ACTTAGTCACCCTGGAATATTTATCGGAAATGATCAAATTTATAATGTAATTCGTTACTA 124

|||||||||||||||||||||||||||||||||||||||||||||||||||| ||||||

Sbjct 101 ACTTAGTCACCCTGGAATATTTATCGGAAATGATCAAATTTATAATGTAATT-GTTACT- 158

Query 125 GCTCATGCATTTATTATAAttttttttATAGTAATACCTATTATAATTGGAGGATTTGGA 184

||||||||||||||||||||||||||||||||||||||||||||||||||||||||||||

Sbjct 159 GCTCATGCATTTATTATAATTTTTTTTATAGTAATACCTATTATAATTGGAGGATTTGGA 218

Query 185 AATTGGTTAGTCCCATTAATATTAGGTGCTCCTGATATAGCTTTCCCTCGAATAAATAAT 244

||||||||||||||||||||||||||||||||||||||||||||||||||||||||||||

Sbjct 219 AATTGGTTAGTCCCATTAATATTAGGTGCTCCTGATATAGCTTTCCCTCGAATAAATAAT 278

Query 245 ATAAGTTTTTGAATACTTCCTCCCTCTTTAACTCTTTTAATTTCTAGATCTATAGTAGAA 304

||||||||||||||||||||||||||||||||||||||||||||||||||||||||||||

Sbjct 279 ATAAGTTTTTGAATACTTCCTCCCTCTTTAACTCTTTTAATTTCTAGATCTATAGTAGAA 338

Query 305 AATGGAGCAGGAACAGGTTGAACTGTATACCCTCCTCTTTCTTCTGGAACTGCTCATGCA 364

||||||||||||||||||||||||||||||||||||||||||||||||||||||||||||

Sbjct 339 AATGGAGCAGGAACAGGTTGAACTGTATACCCTCCTCTTTCTTCTGGAACTGCTCATGCA 398

Query 365 GGAGCTTCTGTAGATTTAGCTATTTTTTCTCTTCATTTAGCAGGAATTTCTTCTATTTTA 424

||||||||||||||||||||||||||||||||||||||||||||||||||||||||||||

Sbjct 399 GGAGCTTCTGTAGATTTAGCTATTTTTTCTCTTCATTTAGCAGGAATTTCTTCTATTTTA 458

Query 425 GGAGCAGTAAATTTTATTACAACTGTAATTAATATACGATCAACTGGAATTACACTTGAT 484

||||||||||||||||||||||||||||||||||||||||||||||||||||||||||||

Sbjct 459 GGAGCAGTAAATTTTATTACAACTGTAATTAATATACGATCAACTGGAATTACACTTGAT 518

Query 485 CGTTTACCTTTATTTGTCTGATCTGTAATTATTACAGCTATTTTATTACTTCTTTCATTA 544

||||||||||||||||||||||||||||||||||||||||||||||||||||||||||||

Sbjct 519 CGTTTACCTTTATTTGTCTGATCTGTAATTATTACAGCTATTTTATTACTTCTTTCATTA 578

Query 545 CCAGTTTTAGCAGGAGCTATTACTATATTATTAACTGATCGAAATTTTAATACATCATTT 604

||||||||||||||||||||||||||||||||||||||||||||||||||||||||||||

Sbjct 579 CCAGTTTTAGCAGGAGCTATTACTATATTATTAACTGATCGAAATTTTAATACATCATTT 638

Query 605 TTTGACCCAATTGGAGGAGGTGACCCTATTCTTTATCAACATCTATTTTGATTTTTTGG 663

||||||||| ||||||||||||||||||||||||||||| |||||||||||||||||||

Sbjct 639 TTTGACCCACTTGGAGGAGGTGACCCTATTCTTTATCAAGATCTATTTTGATTTTTTGG 697

>Aedes malayensis voucher Aedes_malayensis_S45_102A cytochrome c oxidase subunit I (COX1) gene, partial cds; mitochondrial

Sequence ID: MW321935.1 Length: 677

Range 1: 26 to 677

Score:1182 bits(640), Expect:0.0,

Identities:650/654(99%), Gaps:3/654(0%), Strand: Plus/Plus

Query 6 TATTTTTGGAGTATGATCGGGGA-AGTCGGAACTTCATTAAGAGTTTTAATTCGAACTGA 64

||||||||||||||||||||||| ||||||||||||||||||||||||||||||||||||

Sbjct 26 TATTTTTGGAGTATGATCGGGGATAGTCGGAACTTCATTAAGAGTTTTAATTCGAACTGA 85

Query 65 ACTTAGTCACCCTGGAATATTTATCGGAAATGATCAAATTTATAATGTAATTCGTTACTA 124

|||||||||||||||||||||||||||||||||||||||||||||||||||| ||||||

Sbjct 86 ACTTAGTCACCCTGGAATATTTATCGGAAATGATCAAATTTATAATGTAATT-GTTACT- 143

Query 125 GCTCATGCATTTATTATAAttttttttATAGTAATACCTATTATAATTGGAGGATTTGGA 184

||||||||||||||||||||||||||||||||||||||||||||||||||||||||||||

Sbjct 144 GCTCATGCATTTATTATAATTTTTTTTATAGTAATACCTATTATAATTGGAGGATTTGGA 203

Query 185 AATTGGTTAGTCCCATTAATATTAGGTGCTCCTGATATAGCTTTCCCTCGAATAAATAAT 244

||||||||||||||||||||||||||||||||||||||||||||||||||||||||||||

Sbjct 204 AATTGGTTAGTCCCATTAATATTAGGTGCTCCTGATATAGCTTTCCCTCGAATAAATAAT 263

Query 245 ATAAGTTTTTGAATACTTCCTCCCTCTTTAACTCTTTTAATTTCTAGATCTATAGTAGAA 304

||||||||||||||||||||||||||||||||||||||||||||||||||||||||||||

Sbjct 264 ATAAGTTTTTGAATACTTCCTCCCTCTTTAACTCTTTTAATTTCTAGATCTATAGTAGAA 323

Query 305 AATGGAGCAGGAACAGGTTGAACTGTATACCCTCCTCTTTCTTCTGGAACTGCTCATGCA 364

||||||||||||||||||||||||||||||||||||||||||||||||||||||||||||

Sbjct 324 AATGGAGCAGGAACAGGTTGAACTGTATACCCTCCTCTTTCTTCTGGAACTGCTCATGCA 383

Query 365 GGAGCTTCTGTAGATTTAGCTATTTTTTCTCTTCATTTAGCAGGAATTTCTTCTATTTTA 424

||||||||||||||||||||||||||||||||||||||||||||||||||||||||||||

Sbjct 384 GGAGCTTCTGTAGATTTAGCTATTTTTTCTCTTCATTTAGCAGGAATTTCTTCTATTTTA 443

Query 425 GGAGCAGTAAATTTTATTACAACTGTAATTAATATACGATCAACTGGAATTACACTTGAT 484

||||||||||||||||||||||||||||||||||||||||||||||||||||||||||||

Sbjct 444 GGAGCAGTAAATTTTATTACAACTGTAATTAATATACGATCAACTGGAATTACACTTGAT 503

Query 485 CGTTTACCTTTATTTGTCTGATCTGTAATTATTACAGCTATTTTATTACTTCTTTCATTA 544

||| ||||||||||||||||||||||||||||||||||||||||||||||||||||||||

Sbjct 504 CGTATACCTTTATTTGTCTGATCTGTAATTATTACAGCTATTTTATTACTTCTTTCATTA 563

Query 545 CCAGTTTTAGCAGGAGCTATTACTATATTATTAACTGATCGAAATTTTAATACATCATTT 604

||||||||||||||||||||||||||||||||||||||||||||||||||||||||||||

Sbjct 564 CCAGTTTTAGCAGGAGCTATTACTATATTATTAACTGATCGAAATTTTAATACATCATTT 623

Query 605 TTTGACCCAATTGGAGGAGGTGACCCTATTCTTTATCAACATCTATTTTGATTT 658

||||||||||||||||||||||||||||||||||||||||||||||||||||||

Sbjct 624 TTTGACCCAATTGGAGGAGGTGACCCTATTCTTTATCAACATCTATTTTGATTT 677

>Aedes scutellaris isolate scu1 cytochrome oxidase subunit 1 (COI) gene, partial cds; mitochondrial

Sequence ID: KP843372.1 Length: 658

>Aedes scutellaris isolate scu4 cytochrome oxidase subunit 1 (COI) gene, partial cds; mitochondrial

Sequence ID: KP843375.1 Length: 658

>Aedes scutellaris isolate scu5 cytochrome oxidase subunit 1 (COI) gene, partial cds; mitochondrial

Sequence ID: KP843376.1 Length: 658

>Aedes scutellaris isolate scu6 cytochrome oxidase subunit 1 (COI) gene, partial cds; mitochondrial

Sequence ID: KP843377.1 Length: 658

>Aedes scutellaris isolate scu9 cytochrome oxidase subunit 1 (COI) gene, partial cds; mitochondrial

Sequence ID: KP843380.1 Length: 658

>Aedes scutellaris isolate scu10 cytochrome oxidase subunit 1 (COI) gene, partial cds; mitochondrial

Sequence ID: KP843381.1 Length: 658

Range 1: 13 to 658

Score:1177 bits(637), Expect:0.0,

Identities:645/648(99%), Gaps:3/648(0%), Strand: Plus/Plus

Query 6 TATTTTTGGAGTATGATCGGGGA-AGTCGGAACTTCATTAAGAGTTTTAATTCGAACTGA 64

||||||||||||||||||||||| ||||||||||||||||||||||||||||||||||||

Sbjct 13 TATTTTTGGAGTATGATCGGGGATAGTCGGAACTTCATTAAGAGTTTTAATTCGAACTGA 72

Query 65 ACTTAGTCACCCTGGAATATTTATCGGAAATGATCAAATTTATAATGTAATTCGTTACTA 124

|||||||||||||||||||||||||||||||||||||||||||||||||||| ||||||

Sbjct 73 ACTTAGTCACCCTGGAATATTTATCGGAAATGATCAAATTTATAATGTAATT-GTTACT- 130

Query 125 GCTCATGCATTTATTATAAttttttttATAGTAATACCTATTATAATTGGAGGATTTGGA 184

||||||||||||||||||||||||||||||||||||||||||||||||||||||||||||

Sbjct 131 GCTCATGCATTTATTATAATTTTTTTTATAGTAATACCTATTATAATTGGAGGATTTGGA 190

Query 185 AATTGGTTAGTCCCATTAATATTAGGTGCTCCTGATATAGCTTTCCCTCGAATAAATAAT 244

||||||||||||||||||||||||||||||||||||||||||||||||||||||||||||

Sbjct 191 AATTGGTTAGTCCCATTAATATTAGGTGCTCCTGATATAGCTTTCCCTCGAATAAATAAT 250

Query 245 ATAAGTTTTTGAATACTTCCTCCCTCTTTAACTCTTTTAATTTCTAGATCTATAGTAGAA 304

||||||||||||||||||||||||||||||||||||||||||||||||||||||||||||

Sbjct 251 ATAAGTTTTTGAATACTTCCTCCCTCTTTAACTCTTTTAATTTCTAGATCTATAGTAGAA 310

Query 305 AATGGAGCAGGAACAGGTTGAACTGTATACCCTCCTCTTTCTTCTGGAACTGCTCATGCA 364

||||||||||||||||||||||||||||||||||||||||||||||||||||||||||||

Sbjct 311 AATGGAGCAGGAACAGGTTGAACTGTATACCCTCCTCTTTCTTCTGGAACTGCTCATGCA 370

Query 365 GGAGCTTCTGTAGATTTAGCTATTTTTTCTCTTCATTTAGCAGGAATTTCTTCTATTTTA 424

||||||||||||||||||||||||||||||||||||||||||||||||||||||||||||

Sbjct 371 GGAGCTTCTGTAGATTTAGCTATTTTTTCTCTTCATTTAGCAGGAATTTCTTCTATTTTA 430

Query 425 GGAGCAGTAAATTTTATTACAACTGTAATTAATATACGATCAACTGGAATTACACTTGAT 484

||||||||||||||||||||||||||||||||||||||||||||||||||||||||||||

Sbjct 431 GGAGCAGTAAATTTTATTACAACTGTAATTAATATACGATCAACTGGAATTACACTTGAT 490

Query 485 CGTTTACCTTTATTTGTCTGATCTGTAATTATTACAGCTATTTTATTACTTCTTTCATTA 544

||||||||||||||||||||||||||||||||||||||||||||||||||||||||||||

Sbjct 491 CGTTTACCTTTATTTGTCTGATCTGTAATTATTACAGCTATTTTATTACTTCTTTCATTA 550

Query 545 CCAGTTTTAGCAGGAGCTATTACTATATTATTAACTGATCGAAATTTTAATACATCATTT 604

||||||||||||||||||||||||||||||||||||||||||||||||||||||||||||

Sbjct 551 CCAGTTTTAGCAGGAGCTATTACTATATTATTAACTGATCGAAATTTTAATACATCATTT 610

Query 605 TTTGACCCAATTGGAGGAGGTGACCCTATTCTTTATCAACATCTATTT 652

||||||||||||||||||||||||||||||||||||||||||||||||

Sbjct 611 TTTGACCCAATTGGAGGAGGTGACCCTATTCTTTATCAACATCTATTT 658

>Aedes malayensis voucher Aedes_malayensis_KR41_L4_4 cytochrome c oxidase subunit I (COX1) gene, partial cds; mitochondrial

Sequence ID: MW321934.1 Length: 677

Range 1: 26 to 677

Score:1177 bits(637), Expect:0.0,

Identities:649/654(99%), Gaps:3/654(0%), Strand: Plus/Plus

Query 6 TATTTTTGGAGTATGATCGGGGA-AGTCGGAACTTCATTAAGAGTTTTAATTCGAACTGA 64

||||||||||||||||||||| | ||||||||||||||||||||||||||||||||||||

Sbjct 26 TATTTTTGGAGTATGATCGGGAATAGTCGGAACTTCATTAAGAGTTTTAATTCGAACTGA 85

Query 65 ACTTAGTCACCCTGGAATATTTATCGGAAATGATCAAATTTATAATGTAATTCGTTACTA 124

|||||||||||||||||||||||||||||||||||||||||||||||||||| ||||||

Sbjct 86 ACTTAGTCACCCTGGAATATTTATCGGAAATGATCAAATTTATAATGTAATT-GTTACT- 143

Query 125 GCTCATGCATTTATTATAAttttttttATAGTAATACCTATTATAATTGGAGGATTTGGA 184

||||||||||||||||||||||||||||||||||||||||||||||||||||||||||||

Sbjct 144 GCTCATGCATTTATTATAATTTTTTTTATAGTAATACCTATTATAATTGGAGGATTTGGA 203

Query 185 AATTGGTTAGTCCCATTAATATTAGGTGCTCCTGATATAGCTTTCCCTCGAATAAATAAT 244

||||||||||||||||||||||||||||||||||||||||||||||||||||||||||||

Sbjct 204 AATTGGTTAGTCCCATTAATATTAGGTGCTCCTGATATAGCTTTCCCTCGAATAAATAAT 263

Query 245 ATAAGTTTTTGAATACTTCCTCCCTCTTTAACTCTTTTAATTTCTAGATCTATAGTAGAA 304

||||||||||||||||||||||||||||||||||||||||||||||||||||||||||||

Sbjct 264 ATAAGTTTTTGAATACTTCCTCCCTCTTTAACTCTTTTAATTTCTAGATCTATAGTAGAA 323

Query 305 AATGGAGCAGGAACAGGTTGAACTGTATACCCTCCTCTTTCTTCTGGAACTGCTCATGCA 364

||||||||||||||||||||||||||||||||||||||||||||||||||||||||||||

Sbjct 324 AATGGAGCAGGAACAGGTTGAACTGTATACCCTCCTCTTTCTTCTGGAACTGCTCATGCA 383

Query 365 GGAGCTTCTGTAGATTTAGCTATTTTTTCTCTTCATTTAGCAGGAATTTCTTCTATTTTA 424

||||||||||||||||||||||||||||||||||||||||||||||||||||||||||||

Sbjct 384 GGAGCTTCTGTAGATTTAGCTATTTTTTCTCTTCATTTAGCAGGAATTTCTTCTATTTTA 443

Query 425 GGAGCAGTAAATTTTATTACAACTGTAATTAATATACGATCAACTGGAATTACACTTGAT 484

||||||||||||||||||||||||||||||||||||||||||||||||||||||||||||

Sbjct 444 GGAGCAGTAAATTTTATTACAACTGTAATTAATATACGATCAACTGGAATTACACTTGAT 503

Query 485 CGTTTACCTTTATTTGTCTGATCTGTAATTATTACAGCTATTTTATTACTTCTTTCATTA 544

|| |||||||||||||||||||||||||||||||||||||||||||||||||||||||||

Sbjct 504 CGCTTACCTTTATTTGTCTGATCTGTAATTATTACAGCTATTTTATTACTTCTTTCATTA 563

Query 545 CCAGTTTTAGCAGGAGCTATTACTATATTATTAACTGATCGAAATTTTAATACATCATTT 604

||||||||||||||||||||||||||||||||||||||||||||||||||||||||||||

Sbjct 564 CCAGTTTTAGCAGGAGCTATTACTATATTATTAACTGATCGAAATTTTAATACATCATTT 623

Query 605 TTTGACCCAATTGGAGGAGGTGACCCTATTCTTTATCAACATCTATTTTGATTT 658

||||||||||||||||||||||||||||||||||||||||||||||||||||||

Sbjct 624 TTTGACCCAATTGGAGGAGGTGACCCTATTCTTTATCAACATCTATTTTGATTT 677

>Aedes malayensis voucher DNUS-WCP5 cytochrome c oxidase subunit 1 (cox1) gene, partial cds; mitochondrial

Sequence ID: KY420768.1 Length: 656

Range 1: 11 to 656

Score:1171 bits(634), Expect:0.0,

Identities:644/648(99%), Gaps:3/648(0%), Strand: Plus/Plus

Query 6 TATTTTTGGAGTATGATCGGGGA-AGTCGGAACTTCATTAAGAGTTTTAATTCGAACTGA 64

||||||||||||||||||||||| ||||||||||||||||||||||||||||||||||||

Sbjct 11 TATTTTTGGAGTATGATCGGGGATAGTCGGAACTTCATTAAGAGTTTTAATTCGAACTGA 70

Query 65 ACTTAGTCACCCTGGAATATTTATCGGAAATGATCAAATTTATAATGTAATTCGTTACTA 124

|||||||||||||||||||||||||||||||||||||||||||||||||||| ||||||

Sbjct 71 ACTTAGTCACCCTGGAATATTTATCGGAAATGATCAAATTTATAATGTAATT-GTTACT- 128

Query 125 GCTCATGCATTTATTATAAttttttttATAGTAATACCTATTATAATTGGAGGATTTGGA 184

||||||||||||||||||||||||||||||||||||||||||||||||||||||||||||

Sbjct 129 GCTCATGCATTTATTATAATTTTTTTTATAGTAATACCTATTATAATTGGAGGATTTGGA 188

Query 185 AATTGGTTAGTCCCATTAATATTAGGTGCTCCTGATATAGCTTTCCCTCGAATAAATAAT 244

||||||||||||||||||||||||||||||||||||||||||||||||||||||||||||

Sbjct 189 AATTGGTTAGTCCCATTAATATTAGGTGCTCCTGATATAGCTTTCCCTCGAATAAATAAT 248

Query 245 ATAAGTTTTTGAATACTTCCTCCCTCTTTAACTCTTTTAATTTCTAGATCTATAGTAGAA 304

||||||||||||||||||||||||||||||||||||||||||||||||||||||||||||

Sbjct 249 ATAAGTTTTTGAATACTTCCTCCCTCTTTAACTCTTTTAATTTCTAGATCTATAGTAGAA 308

Query 305 AATGGAGCAGGAACAGGTTGAACTGTATACCCTCCTCTTTCTTCTGGAACTGCTCATGCA 364

||||||||||||||||||||||||||||||||||||||||||||||||||||||||||||

Sbjct 309 AATGGAGCAGGAACAGGTTGAACTGTATACCCTCCTCTTTCTTCTGGAACTGCTCATGCA 368

Query 365 GGAGCTTCTGTAGATTTAGCTATTTTTTCTCTTCATTTAGCAGGAATTTCTTCTATTTTA 424

||||||||||||||||||||||||||||||||||||||||||||||||||||||||||||

Sbjct 369 GGAGCTTCTGTAGATTTAGCTATTTTTTCTCTTCATTTAGCAGGAATTTCTTCTATTTTA 428

Query 425 GGAGCAGTAAATTTTATTACAACTGTAATTAATATACGATCAACTGGAATTACACTTGAT 484

||||||||||||||||||||||||||||||||||||||||||||||||||||||||||||

Sbjct 429 GGAGCAGTAAATTTTATTACAACTGTAATTAATATACGATCAACTGGAATTACACTTGAT 488

Query 485 CGTTTACCTTTATTTGTCTGATCTGTAATTATTACAGCTATTTTATTACTTCTTTCATTA 544

||| ||||||||||||||||||||||||||||||||||||||||||||||||||||||||

Sbjct 489 CGTATACCTTTATTTGTCTGATCTGTAATTATTACAGCTATTTTATTACTTCTTTCATTA 548

Query 545 CCAGTTTTAGCAGGAGCTATTACTATATTATTAACTGATCGAAATTTTAATACATCATTT 604

||||||||||||||||||||||||||||||||||||||||||||||||||||||||||||

Sbjct 549 CCAGTTTTAGCAGGAGCTATTACTATATTATTAACTGATCGAAATTTTAATACATCATTT 608

Query 605 TTTGACCCAATTGGAGGAGGTGACCCTATTCTTTATCAACATCTATTT 652

||||||||||||||||||||||||||||||||||||||||||||||||

Sbjct 609 TTTGACCCAATTGGAGGAGGTGACCCTATTCTTTATCAACATCTATTT 656

>Aedes malayensis voucher DNUS-Sentosa1 cytochrome c oxidase subunit 1 (cox1) gene, partial cds; mitochondrial

Sequence ID: KY420767.1 Length: 657

Range 1: 12 to 657

Score:1171 bits(634), Expect:0.0,

Identities:644/648(99%), Gaps:3/648(0%), Strand: Plus/Plus

Query 6 TATTTTTGGAGTATGATCGGGGA-AGTCGGAACTTCATTAAGAGTTTTAATTCGAACTGA 64

||||||||||||||||||||||| ||||||||||||||||||||||||||||||||||||

Sbjct 12 TATTTTTGGAGTATGATCGGGGATAGTCGGAACTTCATTAAGAGTTTTAATTCGAACTGA 71

Query 65 ACTTAGTCACCCTGGAATATTTATCGGAAATGATCAAATTTATAATGTAATTCGTTACTA 124

|||||||||||||||||||||||||||||||||||||||||||||||||||| ||||||

Sbjct 72 ACTTAGTCACCCTGGAATATTTATCGGAAATGATCAAATTTATAATGTAATT-GTTACT- 129

Query 125 GCTCATGCATTTATTATAAttttttttATAGTAATACCTATTATAATTGGAGGATTTGGA 184

||||||||||||||||||||||||||||||||||||||||||||||||||||||||||||

Sbjct 130 GCTCATGCATTTATTATAATTTTTTTTATAGTAATACCTATTATAATTGGAGGATTTGGA 189

Query 185 AATTGGTTAGTCCCATTAATATTAGGTGCTCCTGATATAGCTTTCCCTCGAATAAATAAT 244

||||||||||||||||||||||||||||||||||||||||||||||||||||||||||||

Sbjct 190 AATTGGTTAGTCCCATTAATATTAGGTGCTCCTGATATAGCTTTCCCTCGAATAAATAAT 249

Query 245 ATAAGTTTTTGAATACTTCCTCCCTCTTTAACTCTTTTAATTTCTAGATCTATAGTAGAA 304

||||||||||||||||||||||||||||||||||||||||||||||||||||||||||||

Sbjct 250 ATAAGTTTTTGAATACTTCCTCCCTCTTTAACTCTTTTAATTTCTAGATCTATAGTAGAA 309

Query 305 AATGGAGCAGGAACAGGTTGAACTGTATACCCTCCTCTTTCTTCTGGAACTGCTCATGCA 364

||||||||||||||||||||||||||||||||||||||||||||||||||||||||||||

Sbjct 310 AATGGAGCAGGAACAGGTTGAACTGTATACCCTCCTCTTTCTTCTGGAACTGCTCATGCA 369

Query 365 GGAGCTTCTGTAGATTTAGCTATTTTTTCTCTTCATTTAGCAGGAATTTCTTCTATTTTA 424

||||||||||||||||||||||||||||||||||||||||||||||||||||||||||||

Sbjct 370 GGAGCTTCTGTAGATTTAGCTATTTTTTCTCTTCATTTAGCAGGAATTTCTTCTATTTTA 429

Query 425 GGAGCAGTAAATTTTATTACAACTGTAATTAATATACGATCAACTGGAATTACACTTGAT 484

||||||||||||||||||||||||||||||||||||||||||||||||||||||||||||

Sbjct 430 GGAGCAGTAAATTTTATTACAACTGTAATTAATATACGATCAACTGGAATTACACTTGAT 489

Query 485 CGTTTACCTTTATTTGTCTGATCTGTAATTATTACAGCTATTTTATTACTTCTTTCATTA 544

||| ||||||||||||||||||||||||||||||||||||||||||||||||||||||||

Sbjct 490 CGTATACCTTTATTTGTCTGATCTGTAATTATTACAGCTATTTTATTACTTCTTTCATTA 549

Query 545 CCAGTTTTAGCAGGAGCTATTACTATATTATTAACTGATCGAAATTTTAATACATCATTT 604

||||||||||||||||||||||||||||||||||||||||||||||||||||||||||||

Sbjct 550 CCAGTTTTAGCAGGAGCTATTACTATATTATTAACTGATCGAAATTTTAATACATCATTT 609

Query 605 TTTGACCCAATTGGAGGAGGTGACCCTATTCTTTATCAACATCTATTT 652

||||||||||||||||||||||||||||||||||||||||||||||||

Sbjct 610 TTTGACCCAATTGGAGGAGGTGACCCTATTCTTTATCAACATCTATTT 657

>Aedes malayensis voucher DNUS-WCP10 cytochrome c oxidase subunit 1 (cox1) gene, partial cds; mitochondrial

Sequence ID: KY420726.1 Length: 658

>Aedes malayensis voucher DNUS-WCP9 cytochrome c oxidase subunit 1 (cox1) gene, partial cds; mitochondrial

Sequence ID: KY420727.1 Length: 658

>Aedes malayensis voucher DNUS-WCP7 cytochrome c oxidase subunit 1 (cox1) gene, partial cds; mitochondrial

Sequence ID: KY420728.1 Length: 658

>Aedes malayensis voucher DNUS-WCP6 cytochrome c oxidase subunit 1 (cox1) gene, partial cds; mitochondrial

Sequence ID: KY420729.1 Length: 658

>Aedes malayensis voucher DNUS-WCP3 cytochrome c oxidase subunit 1 (cox1) gene, partial cds; mitochondrial

Sequence ID: KY420730.1 Length: 658

>Aedes malayensis voucher DNUS-WCP2 cytochrome c oxidase subunit 1 (cox1) gene, partial cds; mitochondrial

Sequence ID: KY420731.1 Length: 658

>Aedes malayensis voucher DNUS-WCP1 cytochrome c oxidase subunit 1 (cox1) gene, partial cds; mitochondrial

Sequence ID: KY420732.1 Length: 658

>Aedes malayensis voucher DNUS-Sentosa2 cytochrome c oxidase subunit 1 (cox1) gene, partial cds; mitochondrial

Sequence ID: KY420733.1 Length: 658

>Aedes malayensis voucher DNUS-Sentosa7 cytochrome c oxidase subunit 1 (cox1) gene, partial cds; mitochondrial

Sequence ID: KY420734.1 Length: 658

>Aedes malayensis voucher DNUS-Sentosa6 cytochrome c oxidase subunit 1 (cox1) gene, partial cds; mitochondrial

Sequence ID: KY420735.1 Length: 658

>Aedes malayensis voucher DNUS-Sembawang10 cytochrome c oxidase subunit 1 (cox1) gene, partial cds; mitochondrial

Sequence ID: KY420736.1 Length: 658

>Aedes malayensis voucher DNUS-Sembawang9 cytochrome c oxidase subunit 1 (cox1) gene, partial cds; mitochondrial

Sequence ID: KY420737.1 Length: 658

>Aedes malayensis voucher DNUS-Sembawang8 cytochrome c oxidase subunit 1 (cox1) gene, partial cds; mitochondrial

Sequence ID: KY420738.1 Length: 658

>Aedes malayensis voucher DNUS-Sembawang7 cytochrome c oxidase subunit 1 (cox1) gene, partial cds; mitochondrial

Sequence ID: KY420739.1 Length: 658

>Aedes malayensis voucher DNUS-Sembawang6 cytochrome c oxidase subunit 1 (cox1) gene, partial cds; mitochondrial

Sequence ID: KY420740.1 Length: 658

>Aedes malayensis voucher DNUS-Sembawang5 cytochrome c oxidase subunit 1 (cox1) gene, partial cds; mitochondrial

Sequence ID: KY420741.1 Length: 658

>Aedes malayensis voucher DNUS-Sembawang4 cytochrome c oxidase subunit 1 (cox1) gene, partial cds; mitochondrial

Sequence ID: KY420742.1 Length: 658

>Aedes malayensis voucher DNUS-Sembawang3 cytochrome c oxidase subunit 1 (cox1) gene, partial cds; mitochondrial

Sequence ID: KY420743.1 Length: 658

>Aedes malayensis voucher DNUS-Sembawang2 cytochrome c oxidase subunit 1 (cox1) gene, partial cds; mitochondrial

Sequence ID: KY420744.1 Length: 658

>Aedes malayensis voucher DNUS-Sembawang1 cytochrome c oxidase subunit 1 (cox1) gene, partial cds; mitochondrial

Sequence ID: KY420745.1 Length: 658

>Aedes malayensis voucher DNUS-MtFaber7 cytochrome c oxidase subunit 1 (cox1) gene, partial cds; mitochondrial

Sequence ID: KY420746.1 Length: 658

>Aedes malayensis voucher DNUS-MtFaber3 cytochrome c oxidase subunit 1 (cox1) gene, partial cds; mitochondrial

Sequence ID: KY420747.1 Length: 658

>Aedes malayensis voucher DNUS-MtFaber2 cytochrome c oxidase subunit 1 (cox1) gene, partial cds; mitochondrial

Sequence ID: KY420748.1 Length: 658

>Aedes malayensis voucher DNUS-ECP3-10 cytochrome c oxidase subunit 1 (cox1) gene, partial cds; mitochondrial

Sequence ID: KY420749.1 Length: 658

>Aedes malayensis voucher DNUS-ECP2-6 cytochrome c oxidase subunit 1 (cox1) gene, partial cds; mitochondrial

Sequence ID: KY420750.1 Length: 658

>Aedes malayensis voucher DNUS-ECP2-5 cytochrome c oxidase subunit 1 (cox1) gene, partial cds; mitochondrial

Sequence ID: KY420751.1 Length: 658

>Aedes malayensis voucher DNUS-ECP1-10 cytochrome c oxidase subunit 1 (cox1) gene, partial cds; mitochondrial

Sequence ID: KY420752.1 Length: 658

>Aedes malayensis voucher DNUS-ECP1-9 cytochrome c oxidase subunit 1 (cox1) gene, partial cds; mitochondrial

Sequence ID: KY420753.1 Length: 658

>Aedes malayensis voucher DNUS-ECP1-8 cytochrome c oxidase subunit 1 (cox1) gene, partial cds; mitochondrial

Sequence ID: KY420754.1 Length: 658

>Aedes malayensis voucher DNUS-ECP1-7 cytochrome c oxidase subunit 1 (cox1) gene, partial cds; mitochondrial

Sequence ID: KY420755.1 Length: 658

>Aedes malayensis voucher DNUS-ECP1-6 cytochrome c oxidase subunit 1 (cox1) gene, partial cds; mitochondrial

Sequence ID: KY420756.1 Length: 658

>Aedes malayensis voucher DNUS-ECP1-5 cytochrome c oxidase subunit 1 (cox1) gene, partial cds; mitochondrial

Sequence ID: KY420757.1 Length: 658

>Aedes malayensis voucher DNUS-ECP1-1 cytochrome c oxidase subunit 1 (cox1) gene, partial cds; mitochondrial

Sequence ID: KY420758.1 Length: 658

>Aedes malayensis voucher DNUS-Clementi8 cytochrome c oxidase subunit 1 (cox1) gene, partial cds; mitochondrial

Sequence ID: KY420759.1 Length: 658

>Aedes malayensis voucher DNUS-BukitBatok9 cytochrome c oxidase subunit 1 (cox1) gene, partial cds; mitochondrial

Sequence ID: KY420760.1 Length: 658

>Aedes malayensis voucher DNUS-BukitBatok8 cytochrome c oxidase subunit 1 (cox1) gene, partial cds; mitochondrial

Sequence ID: KY420761.1 Length: 658

>Aedes malayensis voucher DNUS-BukitBatok7 cytochrome c oxidase subunit 1 (cox1) gene, partial cds; mitochondrial

Sequence ID: KY420762.1 Length: 658

>Aedes malayensis voucher DNUS-BukitBatok6 cytochrome c oxidase subunit 1 (cox1) gene, partial cds; mitochondrial

Sequence ID: KY420763.1 Length: 658

>Aedes malayensis voucher DNUS-BukitBatok5 cytochrome c oxidase subunit 1 (cox1) gene, partial cds; mitochondrial

Sequence ID: KY420764.1 Length: 658

>Aedes malayensis voucher DNUS-BukitBatok2 cytochrome c oxidase subunit 1 (cox1) gene, partial cds; mitochondrial

Sequence ID: KY420765.1 Length: 658

>Aedes malayensis voucher DNUS-BukitBatok1 cytochrome c oxidase subunit 1 (cox1) gene, partial cds; mitochondrial

Sequence ID: KY420766.1 Length: 658

>Aedes malayensis voucher DNUS-Clementi7 cytochrome c oxidase subunit 1 (cox1) gene, partial cds; mitochondrial

Sequence ID: KY420814.1 Length: 658

Range 1: 13 to 658

Score:1171 bits(634), Expect:0.0,

Identities:644/648(99%), Gaps:3/648(0%), Strand: Plus/Plus

Query 6 TATTTTTGGAGTATGATCGGGGA-AGTCGGAACTTCATTAAGAGTTTTAATTCGAACTGA 64

||||||||||||||||||||||| ||||||||||||||||||||||||||||||||||||

Sbjct 13 TATTTTTGGAGTATGATCGGGGATAGTCGGAACTTCATTAAGAGTTTTAATTCGAACTGA 72

Query 65 ACTTAGTCACCCTGGAATATTTATCGGAAATGATCAAATTTATAATGTAATTCGTTACTA 124

|||||||||||||||||||||||||||||||||||||||||||||||||||| ||||||

Sbjct 73 ACTTAGTCACCCTGGAATATTTATCGGAAATGATCAAATTTATAATGTAATT-GTTACT- 130

Query 125 GCTCATGCATTTATTATAAttttttttATAGTAATACCTATTATAATTGGAGGATTTGGA 184

||||||||||||||||||||||||||||||||||||||||||||||||||||||||||||

Sbjct 131 GCTCATGCATTTATTATAATTTTTTTTATAGTAATACCTATTATAATTGGAGGATTTGGA 190

Query 185 AATTGGTTAGTCCCATTAATATTAGGTGCTCCTGATATAGCTTTCCCTCGAATAAATAAT 244

||||||||||||||||||||||||||||||||||||||||||||||||||||||||||||

Sbjct 191 AATTGGTTAGTCCCATTAATATTAGGTGCTCCTGATATAGCTTTCCCTCGAATAAATAAT 250

Query 245 ATAAGTTTTTGAATACTTCCTCCCTCTTTAACTCTTTTAATTTCTAGATCTATAGTAGAA 304

||||||||||||||||||||||||||||||||||||||||||||||||||||||||||||

Sbjct 251 ATAAGTTTTTGAATACTTCCTCCCTCTTTAACTCTTTTAATTTCTAGATCTATAGTAGAA 310

Query 305 AATGGAGCAGGAACAGGTTGAACTGTATACCCTCCTCTTTCTTCTGGAACTGCTCATGCA 364

||||||||||||||||||||||||||||||||||||||||||||||||||||||||||||

Sbjct 311 AATGGAGCAGGAACAGGTTGAACTGTATACCCTCCTCTTTCTTCTGGAACTGCTCATGCA 370

Query 365 GGAGCTTCTGTAGATTTAGCTATTTTTTCTCTTCATTTAGCAGGAATTTCTTCTATTTTA 424

||||||||||||||||||||||||||||||||||||||||||||||||||||||||||||

Sbjct 371 GGAGCTTCTGTAGATTTAGCTATTTTTTCTCTTCATTTAGCAGGAATTTCTTCTATTTTA 430

Query 425 GGAGCAGTAAATTTTATTACAACTGTAATTAATATACGATCAACTGGAATTACACTTGAT 484

||||||||||||||||||||||||||||||||||||||||||||||||||||||||||||

Sbjct 431 GGAGCAGTAAATTTTATTACAACTGTAATTAATATACGATCAACTGGAATTACACTTGAT 490

Query 485 CGTTTACCTTTATTTGTCTGATCTGTAATTATTACAGCTATTTTATTACTTCTTTCATTA 544

||| ||||||||||||||||||||||||||||||||||||||||||||||||||||||||

Sbjct 491 CGTATACCTTTATTTGTCTGATCTGTAATTATTACAGCTATTTTATTACTTCTTTCATTA 550

Query 545 CCAGTTTTAGCAGGAGCTATTACTATATTATTAACTGATCGAAATTTTAATACATCATTT 604

||||||||||||||||||||||||||||||||||||||||||||||||||||||||||||

Sbjct 551 CCAGTTTTAGCAGGAGCTATTACTATATTATTAACTGATCGAAATTTTAATACATCATTT 610

Query 605 TTTGACCCAATTGGAGGAGGTGACCCTATTCTTTATCAACATCTATTT 652

||||||||||||||||||||||||||||||||||||||||||||||||

Sbjct 611 TTTGACCCAATTGGAGGAGGTGACCCTATTCTTTATCAACATCTATTT 658

>Aedes malayensis voucher DNUS-Clementi9 cytochrome c oxidase subunit 1 (cox1) gene, partial cds; mitochondrial

Sequence ID: KY420725.1 Length: 658

Range 1: 13 to 658

Score:1171 bits(634), Expect:0.0,

Identities:644/648(99%), Gaps:3/648(0%), Strand: Plus/Plus

Query 6 TATTTTTGGAGTATGATCGGGGA-AGTCGGAACTTCATTAAGAGTTTTAATTCGAACTGA 64

||||||||||||||||||||||| ||||||||||||||||||||||||||||||||||||

Sbjct 13 TATTTTTGGAGTATGATCGGGGATAGTCGGAACTTCATTAAGAGTTTTAATTCGAACTGA 72

Query 65 ACTTAGTCACCCTGGAATATTTATCGGAAATGATCAAATTTATAATGTAATTCGTTACTA 124

|||||||||||||||||||||||||||||||||||||||||||||||||||| ||||||

Sbjct 73 ACTTAGTCACCCTGGAATATTTATCGGAAATGATCAAATTTATAATGTAATT-GTTACT- 130

Query 125 GCTCATGCATTTATTATAAttttttttATAGTAATACCTATTATAATTGGAGGATTTGGA 184

||||||||||||||||||||||||||||||||||||||||||||||||||||||||||||

Sbjct 131 GCTCATGCATTTATTATAATTTTTTTTATAGTAATACCTATTATAATTGGAGGATTTGGA 190

Query 185 AATTGGTTAGTCCCATTAATATTAGGTGCTCCTGATATAGCTTTCCCTCGAATAAATAAT 244

||||||||||||||||||||||||||||||||||||||||||||||||||||||||||||

Sbjct 191 AATTGGTTAGTCCCATTAATATTAGGTGCTCCTGATATAGCTTTCCCTCGAATAAATAAT 250

Query 245 ATAAGTTTTTGAATACTTCCTCCCTCTTTAACTCTTTTAATTTCTAGATCTATAGTAGAA 304

||||||||||||||||||||||||||||||||||||||||||||||||||||||||||||

Sbjct 251 ATAAGTTTTTGAATACTTCCTCCCTCTTTAACTCTTTTAATTTCTAGATCTATAGTAGAA 310

Query 305 AATGGAGCAGGAACAGGTTGAACTGTATACCCTCCTCTTTCTTCTGGAACTGCTCATGCA 364

||||||||||||||||||||||||||||||||||||||||||||||||||||||||||||

Sbjct 311 AATGGAGCAGGAACAGGTTGAACTGTATACCCTCCTCTTTCTTCTGGAACTGCTCATGCA 370

Query 365 GGAGCTTCTGTAGATTTAGCTATTTTTTCTCTTCATTTAGCAGGAATTTCTTCTATTTTA 424

||||||||||||||||||||||||||||||||||||||||||||||||||||||||||||

Sbjct 371 GGAGCTTCTGTAGATTTAGCTATTTTTTCTCTTCATTTAGCAGGAATTTCTTCTATTTTA 430

Query 425 GGAGCAGTAAATTTTATTACAACTGTAATTAATATACGATCAACTGGAATTACACTTGAT 484

||||||||||||||||||||||||||||||||||||||||||||||||||||||||||||

Sbjct 431 GGAGCAGTAAATTTTATTACAACTGTAATTAATATACGATCAACTGGAATTACACTTGAT 490

Query 485 CGTTTACCTTTATTTGTCTGATCTGTAATTATTACAGCTATTTTATTACTTCTTTCATTA 544

||| ||||||||||||||||||||||||||||||||||||||||||||||||||||||||

Sbjct 491 CGTATACCTTTATTTGTCTGATCTGTAATTATTACAGCTATTTTATTACTTCTTTCATTA 550

Query 545 CCAGTTTTAGCAGGAGCTATTACTATATTATTAACTGATCGAAATTTTAATACATCATTT 604

||||||||||||||||||||||||||||||||||||||||||||||||||||||||||||

Sbjct 551 CCAGTTTTAGCAGGAGCTATTACTATATTATTAACTGATCGAAATTTTAATACATCATTT 610

Query 605 TTTGACCCAATTGGAGGAGGTGACCCTATTCTTTATCAACATCTATTT 652

||||||||||||||||||||||||||||||||||||||||||||||||

Sbjct 611 TTTGACCCAATTGGAGGAGGTGACCCTATTCTTTATCAACATCTATTT 658

>Aedes malayensis voucher Aedes_malayensis_S39_109A cytochrome c oxidase subunit I (COX1) gene, partial cds; mitochondrial

Sequence ID: MW321936.1 Length: 677

Range 1: 26 to 677

Score:1171 bits(634), Expect:0.0,

Identities:648/654(99%), Gaps:3/654(0%), Strand: Plus/Plus

Query 6 TATTTTTGGAGTATGATCGGGGA-AGTCGGAACTTCATTAAGAGTTTTAATTCGAACTGA 64

||||||||||||||||||||| | ||||||||||||||||||||||||||||||||||||

Sbjct 26 TATTTTTGGAGTATGATCGGGAATAGTCGGAACTTCATTAAGAGTTTTAATTCGAACTGA 85

Query 65 ACTTAGTCACCCTGGAATATTTATCGGAAATGATCAAATTTATAATGTAATTCGTTACTA 124

|||||||||||||||||||||||||||||||||||||||||||||||||||| ||||||

Sbjct 86 ACTTAGTCACCCTGGAATATTTATCGGAAATGATCAAATTTATAATGTAATT-GTTACT- 143

Query 125 GCTCATGCATTTATTATAAttttttttATAGTAATACCTATTATAATTGGAGGATTTGGA 184

||||||||||||||||||||||| ||||||||||||||||||||||||||||||||||||

Sbjct 144 GCTCATGCATTTATTATAATTTTCTTTATAGTAATACCTATTATAATTGGAGGATTTGGA 203

Query 185 AATTGGTTAGTCCCATTAATATTAGGTGCTCCTGATATAGCTTTCCCTCGAATAAATAAT 244

||||||||||||||||||||||||||||||||||||||||||||||||||||||||||||

Sbjct 204 AATTGGTTAGTCCCATTAATATTAGGTGCTCCTGATATAGCTTTCCCTCGAATAAATAAT 263

Query 245 ATAAGTTTTTGAATACTTCCTCCCTCTTTAACTCTTTTAATTTCTAGATCTATAGTAGAA 304

||||||||||||||||||||||||||||||||||||||||||||||||||||||||||||

Sbjct 264 ATAAGTTTTTGAATACTTCCTCCCTCTTTAACTCTTTTAATTTCTAGATCTATAGTAGAA 323

Query 305 AATGGAGCAGGAACAGGTTGAACTGTATACCCTCCTCTTTCTTCTGGAACTGCTCATGCA 364

||||||||||||||||||||||||||||||||||||||||||||||||||||||||||||

Sbjct 324 AATGGAGCAGGAACAGGTTGAACTGTATACCCTCCTCTTTCTTCTGGAACTGCTCATGCA 383

Query 365 GGAGCTTCTGTAGATTTAGCTATTTTTTCTCTTCATTTAGCAGGAATTTCTTCTATTTTA 424

||||||||||||||||||||||||||||||||||||||||||||||||||||||||||||

Sbjct 384 GGAGCTTCTGTAGATTTAGCTATTTTTTCTCTTCATTTAGCAGGAATTTCTTCTATTTTA 443

Query 425 GGAGCAGTAAATTTTATTACAACTGTAATTAATATACGATCAACTGGAATTACACTTGAT 484

||||||||||||||||||||||||||||||||||||||||||||||||||||||||||||

Sbjct 444 GGAGCAGTAAATTTTATTACAACTGTAATTAATATACGATCAACTGGAATTACACTTGAT 503

Query 485 CGTTTACCTTTATTTGTCTGATCTGTAATTATTACAGCTATTTTATTACTTCTTTCATTA 544

|| |||||||||||||||||||||||||||||||||||||||||||||||||||||||||

Sbjct 504 CGCTTACCTTTATTTGTCTGATCTGTAATTATTACAGCTATTTTATTACTTCTTTCATTA 563

Query 545 CCAGTTTTAGCAGGAGCTATTACTATATTATTAACTGATCGAAATTTTAATACATCATTT 604

||||||||||||||||||||||||||||||||||||||||||||||||||||||||||||

Sbjct 564 CCAGTTTTAGCAGGAGCTATTACTATATTATTAACTGATCGAAATTTTAATACATCATTT 623

Query 605 TTTGACCCAATTGGAGGAGGTGACCCTATTCTTTATCAACATCTATTTTGATTT 658

||||||||||||||||||||||||||||||||||||||||||||||||||||||

Sbjct 624 TTTGACCCAATTGGAGGAGGTGACCCTATTCTTTATCAACATCTATTTTGATTT 677

>Aedes malayensis voucher DNUS-Sentosa3 cytochrome c oxidase subunit 1 (cox1) gene, partial cds; mitochondrial

Sequence ID: KY420776.1 Length: 658

>Aedes malayensis voucher DNUS-Sentosa10 cytochrome c oxidase subunit 1 (cox1) gene, partial cds; mitochondrial

Sequence ID: KY420777.1 Length: 658

>Aedes malayensis voucher DNUS-Sentosa9 cytochrome c oxidase subunit 1 (cox1) gene, partial cds; mitochondrial

Sequence ID: KY420778.1 Length: 658

>Aedes malayensis voucher DNUS-Sentosa5 cytochrome c oxidase subunit 1 (cox1) gene, partial cds; mitochondrial

Sequence ID: KY420779.1 Length: 658

>Aedes malayensis voucher DNUS-PulauS4 cytochrome c oxidase subunit 1 (cox1) gene, partial cds; mitochondrial

Sequence ID: KY420780.1 Length: 658

>Aedes malayensis voucher DNUS-PulauS3 cytochrome c oxidase subunit 1 (cox1) gene, partial cds; mitochondrial

Sequence ID: KY420781.1 Length: 658

>Aedes malayensis voucher DNUS-PulauS2 cytochrome c oxidase subunit 1 (cox1) gene, partial cds; mitochondrial

Sequence ID: KY420782.1 Length: 658

>Aedes malayensis voucher DNUS-PulauS1 cytochrome c oxidase subunit 1 (cox1) gene, partial cds; mitochondrial

Sequence ID: KY420783.1 Length: 658

>Aedes malayensis voucher DNUS-MtFaber10 cytochrome c oxidase subunit 1 (cox1) gene, partial cds; mitochondrial

Sequence ID: KY420784.1 Length: 658

>Aedes malayensis voucher DNUS-MtFaber9 cytochrome c oxidase subunit 1 (cox1) gene, partial cds; mitochondrial

Sequence ID: KY420785.1 Length: 658

>Aedes malayensis voucher DNUS-MtFaber8 cytochrome c oxidase subunit 1 (cox1) gene, partial cds; mitochondrial

Sequence ID: KY420786.1 Length: 658

>Aedes malayensis voucher DNUS-MtFaber6 cytochrome c oxidase subunit 1 (cox1) gene, partial cds; mitochondrial

Sequence ID: KY420787.1 Length: 658

>Aedes malayensis voucher DNUS-MtFaber5 cytochrome c oxidase subunit 1 (cox1) gene, partial cds; mitochondrial

Sequence ID: KY420788.1 Length: 658

>Aedes malayensis voucher DNUS-MtFaber4 cytochrome c oxidase subunit 1 (cox1) gene, partial cds; mitochondrial

Sequence ID: KY420789.1 Length: 658

>Aedes malayensis voucher DNUS-MtFaber1 cytochrome c oxidase subunit 1 (cox1) gene, partial cds; mitochondrial

Sequence ID: KY420790.1 Length: 658

>Aedes malayensis voucher DNUS-ECP3-9 cytochrome c oxidase subunit 1 (cox1) gene, partial cds; mitochondrial

Sequence ID: KY420791.1 Length: 658

>Aedes malayensis voucher DNUS-ECP3-8 cytochrome c oxidase subunit 1 (cox1) gene, partial cds; mitochondrial

Sequence ID: KY420792.1 Length: 658

>Aedes malayensis voucher DNUS-ECP3-7 cytochrome c oxidase subunit 1 (cox1) gene, partial cds; mitochondrial

Sequence ID: KY420793.1 Length: 658

>Aedes malayensis voucher DNUS-ECP3-6 cytochrome c oxidase subunit 1 (cox1) gene, partial cds; mitochondrial

Sequence ID: KY420794.1 Length: 658

>Aedes malayensis voucher DNUS-ECP3-5 cytochrome c oxidase subunit 1 (cox1) gene, partial cds; mitochondrial

Sequence ID: KY420795.1 Length: 658

>Aedes malayensis voucher DNUS-ECP3-4 cytochrome c oxidase subunit 1 (cox1) gene, partial cds; mitochondrial

Sequence ID: KY420796.1 Length: 658

>Aedes malayensis voucher DNUS-ECP3-3 cytochrome c oxidase subunit 1 (cox1) gene, partial cds; mitochondrial

Sequence ID: KY420797.1 Length: 658

>Aedes malayensis voucher DNUS-ECP3-1 cytochrome c oxidase subunit 1 (cox1) gene, partial cds; mitochondrial

Sequence ID: KY420798.1 Length: 658

>Aedes malayensis voucher DNUS-ECP2-9 cytochrome c oxidase subunit 1 (cox1) gene, partial cds; mitochondrial

Sequence ID: KY420799.1 Length: 658

>Aedes malayensis voucher DNUS-ECP2-8 cytochrome c oxidase subunit 1 (cox1) gene, partial cds; mitochondrial

Sequence ID: KY420800.1 Length: 658

>Aedes malayensis voucher DNUS-ECP2-7 cytochrome c oxidase subunit 1 (cox1) gene, partial cds; mitochondrial

Sequence ID: KY420801.1 Length: 658

>Aedes malayensis voucher DNUS-ECP2-4 cytochrome c oxidase subunit 1 (cox1) gene, partial cds; mitochondrial

Sequence ID: KY420802.1 Length: 658

>Aedes malayensis voucher DNUS-ECP2-3 cytochrome c oxidase subunit 1 (cox1) gene, partial cds; mitochondrial

Sequence ID: KY420803.1 Length: 658

>Aedes malayensis voucher DNUS-ECP2-1 cytochrome c oxidase subunit 1 (cox1) gene, partial cds; mitochondrial

Sequence ID: KY420804.1 Length: 658

>Aedes malayensis voucher DNUS-ECP1-4 cytochrome c oxidase subunit 1 (cox1) gene, partial cds; mitochondrial

Sequence ID: KY420805.1 Length: 658

>Aedes malayensis voucher DNUS-Clementi5 cytochrome c oxidase subunit 1 (cox1) gene, partial cds; mitochondrial

Sequence ID: KY420806.1 Length: 658

>Aedes malayensis voucher DNUS-Clementi4 cytochrome c oxidase subunit 1 (cox1) gene, partial cds; mitochondrial

Sequence ID: KY420807.1 Length: 658

>Aedes malayensis voucher DNUS-Clementi2 cytochrome c oxidase subunit 1 (cox1) gene, partial cds; mitochondrial

Sequence ID: KY420808.1 Length: 658

>Aedes malayensis voucher DNUS-BukitBatok10 cytochrome c oxidase subunit 1 (cox1) gene, partial cds; mitochondrial

Sequence ID: KY420809.1 Length: 658

>Aedes malayensis voucher DNUS-BukitBatok4 cytochrome c oxidase subunit 1 (cox1) gene, partial cds; mitochondrial

Sequence ID: KY420810.1 Length: 658

>Aedes malayensis voucher DNUS-BukitBatok3 cytochrome c oxidase subunit 1 (cox1) gene, partial cds; mitochondrial

Sequence ID: KY420811.1 Length: 658

>Aedes malayensis voucher DNUS-Clementi10 cytochrome c oxidase subunit 1 (cox1) gene, partial cds; mitochondrial

Sequence ID: KY420812.1 Length: 658

Range 1: 13 to 658

Score:1166 bits(631), Expect:0.0,

Identities:643/648(99%), Gaps:3/648(0%), Strand: Plus/Plus

Query 6 TATTTTTGGAGTATGATCGGGGA-AGTCGGAACTTCATTAAGAGTTTTAATTCGAACTGA 64

||||||||||||||||||||| | ||||||||||||||||||||||||||||||||||||

Sbjct 13 TATTTTTGGAGTATGATCGGGAATAGTCGGAACTTCATTAAGAGTTTTAATTCGAACTGA 72

Query 65 ACTTAGTCACCCTGGAATATTTATCGGAAATGATCAAATTTATAATGTAATTCGTTACTA 124

|||||||||||||||||||||||||||||||||||||||||||||||||||| ||||||

Sbjct 73 ACTTAGTCACCCTGGAATATTTATCGGAAATGATCAAATTTATAATGTAATT-GTTACT- 130

Query 125 GCTCATGCATTTATTATAAttttttttATAGTAATACCTATTATAATTGGAGGATTTGGA 184

||||||||||||||||||||||||||||||||||||||||||||||||||||||||||||

Sbjct 131 GCTCATGCATTTATTATAATTTTTTTTATAGTAATACCTATTATAATTGGAGGATTTGGA 190

Query 185 AATTGGTTAGTCCCATTAATATTAGGTGCTCCTGATATAGCTTTCCCTCGAATAAATAAT 244

||||||||||||||||||||||||||||||||||||||||||||||||||||||||||||

Sbjct 191 AATTGGTTAGTCCCATTAATATTAGGTGCTCCTGATATAGCTTTCCCTCGAATAAATAAT 250

Query 245 ATAAGTTTTTGAATACTTCCTCCCTCTTTAACTCTTTTAATTTCTAGATCTATAGTAGAA 304

||||||||||||||||||||||||||||||||||||||||||||||||||||||||||||

Sbjct 251 ATAAGTTTTTGAATACTTCCTCCCTCTTTAACTCTTTTAATTTCTAGATCTATAGTAGAA 310

Query 305 AATGGAGCAGGAACAGGTTGAACTGTATACCCTCCTCTTTCTTCTGGAACTGCTCATGCA 364

||||||||||||||||||||||||||||||||||||||||||||||||||||||||||||

Sbjct 311 AATGGAGCAGGAACAGGTTGAACTGTATACCCTCCTCTTTCTTCTGGAACTGCTCATGCA 370

Query 365 GGAGCTTCTGTAGATTTAGCTATTTTTTCTCTTCATTTAGCAGGAATTTCTTCTATTTTA 424

||||||||||||||||||||||||||||||||||||||||||||||||||||||||||||

Sbjct 371 GGAGCTTCTGTAGATTTAGCTATTTTTTCTCTTCATTTAGCAGGAATTTCTTCTATTTTA 430

Query 425 GGAGCAGTAAATTTTATTACAACTGTAATTAATATACGATCAACTGGAATTACACTTGAT 484

||||||||||||||||||||||||||||||||||||||||||||||||||||||||||||

Sbjct 431 GGAGCAGTAAATTTTATTACAACTGTAATTAATATACGATCAACTGGAATTACACTTGAT 490

Query 485 CGTTTACCTTTATTTGTCTGATCTGTAATTATTACAGCTATTTTATTACTTCTTTCATTA 544

|| |||||||||||||||||||||||||||||||||||||||||||||||||||||||||

Sbjct 491 CGCTTACCTTTATTTGTCTGATCTGTAATTATTACAGCTATTTTATTACTTCTTTCATTA 550

Query 545 CCAGTTTTAGCAGGAGCTATTACTATATTATTAACTGATCGAAATTTTAATACATCATTT 604

||||||||||||||||||||||||||||||||||||||||||||||||||||||||||||

Sbjct 551 CCAGTTTTAGCAGGAGCTATTACTATATTATTAACTGATCGAAATTTTAATACATCATTT 610

Query 605 TTTGACCCAATTGGAGGAGGTGACCCTATTCTTTATCAACATCTATTT 652

||||||||||||||||||||||||||||||||||||||||||||||||

Sbjct 611 TTTGACCCAATTGGAGGAGGTGACCCTATTCTTTATCAACATCTATTT 658

>Aedes malayensis voucher DNUS-ECP1-3 cytochrome c oxidase subunit 1 (cox1) gene, partial cds; mitochondrial

Sequence ID: KY420770.1 Length: 658

>Aedes malayensis voucher DNUS-ECP1-2 cytochrome c oxidase subunit 1 (cox1) gene, partial cds; mitochondrial

Sequence ID: KY420771.1 Length: 658

Range 1: 13 to 658

Score:1166 bits(631), Expect:0.0,

Identities:643/648(99%), Gaps:3/648(0%), Strand: Plus/Plus

Query 6 TATTTTTGGAGTATGATCGGGGA-AGTCGGAACTTCATTAAGAGTTTTAATTCGAACTGA 64

||||||||||||||||||||| | ||||||||||||||||||||||||||||||||||||

Sbjct 13 TATTTTTGGAGTATGATCGGGAATAGTCGGAACTTCATTAAGAGTTTTAATTCGAACTGA 72

Query 65 ACTTAGTCACCCTGGAATATTTATCGGAAATGATCAAATTTATAATGTAATTCGTTACTA 124

|||||||||||||||||||||||||||||||||||||||||||||||||||| ||||||

Sbjct 73 ACTTAGTCACCCTGGAATATTTATCGGAAATGATCAAATTTATAATGTAATT-GTTACT- 130

Query 125 GCTCATGCATTTATTATAAttttttttATAGTAATACCTATTATAATTGGAGGATTTGGA 184

||||||||||||||||||||||||||||||||||||||||||||||||||||||||||||

Sbjct 131 GCTCATGCATTTATTATAATTTTTTTTATAGTAATACCTATTATAATTGGAGGATTTGGA 190

Query 185 AATTGGTTAGTCCCATTAATATTAGGTGCTCCTGATATAGCTTTCCCTCGAATAAATAAT 244

||||||||||||||||||||||||||||||||||||||||||||||||||||||||||||

Sbjct 191 AATTGGTTAGTCCCATTAATATTAGGTGCTCCTGATATAGCTTTCCCTCGAATAAATAAT 250

Query 245 ATAAGTTTTTGAATACTTCCTCCCTCTTTAACTCTTTTAATTTCTAGATCTATAGTAGAA 304

||||||||||||||||||||||||||||||||||||||||||||||||||||||||||||

Sbjct 251 ATAAGTTTTTGAATACTTCCTCCCTCTTTAACTCTTTTAATTTCTAGATCTATAGTAGAA 310

Query 305 AATGGAGCAGGAACAGGTTGAACTGTATACCCTCCTCTTTCTTCTGGAACTGCTCATGCA 364

||||||||||||||||||||||||||||||||||||||||||||||||||||||||||||

Sbjct 311 AATGGAGCAGGAACAGGTTGAACTGTATACCCTCCTCTTTCTTCTGGAACTGCTCATGCA 370

Query 365 GGAGCTTCTGTAGATTTAGCTATTTTTTCTCTTCATTTAGCAGGAATTTCTTCTATTTTA 424

||||||||||||||||||||||||||||||||||||||||||||||||||||||||||||

Sbjct 371 GGAGCTTCTGTAGATTTAGCTATTTTTTCTCTTCATTTAGCAGGAATTTCTTCTATTTTA 430

Query 425 GGAGCAGTAAATTTTATTACAACTGTAATTAATATACGATCAACTGGAATTACACTTGAT 484

||||||||||||||||||||||||||||||||||||||||||||||||||||||||||||

Sbjct 431 GGAGCAGTAAATTTTATTACAACTGTAATTAATATACGATCAACTGGAATTACACTTGAT 490

Query 485 CGTTTACCTTTATTTGTCTGATCTGTAATTATTACAGCTATTTTATTACTTCTTTCATTA 544

||| ||||||||||||||||||||||||||||||||||||||||||||||||||||||||

Sbjct 491 CGTATACCTTTATTTGTCTGATCTGTAATTATTACAGCTATTTTATTACTTCTTTCATTA 550

Query 545 CCAGTTTTAGCAGGAGCTATTACTATATTATTAACTGATCGAAATTTTAATACATCATTT 604

||||||||||||||||||||||||||||||||||||||||||||||||||||||||||||

Sbjct 551 CCAGTTTTAGCAGGAGCTATTACTATATTATTAACTGATCGAAATTTTAATACATCATTT 610

Query 605 TTTGACCCAATTGGAGGAGGTGACCCTATTCTTTATCAACATCTATTT 652

||||||||||||||||||||||||||||||||||||||||||||||||

Sbjct 611 TTTGACCCAATTGGAGGAGGTGACCCTATTCTTTATCAACATCTATTT 658

>Aedes albopictus isolate AB1S1_04B cytochrome oxidase subunit 1 (COI) gene, partial cds; mitochondrial

Sequence ID: KM613084.1 Length: 639

>Aedes albopictus isolate AB1S9_04B cytochrome oxidase subunit 1 (COI) gene, partial cds; mitochondrial

Sequence ID: KM613086.1 Length: 639

>Aedes albopictus isolate AB2_04B cytochrome oxidase subunit 1 (COI) gene, partial cds; mitochondrial

Sequence ID: KM613087.1 Length: 639

>Aedes albopictus isolate AB2S4_inB cytochrome oxidase subunit 1 (COI) gene, partial cds; mitochondrial

Sequence ID: KM613089.1 Length: 639

>Aedes albopictus isolate AB3_inB cytochrome oxidase subunit 1 (COI) gene, partial cds; mitochondrial

Sequence ID: KM613090.1 Length: 639

>Aedes albopictus isolate AB4_inB cytochrome oxidase subunit 1 (COI) gene, partial cds; mitochondrial

Sequence ID: KM613093.1 Length: 639

>Aedes albopictus isolate AB5_inB cytochrome oxidase subunit 1 (COI) gene, partial cds; mitochondrial

Sequence ID: KM613095.1 Length: 639

>Aedes albopictus isolate AB1_3S13_08P cytochrome oxidase subunit 1 (COI) gene, partial cds; mitochondrial

Sequence ID: KM613096.1 Length: 639

Range 1: 1 to 639

Score:1164 bits(630), Expect:0.0,

Identities:638/641(99%), Gaps:3/641(0%), Strand: Plus/Plus

Query 13 GGAGTATGATCGGGGA-AGTCGGAACTTCATTAAGAGTTTTAATTCGAACTGAACTTAGT 71

|||||||||||||||| |||||||||||||||||||||||||||||||||||||||||||

Sbjct 1 GGAGTATGATCGGGGATAGTCGGAACTTCATTAAGAGTTTTAATTCGAACTGAACTTAGT 60

Query 72 CACCCTGGAATATTTATCGGAAATGATCAAATTTATAATGTAATTCGTTACTAGCTCATG 131

||||||||||||||||||||||||||||||||||||||||||||| |||||| |||||||

Sbjct 61 CACCCTGGAATATTTATCGGAAATGATCAAATTTATAATGTAATT-GTTACT-GCTCATG 118

Query 132 CATTTATTATAAttttttttATAGTAATACCTATTATAATTGGAGGATTTGGAAATTGGT 191

||||||||||||||||||||||||||||||||||||||||||||||||||||||||||||

Sbjct 119 CATTTATTATAATTTTTTTTATAGTAATACCTATTATAATTGGAGGATTTGGAAATTGGT 178

Query 192 TAGTCCCATTAATATTAGGTGCTCCTGATATAGCTTTCCCTCGAATAAATAATATAAGTT 251

||||||||||||||||||||||||||||||||||||||||||||||||||||||||||||

Sbjct 179 TAGTCCCATTAATATTAGGTGCTCCTGATATAGCTTTCCCTCGAATAAATAATATAAGTT 238

Query 252 TTTGAATACTTCCTCCCTCTTTAACTCTTTTAATTTCTAGATCTATAGTAGAAAATGGAG 311

||||||||||||||||||||||||||||||||||||||||||||||||||||||||||||

Sbjct 239 TTTGAATACTTCCTCCCTCTTTAACTCTTTTAATTTCTAGATCTATAGTAGAAAATGGAG 298

Query 312 CAGGAACAGGTTGAACTGTATACCCTCCTCTTTCTTCTGGAACTGCTCATGCAGGAGCTT 371

||||||||||||||||||||||||||||||||||||||||||||||||||||||||||||

Sbjct 299 CAGGAACAGGTTGAACTGTATACCCTCCTCTTTCTTCTGGAACTGCTCATGCAGGAGCTT 358

Query 372 CTGTAGATTTAGCTATTTTTTCTCTTCATTTAGCAGGAATTTCTTCTATTTTAGGAGCAG 431

||||||||||||||||||||||||||||||||||||||||||||||||||||||||||||

Sbjct 359 CTGTAGATTTAGCTATTTTTTCTCTTCATTTAGCAGGAATTTCTTCTATTTTAGGAGCAG 418

Query 432 TAAATTTTATTACAACTGTAATTAATATACGATCAACTGGAATTACACTTGATCGTTTAC 491

||||||||||||||||||||||||||||||||||||||||||||||||||||||||||||

Sbjct 419 TAAATTTTATTACAACTGTAATTAATATACGATCAACTGGAATTACACTTGATCGTTTAC 478

Query 492 CTTTATTTGTCTGATCTGTAATTATTACAGCTATTTTATTACTTCTTTCATTACCAGTTT 551

||||||||||||||||||||||||||||||||||||||||||||||||||||||||||||

Sbjct 479 CTTTATTTGTCTGATCTGTAATTATTACAGCTATTTTATTACTTCTTTCATTACCAGTTT 538

Query 552 TAGCAGGAGCTATTACTATATTATTAACTGATCGAAATTTTAATACATCATTTTTTGACC 611

||||||||||||||||||||||||||||||||||||||||||||||||||||||||||||

Sbjct 539 TAGCAGGAGCTATTACTATATTATTAACTGATCGAAATTTTAATACATCATTTTTTGACC 598

Query 612 CAATTGGAGGAGGTGACCCTATTCTTTATCAACATCTATTT 652

|||||||||||||||||||||||||||||||||||||||||

Sbjct 599 CAATTGGAGGAGGTGACCCTATTCTTTATCAACATCTATTT 639

>Aedes malayensis voucher DNUS-WCP4 cytochrome c oxidase subunit 1 (cox1) gene, partial cds; mitochondrial

Sequence ID: KY420724.1 Length: 661

Range 1: 13 to 655

Score:1162 bits(629), Expect:0.0,

Identities:641/646(99%), Gaps:4/646(0%), Strand: Plus/Plus

Query 6 TATTTTTGGAGTATGATCGGGGA-AGTCGGAACTTCATTAAGAGTTTTAATTCGAACTGA 64

||||||||||||||||||||||| ||||||||||||||||||||||||||||||||||||

Sbjct 13 TATTTTTGGAGTATGATCGGGGATAGTCGGAACTTCATTAAGAGTTTTAATTCGAACTGA 72

Query 65 ACTTAGTCACCCTGGAATATTTATCGGAAATGATCAAATTTATAATGTAATTCGTTACTA 124

|||||||||||||||||||||||||||||||||||||||||||||||||||| ||||||

Sbjct 73 ACTTAGTCACCCTGGAATATTTATCGGAAATGATCAAATTTATAATGTAATT-GTTACT- 130

Query 125 GCTCATGCATTTATTATAAttttttttATAGTAATACCTATTATAATTGGAGGATTTGGA 184

||||||||||||||||||||||||||||||||||||||||||||||||||||||||||||

Sbjct 131 GCTCATGCATTTATTATAATTTTTTTTATAGTAATACCTATTATAATTGGAGGATTTGGA 190

Query 185 AATTGGTTAGTCCCATTAATATTAGGTGCTCCTGATATAGCTTTCCCTCGAATAAATAAT 244

||||||||||||||||||||||||||||||||||||||||||||||||||||||||||||

Sbjct 191 AATTGGTTAGTCCCATTAATATTAGGTGCTCCTGATATAGCTTTCCCTCGAATAAATAAT 250

Query 245 ATAAGTTTTTGAATACTTCCTCCCTCTTTAACTCTTTTAATTTCTAGATCTATAGTAGAA 304

||||||||||||||||||||||||||||||||||||||||||||||||||||||||||||

Sbjct 251 ATAAGTTTTTGAATACTTCCTCCCTCTTTAACTCTTTTAATTTCTAGATCTATAGTAGAA 310

Query 305 AATGGAGCAGGAACAGGTTGAACTGTATACCCTCCTCTTTCTTCTGGAACTGCTCATGCA 364

||||||||||||||||||||||||||||||||||||||||||||||||||||||||||||

Sbjct 311 AATGGAGCAGGAACAGGTTGAACTGTATACCCTCCTCTTTCTTCTGGAACTGCTCATGCA 370

Query 365 GGAGCTTCTGTAGATTTAGCTATTTTTTCTCTTCATTTAGCAGGAATTTCTTCTATTTTA 424

||||||||||||||||||||||||||||||||||||||||||||||||||||||||||||

Sbjct 371 GGAGCTTCTGTAGATTTAGCTATTTTTTCTCTTCATTTAGCAGGAATTTCTTCTATTTTA 430

Query 425 GGAGCAGTAAATTTTATTACAACTGTAATTAATATACGATCAACTGGAATTACACTTGAT 484

||||||||||||||||||||||||||||||||||||||||||||||||||||||||||||

Sbjct 431 GGAGCAGTAAATTTTATTACAACTGTAATTAATATACGATCAACTGGAATTACACTTGAT 490

Query 485 CGTTTACCTTTATTTGTCTGATCTGTAATTATTACAGCTATTTTATTACTTCTTTCATTA 544

||| ||||||||||||||||||||||||||||||||||||||||||||||||||||||||

Sbjct 491 CGTATACCTTTATTTGTCTGATCTGTAATTATTACAGCTATTTTATTACTTCTTTCATTA 550

Query 545 CCAGTTTTAGCAGGAGCTATTACTATATTATTAACTGATCGAAATTTTAATACATCATTT 604

||||||||||||||||||||||||||||||||||||||||||||||||||||||||||||

Sbjct 551 CCAGTTTTAGCAGGAGCTATTACTATATTATTAACTGATCGAAATTTTAATACATCATTT 610

Query 605 TTTGACCCAATTGGAGGAGGTGACCCTATTCTTTATCAACATCTAT 650

|||||||||||||||||||||||||||||||||||||||||| |||

Sbjct 611 TTTGACCCAATTGGAGGAGGTGACCCTATTCTTTATCAACAT-TAT 655

>Aedes malayensis voucher DNUS-Clementi1 cytochrome c oxidase subunit 1 (cox1) gene, partial cds; mitochondrial

Sequence ID: KY420775.1 Length: 657

Range 1: 12 to 657

Score:1160 bits(628), Expect:0.0,

Identities:642/648(99%), Gaps:3/648(0%), Strand: Plus/Plus

Query 6 TATTTTTGGAGTATGATCGGGGA-AGTCGGAACTTCATTAAGAGTTTTAATTCGAACTGA 64

||||||||||||||||||||| | ||||||||||||||||||||||||||||||||||||

Sbjct 12 TATTTTTGGAGTATGATCGGGAATAGTCGGAACTTCATTAAGAGTTTTAATTCGAACTGA 71

Query 65 ACTTAGTCACCCTGGAATATTTATCGGAAATGATCAAATTTATAATGTAATTCGTTACTA 124

|||||||||||||||||||||||||||||||||||||||||||||||||||| ||||||

Sbjct 72 ACTTAGTCACCCTGGAATATTTATCGGAAATGATCAAATTTATAATGTAATT-GTTACT- 129

Query 125 GCTCATGCATTTATTATAAttttttttATAGTAATACCTATTATAATTGGAGGATTTGGA 184

||||||||||||||||||||||| ||||||||||||||||||||||||||||||||||||

Sbjct 130 GCTCATGCATTTATTATAATTTTCTTTATAGTAATACCTATTATAATTGGAGGATTTGGA 189

Query 185 AATTGGTTAGTCCCATTAATATTAGGTGCTCCTGATATAGCTTTCCCTCGAATAAATAAT 244

||||||||||||||||||||||||||||||||||||||||||||||||||||||||||||

Sbjct 190 AATTGGTTAGTCCCATTAATATTAGGTGCTCCTGATATAGCTTTCCCTCGAATAAATAAT 249

Query 245 ATAAGTTTTTGAATACTTCCTCCCTCTTTAACTCTTTTAATTTCTAGATCTATAGTAGAA 304

||||||||||||||||||||||||||||||||||||||||||||||||||||||||||||

Sbjct 250 ATAAGTTTTTGAATACTTCCTCCCTCTTTAACTCTTTTAATTTCTAGATCTATAGTAGAA 309

Query 305 AATGGAGCAGGAACAGGTTGAACTGTATACCCTCCTCTTTCTTCTGGAACTGCTCATGCA 364

||||||||||||||||||||||||||||||||||||||||||||||||||||||||||||

Sbjct 310 AATGGAGCAGGAACAGGTTGAACTGTATACCCTCCTCTTTCTTCTGGAACTGCTCATGCA 369

Query 365 GGAGCTTCTGTAGATTTAGCTATTTTTTCTCTTCATTTAGCAGGAATTTCTTCTATTTTA 424

||||||||||||||||||||||||||||||||||||||||||||||||||||||||||||

Sbjct 370 GGAGCTTCTGTAGATTTAGCTATTTTTTCTCTTCATTTAGCAGGAATTTCTTCTATTTTA 429

Query 425 GGAGCAGTAAATTTTATTACAACTGTAATTAATATACGATCAACTGGAATTACACTTGAT 484

||||||||||||||||||||||||||||||||||||||||||||||||||||||||||||

Sbjct 430 GGAGCAGTAAATTTTATTACAACTGTAATTAATATACGATCAACTGGAATTACACTTGAT 489

Query 485 CGTTTACCTTTATTTGTCTGATCTGTAATTATTACAGCTATTTTATTACTTCTTTCATTA 544

|| |||||||||||||||||||||||||||||||||||||||||||||||||||||||||

Sbjct 490 CGCTTACCTTTATTTGTCTGATCTGTAATTATTACAGCTATTTTATTACTTCTTTCATTA 549

Query 545 CCAGTTTTAGCAGGAGCTATTACTATATTATTAACTGATCGAAATTTTAATACATCATTT 604

||||||||||||||||||||||||||||||||||||||||||||||||||||||||||||

Sbjct 550 CCAGTTTTAGCAGGAGCTATTACTATATTATTAACTGATCGAAATTTTAATACATCATTT 609

Query 605 TTTGACCCAATTGGAGGAGGTGACCCTATTCTTTATCAACATCTATTT 652

||||||||||||||||||||||||||||||||||||||||||||||||

Sbjct 610 TTTGACCCAATTGGAGGAGGTGACCCTATTCTTTATCAACATCTATTT 657

>Aedes malayensis voucher DNUS-Sentosa8 cytochrome c oxidase subunit 1 (cox1) gene, partial cds; mitochondrial

Sequence ID: KY420772.1 Length: 658

>Aedes malayensis voucher DNUS-Clementi6 cytochrome c oxidase subunit 1 (cox1) gene, partial cds; mitochondrial

Sequence ID: KY420773.1 Length: 658

>Aedes malayensis voucher DNUS-Clementi3 cytochrome c oxidase subunit 1 (cox1) gene, partial cds; mitochondrial

Sequence ID: KY420774.1 Length: 658

Range 1: 13 to 658

Score:1160 bits(628), Expect:0.0,

Identities:642/648(99%), Gaps:3/648(0%), Strand: Plus/Plus

Query 6 TATTTTTGGAGTATGATCGGGGA-AGTCGGAACTTCATTAAGAGTTTTAATTCGAACTGA 64

||||||||||||||||||||| | ||||||||||||||||||||||||||||||||||||

Sbjct 13 TATTTTTGGAGTATGATCGGGAATAGTCGGAACTTCATTAAGAGTTTTAATTCGAACTGA 72

Query 65 ACTTAGTCACCCTGGAATATTTATCGGAAATGATCAAATTTATAATGTAATTCGTTACTA 124

|||||||||||||||||||||||||||||||||||||||||||||||||||| ||||||

Sbjct 73 ACTTAGTCACCCTGGAATATTTATCGGAAATGATCAAATTTATAATGTAATT-GTTACT- 130

Query 125 GCTCATGCATTTATTATAAttttttttATAGTAATACCTATTATAATTGGAGGATTTGGA 184

||||||||||||||||||||||| ||||||||||||||||||||||||||||||||||||

Sbjct 131 GCTCATGCATTTATTATAATTTTCTTTATAGTAATACCTATTATAATTGGAGGATTTGGA 190

Query 185 AATTGGTTAGTCCCATTAATATTAGGTGCTCCTGATATAGCTTTCCCTCGAATAAATAAT 244

||||||||||||||||||||||||||||||||||||||||||||||||||||||||||||

Sbjct 191 AATTGGTTAGTCCCATTAATATTAGGTGCTCCTGATATAGCTTTCCCTCGAATAAATAAT 250

Query 245 ATAAGTTTTTGAATACTTCCTCCCTCTTTAACTCTTTTAATTTCTAGATCTATAGTAGAA 304

||||||||||||||||||||||||||||||||||||||||||||||||||||||||||||

Sbjct 251 ATAAGTTTTTGAATACTTCCTCCCTCTTTAACTCTTTTAATTTCTAGATCTATAGTAGAA 310

Query 305 AATGGAGCAGGAACAGGTTGAACTGTATACCCTCCTCTTTCTTCTGGAACTGCTCATGCA 364

||||||||||||||||||||||||||||||||||||||||||||||||||||||||||||

Sbjct 311 AATGGAGCAGGAACAGGTTGAACTGTATACCCTCCTCTTTCTTCTGGAACTGCTCATGCA 370

Query 365 GGAGCTTCTGTAGATTTAGCTATTTTTTCTCTTCATTTAGCAGGAATTTCTTCTATTTTA 424

||||||||||||||||||||||||||||||||||||||||||||||||||||||||||||

Sbjct 371 GGAGCTTCTGTAGATTTAGCTATTTTTTCTCTTCATTTAGCAGGAATTTCTTCTATTTTA 430

Query 425 GGAGCAGTAAATTTTATTACAACTGTAATTAATATACGATCAACTGGAATTACACTTGAT 484

||||||||||||||||||||||||||||||||||||||||||||||||||||||||||||

Sbjct 431 GGAGCAGTAAATTTTATTACAACTGTAATTAATATACGATCAACTGGAATTACACTTGAT 490

Query 485 CGTTTACCTTTATTTGTCTGATCTGTAATTATTACAGCTATTTTATTACTTCTTTCATTA 544

|| |||||||||||||||||||||||||||||||||||||||||||||||||||||||||

Sbjct 491 CGCTTACCTTTATTTGTCTGATCTGTAATTATTACAGCTATTTTATTACTTCTTTCATTA 550

Query 545 CCAGTTTTAGCAGGAGCTATTACTATATTATTAACTGATCGAAATTTTAATACATCATTT 604

||||||||||||||||||||||||||||||||||||||||||||||||||||||||||||

Sbjct 551 CCAGTTTTAGCAGGAGCTATTACTATATTATTAACTGATCGAAATTTTAATACATCATTT 610

Query 605 TTTGACCCAATTGGAGGAGGTGACCCTATTCTTTATCAACATCTATTT 652

||||||||||||||||||||||||||||||||||||||||||||||||

Sbjct 611 TTTGACCCAATTGGAGGAGGTGACCCTATTCTTTATCAACATCTATTT 658

>Aedes albopictus isolate AB1S5_inB cytochrome oxidase subunit 1 (COI) gene, partial cds; mitochondrial

Sequence ID: KM613085.1 Length: 639

>Aedes albopictus isolate AB22_inP cytochrome oxidase subunit 1 (COI) gene, partial cds; mitochondrial

Sequence ID: KM613113.1 Length: 639

>Aedes albopictus isolate AB24_inP cytochrome oxidase subunit 1 (COI) gene, partial cds; mitochondrial

Sequence ID: KM613115.1 Length: 639

Range 1: 1 to 639

Score:1158 bits(627), Expect:0.0,

Identities:637/641(99%), Gaps:3/641(0%), Strand: Plus/Plus

Query 13 GGAGTATGATCGGGGA-AGTCGGAACTTCATTAAGAGTTTTAATTCGAACTGAACTTAGT 71

|||||||||||||| | |||||||||||||||||||||||||||||||||||||||||||

Sbjct 1 GGAGTATGATCGGGAATAGTCGGAACTTCATTAAGAGTTTTAATTCGAACTGAACTTAGT 60

Query 72 CACCCTGGAATATTTATCGGAAATGATCAAATTTATAATGTAATTCGTTACTAGCTCATG 131

||||||||||||||||||||||||||||||||||||||||||||| |||||| |||||||

Sbjct 61 CACCCTGGAATATTTATCGGAAATGATCAAATTTATAATGTAATT-GTTACT-GCTCATG 118

Query 132 CATTTATTATAAttttttttATAGTAATACCTATTATAATTGGAGGATTTGGAAATTGGT 191

||||||||||||||||||||||||||||||||||||||||||||||||||||||||||||

Sbjct 119 CATTTATTATAATTTTTTTTATAGTAATACCTATTATAATTGGAGGATTTGGAAATTGGT 178

Query 192 TAGTCCCATTAATATTAGGTGCTCCTGATATAGCTTTCCCTCGAATAAATAATATAAGTT 251

||||||||||||||||||||||||||||||||||||||||||||||||||||||||||||

Sbjct 179 TAGTCCCATTAATATTAGGTGCTCCTGATATAGCTTTCCCTCGAATAAATAATATAAGTT 238

Query 252 TTTGAATACTTCCTCCCTCTTTAACTCTTTTAATTTCTAGATCTATAGTAGAAAATGGAG 311

||||||||||||||||||||||||||||||||||||||||||||||||||||||||||||

Sbjct 239 TTTGAATACTTCCTCCCTCTTTAACTCTTTTAATTTCTAGATCTATAGTAGAAAATGGAG 298

Query 312 CAGGAACAGGTTGAACTGTATACCCTCCTCTTTCTTCTGGAACTGCTCATGCAGGAGCTT 371

||||||||||||||||||||||||||||||||||||||||||||||||||||||||||||

Sbjct 299 CAGGAACAGGTTGAACTGTATACCCTCCTCTTTCTTCTGGAACTGCTCATGCAGGAGCTT 358

Query 372 CTGTAGATTTAGCTATTTTTTCTCTTCATTTAGCAGGAATTTCTTCTATTTTAGGAGCAG 431

||||||||||||||||||||||||||||||||||||||||||||||||||||||||||||

Sbjct 359 CTGTAGATTTAGCTATTTTTTCTCTTCATTTAGCAGGAATTTCTTCTATTTTAGGAGCAG 418

Query 432 TAAATTTTATTACAACTGTAATTAATATACGATCAACTGGAATTACACTTGATCGTTTAC 491

||||||||||||||||||||||||||||||||||||||||||||||||||||||||||||

Sbjct 419 TAAATTTTATTACAACTGTAATTAATATACGATCAACTGGAATTACACTTGATCGTTTAC 478

Query 492 CTTTATTTGTCTGATCTGTAATTATTACAGCTATTTTATTACTTCTTTCATTACCAGTTT 551

||||||||||||||||||||||||||||||||||||||||||||||||||||||||||||

Sbjct 479 CTTTATTTGTCTGATCTGTAATTATTACAGCTATTTTATTACTTCTTTCATTACCAGTTT 538

Query 552 TAGCAGGAGCTATTACTATATTATTAACTGATCGAAATTTTAATACATCATTTTTTGACC 611

||||||||||||||||||||||||||||||||||||||||||||||||||||||||||||

Sbjct 539 TAGCAGGAGCTATTACTATATTATTAACTGATCGAAATTTTAATACATCATTTTTTGACC 598

Query 612 CAATTGGAGGAGGTGACCCTATTCTTTATCAACATCTATTT 652

|||||||||||||||||||||||||||||||||||||||||

Sbjct 599 CAATTGGAGGAGGTGACCCTATTCTTTATCAACATCTATTT 639

>Aedes albopictus isolate AB1_04B cytochrome oxidase subunit 1 (COI) gene, partial cds; mitochondrial

Sequence ID: KM613083.1 Length: 639

>Aedes albopictus isolate AB2S2_04B cytochrome oxidase subunit 1 (COI) gene, partial cds; mitochondrial

Sequence ID: KM613088.1 Length: 639

>Aedes albopictus isolate AB3S3_04B cytochrome oxidase subunit 1 (COI) gene, partial cds; mitochondrial

Sequence ID: KM613091.1 Length: 639

Range 1: 1 to 639

Score:1158 bits(627), Expect:0.0,

Identities:637/641(99%), Gaps:3/641(0%), Strand: Plus/Plus

Query 13 GGAGTATGATCGGGGA-AGTCGGAACTTCATTAAGAGTTTTAATTCGAACTGAACTTAGT 71

|||||||||||||||| |||||||||||||||||||||||||||||||||||||||||||

Sbjct 1 GGAGTATGATCGGGGATAGTCGGAACTTCATTAAGAGTTTTAATTCGAACTGAACTTAGT 60

Query 72 CACCCTGGAATATTTATCGGAAATGATCAAATTTATAATGTAATTCGTTACTAGCTCATG 131

|| |||||||||||||||||||||||||||||||||||||||||| |||||| |||||||

Sbjct 61 CATCCTGGAATATTTATCGGAAATGATCAAATTTATAATGTAATT-GTTACT-GCTCATG 118

Query 132 CATTTATTATAAttttttttATAGTAATACCTATTATAATTGGAGGATTTGGAAATTGGT 191

||||||||||||||||||||||||||||||||||||||||||||||||||||||||||||

Sbjct 119 CATTTATTATAATTTTTTTTATAGTAATACCTATTATAATTGGAGGATTTGGAAATTGGT 178

Query 192 TAGTCCCATTAATATTAGGTGCTCCTGATATAGCTTTCCCTCGAATAAATAATATAAGTT 251

||||||||||||||||||||||||||||||||||||||||||||||||||||||||||||

Sbjct 179 TAGTCCCATTAATATTAGGTGCTCCTGATATAGCTTTCCCTCGAATAAATAATATAAGTT 238

Query 252 TTTGAATACTTCCTCCCTCTTTAACTCTTTTAATTTCTAGATCTATAGTAGAAAATGGAG 311

||||||||||||||||||||||||||||||||||||||||||||||||||||||||||||

Sbjct 239 TTTGAATACTTCCTCCCTCTTTAACTCTTTTAATTTCTAGATCTATAGTAGAAAATGGAG 298

Query 312 CAGGAACAGGTTGAACTGTATACCCTCCTCTTTCTTCTGGAACTGCTCATGCAGGAGCTT 371

||||||||||||||||||||||||||||||||||||||||||||||||||||||||||||

Sbjct 299 CAGGAACAGGTTGAACTGTATACCCTCCTCTTTCTTCTGGAACTGCTCATGCAGGAGCTT 358

Query 372 CTGTAGATTTAGCTATTTTTTCTCTTCATTTAGCAGGAATTTCTTCTATTTTAGGAGCAG 431

||||||||||||||||||||||||||||||||||||||||||||||||||||||||||||

Sbjct 359 CTGTAGATTTAGCTATTTTTTCTCTTCATTTAGCAGGAATTTCTTCTATTTTAGGAGCAG 418

Query 432 TAAATTTTATTACAACTGTAATTAATATACGATCAACTGGAATTACACTTGATCGTTTAC 491

||||||||||||||||||||||||||||||||||||||||||||||||||||||||||||

Sbjct 419 TAAATTTTATTACAACTGTAATTAATATACGATCAACTGGAATTACACTTGATCGTTTAC 478

Query 492 CTTTATTTGTCTGATCTGTAATTATTACAGCTATTTTATTACTTCTTTCATTACCAGTTT 551

||||||||||||||||||||||||||||||||||||||||||||||||||||||||||||

Sbjct 479 CTTTATTTGTCTGATCTGTAATTATTACAGCTATTTTATTACTTCTTTCATTACCAGTTT 538

Query 552 TAGCAGGAGCTATTACTATATTATTAACTGATCGAAATTTTAATACATCATTTTTTGACC 611

||||||||||||||||||||||||||||||||||||||||||||||||||||||||||||

Sbjct 539 TAGCAGGAGCTATTACTATATTATTAACTGATCGAAATTTTAATACATCATTTTTTGACC 598

Query 612 CAATTGGAGGAGGTGACCCTATTCTTTATCAACATCTATTT 652

|||||||||||||||||||||||||||||||||||||||||

Sbjct 599 CAATTGGAGGAGGTGACCCTATTCTTTATCAACATCTATTT 639

>Aedes scutellaris isolate scu2 cytochrome oxidase subunit 1 (COI) gene, partial cds; mitochondrial

Sequence ID: KP843373.1 Length: 658

>Aedes scutellaris isolate scu3 cytochrome oxidase subunit 1 (COI) gene, partial cds; mitochondrial

Sequence ID: KP843374.1 Length: 658

>Aedes scutellaris isolate scu7 cytochrome oxidase subunit 1 (COI) gene, partial cds; mitochondrial

Sequence ID: KP843378.1 Length: 658

>Aedes scutellaris isolate scu8 cytochrome oxidase subunit 1 (COI) gene, partial cds; mitochondrial

Sequence ID: KP843379.1 Length: 658

Range 1: 13 to 658

Score:1149 bits(622), Expect:0.0,

Identities:640/648(99%), Gaps:3/648(0%), Strand: Plus/Plus

Query 6 TATTTTTGGAGTATGATCGGGGA-AGTCGGAACTTCATTAAGAGTTTTAATTCGAACTGA 64

|||||||||||||||||| || | ||||||||||||||||||||||||||||||||||||

Sbjct 13 TATTTTTGGAGTATGATCTGGAATAGTCGGAACTTCATTAAGAGTTTTAATTCGAACTGA 72

Query 65 ACTTAGTCACCCTGGAATATTTATCGGAAATGATCAAATTTATAATGTAATTCGTTACTA 124

|||||||||||||||||||||||||||||||||||||||||||||||||||| ||||||

Sbjct 73 ACTTAGTCACCCTGGAATATTTATCGGAAATGATCAAATTTATAATGTAATT-GTTACT- 130

Query 125 GCTCATGCATTTATTATAAttttttttATAGTAATACCTATTATAATTGGAGGATTTGGA 184

||||||||||||||||||||||||||||||||||||||||||||||||||||||||||||

Sbjct 131 GCTCATGCATTTATTATAATTTTTTTTATAGTAATACCTATTATAATTGGAGGATTTGGA 190

Query 185 AATTGGTTAGTCCCATTAATATTAGGTGCTCCTGATATAGCTTTCCCTCGAATAAATAAT 244

||||||||||||||||||||||||||||||||||||||||||||||||||||||||||||

Sbjct 191 AATTGGTTAGTCCCATTAATATTAGGTGCTCCTGATATAGCTTTCCCTCGAATAAATAAT 250

Query 245 ATAAGTTTTTGAATACTTCCTCCCTCTTTAACTCTTTTAATTTCTAGATCTATAGTAGAA 304

|||||||||||||||||||||||||||||||| |||||| ||||||||||||||||||||

Sbjct 251 ATAAGTTTTTGAATACTTCCTCCCTCTTTAACCCTTTTACTTTCTAGATCTATAGTAGAA 310

Query 305 AATGGAGCAGGAACAGGTTGAACTGTATACCCTCCTCTTTCTTCTGGAACTGCTCATGCA 364

||||||||||||||||||||||||||||||||||||||||||||||||||||||||||||

Sbjct 311 AATGGAGCAGGAACAGGTTGAACTGTATACCCTCCTCTTTCTTCTGGAACTGCTCATGCA 370

Query 365 GGAGCTTCTGTAGATTTAGCTATTTTTTCTCTTCATTTAGCAGGAATTTCTTCTATTTTA 424

||||||||||||||||||||||||||||||||||||||||||||||||||||||||||||

Sbjct 371 GGAGCTTCTGTAGATTTAGCTATTTTTTCTCTTCATTTAGCAGGAATTTCTTCTATTTTA 430

Query 425 GGAGCAGTAAATTTTATTACAACTGTAATTAATATACGATCAACTGGAATTACACTTGAT 484

||||||||||||||||||||||||||||||||||||||||||||||||||||||||||||

Sbjct 431 GGAGCAGTAAATTTTATTACAACTGTAATTAATATACGATCAACTGGAATTACACTTGAT 490

Query 485 CGTTTACCTTTATTTGTCTGATCTGTAATTATTACAGCTATTTTATTACTTCTTTCATTA 544

||||||||||||||||||||||| ||||||||||||||||||||||||||||||||||||

Sbjct 491 CGTTTACCTTTATTTGTCTGATCAGTAATTATTACAGCTATTTTATTACTTCTTTCATTA 550

Query 545 CCAGTTTTAGCAGGAGCTATTACTATATTATTAACTGATCGAAATTTTAATACATCATTT 604

||||||||||||||||||||||||||||||||||||||||||||||||||||||||||||

Sbjct 551 CCAGTTTTAGCAGGAGCTATTACTATATTATTAACTGATCGAAATTTTAATACATCATTT 610

Query 605 TTTGACCCAATTGGAGGAGGTGACCCTATTCTTTATCAACATCTATTT 652

||||||||||||||||||||||||||||||||||||||||||||||||

Sbjct 611 TTTGACCCAATTGGAGGAGGTGACCCTATTCTTTATCAACATCTATTT 658

>Aedes malayensis isolate NN519 cytochrome c oxidase subunit 1 (COI) gene, partial cds; mitochondrial

Sequence ID: MG921173.1 Length: 658

>Aedes malayensis isolate NN520 cytochrome c oxidase subunit 1 (COI) gene, partial cds; mitochondrial

Sequence ID: MG921174.1 Length: 658

>Aedes malayensis isolate NN521 cytochrome c oxidase subunit 1 (COI) gene, partial cds; mitochondrial

Sequence ID: MG921175.1 Length: 658

>Aedes malayensis isolate NN523 cytochrome c oxidase subunit 1 (COI) gene, partial cds; mitochondrial

Sequence ID: MG921176.1 Length: 658

Range 1: 13 to 658

Score:1144 bits(619), Expect:0.0,

Identities:639/648(99%), Gaps:3/648(0%), Strand: Plus/Plus

Query 6 TATTTTTGGAGTATGATCGGGGA-AGTCGGAACTTCATTAAGAGTTTTAATTCGAACTGA 64

|||||||||||||||||| || | ||||||||||||||||||||||||||||||||||||

Sbjct 13 TATTTTTGGAGTATGATCTGGAATAGTCGGAACTTCATTAAGAGTTTTAATTCGAACTGA 72

Query 65 ACTTAGTCACCCTGGAATATTTATCGGAAATGATCAAATTTATAATGTAATTCGTTACTA 124

|||||||||||||||||||||||||||||||||||||||||||||||||||| ||||||

Sbjct 73 ACTTAGTCACCCTGGAATATTTATCGGAAATGATCAAATTTATAATGTAATT-GTTACT- 130

Query 125 GCTCATGCATTTATTATAAttttttttATAGTAATACCTATTATAATTGGAGGATTTGGA 184

||||||||||||||||||||||||||||||||||||||||||||||||||||||||||||

Sbjct 131 GCTCATGCATTTATTATAATTTTTTTTATAGTAATACCTATTATAATTGGAGGATTTGGA 190

Query 185 AATTGGTTAGTCCCATTAATATTAGGTGCTCCTGATATAGCTTTCCCTCGAATAAATAAT 244

||||| ||||||||||||||||||||||||||||||||||||||||||||||||||||||

Sbjct 191 AATTGATTAGTCCCATTAATATTAGGTGCTCCTGATATAGCTTTCCCTCGAATAAATAAT 250

Query 245 ATAAGTTTTTGAATACTTCCTCCCTCTTTAACTCTTTTAATTTCTAGATCTATAGTAGAA 304

|||||||||||||||||||||||||||||||| |||||| ||||||||||||||||||||

Sbjct 251 ATAAGTTTTTGAATACTTCCTCCCTCTTTAACCCTTTTACTTTCTAGATCTATAGTAGAA 310

Query 305 AATGGAGCAGGAACAGGTTGAACTGTATACCCTCCTCTTTCTTCTGGAACTGCTCATGCA 364

||||||||||||||||||||||||||||||||||||||||||||||||||||||||||||

Sbjct 311 AATGGAGCAGGAACAGGTTGAACTGTATACCCTCCTCTTTCTTCTGGAACTGCTCATGCA 370

Query 365 GGAGCTTCTGTAGATTTAGCTATTTTTTCTCTTCATTTAGCAGGAATTTCTTCTATTTTA 424

||||||||||||||||||||||||||||||||||||||||||||||||||||||||||||

Sbjct 371 GGAGCTTCTGTAGATTTAGCTATTTTTTCTCTTCATTTAGCAGGAATTTCTTCTATTTTA 430

Query 425 GGAGCAGTAAATTTTATTACAACTGTAATTAATATACGATCAACTGGAATTACACTTGAT 484

||||||||||||||||||||||||||||||||||||||||||||||||||||||||||||

Sbjct 431 GGAGCAGTAAATTTTATTACAACTGTAATTAATATACGATCAACTGGAATTACACTTGAT 490

Query 485 CGTTTACCTTTATTTGTCTGATCTGTAATTATTACAGCTATTTTATTACTTCTTTCATTA 544

||||||||||||||||||||||| ||||||||||||||||||||||||||||||||||||

Sbjct 491 CGTTTACCTTTATTTGTCTGATCAGTAATTATTACAGCTATTTTATTACTTCTTTCATTA 550

Query 545 CCAGTTTTAGCAGGAGCTATTACTATATTATTAACTGATCGAAATTTTAATACATCATTT 604

||||||||||||||||||||||||||||||||||||||||||||||||||||||||||||

Sbjct 551 CCAGTTTTAGCAGGAGCTATTACTATATTATTAACTGATCGAAATTTTAATACATCATTT 610

Query 605 TTTGACCCAATTGGAGGAGGTGACCCTATTCTTTATCAACATCTATTT 652

||||||||||||||||||||||||||||||||||||||||||||||||

Sbjct 611 TTTGACCCAATTGGAGGAGGTGACCCTATTCTTTATCAACATCTATTT 658

>Aedes malayensis voucher DNUS-WCP8 cytochrome c oxidase subunit 1 (cox1) gene, partial cds; mitochondrial

Sequence ID: KY420813.1 Length: 635

Range 1: 1 to 635

Score:1140 bits(617), Expect:0.0,

Identities:631/637(99%), Gaps:3/637(0%), Strand: Plus/Plus

Query 17 TATGATCGGGGA-AGTCGGAACTTCATTAAGAGTTTTAATTCGAACTGAACTTAGTCACC 75

|||||||||| | ||||||||||||||||||||||||||||||||||||||||||||| |

Sbjct 1 TATGATCGGGAATAGTCGGAACTTCATTAAGAGTTTTAATTCGAACTGAACTTAGTCATC 60

Query 76 CTGGAATATTTATCGGAAATGATCAAATTTATAATGTAATTCGTTACTAGCTCATGCATT 135

||||||||||||||||||||||||||||||||||||||||| |||||| |||||||||||

Sbjct 61 CTGGAATATTTATCGGAAATGATCAAATTTATAATGTAATT-GTTACT-GCTCATGCATT 118

Query 136 TATTATAAttttttttATAGTAATACCTATTATAATTGGAGGATTTGGAAATTGGTTAGT 195

||||||||||||||||||||||||||||||||||||||||||||||||||||||||||||

Sbjct 119 TATTATAATTTTTTTTATAGTAATACCTATTATAATTGGAGGATTTGGAAATTGGTTAGT 178

Query 196 CCCATTAATATTAGGTGCTCCTGATATAGCTTTCCCTCGAATAAATAATATAAGTTTTTG 255

||||||||||||||||||||||||||||||||||||||||||||||||||||||||||||

Sbjct 179 CCCATTAATATTAGGTGCTCCTGATATAGCTTTCCCTCGAATAAATAATATAAGTTTTTG 238

Query 256 AATACTTCCTCCCTCTTTAACTCTTTTAATTTCTAGATCTATAGTAGAAAATGGAGCAGG 315

||||||||||||||||||||||||||||||||||||||||||||||||||||||||||||

Sbjct 239 AATACTTCCTCCCTCTTTAACTCTTTTAATTTCTAGATCTATAGTAGAAAATGGAGCAGG 298

Query 316 AACAGGTTGAACTGTATACCCTCCTCTTTCTTCTGGAACTGCTCATGCAGGAGCTTCTGT 375

||||||||||||||||||||||||||||||||||||||||||||||||||||||||||||

Sbjct 299 AACAGGTTGAACTGTATACCCTCCTCTTTCTTCTGGAACTGCTCATGCAGGAGCTTCTGT 358

Query 376 AGATTTAGCTATTTTTTCTCTTCATTTAGCAGGAATTTCTTCTATTTTAGGAGCAGTAAA 435

||||||||||||||||||||||||||||||||||||||||||||||||||||||||||||

Sbjct 359 AGATTTAGCTATTTTTTCTCTTCATTTAGCAGGAATTTCTTCTATTTTAGGAGCAGTAAA 418

Query 436 TTTTATTACAACTGTAATTAATATACGATCAACTGGAATTACACTTGATCGTTTACCTTT 495

||||||||||||||||||||||||||||||||||||||||||||||||||| ||||||||

Sbjct 419 TTTTATTACAACTGTAATTAATATACGATCAACTGGAATTACACTTGATCGCTTACCTTT 478

Query 496 ATTTGTCTGATCTGTAATTATTACAGCTATTTTATTACTTCTTTCATTACCAGTTTTAGC 555

||||||||||||||||||||||||||||||||||||||||||||||||||||||||||||

Sbjct 479 ATTTGTCTGATCTGTAATTATTACAGCTATTTTATTACTTCTTTCATTACCAGTTTTAGC 538

Query 556 AGGAGCTATTACTATATTATTAACTGATCGAAATTTTAATACATCATTTTTTGACCCAAT 615

||||||||||||||||||||||||||||||||||||||||||||||||||||||||||||

Sbjct 539 AGGAGCTATTACTATATTATTAACTGATCGAAATTTTAATACATCATTTTTTGACCCAAT 598

Query 616 TGGAGGAGGTGACCCTATTCTTTATCAACATCTATTT 652

|||||||||||||||||||||||||||||||||||||

Sbjct 599 TGGAGGAGGTGACCCTATTCTTTATCAACATCTATTT 635

>Aedes albopictus isolate AA004CK cytochrome oxidase subunit 1 (COI) gene, partial cds; mitochondrial

Sequence ID: KM613121.1 Length: 639

>Aedes albopictus isolate AA008CK cytochrome oxidase subunit 1 (COI) gene, partial cds; mitochondrial

Sequence ID: KM613122.1 Length: 639

>Aedes albopictus isolate AA1CK cytochrome oxidase subunit 1 (COI) gene, partial cds; mitochondrial

Sequence ID: KM613123.1 Length: 639

>Aedes albopictus isolate AA2CK cytochrome oxidase subunit 1 (COI) gene, partial cds; mitochondrial

Sequence ID: KM613124.1 Length: 639

>Aedes albopictus isolate AA3CK cytochrome oxidase subunit 1 (COI) gene, partial cds; mitochondrial

Sequence ID: KM613125.1 Length: 639

>Aedes albopictus isolate AA4CK cytochrome oxidase subunit 1 (COI) gene, partial cds; mitochondrial

Sequence ID: KM613126.1 Length: 639

>Aedes albopictus isolate AA6CK cytochrome oxidase subunit 1 (COI) gene, partial cds; mitochondrial

Sequence ID: KM613127.1 Length: 639

>Aedes albopictus isolate AA7CK cytochrome oxidase subunit 1 (COI) gene, partial cds; mitochondrial

Sequence ID: KM613128.1 Length: 639

>Aedes albopictus isolate AA8CK cytochrome oxidase subunit 1 (COI) gene, partial cds; mitochondrial

Sequence ID: KM613129.1 Length: 639

Range 1: 1 to 639

Score:1136 bits(615), Expect:0.0,

Identities:633/641(99%), Gaps:3/641(0%), Strand: Plus/Plus

Query 13 GGAGTATGATCGGGGA-AGTCGGAACTTCATTAAGAGTTTTAATTCGAACTGAACTTAGT 71

||||||||||| || | |||||||||||||||||||||||||||||||||||||||||||

Sbjct 1 GGAGTATGATCTGGAATAGTCGGAACTTCATTAAGAGTTTTAATTCGAACTGAACTTAGT 60

Query 72 CACCCTGGAATATTTATCGGAAATGATCAAATTTATAATGTAATTCGTTACTAGCTCATG 131

||||||||||||||||||||||||||||||||||||||||||||| |||||| |||||||

Sbjct 61 CACCCTGGAATATTTATCGGAAATGATCAAATTTATAATGTAATT-GTTACT-GCTCATG 118

Query 132 CATTTATTATAAttttttttATAGTAATACCTATTATAATTGGAGGATTTGGAAATTGGT 191

||||||||||||||||||||||||||||||||||||||||||||||||||||||||||||

Sbjct 119 CATTTATTATAATTTTTTTTATAGTAATACCTATTATAATTGGAGGATTTGGAAATTGGT 178

Query 192 TAGTCCCATTAATATTAGGTGCTCCTGATATAGCTTTCCCTCGAATAAATAATATAAGTT 251

||||||||||||||||||||||||||||||||||||||||||||||||||||||||||||

Sbjct 179 TAGTCCCATTAATATTAGGTGCTCCTGATATAGCTTTCCCTCGAATAAATAATATAAGTT 238

Query 252 TTTGAATACTTCCTCCCTCTTTAACTCTTTTAATTTCTAGATCTATAGTAGAAAATGGAG 311

||||||||||||||||||||||||| |||||| |||||||||||||||||||||||||||

Sbjct 239 TTTGAATACTTCCTCCCTCTTTAACCCTTTTACTTTCTAGATCTATAGTAGAAAATGGAG 298

Query 312 CAGGAACAGGTTGAACTGTATACCCTCCTCTTTCTTCTGGAACTGCTCATGCAGGAGCTT 371

||||||||||||||||||||||||||||||||||||||||||||||||||||||||||||

Sbjct 299 CAGGAACAGGTTGAACTGTATACCCTCCTCTTTCTTCTGGAACTGCTCATGCAGGAGCTT 358

Query 372 CTGTAGATTTAGCTATTTTTTCTCTTCATTTAGCAGGAATTTCTTCTATTTTAGGAGCAG 431

||||||||||||||||||||||||||||||||||||||||||||||||||||||||||||

Sbjct 359 CTGTAGATTTAGCTATTTTTTCTCTTCATTTAGCAGGAATTTCTTCTATTTTAGGAGCAG 418

Query 432 TAAATTTTATTACAACTGTAATTAATATACGATCAACTGGAATTACACTTGATCGTTTAC 491

||||||||||||||||||||||||||||||||||||||||||||||||||||||||||||

Sbjct 419 TAAATTTTATTACAACTGTAATTAATATACGATCAACTGGAATTACACTTGATCGTTTAC 478

Query 492 CTTTATTTGTCTGATCTGTAATTATTACAGCTATTTTATTACTTCTTTCATTACCAGTTT 551

|||||||||||||||| |||||||||||||||||||||||||||||||||||||||||||

Sbjct 479 CTTTATTTGTCTGATCAGTAATTATTACAGCTATTTTATTACTTCTTTCATTACCAGTTT 538

Query 552 TAGCAGGAGCTATTACTATATTATTAACTGATCGAAATTTTAATACATCATTTTTTGACC 611

||||||||||||||||||||||||||||||||||||||||||||||||||||||||||||

Sbjct 539 TAGCAGGAGCTATTACTATATTATTAACTGATCGAAATTTTAATACATCATTTTTTGACC 598

Query 612 CAATTGGAGGAGGTGACCCTATTCTTTATCAACATCTATTT 652

|||||||||||||||||||||||||||||||||||||||||

Sbjct 599 CAATTGGAGGAGGTGACCCTATTCTTTATCAACATCTATTT 639

>Aedes malayensis voucher DNUS-Sentosa4 cytochrome c oxidase subunit 1 (cox1) gene, partial cds; mitochondrial

Sequence ID: KY420769.1 Length: 621

Range 1: 1 to 621

Score:1133 bits(613), Expect:0.0,

Identities:620/623(99%), Gaps:2/623(0%), Strand: Plus/Plus

Query 30 GTCGGAACTTCATTAAGAGTTTTAATTCGAACTGAACTTAGTCACCCTGGAATATTTATC 89

||||||||||||||||||||||||||||||||||||||||||||||||||||||||||||

Sbjct 1 GTCGGAACTTCATTAAGAGTTTTAATTCGAACTGAACTTAGTCACCCTGGAATATTTATC 60

Query 90 GGAAATGATCAAATTTATAATGTAATTCGTTACTAGCTCATGCATTTATTATAAtttttt 149

||||||||||||||||||||||||||| |||||| |||||||||||||||||||||||||

Sbjct 61 GGAAATGATCAAATTTATAATGTAATT-GTTACT-GCTCATGCATTTATTATAATTTTTT 118

Query 150 ttATAGTAATACCTATTATAATTGGAGGATTTGGAAATTGGTTAGTCCCATTAATATTAG 209

||||||||||||||||||||||||||||||||||||||||||||||||||||||||||||

Sbjct 119 TTATAGTAATACCTATTATAATTGGAGGATTTGGAAATTGGTTAGTCCCATTAATATTAG 178

Query 210 GTGCTCCTGATATAGCTTTCCCTCGAATAAATAATATAAGTTTTTGAATACTTCCTCCCT 269

||||||||||||||||||||||||||||||||||||||||||||||||||||||||||||

Sbjct 179 GTGCTCCTGATATAGCTTTCCCTCGAATAAATAATATAAGTTTTTGAATACTTCCTCCCT 238

Query 270 CTTTAACTCTTTTAATTTCTAGATCTATAGTAGAAAATGGAGCAGGAACAGGTTGAACTG 329

||||||||||||||||||||||||||||||||||||||||||||||||||||||||||||

Sbjct 239 CTTTAACTCTTTTAATTTCTAGATCTATAGTAGAAAATGGAGCAGGAACAGGTTGAACTG 298

Query 330 TATACCCTCCTCTTTCTTCTGGAACTGCTCATGCAGGAGCTTCTGTAGATTTAGCTATTT 389

||||||||||||||||||||||||||||||||||||||||||||||||||||||||||||

Sbjct 299 TATACCCTCCTCTTTCTTCTGGAACTGCTCATGCAGGAGCTTCTGTAGATTTAGCTATTT 358

Query 390 TTTCTCTTCATTTAGCAGGAATTTCTTCTATTTTAGGAGCAGTAAATTTTATTACAACTG 449

||||||||||||||||||||||||||||||||||||||||||||||||||||||||||||

Sbjct 359 TTTCTCTTCATTTAGCAGGAATTTCTTCTATTTTAGGAGCAGTAAATTTTATTACAACTG 418

Query 450 TAATTAATATACGATCAACTGGAATTACACTTGATCGTTTACCTTTATTTGTCTGATCTG 509

|||||||||||||||||||||||||||||||||||||| |||||||||||||||||||||

Sbjct 419 TAATTAATATACGATCAACTGGAATTACACTTGATCGTATACCTTTATTTGTCTGATCTG 478

Query 510 TAATTATTACAGCTATTTTATTACTTCTTTCATTACCAGTTTTAGCAGGAGCTATTACTA 569

||||||||||||||||||||||||||||||||||||||||||||||||||||||||||||

Sbjct 479 TAATTATTACAGCTATTTTATTACTTCTTTCATTACCAGTTTTAGCAGGAGCTATTACTA 538

Query 570 TATTATTAACTGATCGAAATTTTAATACATCATTTTTTGACCCAATTGGAGGAGGTGACC 629

||||||||||||||||||||||||||||||||||||||||||||||||||||||||||||

Sbjct 539 TATTATTAACTGATCGAAATTTTAATACATCATTTTTTGACCCAATTGGAGGAGGTGACC 598

Query 630 CTATTCTTTATCAACATCTATTT 652

|||||||||||||||||||||||

Sbjct 599 CTATTCTTTATCAACATCTATTT 621

>Aedes scutellaris voucher AS107 cytochrome c oxidase subunit I (COX1) gene, partial cds; mitochondrial

Sequence ID: MW664823.1 Length: 618

Range 1: 1 to 618

Score:1125 bits(609), Expect:0.0,

Identities:617/620(99%), Gaps:3/620(0%), Strand: Plus/Plus

Query 19 TGATCGGGGA-AGTCGGAACTTCATTAAGAGTTTTAATTCGAACTGAACTTAGTCACCCT 77

|||||||||| |||||||||||||||||||||||||||||||||||||||||||||||||

Sbjct 1 TGATCGGGGATAGTCGGAACTTCATTAAGAGTTTTAATTCGAACTGAACTTAGTCACCCT 60

Query 78 GGAATATTTATCGGAAATGATCAAATTTATAATGTAATTCGTTACTAGCTCATGCATTTA 137

||||||||||||||||||||||||||||||||||||||| |||||| |||||||||||||

Sbjct 61 GGAATATTTATCGGAAATGATCAAATTTATAATGTAATT-GTTACT-GCTCATGCATTTA 118

Query 138 TTATAAttttttttATAGTAATACCTATTATAATTGGAGGATTTGGAAATTGGTTAGTCC 197

||||||||||||||||||||||||||||||||||||||||||||||||||||||||||||

Sbjct 119 TTATAATTTTTTTTATAGTAATACCTATTATAATTGGAGGATTTGGAAATTGGTTAGTCC 178

Query 198 CATTAATATTAGGTGCTCCTGATATAGCTTTCCCTCGAATAAATAATATAAGTTTTTGAA 257

||||||||||||||||||||||||||||||||||||||||||||||||||||||||||||

Sbjct 179 CATTAATATTAGGTGCTCCTGATATAGCTTTCCCTCGAATAAATAATATAAGTTTTTGAA 238

Query 258 TACTTCCTCCCTCTTTAACTCTTTTAATTTCTAGATCTATAGTAGAAAATGGAGCAGGAA 317

||||||||||||||||||||||||||||||||||||||||||||||||||||||||||||

Sbjct 239 TACTTCCTCCCTCTTTAACTCTTTTAATTTCTAGATCTATAGTAGAAAATGGAGCAGGAA 298

Query 318 CAGGTTGAACTGTATACCCTCCTCTTTCTTCTGGAACTGCTCATGCAGGAGCTTCTGTAG 377

||||||||||||||||||||||||||||||||||||||||||||||||||||||||||||

Sbjct 299 CAGGTTGAACTGTATACCCTCCTCTTTCTTCTGGAACTGCTCATGCAGGAGCTTCTGTAG 358

Query 378 ATTTAGCTATTTTTTCTCTTCATTTAGCAGGAATTTCTTCTATTTTAGGAGCAGTAAATT 437

||||||||||||||||||||||||||||||||||||||||||||||||||||||||||||

Sbjct 359 ATTTAGCTATTTTTTCTCTTCATTTAGCAGGAATTTCTTCTATTTTAGGAGCAGTAAATT 418

Query 438 TTATTACAACTGTAATTAATATACGATCAACTGGAATTACACTTGATCGTTTACCTTTAT 497

||||||||||||||||||||||||||||||||||||||||||||||||||||||||||||

Sbjct 419 TTATTACAACTGTAATTAATATACGATCAACTGGAATTACACTTGATCGTTTACCTTTAT 478

Query 498 TTGTCTGATCTGTAATTATTACAGCTATTTTATTACTTCTTTCATTACCAGTTTTAGCAG 557

||||||||||||||||||||||||||||||||||||||||||||||||||||||||||||

Sbjct 479 TTGTCTGATCTGTAATTATTACAGCTATTTTATTACTTCTTTCATTACCAGTTTTAGCAG 538

Query 558 GAGCTATTACTATATTATTAACTGATCGAAATTTTAATACATCATTTTTTGACCCAATTG 617

||||||||||||||||||||||||||||||||||||||||||||||||||||||||||||

Sbjct 539 GAGCTATTACTATATTATTAACTGATCGAAATTTTAATACATCATTTTTTGACCCAATTG 598

Query 618 GAGGAGGTGACCCTATTCTT 637

||||||||||||||||||||

Sbjct 599 GAGGAGGTGACCCTATTCTT 618

>Aedes scutellaris voucher AS072 cytochrome c oxidase subunit I (COX1) gene, partial cds; mitochondrial

Sequence ID: MW664822.1 Length: 618

Range 1: 1 to 618

Score:1125 bits(609), Expect:0.0,

Identities:617/620(99%), Gaps:3/620(0%), Strand: Plus/Plus

Query 19 TGATCGGGGA-AGTCGGAACTTCATTAAGAGTTTTAATTCGAACTGAACTTAGTCACCCT 77

|||||||||| |||||||||||||||||||||||||||||||||||||||||||||||||

Sbjct 1 TGATCGGGGATAGTCGGAACTTCATTAAGAGTTTTAATTCGAACTGAACTTAGTCACCCT 60

Query 78 GGAATATTTATCGGAAATGATCAAATTTATAATGTAATTCGTTACTAGCTCATGCATTTA 137

||||||||||||||||||||||||||||||||||||||| |||||| |||||||||||||

Sbjct 61 GGAATATTTATCGGAAATGATCAAATTTATAATGTAATT-GTTACT-GCTCATGCATTTA 118

Query 138 TTATAAttttttttATAGTAATACCTATTATAATTGGAGGATTTGGAAATTGGTTAGTCC 197

||||||||||||||||||||||||||||||||||||||||||||||||||||||||||||

Sbjct 119 TTATAATTTTTTTTATAGTAATACCTATTATAATTGGAGGATTTGGAAATTGGTTAGTCC 178

Query 198 CATTAATATTAGGTGCTCCTGATATAGCTTTCCCTCGAATAAATAATATAAGTTTTTGAA 257

||||||||||||||||||||||||||||||||||||||||||||||||||||||||||||

Sbjct 179 CATTAATATTAGGTGCTCCTGATATAGCTTTCCCTCGAATAAATAATATAAGTTTTTGAA 238

Query 258 TACTTCCTCCCTCTTTAACTCTTTTAATTTCTAGATCTATAGTAGAAAATGGAGCAGGAA 317

||||||||||||||||||||||||||||||||||||||||||||||||||||||||||||

Sbjct 239 TACTTCCTCCCTCTTTAACTCTTTTAATTTCTAGATCTATAGTAGAAAATGGAGCAGGAA 298

Query 318 CAGGTTGAACTGTATACCCTCCTCTTTCTTCTGGAACTGCTCATGCAGGAGCTTCTGTAG 377

||||||||||||||||||||||||||||||||||||||||||||||||||||||||||||

Sbjct 299 CAGGTTGAACTGTATACCCTCCTCTTTCTTCTGGAACTGCTCATGCAGGAGCTTCTGTAG 358

Query 378 ATTTAGCTATTTTTTCTCTTCATTTAGCAGGAATTTCTTCTATTTTAGGAGCAGTAAATT 437

||||||||||||||||||||||||||||||||||||||||||||||||||||||||||||

Sbjct 359 ATTTAGCTATTTTTTCTCTTCATTTAGCAGGAATTTCTTCTATTTTAGGAGCAGTAAATT 418

Query 438 TTATTACAACTGTAATTAATATACGATCAACTGGAATTACACTTGATCGTTTACCTTTAT 497

||||||||||||||||||||||||||||||||||||||||||||||||||||||||||||

Sbjct 419 TTATTACAACTGTAATTAATATACGATCAACTGGAATTACACTTGATCGTTTACCTTTAT 478

Query 498 TTGTCTGATCTGTAATTATTACAGCTATTTTATTACTTCTTTCATTACCAGTTTTAGCAG 557

||||||||||||||||||||||||||||||||||||||||||||||||||||||||||||

Sbjct 479 TTGTCTGATCTGTAATTATTACAGCTATTTTATTACTTCTTTCATTACCAGTTTTAGCAG 538

Query 558 GAGCTATTACTATATTATTAACTGATCGAAATTTTAATACATCATTTTTTGACCCAATTG 617

||||||||||||||||||||||||||||||||||||||||||||||||||||||||||||

Sbjct 539 GAGCTATTACTATATTATTAACTGATCGAAATTTTAATACATCATTTTTTGACCCAATTG 598

Query 618 GAGGAGGTGACCCTATTCTT 637

||||||||||||||||||||

Sbjct 599 GAGGAGGTGACCCTATTCTT 618

>Aedes scutellaris voucher AS105 cytochrome c oxidase subunit I (COX1) gene, partial cds; mitochondrial

Sequence ID: MW664821.1 Length: 618

Range 1: 1 to 618

Score:1125 bits(609), Expect:0.0,

Identities:617/620(99%), Gaps:3/620(0%), Strand: Plus/Plus

Query 19 TGATCGGGGA-AGTCGGAACTTCATTAAGAGTTTTAATTCGAACTGAACTTAGTCACCCT 77

|||||||||| |||||||||||||||||||||||||||||||||||||||||||||||||

Sbjct 1 TGATCGGGGATAGTCGGAACTTCATTAAGAGTTTTAATTCGAACTGAACTTAGTCACCCT 60

Query 78 GGAATATTTATCGGAAATGATCAAATTTATAATGTAATTCGTTACTAGCTCATGCATTTA 137

||||||||||||||||||||||||||||||||||||||| |||||| |||||||||||||

Sbjct 61 GGAATATTTATCGGAAATGATCAAATTTATAATGTAATT-GTTACT-GCTCATGCATTTA 118

Query 138 TTATAAttttttttATAGTAATACCTATTATAATTGGAGGATTTGGAAATTGGTTAGTCC 197

||||||||||||||||||||||||||||||||||||||||||||||||||||||||||||

Sbjct 119 TTATAATTTTTTTTATAGTAATACCTATTATAATTGGAGGATTTGGAAATTGGTTAGTCC 178

Query 198 CATTAATATTAGGTGCTCCTGATATAGCTTTCCCTCGAATAAATAATATAAGTTTTTGAA 257

||||||||||||||||||||||||||||||||||||||||||||||||||||||||||||

Sbjct 179 CATTAATATTAGGTGCTCCTGATATAGCTTTCCCTCGAATAAATAATATAAGTTTTTGAA 238

Query 258 TACTTCCTCCCTCTTTAACTCTTTTAATTTCTAGATCTATAGTAGAAAATGGAGCAGGAA 317

||||||||||||||||||||||||||||||||||||||||||||||||||||||||||||

Sbjct 239 TACTTCCTCCCTCTTTAACTCTTTTAATTTCTAGATCTATAGTAGAAAATGGAGCAGGAA 298

Query 318 CAGGTTGAACTGTATACCCTCCTCTTTCTTCTGGAACTGCTCATGCAGGAGCTTCTGTAG 377

||||||||||||||||||||||||||||||||||||||||||||||||||||||||||||

Sbjct 299 CAGGTTGAACTGTATACCCTCCTCTTTCTTCTGGAACTGCTCATGCAGGAGCTTCTGTAG 358

Query 378 ATTTAGCTATTTTTTCTCTTCATTTAGCAGGAATTTCTTCTATTTTAGGAGCAGTAAATT 437

||||||||||||||||||||||||||||||||||||||||||||||||||||||||||||

Sbjct 359 ATTTAGCTATTTTTTCTCTTCATTTAGCAGGAATTTCTTCTATTTTAGGAGCAGTAAATT 418

Query 438 TTATTACAACTGTAATTAATATACGATCAACTGGAATTACACTTGATCGTTTACCTTTAT 497

||||||||||||||||||||||||||||||||||||||||||||||||||||||||||||

Sbjct 419 TTATTACAACTGTAATTAATATACGATCAACTGGAATTACACTTGATCGTTTACCTTTAT 478

Query 498 TTGTCTGATCTGTAATTATTACAGCTATTTTATTACTTCTTTCATTACCAGTTTTAGCAG 557

||||||||||||||||||||||||||||||||||||||||||||||||||||||||||||

Sbjct 479 TTGTCTGATCTGTAATTATTACAGCTATTTTATTACTTCTTTCATTACCAGTTTTAGCAG 538

Query 558 GAGCTATTACTATATTATTAACTGATCGAAATTTTAATACATCATTTTTTGACCCAATTG 617

||||||||||||||||||||||||||||||||||||||||||||||||||||||||||||

Sbjct 539 GAGCTATTACTATATTATTAACTGATCGAAATTTTAATACATCATTTTTTGACCCAATTG 598

Query 618 GAGGAGGTGACCCTATTCTT 637

||||||||||||||||||||

Sbjct 599 GAGGAGGTGACCCTATTCTT 618

>Aedes scutellaris voucher AS038 cytochrome c oxidase subunit I (COX1) gene, partial cds; mitochondrial

Sequence ID: MW664820.1 Length: 618

Range 1: 1 to 618

Score:1125 bits(609), Expect:0.0,

Identities:617/620(99%), Gaps:3/620(0%), Strand: Plus/Plus

Query 19 TGATCGGGGA-AGTCGGAACTTCATTAAGAGTTTTAATTCGAACTGAACTTAGTCACCCT 77

|||||||||| |||||||||||||||||||||||||||||||||||||||||||||||||

Sbjct 1 TGATCGGGGATAGTCGGAACTTCATTAAGAGTTTTAATTCGAACTGAACTTAGTCACCCT 60

Query 78 GGAATATTTATCGGAAATGATCAAATTTATAATGTAATTCGTTACTAGCTCATGCATTTA 137

||||||||||||||||||||||||||||||||||||||| |||||| |||||||||||||

Sbjct 61 GGAATATTTATCGGAAATGATCAAATTTATAATGTAATT-GTTACT-GCTCATGCATTTA 118

Query 138 TTATAAttttttttATAGTAATACCTATTATAATTGGAGGATTTGGAAATTGGTTAGTCC 197

||||||||||||||||||||||||||||||||||||||||||||||||||||||||||||

Sbjct 119 TTATAATTTTTTTTATAGTAATACCTATTATAATTGGAGGATTTGGAAATTGGTTAGTCC 178

Query 198 CATTAATATTAGGTGCTCCTGATATAGCTTTCCCTCGAATAAATAATATAAGTTTTTGAA 257

||||||||||||||||||||||||||||||||||||||||||||||||||||||||||||

Sbjct 179 CATTAATATTAGGTGCTCCTGATATAGCTTTCCCTCGAATAAATAATATAAGTTTTTGAA 238

Query 258 TACTTCCTCCCTCTTTAACTCTTTTAATTTCTAGATCTATAGTAGAAAATGGAGCAGGAA 317

||||||||||||||||||||||||||||||||||||||||||||||||||||||||||||

Sbjct 239 TACTTCCTCCCTCTTTAACTCTTTTAATTTCTAGATCTATAGTAGAAAATGGAGCAGGAA 298

Query 318 CAGGTTGAACTGTATACCCTCCTCTTTCTTCTGGAACTGCTCATGCAGGAGCTTCTGTAG 377

||||||||||||||||||||||||||||||||||||||||||||||||||||||||||||

Sbjct 299 CAGGTTGAACTGTATACCCTCCTCTTTCTTCTGGAACTGCTCATGCAGGAGCTTCTGTAG 358

Query 378 ATTTAGCTATTTTTTCTCTTCATTTAGCAGGAATTTCTTCTATTTTAGGAGCAGTAAATT 437

||||||||||||||||||||||||||||||||||||||||||||||||||||||||||||

Sbjct 359 ATTTAGCTATTTTTTCTCTTCATTTAGCAGGAATTTCTTCTATTTTAGGAGCAGTAAATT 418

Query 438 TTATTACAACTGTAATTAATATACGATCAACTGGAATTACACTTGATCGTTTACCTTTAT 497

||||||||||||||||||||||||||||||||||||||||||||||||||||||||||||

Sbjct 419 TTATTACAACTGTAATTAATATACGATCAACTGGAATTACACTTGATCGTTTACCTTTAT 478

Query 498 TTGTCTGATCTGTAATTATTACAGCTATTTTATTACTTCTTTCATTACCAGTTTTAGCAG 557

||||||||||||||||||||||||||||||||||||||||||||||||||||||||||||

Sbjct 479 TTGTCTGATCTGTAATTATTACAGCTATTTTATTACTTCTTTCATTACCAGTTTTAGCAG 538

Query 558 GAGCTATTACTATATTATTAACTGATCGAAATTTTAATACATCATTTTTTGACCCAATTG 617

||||||||||||||||||||||||||||||||||||||||||||||||||||||||||||

Sbjct 539 GAGCTATTACTATATTATTAACTGATCGAAATTTTAATACATCATTTTTTGACCCAATTG 598

Query 618 GAGGAGGTGACCCTATTCTT 637

||||||||||||||||||||

Sbjct 599 GAGGAGGTGACCCTATTCTT 618

>Aedes scutellaris voucher AS034 cytochrome c oxidase subunit I (COX1) gene, partial cds; mitochondrial

Sequence ID: MW664819.1 Length: 618

Range 1: 1 to 618

Score:1125 bits(609), Expect:0.0,

Identities:617/620(99%), Gaps:3/620(0%), Strand: Plus/Plus

Query 19 TGATCGGGGA-AGTCGGAACTTCATTAAGAGTTTTAATTCGAACTGAACTTAGTCACCCT 77

|||||||||| |||||||||||||||||||||||||||||||||||||||||||||||||

Sbjct 1 TGATCGGGGATAGTCGGAACTTCATTAAGAGTTTTAATTCGAACTGAACTTAGTCACCCT 60

Query 78 GGAATATTTATCGGAAATGATCAAATTTATAATGTAATTCGTTACTAGCTCATGCATTTA 137

||||||||||||||||||||||||||||||||||||||| |||||| |||||||||||||

Sbjct 61 GGAATATTTATCGGAAATGATCAAATTTATAATGTAATT-GTTACT-GCTCATGCATTTA 118

Query 138 TTATAAttttttttATAGTAATACCTATTATAATTGGAGGATTTGGAAATTGGTTAGTCC 197

||||||||||||||||||||||||||||||||||||||||||||||||||||||||||||

Sbjct 119 TTATAATTTTTTTTATAGTAATACCTATTATAATTGGAGGATTTGGAAATTGGTTAGTCC 178

Query 198 CATTAATATTAGGTGCTCCTGATATAGCTTTCCCTCGAATAAATAATATAAGTTTTTGAA 257

||||||||||||||||||||||||||||||||||||||||||||||||||||||||||||

Sbjct 179 CATTAATATTAGGTGCTCCTGATATAGCTTTCCCTCGAATAAATAATATAAGTTTTTGAA 238

Query 258 TACTTCCTCCCTCTTTAACTCTTTTAATTTCTAGATCTATAGTAGAAAATGGAGCAGGAA 317

||||||||||||||||||||||||||||||||||||||||||||||||||||||||||||

Sbjct 239 TACTTCCTCCCTCTTTAACTCTTTTAATTTCTAGATCTATAGTAGAAAATGGAGCAGGAA 298

Query 318 CAGGTTGAACTGTATACCCTCCTCTTTCTTCTGGAACTGCTCATGCAGGAGCTTCTGTAG 377

||||||||||||||||||||||||||||||||||||||||||||||||||||||||||||

Sbjct 299 CAGGTTGAACTGTATACCCTCCTCTTTCTTCTGGAACTGCTCATGCAGGAGCTTCTGTAG 358

Query 378 ATTTAGCTATTTTTTCTCTTCATTTAGCAGGAATTTCTTCTATTTTAGGAGCAGTAAATT 437

||||||||||||||||||||||||||||||||||||||||||||||||||||||||||||

Sbjct 359 ATTTAGCTATTTTTTCTCTTCATTTAGCAGGAATTTCTTCTATTTTAGGAGCAGTAAATT 418

Query 438 TTATTACAACTGTAATTAATATACGATCAACTGGAATTACACTTGATCGTTTACCTTTAT 497

||||||||||||||||||||||||||||||||||||||||||||||||||||||||||||

Sbjct 419 TTATTACAACTGTAATTAATATACGATCAACTGGAATTACACTTGATCGTTTACCTTTAT 478

Query 498 TTGTCTGATCTGTAATTATTACAGCTATTTTATTACTTCTTTCATTACCAGTTTTAGCAG 557

||||||||||||||||||||||||||||||||||||||||||||||||||||||||||||

Sbjct 479 TTGTCTGATCTGTAATTATTACAGCTATTTTATTACTTCTTTCATTACCAGTTTTAGCAG 538

Query 558 GAGCTATTACTATATTATTAACTGATCGAAATTTTAATACATCATTTTTTGACCCAATTG 617

||||||||||||||||||||||||||||||||||||||||||||||||||||||||||||

Sbjct 539 GAGCTATTACTATATTATTAACTGATCGAAATTTTAATACATCATTTTTTGACCCAATTG 598

Query 618 GAGGAGGTGACCCTATTCTT 637

||||||||||||||||||||

Sbjct 599 GAGGAGGTGACCCTATTCTT 618

>Aedes scutellaris voucher AS032 cytochrome c oxidase subunit I (COX1) gene, partial cds; mitochondrial

Sequence ID: MW664818.1 Length: 618

Range 1: 1 to 618

Score:1125 bits(609), Expect:0.0,

Identities:617/620(99%), Gaps:3/620(0%), Strand: Plus/Plus

Query 19 TGATCGGGGA-AGTCGGAACTTCATTAAGAGTTTTAATTCGAACTGAACTTAGTCACCCT 77

|||||||||| |||||||||||||||||||||||||||||||||||||||||||||||||

Sbjct 1 TGATCGGGGATAGTCGGAACTTCATTAAGAGTTTTAATTCGAACTGAACTTAGTCACCCT 60

Query 78 GGAATATTTATCGGAAATGATCAAATTTATAATGTAATTCGTTACTAGCTCATGCATTTA 137

||||||||||||||||||||||||||||||||||||||| |||||| |||||||||||||

Sbjct 61 GGAATATTTATCGGAAATGATCAAATTTATAATGTAATT-GTTACT-GCTCATGCATTTA 118

Query 138 TTATAAttttttttATAGTAATACCTATTATAATTGGAGGATTTGGAAATTGGTTAGTCC 197

||||||||||||||||||||||||||||||||||||||||||||||||||||||||||||

Sbjct 119 TTATAATTTTTTTTATAGTAATACCTATTATAATTGGAGGATTTGGAAATTGGTTAGTCC 178

Query 198 CATTAATATTAGGTGCTCCTGATATAGCTTTCCCTCGAATAAATAATATAAGTTTTTGAA 257

||||||||||||||||||||||||||||||||||||||||||||||||||||||||||||

Sbjct 179 CATTAATATTAGGTGCTCCTGATATAGCTTTCCCTCGAATAAATAATATAAGTTTTTGAA 238

Query 258 TACTTCCTCCCTCTTTAACTCTTTTAATTTCTAGATCTATAGTAGAAAATGGAGCAGGAA 317

||||||||||||||||||||||||||||||||||||||||||||||||||||||||||||

Sbjct 239 TACTTCCTCCCTCTTTAACTCTTTTAATTTCTAGATCTATAGTAGAAAATGGAGCAGGAA 298

Query 318 CAGGTTGAACTGTATACCCTCCTCTTTCTTCTGGAACTGCTCATGCAGGAGCTTCTGTAG 377

||||||||||||||||||||||||||||||||||||||||||||||||||||||||||||

Sbjct 299 CAGGTTGAACTGTATACCCTCCTCTTTCTTCTGGAACTGCTCATGCAGGAGCTTCTGTAG 358

Query 378 ATTTAGCTATTTTTTCTCTTCATTTAGCAGGAATTTCTTCTATTTTAGGAGCAGTAAATT 437

||||||||||||||||||||||||||||||||||||||||||||||||||||||||||||

Sbjct 359 ATTTAGCTATTTTTTCTCTTCATTTAGCAGGAATTTCTTCTATTTTAGGAGCAGTAAATT 418

Query 438 TTATTACAACTGTAATTAATATACGATCAACTGGAATTACACTTGATCGTTTACCTTTAT 497

||||||||||||||||||||||||||||||||||||||||||||||||||||||||||||

Sbjct 419 TTATTACAACTGTAATTAATATACGATCAACTGGAATTACACTTGATCGTTTACCTTTAT 478

Query 498 TTGTCTGATCTGTAATTATTACAGCTATTTTATTACTTCTTTCATTACCAGTTTTAGCAG 557

||||||||||||||||||||||||||||||||||||||||||||||||||||||||||||

Sbjct 479 TTGTCTGATCTGTAATTATTACAGCTATTTTATTACTTCTTTCATTACCAGTTTTAGCAG 538

Query 558 GAGCTATTACTATATTATTAACTGATCGAAATTTTAATACATCATTTTTTGACCCAATTG 617

||||||||||||||||||||||||||||||||||||||||||||||||||||||||||||

Sbjct 539 GAGCTATTACTATATTATTAACTGATCGAAATTTTAATACATCATTTTTTGACCCAATTG 598

Query 618 GAGGAGGTGACCCTATTCTT 637

||||||||||||||||||||

Sbjct 599 GAGGAGGTGACCCTATTCTT 618

>Aedes scutellaris voucher AS112 cytochrome c oxidase subunit I (COX1) gene, partial cds; mitochondrial

Sequence ID: MW664817.1 Length: 618

Range 1: 1 to 618

Score:1125 bits(609), Expect:0.0,

Identities:617/620(99%), Gaps:3/620(0%), Strand: Plus/Plus

Query 19 TGATCGGGGA-AGTCGGAACTTCATTAAGAGTTTTAATTCGAACTGAACTTAGTCACCCT 77

|||||||||| |||||||||||||||||||||||||||||||||||||||||||||||||

Sbjct 1 TGATCGGGGATAGTCGGAACTTCATTAAGAGTTTTAATTCGAACTGAACTTAGTCACCCT 60

Query 78 GGAATATTTATCGGAAATGATCAAATTTATAATGTAATTCGTTACTAGCTCATGCATTTA 137

||||||||||||||||||||||||||||||||||||||| |||||| |||||||||||||

Sbjct 61 GGAATATTTATCGGAAATGATCAAATTTATAATGTAATT-GTTACT-GCTCATGCATTTA 118

Query 138 TTATAAttttttttATAGTAATACCTATTATAATTGGAGGATTTGGAAATTGGTTAGTCC 197

||||||||||||||||||||||||||||||||||||||||||||||||||||||||||||

Sbjct 119 TTATAATTTTTTTTATAGTAATACCTATTATAATTGGAGGATTTGGAAATTGGTTAGTCC 178

Query 198 CATTAATATTAGGTGCTCCTGATATAGCTTTCCCTCGAATAAATAATATAAGTTTTTGAA 257

||||||||||||||||||||||||||||||||||||||||||||||||||||||||||||

Sbjct 179 CATTAATATTAGGTGCTCCTGATATAGCTTTCCCTCGAATAAATAATATAAGTTTTTGAA 238

Query 258 TACTTCCTCCCTCTTTAACTCTTTTAATTTCTAGATCTATAGTAGAAAATGGAGCAGGAA 317

||||||||||||||||||||||||||||||||||||||||||||||||||||||||||||

Sbjct 239 TACTTCCTCCCTCTTTAACTCTTTTAATTTCTAGATCTATAGTAGAAAATGGAGCAGGAA 298

Query 318 CAGGTTGAACTGTATACCCTCCTCTTTCTTCTGGAACTGCTCATGCAGGAGCTTCTGTAG 377

||||||||||||||||||||||||||||||||||||||||||||||||||||||||||||

Sbjct 299 CAGGTTGAACTGTATACCCTCCTCTTTCTTCTGGAACTGCTCATGCAGGAGCTTCTGTAG 358

Query 378 ATTTAGCTATTTTTTCTCTTCATTTAGCAGGAATTTCTTCTATTTTAGGAGCAGTAAATT 437

||||||||||||||||||||||||||||||||||||||||||||||||||||||||||||

Sbjct 359 ATTTAGCTATTTTTTCTCTTCATTTAGCAGGAATTTCTTCTATTTTAGGAGCAGTAAATT 418

Query 438 TTATTACAACTGTAATTAATATACGATCAACTGGAATTACACTTGATCGTTTACCTTTAT 497

||||||||||||||||||||||||||||||||||||||||||||||||||||||||||||

Sbjct 419 TTATTACAACTGTAATTAATATACGATCAACTGGAATTACACTTGATCGTTTACCTTTAT 478

Query 498 TTGTCTGATCTGTAATTATTACAGCTATTTTATTACTTCTTTCATTACCAGTTTTAGCAG 557

||||||||||||||||||||||||||||||||||||||||||||||||||||||||||||

Sbjct 479 TTGTCTGATCTGTAATTATTACAGCTATTTTATTACTTCTTTCATTACCAGTTTTAGCAG 538

Query 558 GAGCTATTACTATATTATTAACTGATCGAAATTTTAATACATCATTTTTTGACCCAATTG 617

||||||||||||||||||||||||||||||||||||||||||||||||||||||||||||

Sbjct 539 GAGCTATTACTATATTATTAACTGATCGAAATTTTAATACATCATTTTTTGACCCAATTG 598

Query 618 GAGGAGGTGACCCTATTCTT 637

||||||||||||||||||||

Sbjct 599 GAGGAGGTGACCCTATTCTT 618

>Aedes scutellaris voucher AS111 cytochrome c oxidase subunit I (COX1) gene, partial cds; mitochondrial

Sequence ID: MW664816.1 Length: 618

Range 1: 1 to 618

Score:1125 bits(609), Expect:0.0,

Identities:617/620(99%), Gaps:3/620(0%), Strand: Plus/Plus

Query 19 TGATCGGGGA-AGTCGGAACTTCATTAAGAGTTTTAATTCGAACTGAACTTAGTCACCCT 77

|||||||||| |||||||||||||||||||||||||||||||||||||||||||||||||

Sbjct 1 TGATCGGGGATAGTCGGAACTTCATTAAGAGTTTTAATTCGAACTGAACTTAGTCACCCT 60

Query 78 GGAATATTTATCGGAAATGATCAAATTTATAATGTAATTCGTTACTAGCTCATGCATTTA 137

||||||||||||||||||||||||||||||||||||||| |||||| |||||||||||||

Sbjct 61 GGAATATTTATCGGAAATGATCAAATTTATAATGTAATT-GTTACT-GCTCATGCATTTA 118

Query 138 TTATAAttttttttATAGTAATACCTATTATAATTGGAGGATTTGGAAATTGGTTAGTCC 197

||||||||||||||||||||||||||||||||||||||||||||||||||||||||||||

Sbjct 119 TTATAATTTTTTTTATAGTAATACCTATTATAATTGGAGGATTTGGAAATTGGTTAGTCC 178

Query 198 CATTAATATTAGGTGCTCCTGATATAGCTTTCCCTCGAATAAATAATATAAGTTTTTGAA 257

||||||||||||||||||||||||||||||||||||||||||||||||||||||||||||

Sbjct 179 CATTAATATTAGGTGCTCCTGATATAGCTTTCCCTCGAATAAATAATATAAGTTTTTGAA 238

Query 258 TACTTCCTCCCTCTTTAACTCTTTTAATTTCTAGATCTATAGTAGAAAATGGAGCAGGAA 317

||||||||||||||||||||||||||||||||||||||||||||||||||||||||||||

Sbjct 239 TACTTCCTCCCTCTTTAACTCTTTTAATTTCTAGATCTATAGTAGAAAATGGAGCAGGAA 298

Query 318 CAGGTTGAACTGTATACCCTCCTCTTTCTTCTGGAACTGCTCATGCAGGAGCTTCTGTAG 377

||||||||||||||||||||||||||||||||||||||||||||||||||||||||||||

Sbjct 299 CAGGTTGAACTGTATACCCTCCTCTTTCTTCTGGAACTGCTCATGCAGGAGCTTCTGTAG 358

Query 378 ATTTAGCTATTTTTTCTCTTCATTTAGCAGGAATTTCTTCTATTTTAGGAGCAGTAAATT 437

||||||||||||||||||||||||||||||||||||||||||||||||||||||||||||

Sbjct 359 ATTTAGCTATTTTTTCTCTTCATTTAGCAGGAATTTCTTCTATTTTAGGAGCAGTAAATT 418

Query 438 TTATTACAACTGTAATTAATATACGATCAACTGGAATTACACTTGATCGTTTACCTTTAT 497

||||||||||||||||||||||||||||||||||||||||||||||||||||||||||||

Sbjct 419 TTATTACAACTGTAATTAATATACGATCAACTGGAATTACACTTGATCGTTTACCTTTAT 478

Query 498 TTGTCTGATCTGTAATTATTACAGCTATTTTATTACTTCTTTCATTACCAGTTTTAGCAG 557

||||||||||||||||||||||||||||||||||||||||||||||||||||||||||||

Sbjct 479 TTGTCTGATCTGTAATTATTACAGCTATTTTATTACTTCTTTCATTACCAGTTTTAGCAG 538

Query 558 GAGCTATTACTATATTATTAACTGATCGAAATTTTAATACATCATTTTTTGACCCAATTG 617

||||||||||||||||||||||||||||||||||||||||||||||||||||||||||||

Sbjct 539 GAGCTATTACTATATTATTAACTGATCGAAATTTTAATACATCATTTTTTGACCCAATTG 598

Query 618 GAGGAGGTGACCCTATTCTT 637

||||||||||||||||||||

Sbjct 599 GAGGAGGTGACCCTATTCTT 618

>Aedes scutellaris voucher AS110 cytochrome c oxidase subunit I (COX1) gene, partial cds; mitochondrial

Sequence ID: MW664815.1 Length: 618

Range 1: 1 to 618

Score:1125 bits(609), Expect:0.0,

Identities:617/620(99%), Gaps:3/620(0%), Strand: Plus/Plus

Query 19 TGATCGGGGA-AGTCGGAACTTCATTAAGAGTTTTAATTCGAACTGAACTTAGTCACCCT 77

|||||||||| |||||||||||||||||||||||||||||||||||||||||||||||||

Sbjct 1 TGATCGGGGATAGTCGGAACTTCATTAAGAGTTTTAATTCGAACTGAACTTAGTCACCCT 60

Query 78 GGAATATTTATCGGAAATGATCAAATTTATAATGTAATTCGTTACTAGCTCATGCATTTA 137

||||||||||||||||||||||||||||||||||||||| |||||| |||||||||||||

Sbjct 61 GGAATATTTATCGGAAATGATCAAATTTATAATGTAATT-GTTACT-GCTCATGCATTTA 118

Query 138 TTATAAttttttttATAGTAATACCTATTATAATTGGAGGATTTGGAAATTGGTTAGTCC 197

||||||||||||||||||||||||||||||||||||||||||||||||||||||||||||

Sbjct 119 TTATAATTTTTTTTATAGTAATACCTATTATAATTGGAGGATTTGGAAATTGGTTAGTCC 178

Query 198 CATTAATATTAGGTGCTCCTGATATAGCTTTCCCTCGAATAAATAATATAAGTTTTTGAA 257

||||||||||||||||||||||||||||||||||||||||||||||||||||||||||||

Sbjct 179 CATTAATATTAGGTGCTCCTGATATAGCTTTCCCTCGAATAAATAATATAAGTTTTTGAA 238

Query 258 TACTTCCTCCCTCTTTAACTCTTTTAATTTCTAGATCTATAGTAGAAAATGGAGCAGGAA 317

||||||||||||||||||||||||||||||||||||||||||||||||||||||||||||

Sbjct 239 TACTTCCTCCCTCTTTAACTCTTTTAATTTCTAGATCTATAGTAGAAAATGGAGCAGGAA 298

Query 318 CAGGTTGAACTGTATACCCTCCTCTTTCTTCTGGAACTGCTCATGCAGGAGCTTCTGTAG 377

||||||||||||||||||||||||||||||||||||||||||||||||||||||||||||

Sbjct 299 CAGGTTGAACTGTATACCCTCCTCTTTCTTCTGGAACTGCTCATGCAGGAGCTTCTGTAG 358

Query 378 ATTTAGCTATTTTTTCTCTTCATTTAGCAGGAATTTCTTCTATTTTAGGAGCAGTAAATT 437

||||||||||||||||||||||||||||||||||||||||||||||||||||||||||||

Sbjct 359 ATTTAGCTATTTTTTCTCTTCATTTAGCAGGAATTTCTTCTATTTTAGGAGCAGTAAATT 418

Query 438 TTATTACAACTGTAATTAATATACGATCAACTGGAATTACACTTGATCGTTTACCTTTAT 497

||||||||||||||||||||||||||||||||||||||||||||||||||||||||||||

Sbjct 419 TTATTACAACTGTAATTAATATACGATCAACTGGAATTACACTTGATCGTTTACCTTTAT 478

Query 498 TTGTCTGATCTGTAATTATTACAGCTATTTTATTACTTCTTTCATTACCAGTTTTAGCAG 557

||||||||||||||||||||||||||||||||||||||||||||||||||||||||||||

Sbjct 479 TTGTCTGATCTGTAATTATTACAGCTATTTTATTACTTCTTTCATTACCAGTTTTAGCAG 538

Query 558 GAGCTATTACTATATTATTAACTGATCGAAATTTTAATACATCATTTTTTGACCCAATTG 617

||||||||||||||||||||||||||||||||||||||||||||||||||||||||||||

Sbjct 539 GAGCTATTACTATATTATTAACTGATCGAAATTTTAATACATCATTTTTTGACCCAATTG 598

Query 618 GAGGAGGTGACCCTATTCTT 637

||||||||||||||||||||

Sbjct 599 GAGGAGGTGACCCTATTCTT 618

>Aedes scutellaris voucher AS029 cytochrome c oxidase subunit I (COX1) gene, partial cds; mitochondrial

Sequence ID: MW664814.1 Length: 618

Range 1: 1 to 618

Score:1125 bits(609), Expect:0.0,

Identities:617/620(99%), Gaps:3/620(0%), Strand: Plus/Plus

Query 19 TGATCGGGGA-AGTCGGAACTTCATTAAGAGTTTTAATTCGAACTGAACTTAGTCACCCT 77

|||||||||| |||||||||||||||||||||||||||||||||||||||||||||||||

Sbjct 1 TGATCGGGGATAGTCGGAACTTCATTAAGAGTTTTAATTCGAACTGAACTTAGTCACCCT 60

Query 78 GGAATATTTATCGGAAATGATCAAATTTATAATGTAATTCGTTACTAGCTCATGCATTTA 137

||||||||||||||||||||||||||||||||||||||| |||||| |||||||||||||

Sbjct 61 GGAATATTTATCGGAAATGATCAAATTTATAATGTAATT-GTTACT-GCTCATGCATTTA 118

Query 138 TTATAAttttttttATAGTAATACCTATTATAATTGGAGGATTTGGAAATTGGTTAGTCC 197

||||||||||||||||||||||||||||||||||||||||||||||||||||||||||||

Sbjct 119 TTATAATTTTTTTTATAGTAATACCTATTATAATTGGAGGATTTGGAAATTGGTTAGTCC 178

Query 198 CATTAATATTAGGTGCTCCTGATATAGCTTTCCCTCGAATAAATAATATAAGTTTTTGAA 257

||||||||||||||||||||||||||||||||||||||||||||||||||||||||||||

Sbjct 179 CATTAATATTAGGTGCTCCTGATATAGCTTTCCCTCGAATAAATAATATAAGTTTTTGAA 238

Query 258 TACTTCCTCCCTCTTTAACTCTTTTAATTTCTAGATCTATAGTAGAAAATGGAGCAGGAA 317

||||||||||||||||||||||||||||||||||||||||||||||||||||||||||||

Sbjct 239 TACTTCCTCCCTCTTTAACTCTTTTAATTTCTAGATCTATAGTAGAAAATGGAGCAGGAA 298

Query 318 CAGGTTGAACTGTATACCCTCCTCTTTCTTCTGGAACTGCTCATGCAGGAGCTTCTGTAG 377

||||||||||||||||||||||||||||||||||||||||||||||||||||||||||||

Sbjct 299 CAGGTTGAACTGTATACCCTCCTCTTTCTTCTGGAACTGCTCATGCAGGAGCTTCTGTAG 358

Query 378 ATTTAGCTATTTTTTCTCTTCATTTAGCAGGAATTTCTTCTATTTTAGGAGCAGTAAATT 437

||||||||||||||||||||||||||||||||||||||||||||||||||||||||||||

Sbjct 359 ATTTAGCTATTTTTTCTCTTCATTTAGCAGGAATTTCTTCTATTTTAGGAGCAGTAAATT 418

Query 438 TTATTACAACTGTAATTAATATACGATCAACTGGAATTACACTTGATCGTTTACCTTTAT 497

||||||||||||||||||||||||||||||||||||||||||||||||||||||||||||

Sbjct 419 TTATTACAACTGTAATTAATATACGATCAACTGGAATTACACTTGATCGTTTACCTTTAT 478

Query 498 TTGTCTGATCTGTAATTATTACAGCTATTTTATTACTTCTTTCATTACCAGTTTTAGCAG 557

||||||||||||||||||||||||||||||||||||||||||||||||||||||||||||

Sbjct 479 TTGTCTGATCTGTAATTATTACAGCTATTTTATTACTTCTTTCATTACCAGTTTTAGCAG 538

Query 558 GAGCTATTACTATATTATTAACTGATCGAAATTTTAATACATCATTTTTTGACCCAATTG 617

||||||||||||||||||||||||||||||||||||||||||||||||||||||||||||

Sbjct 539 GAGCTATTACTATATTATTAACTGATCGAAATTTTAATACATCATTTTTTGACCCAATTG 598

Query 618 GAGGAGGTGACCCTATTCTT 637

||||||||||||||||||||

Sbjct 599 GAGGAGGTGACCCTATTCTT 618

>Aedes malayensis voucher LA081 cytochrome c oxidase subunit I (COX1) gene, partial cds; mitochondrial

Sequence ID: MW664763.1 Length: 618

Range 1: 1 to 618

Score:1092 bits(591), Expect:0.0,

Identities:611/620(99%), Gaps:3/620(0%), Strand: Plus/Plus

Query 19 TGATCGGGGA-AGTCGGAACTTCATTAAGAGTTTTAATTCGAACTGAACTTAGTCACCCT 77

||||| || | |||||||||||||||||||||||||||||||||||||||||||||||||

Sbjct 1 TGATCTGGAATAGTCGGAACTTCATTAAGAGTTTTAATTCGAACTGAACTTAGTCACCCT 60

Query 78 GGAATATTTATCGGAAATGATCAAATTTATAATGTAATTCGTTACTAGCTCATGCATTTA 137

||||||||||||||||||||||||||||||||||||||| |||||| |||||||||||||

Sbjct 61 GGAATATTTATCGGAAATGATCAAATTTATAATGTAATT-GTTACT-GCTCATGCATTTA 118

Query 138 TTATAAttttttttATAGTAATACCTATTATAATTGGAGGATTTGGAAATTGGTTAGTCC 197

|||||||||||||||||||||||||||||||||||||||||||||||||||| |||||||

Sbjct 119 TTATAATTTTTTTTATAGTAATACCTATTATAATTGGAGGATTTGGAAATTGATTAGTCC 178

Query 198 CATTAATATTAGGTGCTCCTGATATAGCTTTCCCTCGAATAAATAATATAAGTTTTTGAA 257

||||||||||||||||||||||||||||||||||||||||||||||||||||||||||||

Sbjct 179 CATTAATATTAGGTGCTCCTGATATAGCTTTCCCTCGAATAAATAATATAAGTTTTTGAA 238

Query 258 TACTTCCTCCCTCTTTAACTCTTTTAATTTCTAGATCTATAGTAGAAAATGGAGCAGGAA 317

||||||||||||||||||| |||||| |||||||||||||||||||||||||||||||||

Sbjct 239 TACTTCCTCCCTCTTTAACCCTTTTACTTTCTAGATCTATAGTAGAAAATGGAGCAGGAA 298

Query 318 CAGGTTGAACTGTATACCCTCCTCTTTCTTCTGGAACTGCTCATGCAGGAGCTTCTGTAG 377

||||||||||||||||||||||||||||||||||||||||||||||||||||||||||||

Sbjct 299 CAGGTTGAACTGTATACCCTCCTCTTTCTTCTGGAACTGCTCATGCAGGAGCTTCTGTAG 358

Query 378 ATTTAGCTATTTTTTCTCTTCATTTAGCAGGAATTTCTTCTATTTTAGGAGCAGTAAATT 437

||||||||||||||||||||||||||||||||||||||||||||||||||||||||||||

Sbjct 359 ATTTAGCTATTTTTTCTCTTCATTTAGCAGGAATTTCTTCTATTTTAGGAGCAGTAAATT 418

Query 438 TTATTACAACTGTAATTAATATACGATCAACTGGAATTACACTTGATCGTTTACCTTTAT 497

||||||||||||||||||||||||||||||||||||||||||||||||||||||||||||

Sbjct 419 TTATTACAACTGTAATTAATATACGATCAACTGGAATTACACTTGATCGTTTACCTTTAT 478

Query 498 TTGTCTGATCTGTAATTATTACAGCTATTTTATTACTTCTTTCATTACCAGTTTTAGCAG 557

|||||||||| |||||||||||||||||||||||||||||||||||||||||||||||||

Sbjct 479 TTGTCTGATCAGTAATTATTACAGCTATTTTATTACTTCTTTCATTACCAGTTTTAGCAG 538

Query 558 GAGCTATTACTATATTATTAACTGATCGAAATTTTAATACATCATTTTTTGACCCAATTG 617

||||||||||||||||||||||||||||||||||||||||||||||||||||||||||||

Sbjct 539 GAGCTATTACTATATTATTAACTGATCGAAATTTTAATACATCATTTTTTGACCCAATTG 598

Query 618 GAGGAGGTGACCCTATTCTT 637

||||||||||||||||||||

Sbjct 599 GAGGAGGTGACCCTATTCTT 618

>Aedes malayensis voucher LA080 cytochrome c oxidase subunit I (COX1) gene, partial cds; mitochondrial

Sequence ID: MW664762.1 Length: 618

Range 1: 1 to 618

Score:1092 bits(591), Expect:0.0,

Identities:611/620(99%), Gaps:3/620(0%), Strand: Plus/Plus

Query 19 TGATCGGGGA-AGTCGGAACTTCATTAAGAGTTTTAATTCGAACTGAACTTAGTCACCCT 77

||||| || | |||||||||||||||||||||||||||||||||||||||||||||||||

Sbjct 1 TGATCTGGAATAGTCGGAACTTCATTAAGAGTTTTAATTCGAACTGAACTTAGTCACCCT 60

Query 78 GGAATATTTATCGGAAATGATCAAATTTATAATGTAATTCGTTACTAGCTCATGCATTTA 137

||||||||||||||||||||||||||||||||||||||| |||||| |||||||||||||

Sbjct 61 GGAATATTTATCGGAAATGATCAAATTTATAATGTAATT-GTTACT-GCTCATGCATTTA 118

Query 138 TTATAAttttttttATAGTAATACCTATTATAATTGGAGGATTTGGAAATTGGTTAGTCC 197

|||||||||||||||||||||||||||||||||||||||||||||||||||| |||||||

Sbjct 119 TTATAATTTTTTTTATAGTAATACCTATTATAATTGGAGGATTTGGAAATTGATTAGTCC 178

Query 198 CATTAATATTAGGTGCTCCTGATATAGCTTTCCCTCGAATAAATAATATAAGTTTTTGAA 257

||||||||||||||||||||||||||||||||||||||||||||||||||||||||||||

Sbjct 179 CATTAATATTAGGTGCTCCTGATATAGCTTTCCCTCGAATAAATAATATAAGTTTTTGAA 238

Query 258 TACTTCCTCCCTCTTTAACTCTTTTAATTTCTAGATCTATAGTAGAAAATGGAGCAGGAA 317

||||||||||||||||||| |||||| |||||||||||||||||||||||||||||||||

Sbjct 239 TACTTCCTCCCTCTTTAACCCTTTTACTTTCTAGATCTATAGTAGAAAATGGAGCAGGAA 298

Query 318 CAGGTTGAACTGTATACCCTCCTCTTTCTTCTGGAACTGCTCATGCAGGAGCTTCTGTAG 377

||||||||||||||||||||||||||||||||||||||||||||||||||||||||||||

Sbjct 299 CAGGTTGAACTGTATACCCTCCTCTTTCTTCTGGAACTGCTCATGCAGGAGCTTCTGTAG 358

Query 378 ATTTAGCTATTTTTTCTCTTCATTTAGCAGGAATTTCTTCTATTTTAGGAGCAGTAAATT 437

||||||||||||||||||||||||||||||||||||||||||||||||||||||||||||

Sbjct 359 ATTTAGCTATTTTTTCTCTTCATTTAGCAGGAATTTCTTCTATTTTAGGAGCAGTAAATT 418

Query 438 TTATTACAACTGTAATTAATATACGATCAACTGGAATTACACTTGATCGTTTACCTTTAT 497

||||||||||||||||||||||||||||||||||||||||||||||||||||||||||||

Sbjct 419 TTATTACAACTGTAATTAATATACGATCAACTGGAATTACACTTGATCGTTTACCTTTAT 478

Query 498 TTGTCTGATCTGTAATTATTACAGCTATTTTATTACTTCTTTCATTACCAGTTTTAGCAG 557

|||||||||| |||||||||||||||||||||||||||||||||||||||||||||||||

Sbjct 479 TTGTCTGATCAGTAATTATTACAGCTATTTTATTACTTCTTTCATTACCAGTTTTAGCAG 538

Query 558 GAGCTATTACTATATTATTAACTGATCGAAATTTTAATACATCATTTTTTGACCCAATTG 617

||||||||||||||||||||||||||||||||||||||||||||||||||||||||||||

Sbjct 539 GAGCTATTACTATATTATTAACTGATCGAAATTTTAATACATCATTTTTTGACCCAATTG 598

Query 618 GAGGAGGTGACCCTATTCTT 637

||||||||||||||||||||

Sbjct 599 GAGGAGGTGACCCTATTCTT 618

>Aedes riversi mitochondrial COI gene for cytochrome oxidase subunit 1, partial cds, isolate: 054ISH2000

Sequence ID: AB738098.1 Length: 658

>Aedes riversi mitochondrial COI gene for cytochrome oxidase subunit 1, partial cds, isolate: 896ISH2011

Sequence ID: AB738252.1 Length: 658

Range 1: 13 to 658

Score:1055 bits(571), Expect:0.0,

Identities:623/648(96%), Gaps:3/648(0%), Strand: Plus/Plus

Query 6 TATTTTTGGAGTATGATCGGGGA-AGTCGGAACTTCATTAAGAGTTTTAATTCGAACTGA 64

|||||||||||||||||| || | ||| ||||||||||||||||||||||||||||||||

Sbjct 13 TATTTTTGGAGTATGATCTGGAATAGTTGGAACTTCATTAAGAGTTTTAATTCGAACTGA 72

Query 65 ACTTAGTCACCCTGGAATATTTATCGGAAATGATCAAATTTATAATGTAATTCGTTACTA 124

||||||||| ||||| |||||||| ||||||||||||||||||||||||||| ||||||

Sbjct 73 ACTTAGTCATCCTGGGATATTTATTGGAAATGATCAAATTTATAATGTAATT-GTTACT- 130

Query 125 GCTCATGCATTTATTATAAttttttttATAGTAATACCTATTATAATTGGAGGATTTGGA 184

||||||||||||||||||||||||||||||||||||||||||||||||||||||||||||

Sbjct 131 GCTCATGCATTTATTATAATTTTTTTTATAGTAATACCTATTATAATTGGAGGATTTGGA 190

Query 185 AATTGGTTAGTCCCATTAATATTAGGTGCTCCTGATATAGCTTTCCCTCGAATAAATAAT 244

||||| ||||||||| ||||||||||||| ||||| ||||| || |||||||||||||||

Sbjct 191 AATTGATTAGTCCCACTAATATTAGGTGCCCCTGACATAGCCTTTCCTCGAATAAATAAT 250

Query 245 ATAAGTTTTTGAATACTTCCTCCCTCTTTAACTCTTTTAATTTCTAGATCTATAGTAGAA 304

||||||||||||||||||||||| |||||||| |||||| ||||||||||||||||||||

Sbjct 251 ATAAGTTTTTGAATACTTCCTCCTTCTTTAACCCTTTTACTTTCTAGATCTATAGTAGAA 310

Query 305 AATGGAGCAGGAACAGGTTGAACTGTATACCCTCCTCTTTCTTCTGGAACTGCTCATGCA 364

||||||||||||||||||||||||||||| ||||||||||||||||||||||| ||||||

Sbjct 311 AATGGAGCAGGAACAGGTTGAACTGTATATCCTCCTCTTTCTTCTGGAACTGCCCATGCA 370

Query 365 GGAGCTTCTGTAGATTTAGCTATTTTTTCTCTTCATTTAGCAGGAATTTCTTCTATTTTA 424

||||||||||||||||||||||||||||||||||||||||||||||||||||||||||||

Sbjct 371 GGAGCTTCTGTAGATTTAGCTATTTTTTCTCTTCATTTAGCAGGAATTTCTTCTATTTTA 430

Query 425 GGAGCAGTAAATTTTATTACAACTGTAATTAATATACGATCAACTGGAATTACACTTGAT 484

|||||||||||||||||||||||||||||||||||||||||| |||||||||||||||||

Sbjct 431 GGAGCAGTAAATTTTATTACAACTGTAATTAATATACGATCAGCTGGAATTACACTTGAT 490

Query 485 CGTTTACCTTTATTTGTCTGATCTGTAATTATTACAGCTATTTTATTACTTCTTTCATTA 544

||||||||||||||||||||||| ||| ||||||||||||||||||||||||||||||||

Sbjct 491 CGTTTACCTTTATTTGTCTGATCAGTAGTTATTACAGCTATTTTATTACTTCTTTCATTA 550

Query 545 CCAGTTTTAGCAGGAGCTATTACTATATTATTAACTGATCGAAATTTTAATACATCATTT 604

||||||||||||||||||||||||||||||||||||||||||||||||||||||||||||

Sbjct 551 CCAGTTTTAGCAGGAGCTATTACTATATTATTAACTGATCGAAATTTTAATACATCATTT 610

Query 605 TTTGACCCAATTGGAGGAGGTGACCCTATTCTTTATCAACATCTATTT 652

||||| || |||||||||||||||||||||||||||||||||||||||

Sbjct 611 TTTGATCCTATTGGAGGAGGTGACCCTATTCTTTATCAACATCTATTT 658

>Aedes riversi mitochondrial COI gene for cytochrome oxidase subunit 1, partial cds, isolate: 910AMA2011

Sequence ID: AB738253.1 Length: 658

Range 1: 13 to 658

Score:1050 bits(568), Expect:0.0,

Identities:622/648(96%), Gaps:3/648(0%), Strand: Plus/Plus

Query 6 TATTTTTGGAGTATGATCGGGGA-AGTCGGAACTTCATTAAGAGTTTTAATTCGAACTGA 64

|||||||||||||||||| || | ||| ||||||||||||||||||||||||||||||||

Sbjct 13 TATTTTTGGAGTATGATCTGGAATAGTTGGAACTTCATTAAGAGTTTTAATTCGAACTGA 72

Query 65 ACTTAGTCACCCTGGAATATTTATCGGAAATGATCAAATTTATAATGTAATTCGTTACTA 124

||||||||| ||||| |||||||| |||||||| |||||||||||||||||| ||||||

Sbjct 73 ACTTAGTCATCCTGGGATATTTATTGGAAATGACCAAATTTATAATGTAATT-GTTACT- 130

Query 125 GCTCATGCATTTATTATAAttttttttATAGTAATACCTATTATAATTGGAGGATTTGGA 184

||||||||||||||||||||||||||||||||||||||||||||||||||||||||||||

Sbjct 131 GCTCATGCATTTATTATAATTTTTTTTATAGTAATACCTATTATAATTGGAGGATTTGGA 190

Query 185 AATTGGTTAGTCCCATTAATATTAGGTGCTCCTGATATAGCTTTCCCTCGAATAAATAAT 244

||||| ||||||||| ||||||||||||| ||||| ||||| || |||||||||||||||

Sbjct 191 AATTGATTAGTCCCACTAATATTAGGTGCCCCTGACATAGCCTTTCCTCGAATAAATAAT 250

Query 245 ATAAGTTTTTGAATACTTCCTCCCTCTTTAACTCTTTTAATTTCTAGATCTATAGTAGAA 304

|||||||||||||| |||||||| |||||||| |||||| ||||||||||||||||||||

Sbjct 251 ATAAGTTTTTGAATGCTTCCTCCTTCTTTAACCCTTTTACTTTCTAGATCTATAGTAGAA 310

Query 305 AATGGAGCAGGAACAGGTTGAACTGTATACCCTCCTCTTTCTTCTGGAACTGCTCATGCA 364

||||||||||||||||||||||||||||||||||||||||||||||||||||||||||||

Sbjct 311 AATGGAGCAGGAACAGGTTGAACTGTATACCCTCCTCTTTCTTCTGGAACTGCTCATGCA 370

Query 365 GGAGCTTCTGTAGATTTAGCTATTTTTTCTCTTCATTTAGCAGGAATTTCTTCTATTTTA 424

||||||||||||||||||||||||||||||||||||||||||||||||||||||||||||

Sbjct 371 GGAGCTTCTGTAGATTTAGCTATTTTTTCTCTTCATTTAGCAGGAATTTCTTCTATTTTA 430

Query 425 GGAGCAGTAAATTTTATTACAACTGTAATTAATATACGATCAACTGGAATTACACTTGAT 484

|||||||||||||||||||||||||||||||||||||||||| |||||||||||||||||

Sbjct 431 GGAGCAGTAAATTTTATTACAACTGTAATTAATATACGATCAGCTGGAATTACACTTGAT 490

Query 485 CGTTTACCTTTATTTGTCTGATCTGTAATTATTACAGCTATTTTATTACTTCTTTCATTA 544

||||||||||||||||| ||||| ||| ||||||||||||||||||||||||||||||||

Sbjct 491 CGTTTACCTTTATTTGTTTGATCAGTAGTTATTACAGCTATTTTATTACTTCTTTCATTA 550

Query 545 CCAGTTTTAGCAGGAGCTATTACTATATTATTAACTGATCGAAATTTTAATACATCATTT 604

||||||||||||||||||||||||||||||||||||||||||||||||||||||||||||

Sbjct 551 CCAGTTTTAGCAGGAGCTATTACTATATTATTAACTGATCGAAATTTTAATACATCATTT 610

Query 605 TTTGACCCAATTGGAGGAGGTGACCCTATTCTTTATCAACATCTATTT 652

||||| || |||||||||||||||||||||||||||||||||||||||

Sbjct 611 TTTGATCCTATTGGAGGAGGTGACCCTATTCTTTATCAACATCTATTT 658

>Aedes riversi mitochondrial COI gene for cytochrome oxidase subunit 1, partial cds, isolate: L2233

Sequence ID: LC054394.1 Length: 650

Range 1: 8 to 650

Score:1044 bits(565), Expect:0.0,

Identities:619/645(96%), Gaps:3/645(0%), Strand: Plus/Plus

Query 6 TATTTTTGGAGTATGATCGGGGA-AGTCGGAACTTCATTAAGAGTTTTAATTCGAACTGA 64

|||||||||||||||||| || | |||||||||||||||||||| |||||||||||||||

Sbjct 8 TATTTTTGGAGTATGATCTGGAATAGTCGGAACTTCATTAAGAGCTTTAATTCGAACTGA 67

Query 65 ACTTAGTCACCCTGGAATATTTATCGGAAATGATCAAATTTATAATGTAATTCGTTACTA 124

||||||||| |||||||||||||| |||||||| |||||||||||||||||| || |||

Sbjct 68 ACTTAGTCATCCTGGAATATTTATTGGAAATGACCAAATTTATAATGTAATT-GTGACT- 125

Query 125 GCTCATGCATTTATTATAAttttttttATAGTAATACCTATTATAATTGGAGGATTTGGA 184

||||||||||||||||||||||||||||||||||||||||||||||||||||||||||||

Sbjct 126 GCTCATGCATTTATTATAATTTTTTTTATAGTAATACCTATTATAATTGGAGGATTTGGA 185

Query 185 AATTGGTTAGTCCCATTAATATTAGGTGCTCCTGATATAGCTTTCCCTCGAATAAATAAT 244

||||| ||||||||| ||||||||||||| ||||| ||||| || |||||||||||||||

Sbjct 186 AATTGATTAGTCCCACTAATATTAGGTGCCCCTGACATAGCCTTTCCTCGAATAAATAAT 245

Query 245 ATAAGTTTTTGAATACTTCCTCCCTCTTTAACTCTTTTAATTTCTAGATCTATAGTAGAA 304

||||||||||||||||||||||| |||||||| |||||| ||||||||||||||||||||

Sbjct 246 ATAAGTTTTTGAATACTTCCTCCTTCTTTAACCCTTTTACTTTCTAGATCTATAGTAGAA 305

Query 305 AATGGAGCAGGAACAGGTTGAACTGTATACCCTCCTCTTTCTTCTGGAACTGCTCATGCA 364

||||||||||||||||||||||||||||| ||||||||||||||||||||||||||||||

Sbjct 306 AATGGAGCAGGAACAGGTTGAACTGTATATCCTCCTCTTTCTTCTGGAACTGCTCATGCA 365

Query 365 GGAGCTTCTGTAGATTTAGCTATTTTTTCTCTTCATTTAGCAGGAATTTCTTCTATTTTA 424

||||||||||||||||||||||||||||||||||||||||||||||||||||||||||||

Sbjct 366 GGAGCTTCTGTAGATTTAGCTATTTTTTCTCTTCATTTAGCAGGAATTTCTTCTATTTTA 425

Query 425 GGAGCAGTAAATTTTATTACAACTGTAATTAATATACGATCAACTGGAATTACACTTGAT 484

|||||||||||||||||||||||||||||||||||||||||| |||||||||| ||||||

Sbjct 426 GGAGCAGTAAATTTTATTACAACTGTAATTAATATACGATCAGCTGGAATTACCCTTGAT 485

Query 485 CGTTTACCTTTATTTGTCTGATCTGTAATTATTACAGCTATTTTATTACTTCTTTCATTA 544

||||||||||||||||| ||||| ||| ||||||||||||||||||||||||||||||||

Sbjct 486 CGTTTACCTTTATTTGTATGATCAGTAGTTATTACAGCTATTTTATTACTTCTTTCATTA 545

Query 545 CCAGTTTTAGCAGGAGCTATTACTATATTATTAACTGATCGAAATTTTAATACATCATTT 604

||||||||||||||||||||||||||||||||||||||||||||||||||||||||||||

Sbjct 546 CCAGTTTTAGCAGGAGCTATTACTATATTATTAACTGATCGAAATTTTAATACATCATTT 605

Query 605 TTTGACCCAATTGGAGGAGGTGACCCTATTCTTTATCAACATCTA 649

|||||||| ||||||||||||||||||||||||||||||||||||

Sbjct 606 TTTGACCCTATTGGAGGAGGTGACCCTATTCTTTATCAACATCTA 650

>Aedes riversi mitochondrial COI gene for cytochrome oxidase subunit 1, partial cds, isolate: L1065

Sequence ID: LC054391.1 Length: 650

Range 1: 8 to 650

Score:1044 bits(565), Expect:0.0,

Identities:619/645(96%), Gaps:3/645(0%), Strand: Plus/Plus

Query 6 TATTTTTGGAGTATGATCGGGGA-AGTCGGAACTTCATTAAGAGTTTTAATTCGAACTGA 64

|||||||||||||||||| || | ||||||||||||||||||||||||||||||||||||

Sbjct 8 TATTTTTGGAGTATGATCTGGAATAGTCGGAACTTCATTAAGAGTTTTAATTCGAACTGA 67

Query 65 ACTTAGTCACCCTGGAATATTTATCGGAAATGATCAAATTTATAATGTAATTCGTTACTA 124

||||||||| |||||||||||||| |||||||| |||||||||||||||||| || |||

Sbjct 68 ACTTAGTCATCCTGGAATATTTATTGGAAATGACCAAATTTATAATGTAATT-GTGACT- 125

Query 125 GCTCATGCATTTATTATAAttttttttATAGTAATACCTATTATAATTGGAGGATTTGGA 184

||||||||||||||||||||||||||||||||||||||||||||||||||||||||||||

Sbjct 126 GCTCATGCATTTATTATAATTTTTTTTATAGTAATACCTATTATAATTGGAGGATTTGGA 185

Query 185 AATTGGTTAGTCCCATTAATATTAGGTGCTCCTGATATAGCTTTCCCTCGAATAAATAAT 244

||||| ||||||||| ||||||||||||| ||||| ||||| || |||||||||||||||

Sbjct 186 AATTGATTAGTCCCACTAATATTAGGTGCCCCTGACATAGCCTTTCCTCGAATAAATAAT 245

Query 245 ATAAGTTTTTGAATACTTCCTCCCTCTTTAACTCTTTTAATTTCTAGATCTATAGTAGAA 304

||||||||||||||||||||||| |||||||| |||||| ||||||||||||||||||||

Sbjct 246 ATAAGTTTTTGAATACTTCCTCCTTCTTTAACCCTTTTACTTTCTAGATCTATAGTAGAA 305

Query 305 AATGGAGCAGGAACAGGTTGAACTGTATACCCTCCTCTTTCTTCTGGAACTGCTCATGCA 364

||||||||||||||||||||||||||||| ||||||||||||||||||||||||||||||

Sbjct 306 AATGGAGCAGGAACAGGTTGAACTGTATATCCTCCTCTTTCTTCTGGAACTGCTCATGCA 365

Query 365 GGAGCTTCTGTAGATTTAGCTATTTTTTCTCTTCATTTAGCAGGAATTTCTTCTATTTTA 424

||||||||||||||||||||||||||||||||||||||||||||||||||||||||||||

Sbjct 366 GGAGCTTCTGTAGATTTAGCTATTTTTTCTCTTCATTTAGCAGGAATTTCTTCTATTTTA 425

Query 425 GGAGCAGTAAATTTTATTACAACTGTAATTAATATACGATCAACTGGAATTACACTTGAT 484

|| ||||||||||||||||||||||||||||||||||||||| |||||||||| ||||||

Sbjct 426 GGGGCAGTAAATTTTATTACAACTGTAATTAATATACGATCAGCTGGAATTACCCTTGAT 485

Query 485 CGTTTACCTTTATTTGTCTGATCTGTAATTATTACAGCTATTTTATTACTTCTTTCATTA 544

||||||||||||||||| ||||| ||| ||||||||||||||||||||||||||||||||

Sbjct 486 CGTTTACCTTTATTTGTATGATCAGTAGTTATTACAGCTATTTTATTACTTCTTTCATTA 545

Query 545 CCAGTTTTAGCAGGAGCTATTACTATATTATTAACTGATCGAAATTTTAATACATCATTT 604

||||||||||||||||||||||||||||||||||||||||||||||||||||||||||||

Sbjct 546 CCAGTTTTAGCAGGAGCTATTACTATATTATTAACTGATCGAAATTTTAATACATCATTT 605

Query 605 TTTGACCCAATTGGAGGAGGTGACCCTATTCTTTATCAACATCTA 649

|||||||| ||||||||||||||||||||||||||||||||||||

Sbjct 606 TTTGACCCTATTGGAGGAGGTGACCCTATTCTTTATCAACATCTA 650

>Aedes riversi mitochondrial COI gene for cytochrome oxidase subunit 1, partial cds, isolate: 1013YON2011

Sequence ID: AB738283.1 Length: 658

Range 1: 13 to 658

Score:1044 bits(565), Expect:0.0,

Identities:621/648(96%), Gaps:3/648(0%), Strand: Plus/Plus

Query 6 TATTTTTGGAGTATGATCGGGGA-AGTCGGAACTTCATTAAGAGTTTTAATTCGAACTGA 64

|||||||||||||||||| || | ||| ||||||||||||||||||||||||||||||||

Sbjct 13 TATTTTTGGAGTATGATCTGGAATAGTTGGAACTTCATTAAGAGTTTTAATTCGAACTGA 72

Query 65 ACTTAGTCACCCTGGAATATTTATCGGAAATGATCAAATTTATAATGTAATTCGTTACTA 124

||||||||| ||||| |||||||| ||||||||||||||||||||||||||| ||||||

Sbjct 73 ACTTAGTCATCCTGGGATATTTATTGGAAATGATCAAATTTATAATGTAATT-GTTACT- 130

Query 125 GCTCATGCATTTATTATAAttttttttATAGTAATACCTATTATAATTGGAGGATTTGGA 184

||||||||||||||||||||||||||||||||||||||||||||||||||||||||||||

Sbjct 131 GCTCATGCATTTATTATAATTTTTTTTATAGTAATACCTATTATAATTGGAGGATTTGGA 190

Query 185 AATTGGTTAGTCCCATTAATATTAGGTGCTCCTGATATAGCTTTCCCTCGAATAAATAAT 244

||||| ||||||||| ||||||||||||| ||||| ||||| || |||||||||||||||

Sbjct 191 AATTGATTAGTCCCACTAATATTAGGTGCCCCTGACATAGCCTTTCCTCGAATAAATAAT 250

Query 245 ATAAGTTTTTGAATACTTCCTCCCTCTTTAACTCTTTTAATTTCTAGATCTATAGTAGAA 304

||||||||||||||||||||||| |||||||| |||||| ||||||||||||||||||||

Sbjct 251 ATAAGTTTTTGAATACTTCCTCCTTCTTTAACCCTTTTACTTTCTAGATCTATAGTAGAA 310

Query 305 AATGGAGCAGGAACAGGTTGAACTGTATACCCTCCTCTTTCTTCTGGAACTGCTCATGCA 364

|||||||||||||||||||| |||||||| ||||||||||||||||||||||| ||||||

Sbjct 311 AATGGAGCAGGAACAGGTTGGACTGTATATCCTCCTCTTTCTTCTGGAACTGCCCATGCA 370

Query 365 GGAGCTTCTGTAGATTTAGCTATTTTTTCTCTTCATTTAGCAGGAATTTCTTCTATTTTA 424

||||||||||||||||||||||||||||||||||||||||||||||||||||||||||||

Sbjct 371 GGAGCTTCTGTAGATTTAGCTATTTTTTCTCTTCATTTAGCAGGAATTTCTTCTATTTTA 430

Query 425 GGAGCAGTAAATTTTATTACAACTGTAATTAATATACGATCAACTGGAATTACACTTGAT 484

|||||||||||||||||||||||||||||||||||||||||| |||||||||||||||||

Sbjct 431 GGAGCAGTAAATTTTATTACAACTGTAATTAATATACGATCAGCTGGAATTACACTTGAT 490

Query 485 CGTTTACCTTTATTTGTCTGATCTGTAATTATTACAGCTATTTTATTACTTCTTTCATTA 544

||||||||||||||||| ||||| ||| ||||||||||||||||||||||||||||||||

Sbjct 491 CGTTTACCTTTATTTGTTTGATCAGTAGTTATTACAGCTATTTTATTACTTCTTTCATTA 550

Query 545 CCAGTTTTAGCAGGAGCTATTACTATATTATTAACTGATCGAAATTTTAATACATCATTT 604

||||||||||||||||||||||||||||||||||||||||||||||||||||||||||||

Sbjct 551 CCAGTTTTAGCAGGAGCTATTACTATATTATTAACTGATCGAAATTTTAATACATCATTT 610

Query 605 TTTGACCCAATTGGAGGAGGTGACCCTATTCTTTATCAACATCTATTT 652

||||| || |||||||||||||||||||||||||||||||||||||||

Sbjct 611 TTTGATCCTATTGGAGGAGGTGACCCTATTCTTTATCAACATCTATTT 658

>Aedes riversi mitochondrial COI gene for cytochrome oxidase subunit 1, partial cds, isolate: 318AMA2010

Sequence ID: AB738131.1 Length: 658

Range 1: 13 to 658

Score:1044 bits(565), Expect:0.0,

Identities:621/648(96%), Gaps:3/648(0%), Strand: Plus/Plus

Query 6 TATTTTTGGAGTATGATCGGGGA-AGTCGGAACTTCATTAAGAGTTTTAATTCGAACTGA 64

|||||||||||||||||| || | ||| ||||||||||||||||||||||||||||||||

Sbjct 13 TATTTTTGGAGTATGATCTGGAATAGTTGGAACTTCATTAAGAGTTTTAATTCGAACTGA 72

Query 65 ACTTAGTCACCCTGGAATATTTATCGGAAATGATCAAATTTATAATGTAATTCGTTACTA 124

||||||||| ||||| |||||||| |||||||| |||||||||||||||||| ||||||

Sbjct 73 ACTTAGTCATCCTGGGATATTTATTGGAAATGACCAAATTTATAATGTAATT-GTTACT- 130

Query 125 GCTCATGCATTTATTATAAttttttttATAGTAATACCTATTATAATTGGAGGATTTGGA 184

||||||||||||||||||||||||||||||||||||||||||||||||||||||||||||

Sbjct 131 GCTCATGCATTTATTATAATTTTTTTTATAGTAATACCTATTATAATTGGAGGATTTGGA 190

Query 185 AATTGGTTAGTCCCATTAATATTAGGTGCTCCTGATATAGCTTTCCCTCGAATAAATAAT 244

||||| ||||||||| ||||||||||||| ||||| ||||| || |||||||||||||||

Sbjct 191 AATTGATTAGTCCCACTAATATTAGGTGCCCCTGACATAGCCTTTCCTCGAATAAATAAT 250

Query 245 ATAAGTTTTTGAATACTTCCTCCCTCTTTAACTCTTTTAATTTCTAGATCTATAGTAGAA 304

||||||||||||||||||||||| |||||||| |||||| ||||||||||||||||||||

Sbjct 251 ATAAGTTTTTGAATACTTCCTCCTTCTTTAACCCTTTTACTTTCTAGATCTATAGTAGAA 310

Query 305 AATGGAGCAGGAACAGGTTGAACTGTATACCCTCCTCTTTCTTCTGGAACTGCTCATGCA 364

||||||||||||||||||||||||||||| |||||||||||||||||||||||||||||

Sbjct 311 AATGGAGCAGGAACAGGTTGAACTGTATATCCTCCTCTTTCTTCTGGAACTGCTCATGCG 370

Query 365 GGAGCTTCTGTAGATTTAGCTATTTTTTCTCTTCATTTAGCAGGAATTTCTTCTATTTTA 424

||||||||||||||||||||||||||||||||||||||||||||||||||||||||||||

Sbjct 371 GGAGCTTCTGTAGATTTAGCTATTTTTTCTCTTCATTTAGCAGGAATTTCTTCTATTTTA 430

Query 425 GGAGCAGTAAATTTTATTACAACTGTAATTAATATACGATCAACTGGAATTACACTTGAT 484

|||||||||||||||||||||||||||||||||||||||||| |||||||||||||||||

Sbjct 431 GGAGCAGTAAATTTTATTACAACTGTAATTAATATACGATCAGCTGGAATTACACTTGAT 490

Query 485 CGTTTACCTTTATTTGTCTGATCTGTAATTATTACAGCTATTTTATTACTTCTTTCATTA 544

||||||||||||||||| ||||| ||| ||||||||||||||||||||||||||||||||

Sbjct 491 CGTTTACCTTTATTTGTTTGATCAGTAGTTATTACAGCTATTTTATTACTTCTTTCATTA 550

Query 545 CCAGTTTTAGCAGGAGCTATTACTATATTATTAACTGATCGAAATTTTAATACATCATTT 604

||||||||||||||||||||||||||||||||||||||||||||||||||||||||||||

Sbjct 551 CCAGTTTTAGCAGGAGCTATTACTATATTATTAACTGATCGAAATTTTAATACATCATTT 610

Query 605 TTTGACCCAATTGGAGGAGGTGACCCTATTCTTTATCAACATCTATTT 652

||||| || |||||||||||||||||||||||||||||||||||||||

Sbjct 611 TTTGATCCTATTGGAGGAGGTGACCCTATTCTTTATCAACATCTATTT 658

>Aedes riversi mitochondrial COI gene for cytochrome oxidase subunit 1, partial cds, isolate: 309OKI2010

Sequence ID: AB738126.1 Length: 658

>Aedes riversi mitochondrial COI gene for cytochrome oxidase subunit 1, partial cds, isolate: 433OKI2011

Sequence ID: AB738171.1 Length: 658

Range 1: 13 to 658

Score:1044 bits(565), Expect:0.0,

Identities:621/648(96%), Gaps:3/648(0%), Strand: Plus/Plus

Query 6 TATTTTTGGAGTATGATCGGGGA-AGTCGGAACTTCATTAAGAGTTTTAATTCGAACTGA 64

|||||||||||||||||| || | ||| ||||||||||||||||||||||||||||||||

Sbjct 13 TATTTTTGGAGTATGATCTGGAATAGTTGGAACTTCATTAAGAGTTTTAATTCGAACTGA 72

Query 65 ACTTAGTCACCCTGGAATATTTATCGGAAATGATCAAATTTATAATGTAATTCGTTACTA 124

||||||||| |||||||||||||| ||||||||||||||||||||||||||| ||||||

Sbjct 73 ACTTAGTCATCCTGGAATATTTATTGGAAATGATCAAATTTATAATGTAATT-GTTACT- 130

Query 125 GCTCATGCATTTATTATAAttttttttATAGTAATACCTATTATAATTGGAGGATTTGGA 184

||||||||||||||||||||||||||||||||||||||||||||||||||||||||||||

Sbjct 131 GCTCATGCATTTATTATAATTTTTTTTATAGTAATACCTATTATAATTGGAGGATTTGGA 190

Query 185 AATTGGTTAGTCCCATTAATATTAGGTGCTCCTGATATAGCTTTCCCTCGAATAAATAAT 244

||||| ||||||||| ||||||||||||| ||||| ||||| || |||||||||||||||

Sbjct 191 AATTGATTAGTCCCACTAATATTAGGTGCCCCTGACATAGCCTTTCCTCGAATAAATAAT 250

Query 245 ATAAGTTTTTGAATACTTCCTCCCTCTTTAACTCTTTTAATTTCTAGATCTATAGTAGAA 304

||||||||||||||||||||||| |||||||| |||||| ||||||||||||||||||||

Sbjct 251 ATAAGTTTTTGAATACTTCCTCCTTCTTTAACCCTTTTACTTTCTAGATCTATAGTAGAA 310

Query 305 AATGGAGCAGGAACAGGTTGAACTGTATACCCTCCTCTTTCTTCTGGAACTGCTCATGCA 364

||||| |||||||||||||| |||||||| ||||||||||||||||||||||||||||||

Sbjct 311 AATGGGGCAGGAACAGGTTGGACTGTATATCCTCCTCTTTCTTCTGGAACTGCTCATGCA 370

Query 365 GGAGCTTCTGTAGATTTAGCTATTTTTTCTCTTCATTTAGCAGGAATTTCTTCTATTTTA 424

||||||||||||||||||||||||||||||||||||||||||||||||||||||||||||

Sbjct 371 GGAGCTTCTGTAGATTTAGCTATTTTTTCTCTTCATTTAGCAGGAATTTCTTCTATTTTA 430

Query 425 GGAGCAGTAAATTTTATTACAACTGTAATTAATATACGATCAACTGGAATTACACTTGAT 484

|||||||||||||||||||||||||||||||||||||||||| |||||||||||||||||

Sbjct 431 GGAGCAGTAAATTTTATTACAACTGTAATTAATATACGATCAGCTGGAATTACACTTGAT 490

Query 485 CGTTTACCTTTATTTGTCTGATCTGTAATTATTACAGCTATTTTATTACTTCTTTCATTA 544

||||||||||||||||| ||||| ||| ||||||||||||||||||||||||||||||||

Sbjct 491 CGTTTACCTTTATTTGTTTGATCAGTAGTTATTACAGCTATTTTATTACTTCTTTCATTA 550

Query 545 CCAGTTTTAGCAGGAGCTATTACTATATTATTAACTGATCGAAATTTTAATACATCATTT 604

||||||||||||||||||||||||||||||||||||||||||||||||||||||||||||

Sbjct 551 CCAGTTTTAGCAGGAGCTATTACTATATTATTAACTGATCGAAATTTTAATACATCATTT 610

Query 605 TTTGACCCAATTGGAGGAGGTGACCCTATTCTTTATCAACATCTATTT 652

||||| || |||||||||||||| ||||||||||||||||||||||||

Sbjct 611 TTTGATCCTATTGGAGGAGGTGATCCTATTCTTTATCAACATCTATTT 658

>Aedes riversi mitochondrial COI gene for cytochrome oxidase subunit 1, partial cds, isolate: L2193

Sequence ID: LC054393.1 Length: 650

Range 1: 8 to 650

Score:1038 bits(562), Expect:0.0,

Identities:618/645(96%), Gaps:3/645(0%), Strand: Plus/Plus

Query 6 TATTTTTGGAGTATGATCGGGGA-AGTCGGAACTTCATTAAGAGTTTTAATTCGAACTGA 64

|||||||||||||||||| || | ||||||||||||||||||||||||||||||||||||

Sbjct 8 TATTTTTGGAGTATGATCTGGAATAGTCGGAACTTCATTAAGAGTTTTAATTCGAACTGA 67

Query 65 ACTTAGTCACCCTGGAATATTTATCGGAAATGATCAAATTTATAATGTAATTCGTTACTA 124

||||||||| |||||||||||||| |||||||| |||||||||||||||||| || |||

Sbjct 68 ACTTAGTCATCCTGGAATATTTATTGGAAATGACCAAATTTATAATGTAATT-GTGACT- 125

Query 125 GCTCATGCATTTATTATAAttttttttATAGTAATACCTATTATAATTGGAGGATTTGGA 184

||||||||||||||||||||||||||||||||||||||||||||||||||||||||||||

Sbjct 126 GCTCATGCATTTATTATAATTTTTTTTATAGTAATACCTATTATAATTGGAGGATTTGGA 185

Query 185 AATTGGTTAGTCCCATTAATATTAGGTGCTCCTGATATAGCTTTCCCTCGAATAAATAAT 244

||||| ||||||||| ||||||||||||| ||||| ||||| || |||||||||||||||

Sbjct 186 AATTGATTAGTCCCACTAATATTAGGTGCCCCTGACATAGCCTTTCCTCGAATAAATAAT 245

Query 245 ATAAGTTTTTGAATACTTCCTCCCTCTTTAACTCTTTTAATTTCTAGATCTATAGTAGAA 304

||||||||||||||||||||||| |||||||| |||||| ||||||||||||||||||||

Sbjct 246 ATAAGTTTTTGAATACTTCCTCCTTCTTTAACCCTTTTACTTTCTAGATCTATAGTAGAA 305

Query 305 AATGGAGCAGGAACAGGTTGAACTGTATACCCTCCTCTTTCTTCTGGAACTGCTCATGCA 364

||||||||||||||||||||||||||||| ||||||||||||||||||||||||||||||

Sbjct 306 AATGGAGCAGGAACAGGTTGAACTGTATATCCTCCTCTTTCTTCTGGAACTGCTCATGCA 365

Query 365 GGAGCTTCTGTAGATTTAGCTATTTTTTCTCTTCATTTAGCAGGAATTTCTTCTATTTTA 424

||||||||||||||||||||||||||||||||||||||||||||||||||||||||||||

Sbjct 366 GGAGCTTCTGTAGATTTAGCTATTTTTTCTCTTCATTTAGCAGGAATTTCTTCTATTTTA 425

Query 425 GGAGCAGTAAATTTTATTACAACTGTAATTAATATACGATCAACTGGAATTACACTTGAT 484

|| ||||||||||||||||||||||||||||||||||||||| ||||||| || ||||||

Sbjct 426 GGGGCAGTAAATTTTATTACAACTGTAATTAATATACGATCAGCTGGAATCACCCTTGAT 485

Query 485 CGTTTACCTTTATTTGTCTGATCTGTAATTATTACAGCTATTTTATTACTTCTTTCATTA 544

||||||||||||||||| ||||| ||| ||||||||||||||||||||||||||||||||

Sbjct 486 CGTTTACCTTTATTTGTATGATCAGTAGTTATTACAGCTATTTTATTACTTCTTTCATTA 545

Query 545 CCAGTTTTAGCAGGAGCTATTACTATATTATTAACTGATCGAAATTTTAATACATCATTT 604

||||||||||||||||||||||||||||||||||||||||||||||||||||||||||||

Sbjct 546 CCAGTTTTAGCAGGAGCTATTACTATATTATTAACTGATCGAAATTTTAATACATCATTT 605

Query 605 TTTGACCCAATTGGAGGAGGTGACCCTATTCTTTATCAACATCTA 649

|||||||| ||||||||||||||||||||||||||||||||||||

Sbjct 606 TTTGACCCTATTGGAGGAGGTGACCCTATTCTTTATCAACATCTA 650

>Aedes riversi mitochondrial COI gene for cytochrome oxidase subunit 1, partial cds, isolate: L1106

Sequence ID: LC054392.1 Length: 650

Range 1: 8 to 650

Score:1038 bits(562), Expect:0.0,

Identities:618/645(96%), Gaps:3/645(0%), Strand: Plus/Plus

Query 6 TATTTTTGGAGTATGATCGGGGA-AGTCGGAACTTCATTAAGAGTTTTAATTCGAACTGA 64

|||||||||||||||||| || | ||||||||||||||||||||||||||||||||||||

Sbjct 8 TATTTTTGGAGTATGATCTGGAATAGTCGGAACTTCATTAAGAGTTTTAATTCGAACTGA 67

Query 65 ACTTAGTCACCCTGGAATATTTATCGGAAATGATCAAATTTATAATGTAATTCGTTACTA 124

||||||||| |||||||||||||| |||||||| |||||||||||||||||| || |||

Sbjct 68 ACTTAGTCATCCTGGAATATTTATTGGAAATGACCAAATTTATAATGTAATT-GTGACT- 125

Query 125 GCTCATGCATTTATTATAAttttttttATAGTAATACCTATTATAATTGGAGGATTTGGA 184

||||||||||||||||||||||||||||||||||||||||||||||||||||||||||||

Sbjct 126 GCTCATGCATTTATTATAATTTTTTTTATAGTAATACCTATTATAATTGGAGGATTTGGA 185

Query 185 AATTGGTTAGTCCCATTAATATTAGGTGCTCCTGATATAGCTTTCCCTCGAATAAATAAT 244

||||| ||||||||| ||||||||||||| ||||| ||||| || |||||||||||||||

Sbjct 186 AATTGATTAGTCCCACTAATATTAGGTGCCCCTGACATAGCCTTTCCTCGAATAAATAAT 245

Query 245 ATAAGTTTTTGAATACTTCCTCCCTCTTTAACTCTTTTAATTTCTAGATCTATAGTAGAA 304

||||||||||||||||||||||| |||||||| |||||| ||||||||||||||||||||

Sbjct 246 ATAAGTTTTTGAATACTTCCTCCTTCTTTAACCCTTTTACTTTCTAGATCTATAGTAGAA 305

Query 305 AATGGAGCAGGAACAGGTTGAACTGTATACCCTCCTCTTTCTTCTGGAACTGCTCATGCA 364

||||||||||||||||| ||||||||||| ||||||||||||||||||||||||||||||

Sbjct 306 AATGGAGCAGGAACAGGCTGAACTGTATATCCTCCTCTTTCTTCTGGAACTGCTCATGCA 365

Query 365 GGAGCTTCTGTAGATTTAGCTATTTTTTCTCTTCATTTAGCAGGAATTTCTTCTATTTTA 424

||||||||||||||||||||||||||||||||||||||||||||||||||||||||||||

Sbjct 366 GGAGCTTCTGTAGATTTAGCTATTTTTTCTCTTCATTTAGCAGGAATTTCTTCTATTTTA 425

Query 425 GGAGCAGTAAATTTTATTACAACTGTAATTAATATACGATCAACTGGAATTACACTTGAT 484

|| ||||||||||||||||||||||||||||||||||||||| |||||||||| ||||||

Sbjct 426 GGGGCAGTAAATTTTATTACAACTGTAATTAATATACGATCAGCTGGAATTACCCTTGAT 485

Query 485 CGTTTACCTTTATTTGTCTGATCTGTAATTATTACAGCTATTTTATTACTTCTTTCATTA 544

||||||||||||||||| ||||| ||| ||||||||||||||||||||||||||||||||

Sbjct 486 CGTTTACCTTTATTTGTATGATCAGTAGTTATTACAGCTATTTTATTACTTCTTTCATTA 545

Query 545 CCAGTTTTAGCAGGAGCTATTACTATATTATTAACTGATCGAAATTTTAATACATCATTT 604

||||||||||||||||||||||||||||||||||||||||||||||||||||||||||||

Sbjct 546 CCAGTTTTAGCAGGAGCTATTACTATATTATTAACTGATCGAAATTTTAATACATCATTT 605

Query 605 TTTGACCCAATTGGAGGAGGTGACCCTATTCTTTATCAACATCTA 649

|||||||| ||||||||||||||||||||||||||||||||||||

Sbjct 606 TTTGACCCTATTGGAGGAGGTGACCCTATTCTTTATCAACATCTA 650

>Aedes riversi mitochondrial COI gene for cytochrome oxidase subunit 1, partial cds, isolate: L1055

Sequence ID: LC054390.1 Length: 650

Range 1: 8 to 650

Score:1038 bits(562), Expect:0.0,

Identities:618/645(96%), Gaps:3/645(0%), Strand: Plus/Plus

Query 6 TATTTTTGGAGTATGATCGGGGA-AGTCGGAACTTCATTAAGAGTTTTAATTCGAACTGA 64

||||||||||||||| || || | ||||||||||||||||||||||||||||||||||||

Sbjct 8 TATTTTTGGAGTATGGTCTGGAATAGTCGGAACTTCATTAAGAGTTTTAATTCGAACTGA 67

Query 65 ACTTAGTCACCCTGGAATATTTATCGGAAATGATCAAATTTATAATGTAATTCGTTACTA 124

||||||||| |||||||||||||| |||||||| |||||||||||||||||| || |||

Sbjct 68 ACTTAGTCATCCTGGAATATTTATTGGAAATGACCAAATTTATAATGTAATT-GTGACT- 125

Query 125 GCTCATGCATTTATTATAAttttttttATAGTAATACCTATTATAATTGGAGGATTTGGA 184

||||||||||||||||||||||||||||||||||||||||||||||||||||||||||||

Sbjct 126 GCTCATGCATTTATTATAATTTTTTTTATAGTAATACCTATTATAATTGGAGGATTTGGA 185

Query 185 AATTGGTTAGTCCCATTAATATTAGGTGCTCCTGATATAGCTTTCCCTCGAATAAATAAT 244

||||| ||||||||| ||||||||||||| ||||| ||||| || |||||||||||||||

Sbjct 186 AATTGATTAGTCCCACTAATATTAGGTGCCCCTGACATAGCCTTTCCTCGAATAAATAAT 245

Query 245 ATAAGTTTTTGAATACTTCCTCCCTCTTTAACTCTTTTAATTTCTAGATCTATAGTAGAA 304

||||||||||||||||||||||| |||||||| |||||| ||||||||||||||||||||

Sbjct 246 ATAAGTTTTTGAATACTTCCTCCTTCTTTAACCCTTTTACTTTCTAGATCTATAGTAGAA 305

Query 305 AATGGAGCAGGAACAGGTTGAACTGTATACCCTCCTCTTTCTTCTGGAACTGCTCATGCA 364

||||||||||||||||||||||||||||| ||||||||||||||||||||||||||||||

Sbjct 306 AATGGAGCAGGAACAGGTTGAACTGTATATCCTCCTCTTTCTTCTGGAACTGCTCATGCA 365

Query 365 GGAGCTTCTGTAGATTTAGCTATTTTTTCTCTTCATTTAGCAGGAATTTCTTCTATTTTA 424

||||||||||||||||||||||||||||||||||||||||||||||||||||||||||||

Sbjct 366 GGAGCTTCTGTAGATTTAGCTATTTTTTCTCTTCATTTAGCAGGAATTTCTTCTATTTTA 425

Query 425 GGAGCAGTAAATTTTATTACAACTGTAATTAATATACGATCAACTGGAATTACACTTGAT 484

|| ||||||||||||||||||||||||||||||||||||||| |||||||||| ||||||

Sbjct 426 GGGGCAGTAAATTTTATTACAACTGTAATTAATATACGATCAGCTGGAATTACCCTTGAT 485

Query 485 CGTTTACCTTTATTTGTCTGATCTGTAATTATTACAGCTATTTTATTACTTCTTTCATTA 544

||||||||||||||||| ||||| ||| ||||||||||||||||||||||||||||||||

Sbjct 486 CGTTTACCTTTATTTGTGTGATCAGTAGTTATTACAGCTATTTTATTACTTCTTTCATTA 545

Query 545 CCAGTTTTAGCAGGAGCTATTACTATATTATTAACTGATCGAAATTTTAATACATCATTT 604

||||||||||||||||||||||||||||||||||||||||||||||||||||||||||||

Sbjct 546 CCAGTTTTAGCAGGAGCTATTACTATATTATTAACTGATCGAAATTTTAATACATCATTT 605

Query 605 TTTGACCCAATTGGAGGAGGTGACCCTATTCTTTATCAACATCTA 649

|||||||| ||||||||||||||||||||||||||||||||||||

Sbjct 606 TTTGACCCTATTGGAGGAGGTGACCCTATTCTTTATCAACATCTA 650

>Aedes riversi mitochondrial COI gene for cytochrome oxidase subunit 1, partial cds, isolate: 1083KUM2012

Sequence ID: AB738307.1 Length: 658

>Aedes riversi mitochondrial COI gene for cytochrome oxidase subunit 1, partial cds, isolate: 1085KUM2012

Sequence ID: AB738309.1 Length: 658

Range 1: 13 to 658

Score:1038 bits(562), Expect:0.0,

Identities:620/648(96%), Gaps:3/648(0%), Strand: Plus/Plus

Query 6 TATTTTTGGAGTATGATCGGGGA-AGTCGGAACTTCATTAAGAGTTTTAATTCGAACTGA 64

|||||||||||||||||| || | ||| ||||||||||||||||||||||||||||||||

Sbjct 13 TATTTTTGGAGTATGATCTGGAATAGTTGGAACTTCATTAAGAGTTTTAATTCGAACTGA 72

Query 65 ACTTAGTCACCCTGGAATATTTATCGGAAATGATCAAATTTATAATGTAATTCGTTACTA 124

||||||||| ||||| |||||||| ||||||||||||||||||||||||||| ||||||

Sbjct 73 ACTTAGTCATCCTGGGATATTTATTGGAAATGATCAAATTTATAATGTAATT-GTTACT- 130

Query 125 GCTCATGCATTTATTATAAttttttttATAGTAATACCTATTATAATTGGAGGATTTGGA 184

||||||||||||||||||||||||||||||||||||||||||||||||||||||||||||

Sbjct 131 GCTCATGCATTTATTATAATTTTTTTTATAGTAATACCTATTATAATTGGAGGATTTGGA 190

Query 185 AATTGGTTAGTCCCATTAATATTAGGTGCTCCTGATATAGCTTTCCCTCGAATAAATAAT 244

||||| ||||||||| ||||||||||||| ||||| ||||| || |||||||||||||||

Sbjct 191 AATTGATTAGTCCCACTAATATTAGGTGCCCCTGACATAGCCTTTCCTCGAATAAATAAT 250

Query 245 ATAAGTTTTTGAATACTTCCTCCCTCTTTAACTCTTTTAATTTCTAGATCTATAGTAGAA 304

||||||||||||||||||||||| |||||||| |||||| ||||||||||||||||||||

Sbjct 251 ATAAGTTTTTGAATACTTCCTCCTTCTTTAACCCTTTTACTTTCTAGATCTATAGTAGAA 310

Query 305 AATGGAGCAGGAACAGGTTGAACTGTATACCCTCCTCTTTCTTCTGGAACTGCTCATGCA 364

||||| |||||||||||||| |||||||| ||||||||||||||||||||||||||||||

Sbjct 311 AATGGGGCAGGAACAGGTTGGACTGTATATCCTCCTCTTTCTTCTGGAACTGCTCATGCA 370

Query 365 GGAGCTTCTGTAGATTTAGCTATTTTTTCTCTTCATTTAGCAGGAATTTCTTCTATTTTA 424

||||||||||||||||||||||||||||||||||||||||||||||||||||||||||||

Sbjct 371 GGAGCTTCTGTAGATTTAGCTATTTTTTCTCTTCATTTAGCAGGAATTTCTTCTATTTTA 430

Query 425 GGAGCAGTAAATTTTATTACAACTGTAATTAATATACGATCAACTGGAATTACACTTGAT 484

|||||||||||||||||||||||||||||||||||||||||| |||||||||||||||||

Sbjct 431 GGAGCAGTAAATTTTATTACAACTGTAATTAATATACGATCAGCTGGAATTACACTTGAT 490

Query 485 CGTTTACCTTTATTTGTCTGATCTGTAATTATTACAGCTATTTTATTACTTCTTTCATTA 544

||||||||||||||||| ||||| ||| ||||||||||||||||||||||||||||||||

Sbjct 491 CGTTTACCTTTATTTGTTTGATCAGTAGTTATTACAGCTATTTTATTACTTCTTTCATTA 550

Query 545 CCAGTTTTAGCAGGAGCTATTACTATATTATTAACTGATCGAAATTTTAATACATCATTT 604

||||||||||||||||||||||||||||||||||||||||||||||||||||||||||||

Sbjct 551 CCAGTTTTAGCAGGAGCTATTACTATATTATTAACTGATCGAAATTTTAATACATCATTT 610

Query 605 TTTGACCCAATTGGAGGAGGTGACCCTATTCTTTATCAACATCTATTT 652

||||| || |||||||||||||| ||||||||||||||||||||||||

Sbjct 611 TTTGATCCTATTGGAGGAGGTGATCCTATTCTTTATCAACATCTATTT 658

>Aedes riversi mitochondrial COI gene for cytochrome oxidase subunit 1, partial cds, isolate: 538IRI2011

Sequence ID: AB738193.1 Length: 658

Range 1: 13 to 658

Score:1038 bits(562), Expect:0.0,

Identities:620/648(96%), Gaps:3/648(0%), Strand: Plus/Plus

Query 6 TATTTTTGGAGTATGATCGGGGA-AGTCGGAACTTCATTAAGAGTTTTAATTCGAACTGA 64

|||||||||||||||||| || | ||| ||||||||||||||||||||||||||||||||

Sbjct 13 TATTTTTGGAGTATGATCTGGAATAGTTGGAACTTCATTAAGAGTTTTAATTCGAACTGA 72

Query 65 ACTTAGTCACCCTGGAATATTTATCGGAAATGATCAAATTTATAATGTAATTCGTTACTA 124

||||||||| ||||| |||||||| ||||||||||||||||||||||||||| ||||||

Sbjct 73 ACTTAGTCATCCTGGGATATTTATTGGAAATGATCAAATTTATAATGTAATT-GTTACT- 130

Query 125 GCTCATGCATTTATTATAAttttttttATAGTAATACCTATTATAATTGGAGGATTTGGA 184

||||||||||||||||||||||||||||||||||||||||||||||||||||||||||||

Sbjct 131 GCTCATGCATTTATTATAATTTTTTTTATAGTAATACCTATTATAATTGGAGGATTTGGA 190

Query 185 AATTGGTTAGTCCCATTAATATTAGGTGCTCCTGATATAGCTTTCCCTCGAATAAATAAT 244

||||| ||||||||| ||||||||||||| ||||| ||||| || |||||||||||||||

Sbjct 191 AATTGATTAGTCCCACTAATATTAGGTGCCCCTGACATAGCCTTTCCTCGAATAAATAAT 250

Query 245 ATAAGTTTTTGAATACTTCCTCCCTCTTTAACTCTTTTAATTTCTAGATCTATAGTAGAA 304

||||||||||||||||||||||| |||||||| |||||| ||||||||||||||||||||

Sbjct 251 ATAAGTTTTTGAATACTTCCTCCTTCTTTAACCCTTTTACTTTCTAGATCTATAGTAGAA 310

Query 305 AATGGAGCAGGAACAGGTTGAACTGTATACCCTCCTCTTTCTTCTGGAACTGCTCATGCA 364

||||| |||||||||||||| |||||||| ||||||||||||||||||||||| ||||||

Sbjct 311 AATGGGGCAGGAACAGGTTGGACTGTATATCCTCCTCTTTCTTCTGGAACTGCCCATGCA 370

Query 365 GGAGCTTCTGTAGATTTAGCTATTTTTTCTCTTCATTTAGCAGGAATTTCTTCTATTTTA 424

||||||||||||||||||||||||||||||||||||||||||||||||||||||||||||

Sbjct 371 GGAGCTTCTGTAGATTTAGCTATTTTTTCTCTTCATTTAGCAGGAATTTCTTCTATTTTA 430

Query 425 GGAGCAGTAAATTTTATTACAACTGTAATTAATATACGATCAACTGGAATTACACTTGAT 484

|||||||||||||||||||||||||||||||||||||||||| |||||||||||||||||

Sbjct 431 GGAGCAGTAAATTTTATTACAACTGTAATTAATATACGATCAGCTGGAATTACACTTGAT 490

Query 485 CGTTTACCTTTATTTGTCTGATCTGTAATTATTACAGCTATTTTATTACTTCTTTCATTA 544

||||||||||||||||| ||||| ||| ||||||||||||||||||||||||||||||||

Sbjct 491 CGTTTACCTTTATTTGTTTGATCAGTAGTTATTACAGCTATTTTATTACTTCTTTCATTA 550

Query 545 CCAGTTTTAGCAGGAGCTATTACTATATTATTAACTGATCGAAATTTTAATACATCATTT 604

||||||||||||||||||||||||||||||||||||||||||||||||||||||||||||

Sbjct 551 CCAGTTTTAGCAGGAGCTATTACTATATTATTAACTGATCGAAATTTTAATACATCATTT 610

Query 605 TTTGACCCAATTGGAGGAGGTGACCCTATTCTTTATCAACATCTATTT 652

||||| || |||||||||||||||||||||||||||||||||||||||

Sbjct 611 TTTGATCCTATTGGAGGAGGTGACCCTATTCTTTATCAACATCTATTT 658

>Aedes riversi mitochondrial COI gene for cytochrome oxidase subunit 1, partial cds, isolate: 301TOK2010

Sequence ID: AB738123.1 Length: 658

>Aedes riversi mitochondrial COI gene for cytochrome oxidase subunit 1, partial cds, isolate: 676TOK2011

Sequence ID: AB738214.1 Length: 658

Range 1: 13 to 658

Score:1038 bits(562), Expect:0.0,

Identities:620/648(96%), Gaps:3/648(0%), Strand: Plus/Plus

Query 6 TATTTTTGGAGTATGATCGGGGA-AGTCGGAACTTCATTAAGAGTTTTAATTCGAACTGA 64

|||||||||||||||||| || | ||| ||||||||||||||||||||||||||||||||

Sbjct 13 TATTTTTGGAGTATGATCTGGAATAGTTGGAACTTCATTAAGAGTTTTAATTCGAACTGA 72

Query 65 ACTTAGTCACCCTGGAATATTTATCGGAAATGATCAAATTTATAATGTAATTCGTTACTA 124

||||||||| ||||| |||||||| |||||||| |||||||||||||||||| ||||||

Sbjct 73 ACTTAGTCATCCTGGGATATTTATTGGAAATGACCAAATTTATAATGTAATT-GTTACT- 130

Query 125 GCTCATGCATTTATTATAAttttttttATAGTAATACCTATTATAATTGGAGGATTTGGA 184

||||||||||||||||||||||||||||||||||||||||||||||||||||||||||||

Sbjct 131 GCTCATGCATTTATTATAATTTTTTTTATAGTAATACCTATTATAATTGGAGGATTTGGA 190

Query 185 AATTGGTTAGTCCCATTAATATTAGGTGCTCCTGATATAGCTTTCCCTCGAATAAATAAT 244

||||| ||||||||| ||||||||||||| ||||| ||||| || |||||||||||||||

Sbjct 191 AATTGATTAGTCCCACTAATATTAGGTGCCCCTGACATAGCCTTTCCTCGAATAAATAAT 250

Query 245 ATAAGTTTTTGAATACTTCCTCCCTCTTTAACTCTTTTAATTTCTAGATCTATAGTAGAA 304

||||||||||||||||||||||| |||||||| |||||| ||||||||||||||||||||

Sbjct 251 ATAAGTTTTTGAATACTTCCTCCTTCTTTAACCCTTTTACTTTCTAGATCTATAGTAGAA 310

Query 305 AATGGAGCAGGAACAGGTTGAACTGTATACCCTCCTCTTTCTTCTGGAACTGCTCATGCA 364

||||| |||||||||||||| |||||||| ||||||||||||||||||||||||||||||

Sbjct 311 AATGGGGCAGGAACAGGTTGGACTGTATATCCTCCTCTTTCTTCTGGAACTGCTCATGCA 370

Query 365 GGAGCTTCTGTAGATTTAGCTATTTTTTCTCTTCATTTAGCAGGAATTTCTTCTATTTTA 424

||||||||||||||||||||||||||||||||||||||||||||||||||||||||||||

Sbjct 371 GGAGCTTCTGTAGATTTAGCTATTTTTTCTCTTCATTTAGCAGGAATTTCTTCTATTTTA 430

Query 425 GGAGCAGTAAATTTTATTACAACTGTAATTAATATACGATCAACTGGAATTACACTTGAT 484

|||||||||||||||||||||||||||||||||||||||||| |||||||||||||||||

Sbjct 431 GGAGCAGTAAATTTTATTACAACTGTAATTAATATACGATCAGCTGGAATTACACTTGAT 490

Query 485 CGTTTACCTTTATTTGTCTGATCTGTAATTATTACAGCTATTTTATTACTTCTTTCATTA 544

||||||||||||||||| ||||| ||| ||||||||||||||||||||||||||||||||

Sbjct 491 CGTTTACCTTTATTTGTTTGATCAGTAGTTATTACAGCTATTTTATTACTTCTTTCATTA 550

Query 545 CCAGTTTTAGCAGGAGCTATTACTATATTATTAACTGATCGAAATTTTAATACATCATTT 604

||||||||||||||||||||||||||||||||||||||||||||||||||||||||||||

Sbjct 551 CCAGTTTTAGCAGGAGCTATTACTATATTATTAACTGATCGAAATTTTAATACATCATTT 610

Query 605 TTTGACCCAATTGGAGGAGGTGACCCTATTCTTTATCAACATCTATTT 652

||||| || |||||||||||||||||||||||||||||||||||||||

Sbjct 611 TTTGATCCTATTGGAGGAGGTGACCCTATTCTTTATCAACATCTATTT 658

>Aedes riversi mitochondrial COI gene for cytochrome oxidase subunit 1, partial cds, isolate: 274IRI2010

Sequence ID: AB738116.1 Length: 658

Range 1: 13 to 658

Score:1038 bits(562), Expect:0.0,

Identities:621/649(96%), Gaps:5/649(0%), Strand: Plus/Plus

Query 6 TATTTTTGGAGTATGATCGGGGA-AGTCGGAACTTCATTAAGAGTTTTAATTCGAACTGA 64

|||||||||||||||||| || | ||| ||||||||||||||||||||||||||||||||

Sbjct 13 TATTTTTGGAGTATGATCTGGAATAGTTGGAACTTCATTAAGAGTTTTAATTCGAACTGA 72

Query 65 ACTTAGTCACCCTGGAATATTTATCGGAAATGATCAAATTTATAATGTAATTCGTTACTA 124

||||||||| ||||| |||||||| ||||||||||||||||||||||||||| ||||||

Sbjct 73 ACTTAGTCATCCTGGGATATTTATTGGAAATGATCAAATTTATAATGTAATT-GTTACT- 130

Query 125 GCTCATGCATTTATTATAAttttttttATAGTAATACCTATTATAATTGGAGGATTTGGA 184

||||||||||||||||||||||||||||||||||||||||||||||||||||||||||||

Sbjct 131 GCTCATGCATTTATTATAATTTTTTTTATAGTAATACCTATTATAATTGGAGGATTTGGA 190

Query 185 AATTGGTTAGTCCCATTAATATTAGGTGCTCCTGATATAGC-TTTCCCTCGAATAAATAA 243

||||| ||||||||| ||||||||||||| ||||| ||||| |||||| |||||||||||

Sbjct 191 AATTGATTAGTCCCACTAATATTAGGTGCCCCTGACATAGCCTTTCCC-CGAATAAATAA 249

Query 244 TATAAGTTTTTGAATACTTCCTCCCTCTTTAACTCTTTTAATTTCTAGATCTATAGTAGA 303

||||||||||||||| |||||||| ||||||||||||||| |||||||||||||||||||

Sbjct 250 TATAAGTTTTTGAATGCTTCCTCCTTCTTTAACTCTTTTACTTTCTAGATCTATAGTAGA 309

Query 304 AAATGGAGCAGGAACAGGTTGAACTGTATACCCTCCTCTTTCTTCTGGAACTGCTCATGC 363

|||||| |||||||||||||| |||||||| ||||||||||||||||||||||| |||||

Sbjct 310 AAATGGGGCAGGAACAGGTTGGACTGTATATCCTCCTCTTTCTTCTGGAACTGCCCATGC 369

Query 364 AGGAGCTTCTGTAGATTTAGCTATTTTTTCTCTTCATTTAGCAGGAATTTCTTCTATTTT 423

||||||||||||||||||||||||||||||||||||||||||||||||||||||||||||

Sbjct 370 AGGAGCTTCTGTAGATTTAGCTATTTTTTCTCTTCATTTAGCAGGAATTTCTTCTATTTT 429

Query 424 AGGAGCAGTAAATTTTATTACAACTGTAATTAATATACGATCAACTGGAATTACACTTGA 483

||||||||||||||||||||||||||||||||||||||||||| ||||||||||||||||

Sbjct 430 AGGAGCAGTAAATTTTATTACAACTGTAATTAATATACGATCAGCTGGAATTACACTTGA 489

Query 484 TCGTTTACCTTTATTTGTCTGATCTGTAATTATTACAGCTATTTTATTACTTCTTTCATT 543

|||||||||||||||||| ||||| ||| |||||||||||||||||||||||||||||||

Sbjct 490 TCGTTTACCTTTATTTGTTTGATCAGTAGTTATTACAGCTATTTTATTACTTCTTTCATT 549

Query 544 ACCAGTTTTAGCAGGAGCTATTACTATATTATTAACTGATCGAAATTTTAATACATCATT 603

||||||||||||||||||||||||||||||||||||||||||||||||||||||||||||

Sbjct 550 ACCAGTTTTAGCAGGAGCTATTACTATATTATTAACTGATCGAAATTTTAATACATCATT 609

Query 604 TTTTGACCCAATTGGAGGAGGTGACCCTATTCTTTATCAACATCTATTT 652

|||||| || |||||||||||||||||||||||||||||||||||||||

Sbjct 610 TTTTGATCCTATTGGAGGAGGTGACCCTATTCTTTATCAACATCTATTT 658

>Aedes riversi mitochondrial COI gene for cytochrome oxidase subunit 1, partial cds, isolate: 1050YON2011

Sequence ID: AB738295.1 Length: 658

Range 1: 13 to 658

Score:1022 bits(553), Expect:0.0,

Identities:617/648(95%), Gaps:3/648(0%), Strand: Plus/Plus

Query 6 TATTTTTGGAGTATGATCGGGGA-AGTCGGAACTTCATTAAGAGTTTTAATTCGAACTGA 64

|||||||||||||||||| || | ||| ||||||||||||||||||||||||||||||||

Sbjct 13 TATTTTTGGAGTATGATCTGGAATAGTTGGAACTTCATTAAGAGTTTTAATTCGAACTGA 72

Query 65 ACTTAGTCACCCTGGAATATTTATCGGAAATGATCAAATTTATAATGTAATTCGTTACTA 124

||||||||| ||||| |||||||| |||||||| |||||||||||||||||| ||||||

Sbjct 73 ACTTAGTCATCCTGGGATATTTATTGGAAATGACCAAATTTATAATGTAATT-GTTACT- 130

Query 125 GCTCATGCATTTATTATAAttttttttATAGTAATACCTATTATAATTGGAGGATTTGGA 184

||||||||||||||||||||||||||||||||||||||||||||||||||||||||||||

Sbjct 131 GCTCATGCATTTATTATAATTTTTTTTATAGTAATACCTATTATAATTGGAGGATTTGGA 190

Query 185 AATTGGTTAGTCCCATTAATATTAGGTGCTCCTGATATAGCTTTCCCTCGAATAAATAAT 244

||||| ||||||||||||||||||||||| ||||| ||||| || ||||||||||||||

Sbjct 191 AATTGATTAGTCCCATTAATATTAGGTGCCCCTGACATAGCCTTTCCTCGAATAAATAAC 250

Query 245 ATAAGTTTTTGAATACTTCCTCCCTCTTTAACTCTTTTAATTTCTAGATCTATAGTAGAA 304

||||||||||||||||||||||| |||||||| |||||| ||||||||||||||||||||

Sbjct 251 ATAAGTTTTTGAATACTTCCTCCTTCTTTAACCCTTTTACTTTCTAGATCTATAGTAGAA 310

Query 305 AATGGAGCAGGAACAGGTTGAACTGTATACCCTCCTCTTTCTTCTGGAACTGCTCATGCA 364

||||| |||||||||||||| || ||||| ||||||||||||||||||||||| ||||||

Sbjct 311 AATGGGGCAGGAACAGGTTGGACCGTATATCCTCCTCTTTCTTCTGGAACTGCCCATGCA 370

Query 365 GGAGCTTCTGTAGATTTAGCTATTTTTTCTCTTCATTTAGCAGGAATTTCTTCTATTTTA 424

||||||||||||||||||||||||||||||||||||||||||||||||||||||||||||

Sbjct 371 GGAGCTTCTGTAGATTTAGCTATTTTTTCTCTTCATTTAGCAGGAATTTCTTCTATTTTA 430

Query 425 GGAGCAGTAAATTTTATTACAACTGTAATTAATATACGATCAACTGGAATTACACTTGAT 484

|||||||||||||||||||||||||||||||||||||||||| |||||||||||||||||

Sbjct 431 GGAGCAGTAAATTTTATTACAACTGTAATTAATATACGATCAGCTGGAATTACACTTGAT 490

Query 485 CGTTTACCTTTATTTGTCTGATCTGTAATTATTACAGCTATTTTATTACTTCTTTCATTA 544

|| |||||||||||||| ||||| ||| ||||||||||||||||||||||||||||||||

Sbjct 491 CGCTTACCTTTATTTGTTTGATCAGTAGTTATTACAGCTATTTTATTACTTCTTTCATTA 550

Query 545 CCAGTTTTAGCAGGAGCTATTACTATATTATTAACTGATCGAAATTTTAATACATCATTT 604

||||||||||||||||||||||||||||||||||||||||||||||||||||||||||||

Sbjct 551 CCAGTTTTAGCAGGAGCTATTACTATATTATTAACTGATCGAAATTTTAATACATCATTT 610

Query 605 TTTGACCCAATTGGAGGAGGTGACCCTATTCTTTATCAACATCTATTT 652

||||| || |||||||||||||||||||||||||||||||||||||||

Sbjct 611 TTTGATCCTATTGGAGGAGGTGACCCTATTCTTTATCAACATCTATTT 658

>Aedes daitensis mitochondrial COI gene for cytochrome oxidase subunit 1, partial cds, isolate: 052MDA2001

Sequence ID: AB738096.1 Length: 658

>Aedes daitensis mitochondrial COI gene for cytochrome oxidase subunit 1, partial cds, isolate: 052-2MDA2001

Sequence ID: AB738097.1 Length: 658

Range 1: 13 to 658

Score:1022 bits(553), Expect:0.0,

Identities:617/648(95%), Gaps:3/648(0%), Strand: Plus/Plus

Query 6 TATTTTTGGAGTATGATCGGGGA-AGTCGGAACTTCATTAAGAGTTTTAATTCGAACTGA 64

|||||||||||||||||| || | ||| ||||||||||||||||||||||||||||||||

Sbjct 13 TATTTTTGGAGTATGATCTGGAATAGTTGGAACTTCATTAAGAGTTTTAATTCGAACTGA 72

Query 65 ACTTAGTCACCCTGGAATATTTATCGGAAATGATCAAATTTATAATGTAATTCGTTACTA 124

||||||||| |||||||||||||| ||||||||||||||||||||||||||| ||||||

Sbjct 73 ACTTAGTCATCCTGGAATATTTATTGGAAATGATCAAATTTATAATGTAATT-GTTACT- 130

Query 125 GCTCATGCATTTATTATAAttttttttATAGTAATACCTATTATAATTGGAGGATTTGGA 184

||||||||||||||||||||||||||||||||||||||||||||||||||||||||||||

Sbjct 131 GCTCATGCATTTATTATAATTTTTTTTATAGTAATACCTATTATAATTGGAGGATTTGGA 190

Query 185 AATTGGTTAGTCCCATTAATATTAGGTGCTCCTGATATAGCTTTCCCTCGAATAAATAAT 244

|| || ||||||||||||||||||||||| |||||||||||||| |||||||||||||||

Sbjct 191 AACTGATTAGTCCCATTAATATTAGGTGCCCCTGATATAGCTTTTCCTCGAATAAATAAT 250

Query 245 ATAAGTTTTTGAATACTTCCTCCCTCTTTAACTCTTTTAATTTCTAGATCTATAGTAGAA 304

||||||||||||||||||||||| ||||||||||||||| ||||||||||||||||||||

Sbjct 251 ATAAGTTTTTGAATACTTCCTCCTTCTTTAACTCTTTTACTTTCTAGATCTATAGTAGAA 310

Query 305 AATGGAGCAGGAACAGGTTGAACTGTATACCCTCCTCTTTCTTCTGGAACTGCTCATGCA 364

||||| |||||||||||||||||||| || |||||||||||||||||||||||||| ||

Sbjct 311 AATGGTGCAGGAACAGGTTGAACTGTTTATCCTCCTCTTTCTTCTGGAACTGCTCACGCT 370

Query 365 GGAGCTTCTGTAGATTTAGCTATTTTTTCTCTTCATTTAGCAGGAATTTCTTCTATTTTA 424

|||||||||||||||||||||||||||||||||||||||||||| |||||||||||||||

Sbjct 371 GGAGCTTCTGTAGATTTAGCTATTTTTTCTCTTCATTTAGCAGGTATTTCTTCTATTTTA 430

Query 425 GGAGCAGTAAATTTTATTACAACTGTAATTAATATACGATCAACTGGAATTACACTTGAT 484

|||||||||||||||||||||||||||||||||||||||||| |||||||||| ||||||

Sbjct 431 GGAGCAGTAAATTTTATTACAACTGTAATTAATATACGATCAGCTGGAATTACTCTTGAT 490

Query 485 CGTTTACCTTTATTTGTCTGATCTGTAATTATTACAGCTATTTTATTACTTCTTTCATTA 544

||||||||||||||||| ||||| ||| | ||||||||||||||||||||||||||||||

Sbjct 491 CGTTTACCTTTATTTGTTTGATCAGTAGTAATTACAGCTATTTTATTACTTCTTTCATTA 550

Query 545 CCAGTTTTAGCAGGAGCTATTACTATATTATTAACTGATCGAAATTTTAATACATCATTT 604

|| |||||||||||||||||||||||||||||||||||||||||||||||||||||||||

Sbjct 551 CCTGTTTTAGCAGGAGCTATTACTATATTATTAACTGATCGAAATTTTAATACATCATTT 610

Query 605 TTTGACCCAATTGGAGGAGGTGACCCTATTCTTTATCAACATCTATTT 652

||||| |||||||||||||| || |||||||||||||||||| |||||

Sbjct 611 TTTGATCCAATTGGAGGAGGAGATCCTATTCTTTATCAACATTTATTT 658

>Culicidae sp. sc_02090 cytochrome oxidase subunit 1 (COI) gene, partial cds; mitochondrial

Sequence ID: KX052479.1 Length: 658

>Culicidae sp. sc_02111 cytochrome oxidase subunit 1 (COI) gene, partial cds; mitochondrial

Sequence ID: KX052483.1 Length: 658

Range 1: 13 to 658

Score:961 bits(520), Expect:0.0,

Identities:606/648(94%), Gaps:3/648(0%), Strand: Plus/Plus

Query 6 TATTTTTGGAGTATGATCGGGGA-AGTCGGAACTTCATTAAGAGTTTTAATTCGAACTGA 64

|||||||||||| ||||| || | ||| |||||||| |||||||||||||||||||||||

Sbjct 13 TATTTTTGGAGTTTGATCTGGAATAGTAGGAACTTCTTTAAGAGTTTTAATTCGAACTGA 72

Query 65 ACTTAGTCACCCTGGAATATTTATCGGAAATGATCAAATTTATAATGTAATTCGTTACTA 124

||||||||||||||| |||||||| |||||||| |||||||||||||||||| ||||||

Sbjct 73 ACTTAGTCACCCTGGTATATTTATTGGAAATGACCAAATTTATAATGTAATT-GTTACT- 130

Query 125 GCTCATGCATTTATTATAAttttttttATAGTAATACCTATTATAATTGGAGGATTTGGA 184

||||||||||||||||||||||||||||||||||||||||||||||||||||||||||||

Sbjct 131 GCTCATGCATTTATTATAATTTTTTTTATAGTAATACCTATTATAATTGGAGGATTTGGA 190

Query 185 AATTGGTTAGTCCCATTAATATTAGGTGCTCCTGATATAGCTTTCCCTCGAATAAATAAT 244

||||| ||||| || ||||||||||| || |||||||||||||| |||||||||||||||

Sbjct 191 AATTGATTAGTTCCTTTAATATTAGGAGCCCCTGATATAGCTTTTCCTCGAATAAATAAT 250

Query 245 ATAAGTTTTTGAATACTTCCTCCCTCTTTAACTCTTTTAATTTCTAGATCTATAGTAGAA 304

||||||||||||||||||||||| ||||||||||||||| ||||||||||||||||||||

Sbjct 251 ATAAGTTTTTGAATACTTCCTCCTTCTTTAACTCTTTTACTTTCTAGATCTATAGTAGAA 310

Query 305 AATGGAGCAGGAACAGGTTGAACTGTATACCCTCCTCTTTCTTCTGGAACTGCTCATGCA 364

|||||||||||||| ||||||||||| || |||||||||||||||||||| ||||||||

Sbjct 311 AATGGAGCAGGAACTGGTTGAACTGTTTATCCTCCTCTTTCTTCTGGAACCGCTCATGCT 370

Query 365 GGAGCTTCTGTAGATTTAGCTATTTTTTCTCTTCATTTAGCAGGAATTTCTTCTATTTTA 424

|||||||| || |||||||||||||||||| | ||||||||||| ||||| |||||||||

Sbjct 371 GGAGCTTCAGTTGATTTAGCTATTTTTTCTTTACATTTAGCAGGTATTTCATCTATTTTA 430

Query 425 GGAGCAGTAAATTTTATTACAACTGTAATTAATATACGATCAACTGGAATTACACTTGAT 484

||||||||||||||||||||||||||||||||||| |||||| |||||||||||||||||

Sbjct 431 GGAGCAGTAAATTTTATTACAACTGTAATTAATATGCGATCAGCTGGAATTACACTTGAT 490

Query 485 CGTTTACCTTTATTTGTCTGATCTGTAATTATTACAGCTATTTTATTACTTCTTTCATTA 544

|| |||||||||||||| ||||| ||| | |||||||| |||||||||||||||||||||

Sbjct 491 CGATTACCTTTATTTGTTTGATCAGTATTAATTACAGCAATTTTATTACTTCTTTCATTA 550

Query 545 CCAGTTTTAGCAGGAGCTATTACTATATTATTAACTGATCGAAATTTTAATACATCATTT 604

|| |||||||||||||| || |||||||||||||||||||||||||||||||||||||||

Sbjct 551 CCTGTTTTAGCAGGAGCAATCACTATATTATTAACTGATCGAAATTTTAATACATCATTT 610

Query 605 TTTGACCCAATTGGAGGAGGTGACCCTATTCTTTATCAACATCTATTT 652

||||||||||||||||||||||||||||||||||| ||||||||||||

Sbjct 611 TTTGACCCAATTGGAGGAGGTGACCCTATTCTTTACCAACATCTATTT 658

>Toxorhynchites amboinensis voucher ICBRIRI-0002 cytochrome c oxidase subunit I (COI) gene, partial cds; mitochondrial

Sequence ID: JQ235743.1 Length: 669

Range 1: 3 to 662

Score:959 bits(519), Expect:0.0,

Identities:615/662(93%), Gaps:3/662(0%), Strand: Plus/Minus

Query 6 TATTTTTGGAGTATGATCGGGGA-AGTCGGAACTTCATTAAGAGTTTTAATTCGAACTGA 64

|||||||||||| ||||| || | ||| |||||||| |||||||||||||||||||||||

Sbjct 662 TATTTTTGGAGTTTGATCTGGAATAGTAGGAACTTCTTTAAGAGTTTTAATTCGAACTGA 603

Query 65 ACTTAGTCACCCTGGAATATTTATCGGAAATGATCAAATTTATAATGTAATTCGTTACTA 124

||||||||||||||| |||||||| |||||||| |||||||||||||||||| ||||||

Sbjct 602 ACTTAGTCACCCTGGTATATTTATTGGAAATGACCAAATTTATAATGTAATT-GTTACT- 545

Query 125 GCTCATGCATTTATTATAAttttttttATAGTAATACCTATTATAATTGGAGGATTTGGA 184

||||||||||||||||||||||||||||||||||||||||||||||||||||||||||||

Sbjct 544 GCTCATGCATTTATTATAATTTTTTTTATAGTAATACCTATTATAATTGGAGGATTTGGA 485

Query 185 AATTGGTTAGTCCCATTAATATTAGGTGCTCCTGATATAGCTTTCCCTCGAATAAATAAT 244

||||| ||||| || ||||||||||| || |||||||||||||| |||||||||||||||

Sbjct 484 AATTGATTAGTTCCTTTAATATTAGGAGCCCCTGATATAGCTTTTCCTCGAATAAATAAT 425

Query 245 ATAAGTTTTTGAATACTTCCTCCCTCTTTAACTCTTTTAATTTCTAGATCTATAGTAGAA 304

||||||||||||||||||||||| ||||||||||||||| ||||||||||||||||||||

Sbjct 424 ATAAGTTTTTGAATACTTCCTCCTTCTTTAACTCTTTTACTTTCTAGATCTATAGTAGAA 365

Query 305 AATGGAGCAGGAACAGGTTGAACTGTATACCCTCCTCTTTCTTCTGGAACTGCTCATGCA 364

||||||| |||||| ||||||||||| || |||||||||||||||||||| ||||||||

Sbjct 364 AATGGAGTAGGAACTGGTTGAACTGTTTATCCTCCTCTTTCTTCTGGAACCGCTCATGCT 305

Query 365 GGAGCTTCTGTAGATTTAGCTATTTTTTCTCTTCATTTAGCAGGAATTTCTTCTATTTTA 424

|||||||| || |||||||||||||||||| | ||||||||||| ||||| |||||||||

Sbjct 304 GGAGCTTCAGTTGATTTAGCTATTTTTTCTTTACATTTAGCAGGTATTTCATCTATTTTA 245

Query 425 GGAGCAGTAAATTTTATTACAACTGTAATTAATATACGATCAACTGGAATTACACTTGAT 484

|||||||||||||||||||||||||||||||||||||||||| |||||||||| ||||||

Sbjct 244 GGAGCAGTAAATTTTATTACAACTGTAATTAATATACGATCAGCTGGAATTACGCTTGAT 185

Query 485 CGTTTACCTTTATTTGTCTGATCTGTAATTATTACAGCTATTTTATTACTTCTTTCATTA 544

|| |||||||||||||| ||||| ||| | |||||||| |||||||||||||||||||||

Sbjct 184 CGATTACCTTTATTTGTTTGATCAGTAGTAATTACAGCAATTTTATTACTTCTTTCATTA 125

Query 545 CCAGTTTTAGCAGGAGCTATTACTATATTATTAACTGATCGAAATTTTAATACATCATTT 604

|| |||||||||||||| || ||||| |||||||||||||||||||||||||||||||||

Sbjct 124 CCTGTTTTAGCAGGAGCAATCACTATGTTATTAACTGATCGAAATTTTAATACATCATTT 65

Query 605 TTTGACCCAATTGGAGGAGGTGACCCTATTCTTTATCAACATCTATTTTGATTTTTTGGA 664

||||| ||||||||||||||||| ||||||||||| |||||||||||||||||||||||

Sbjct 64 TTTGATCCAATTGGAGGAGGTGATCCTATTCTTTACCAACATCTATTTTGATTTTTTGGT 5

Query 665 CA 666

||

Sbjct 4 CA 3

>Culicidae sp. sc_01327 cytochrome oxidase subunit 1 (COI) gene, partial cds; mitochondrial

Sequence ID: KX052485.1 Length: 658

Range 1: 13 to 658

Score:955 bits(517), Expect:0.0,

Identities:605/648(93%), Gaps:3/648(0%), Strand: Plus/Plus

Query 6 TATTTTTGGAGTATGATCGGGGA-AGTCGGAACTTCATTAAGAGTTTTAATTCGAACTGA 64

|||||||||||| ||||| || | ||| |||||||| |||||||||||||||||||||||

Sbjct 13 TATTTTTGGAGTTTGATCTGGAATAGTAGGAACTTCTTTAAGAGTTTTAATTCGAACTGA 72

Query 65 ACTTAGTCACCCTGGAATATTTATCGGAAATGATCAAATTTATAATGTAATTCGTTACTA 124

||||||||||||||| |||||||| |||||||| |||||||||||||||||| ||||||

Sbjct 73 ACTTAGTCACCCTGGTATATTTATTGGAAATGACCAAATTTATAATGTAATT-GTTACT- 130

Query 125 GCTCATGCATTTATTATAAttttttttATAGTAATACCTATTATAATTGGAGGATTTGGA 184

||||||||||||||||||||||||||||||||||||||||||||||||||||||||||||

Sbjct 131 GCTCATGCATTTATTATAATTTTTTTTATAGTAATACCTATTATAATTGGAGGATTTGGA 190

Query 185 AATTGGTTAGTCCCATTAATATTAGGTGCTCCTGATATAGCTTTCCCTCGAATAAATAAT 244

||||| ||||| || ||||||||||| || |||||||||||||| |||||||||||||||

Sbjct 191 AATTGATTAGTTCCTTTAATATTAGGAGCCCCTGATATAGCTTTTCCTCGAATAAATAAT 250

Query 245 ATAAGTTTTTGAATACTTCCTCCCTCTTTAACTCTTTTAATTTCTAGATCTATAGTAGAA 304

||||||||||||||||||||||| ||||||||||||||| ||||||||||||||||||||

Sbjct 251 ATAAGTTTTTGAATACTTCCTCCTTCTTTAACTCTTTTACTTTCTAGATCTATAGTAGAA 310

Query 305 AATGGAGCAGGAACAGGTTGAACTGTATACCCTCCTCTTTCTTCTGGAACTGCTCATGCA 364

|||||||||||||| ||||||||||| || |||||||||||||||||||| ||||||||

Sbjct 311 AATGGAGCAGGAACTGGTTGAACTGTTTATCCTCCTCTTTCTTCTGGAACCGCTCATGCT 370

Query 365 GGAGCTTCTGTAGATTTAGCTATTTTTTCTCTTCATTTAGCAGGAATTTCTTCTATTTTA 424

|||||||| || |||||||||||||||||| | ||||||||||| ||||| |||||||||

Sbjct 371 GGAGCTTCAGTTGATTTAGCTATTTTTTCTTTACATTTAGCAGGTATTTCATCTATTTTA 430

Query 425 GGAGCAGTAAATTTTATTACAACTGTAATTAATATACGATCAACTGGAATTACACTTGAT 484

||||||||||||||||||||||||||||||||||| |||||| |||||||||||||||||

Sbjct 431 GGAGCAGTAAATTTTATTACAACTGTAATTAATATGCGATCAGCTGGAATTACACTTGAT 490

Query 485 CGTTTACCTTTATTTGTCTGATCTGTAATTATTACAGCTATTTTATTACTTCTTTCATTA 544

|| |||||||||||||| ||||| ||| | |||||||| |||||||||||||||||||||

Sbjct 491 CGATTACCTTTATTTGTTTGATCAGTATTAATTACAGCAATTTTATTACTTCTTTCATTA 550

Query 545 CCAGTTTTAGCAGGAGCTATTACTATATTATTAACTGATCGAAATTTTAATACATCATTT 604

|| |||||||||||||| || ||||| |||||||||||||||||||||||||||||||||

Sbjct 551 CCTGTTTTAGCAGGAGCAATCACTATGTTATTAACTGATCGAAATTTTAATACATCATTT 610

Query 605 TTTGACCCAATTGGAGGAGGTGACCCTATTCTTTATCAACATCTATTT 652

||||||||||||||||||||||||||||||||||| ||||||||||||

Sbjct 611 TTTGACCCAATTGGAGGAGGTGACCCTATTCTTTACCAACATCTATTT 658

>Culicidae sp. sc_05594 cytochrome oxidase subunit 1 (COI) gene, partial cds; mitochondrial

Sequence ID: KX052482.1 Length: 658

>Culicidae sp. sc_01319 cytochrome oxidase subunit 1 (COI) gene, partial cds; mitochondrial

Sequence ID: KX052487.1 Length: 658

>Culicidae sp. sc_07008 cytochrome oxidase subunit 1 (COI) gene, partial cds; mitochondrial

Sequence ID: KX052488.1 Length: 658

>Culicidae sp. sc_02857 cytochrome oxidase subunit 1 (COI) gene, partial cds; mitochondrial

Sequence ID: KX052492.1 Length: 658

>Culicidae sp. sc_02703 cytochrome oxidase subunit 1 (COI) gene, partial cds; mitochondrial

Sequence ID: KX052494.1 Length: 658

>Culicidae sp. sc_02702 cytochrome oxidase subunit 1 (COI) gene, partial cds; mitochondrial

Sequence ID: KX052495.1 Length: 658

>Culicidae sp. sc_02121 cytochrome oxidase subunit 1 (COI) gene, partial cds; mitochondrial

Sequence ID: KX052498.1 Length: 658

>Culicidae sp. sc_07177 cytochrome oxidase subunit 1 (COI) gene, partial cds; mitochondrial

Sequence ID: KX052500.1 Length: 658

>Culicidae sp. sc_05280 cytochrome oxidase subunit 1 (COI) gene, partial cds; mitochondrial

Sequence ID: KX052502.1 Length: 658

>Culicidae sp. sc_07178 cytochrome oxidase subunit 1 (COI) gene, partial cds; mitochondrial

Sequence ID: KX052509.1 Length: 658

>Culicidae sp. sc_07844 cytochrome oxidase subunit 1 (COI) gene, partial cds; mitochondrial

Sequence ID: KX052510.1 Length: 658

Range 1: 13 to 658

Score:955 bits(517), Expect:0.0,

Identities:605/648(93%), Gaps:3/648(0%), Strand: Plus/Plus

Query 6 TATTTTTGGAGTATGATCGGGGA-AGTCGGAACTTCATTAAGAGTTTTAATTCGAACTGA 64

|||||||||||| ||||| || | ||| |||||||| |||||||||||||||||||||||

Sbjct 13 TATTTTTGGAGTTTGATCTGGAATAGTAGGAACTTCTTTAAGAGTTTTAATTCGAACTGA 72

Query 65 ACTTAGTCACCCTGGAATATTTATCGGAAATGATCAAATTTATAATGTAATTCGTTACTA 124

||||||||||||||| |||||||| |||||||| |||||||||||||||||| ||||||

Sbjct 73 ACTTAGTCACCCTGGTATATTTATTGGAAATGACCAAATTTATAATGTAATT-GTTACT- 130

Query 125 GCTCATGCATTTATTATAAttttttttATAGTAATACCTATTATAATTGGAGGATTTGGA 184

||||||||||||||||||||||||||||||||||||||||||||||||||||||||||||

Sbjct 131 GCTCATGCATTTATTATAATTTTTTTTATAGTAATACCTATTATAATTGGAGGATTTGGA 190

Query 185 AATTGGTTAGTCCCATTAATATTAGGTGCTCCTGATATAGCTTTCCCTCGAATAAATAAT 244

||||| ||||| || ||||||||||| || |||||||||||||| |||||||||||||||

Sbjct 191 AATTGATTAGTTCCTTTAATATTAGGAGCCCCTGATATAGCTTTTCCTCGAATAAATAAT 250

Query 245 ATAAGTTTTTGAATACTTCCTCCCTCTTTAACTCTTTTAATTTCTAGATCTATAGTAGAA 304

||||||||||||||||||||||| ||||||||||||||| ||||||||||||||||||||

Sbjct 251 ATAAGTTTTTGAATACTTCCTCCTTCTTTAACTCTTTTACTTTCTAGATCTATAGTAGAA 310

Query 305 AATGGAGCAGGAACAGGTTGAACTGTATACCCTCCTCTTTCTTCTGGAACTGCTCATGCA 364

|||||||||||||| ||||||||||| || |||||||||||||||||||| ||||||||

Sbjct 311 AATGGAGCAGGAACTGGTTGAACTGTTTATCCTCCTCTTTCTTCTGGAACCGCTCATGCT 370

Query 365 GGAGCTTCTGTAGATTTAGCTATTTTTTCTCTTCATTTAGCAGGAATTTCTTCTATTTTA 424

|||||||| || |||||||||||||||||| | ||||||||||| ||||| |||||||||

Sbjct 371 GGAGCTTCAGTTGATTTAGCTATTTTTTCTTTACATTTAGCAGGTATTTCATCTATTTTA 430

Query 425 GGAGCAGTAAATTTTATTACAACTGTAATTAATATACGATCAACTGGAATTACACTTGAT 484

||||||||||||||||||||||||||||||||||| |||||| |||||||||||||||||

Sbjct 431 GGAGCAGTAAATTTTATTACAACTGTAATTAATATGCGATCAGCTGGAATTACACTTGAT 490

Query 485 CGTTTACCTTTATTTGTCTGATCTGTAATTATTACAGCTATTTTATTACTTCTTTCATTA 544

|| |||||||||||||| ||||| ||| | |||||||| |||||||||||||||||||||

Sbjct 491 CGATTACCTTTATTTGTTTGATCAGTATTAATTACAGCAATTTTATTACTTCTTTCATTA 550

Query 545 CCAGTTTTAGCAGGAGCTATTACTATATTATTAACTGATCGAAATTTTAATACATCATTT 604

|| |||||||||||||| || ||||| |||||||||||||||||||||||||||||||||

Sbjct 551 CCTGTTTTAGCAGGAGCAATCACTATGTTATTAACTGATCGAAATTTTAATACATCATTT 610

Query 605 TTTGACCCAATTGGAGGAGGTGACCCTATTCTTTATCAACATCTATTT 652

||||||||||||||||||||||||||||||||||| ||||||||||||

Sbjct 611 TTTGACCCAATTGGAGGAGGTGACCCTATTCTTTACCAACATCTATTT 658

>Culicidae sp. sc_02856 cytochrome oxidase subunit 1 (COI) gene, partial cds; mitochondrial

Sequence ID: KX052491.1 Length: 653

>Culicidae sp. sc_05279 cytochrome oxidase subunit 1 (COI) gene, partial cds; mitochondrial

Sequence ID: KX052501.1 Length: 653

>Culicidae sp. sc_05290 cytochrome oxidase subunit 1 (COI) gene, partial cds; mitochondrial

Sequence ID: KX052503.1 Length: 653

>Culicidae sp. sc_06471 cytochrome oxidase subunit 1 (COI) gene, partial cds; mitochondrial

Sequence ID: KX052505.1 Length: 653

Range 1: 13 to 653

Score:946 bits(512), Expect:0.0,

Identities:600/643(93%), Gaps:3/643(0%), Strand: Plus/Plus

Query 6 TATTTTTGGAGTATGATCGGGGA-AGTCGGAACTTCATTAAGAGTTTTAATTCGAACTGA 64

|||||||||||| ||||| || | ||| |||||||| |||||||||||||||||||||||

Sbjct 13 TATTTTTGGAGTTTGATCTGGAATAGTAGGAACTTCTTTAAGAGTTTTAATTCGAACTGA 72

Query 65 ACTTAGTCACCCTGGAATATTTATCGGAAATGATCAAATTTATAATGTAATTCGTTACTA 124

||||||||||||||| |||||||| |||||||| |||||||||||||||||| ||||||

Sbjct 73 ACTTAGTCACCCTGGTATATTTATTGGAAATGACCAAATTTATAATGTAATT-GTTACT- 130

Query 125 GCTCATGCATTTATTATAAttttttttATAGTAATACCTATTATAATTGGAGGATTTGGA 184

||||||||||||||||||||||||||||||||||||||||||||||||||||||||||||

Sbjct 131 GCTCATGCATTTATTATAATTTTTTTTATAGTAATACCTATTATAATTGGAGGATTTGGA 190

Query 185 AATTGGTTAGTCCCATTAATATTAGGTGCTCCTGATATAGCTTTCCCTCGAATAAATAAT 244

||||| ||||| || ||||||||||| || |||||||||||||| |||||||||||||||

Sbjct 191 AATTGATTAGTTCCTTTAATATTAGGAGCCCCTGATATAGCTTTTCCTCGAATAAATAAT 250

Query 245 ATAAGTTTTTGAATACTTCCTCCCTCTTTAACTCTTTTAATTTCTAGATCTATAGTAGAA 304

||||||||||||||||||||||| ||||||||||||||| ||||||||||||||||||||

Sbjct 251 ATAAGTTTTTGAATACTTCCTCCTTCTTTAACTCTTTTACTTTCTAGATCTATAGTAGAA 310

Query 305 AATGGAGCAGGAACAGGTTGAACTGTATACCCTCCTCTTTCTTCTGGAACTGCTCATGCA 364

|||||||||||||| ||||||||||| || |||||||||||||||||||| ||||||||

Sbjct 311 AATGGAGCAGGAACTGGTTGAACTGTTTATCCTCCTCTTTCTTCTGGAACCGCTCATGCT 370

Query 365 GGAGCTTCTGTAGATTTAGCTATTTTTTCTCTTCATTTAGCAGGAATTTCTTCTATTTTA 424

|||||||| || |||||||||||||||||| | ||||||||||| ||||| |||||||||

Sbjct 371 GGAGCTTCAGTTGATTTAGCTATTTTTTCTTTACATTTAGCAGGTATTTCATCTATTTTA 430

Query 425 GGAGCAGTAAATTTTATTACAACTGTAATTAATATACGATCAACTGGAATTACACTTGAT 484

||||||||||||||||||||||||||||||||||| |||||| |||||||||||||||||

Sbjct 431 GGAGCAGTAAATTTTATTACAACTGTAATTAATATGCGATCAGCTGGAATTACACTTGAT 490

Query 485 CGTTTACCTTTATTTGTCTGATCTGTAATTATTACAGCTATTTTATTACTTCTTTCATTA 544

|| |||||||||||||| ||||| ||| | |||||||| |||||||||||||||||||||

Sbjct 491 CGATTACCTTTATTTGTTTGATCAGTATTAATTACAGCAATTTTATTACTTCTTTCATTA 550

Query 545 CCAGTTTTAGCAGGAGCTATTACTATATTATTAACTGATCGAAATTTTAATACATCATTT 604

|| |||||||||||||| || ||||| |||||||||||||||||||||||||||||||||

Sbjct 551 CCTGTTTTAGCAGGAGCAATCACTATGTTATTAACTGATCGAAATTTTAATACATCATTT 610

Query 605 TTTGACCCAATTGGAGGAGGTGACCCTATTCTTTATCAACATC 647

||||||||||||||||||||||||||||||||||| |||||||

Sbjct 611 TTTGACCCAATTGGAGGAGGTGACCCTATTCTTTACCAACATC 653

>Culicidae sp. sc_05401 cytochrome oxidase subunit 1 (COI) gene, partial cds; mitochondrial

Sequence ID: KX052486.1 Length: 651

Range 1: 13 to 651

Score:942 bits(510), Expect:0.0,

Identities:598/641(93%), Gaps:3/641(0%), Strand: Plus/Plus

Query 6 TATTTTTGGAGTATGATCGGGGA-AGTCGGAACTTCATTAAGAGTTTTAATTCGAACTGA 64

|||||||||||| ||||| || | ||| |||||||| |||||||||||||||||||||||

Sbjct 13 TATTTTTGGAGTTTGATCTGGAATAGTAGGAACTTCTTTAAGAGTTTTAATTCGAACTGA 72

Query 65 ACTTAGTCACCCTGGAATATTTATCGGAAATGATCAAATTTATAATGTAATTCGTTACTA 124

||||||||||||||| |||||||| |||||||| |||||||||||||||||| ||||||

Sbjct 73 ACTTAGTCACCCTGGTATATTTATTGGAAATGACCAAATTTATAATGTAATT-GTTACT- 130

Query 125 GCTCATGCATTTATTATAAttttttttATAGTAATACCTATTATAATTGGAGGATTTGGA 184

||||||||||||||||||||||||||||||||||||||||||||||||||||||||||||

Sbjct 131 GCTCATGCATTTATTATAATTTTTTTTATAGTAATACCTATTATAATTGGAGGATTTGGA 190

Query 185 AATTGGTTAGTCCCATTAATATTAGGTGCTCCTGATATAGCTTTCCCTCGAATAAATAAT 244

||||| ||||| || ||||||||||| || |||||||||||||| |||||||||||||||

Sbjct 191 AATTGATTAGTTCCTTTAATATTAGGAGCCCCTGATATAGCTTTTCCTCGAATAAATAAT 250

Query 245 ATAAGTTTTTGAATACTTCCTCCCTCTTTAACTCTTTTAATTTCTAGATCTATAGTAGAA 304

||||||||||||||||||||||| ||||||||||||||| ||||||||||||||||||||

Sbjct 251 ATAAGTTTTTGAATACTTCCTCCTTCTTTAACTCTTTTACTTTCTAGATCTATAGTAGAA 310

Query 305 AATGGAGCAGGAACAGGTTGAACTGTATACCCTCCTCTTTCTTCTGGAACTGCTCATGCA 364

|||||||||||||| ||||||||||| || |||||||||||||||||||| ||||||||

Sbjct 311 AATGGAGCAGGAACTGGTTGAACTGTTTATCCTCCTCTTTCTTCTGGAACCGCTCATGCT 370

Query 365 GGAGCTTCTGTAGATTTAGCTATTTTTTCTCTTCATTTAGCAGGAATTTCTTCTATTTTA 424

|||||||| || |||||||||||||||||| | ||||||||||| ||||| |||||||||

Sbjct 371 GGAGCTTCAGTTGATTTAGCTATTTTTTCTTTACATTTAGCAGGTATTTCATCTATTTTA 430

Query 425 GGAGCAGTAAATTTTATTACAACTGTAATTAATATACGATCAACTGGAATTACACTTGAT 484

||||||||||||||||||||||||||||||||||| |||||| |||||||||||||||||

Sbjct 431 GGAGCAGTAAATTTTATTACAACTGTAATTAATATGCGATCAGCTGGAATTACACTTGAT 490

Query 485 CGTTTACCTTTATTTGTCTGATCTGTAATTATTACAGCTATTTTATTACTTCTTTCATTA 544

|| |||||||||||||| ||||| ||| | |||||||| |||||||||||||||||||||

Sbjct 491 CGATTACCTTTATTTGTTTGATCAGTATTAATTACAGCAATTTTATTACTTCTTTCATTA 550

Query 545 CCAGTTTTAGCAGGAGCTATTACTATATTATTAACTGATCGAAATTTTAATACATCATTT 604

|| |||||||||||||| || ||||| |||||||||||||||||||||||||||||||||

Sbjct 551 CCTGTTTTAGCAGGAGCAATCACTATGTTATTAACTGATCGAAATTTTAATACATCATTT 610

Query 605 TTTGACCCAATTGGAGGAGGTGACCCTATTCTTTATCAACA 645

||||||||||||||||||||||||||||||||||| |||||

Sbjct 611 TTTGACCCAATTGGAGGAGGTGACCCTATTCTTTACCAACA 651

>Culicidae sp. sc_06676 cytochrome oxidase subunit 1 (COI) gene, partial cds; mitochondrial

Sequence ID: KX052506.1 Length: 653

Range 1: 13 to 653

Score:941 bits(509), Expect:0.0,

Identities:599/643(93%), Gaps:3/643(0%), Strand: Plus/Plus

Query 6 TATTTTTGGAGTATGATCGGGGA-AGTCGGAACTTCATTAAGAGTTTTAATTCGAACTGA 64

|||||||||||| ||||| || | ||| |||||||| |||||||||||||||||||||||

Sbjct 13 TATTTTTGGAGTTTGATCTGGAATAGTAGGAACTTCTTTAAGAGTTTTAATTCGAACTGA 72

Query 65 ACTTAGTCACCCTGGAATATTTATCGGAAATGATCAAATTTATAATGTAATTCGTTACTA 124

||||||||||||||| |||||||| |||||||| |||||||||||||||||| ||||||

Sbjct 73 ACTTAGTCACCCTGGTATATTTATTGGAAATGACCAAATTTATAATGTAATT-GTTACT- 130

Query 125 GCTCATGCATTTATTATAAttttttttATAGTAATACCTATTATAATTGGAGGATTTGGA 184

||||||||||||||||||||||||||||||||||||||||||||||||||||||||||||

Sbjct 131 GCTCATGCATTTATTATAATTTTTTTTATAGTAATACCTATTATAATTGGAGGATTTGGA 190

Query 185 AATTGGTTAGTCCCATTAATATTAGGTGCTCCTGATATAGCTTTCCCTCGAATAAATAAT 244

||||| ||||| || ||||||||||| || |||||||||||||| |||||||||||||||

Sbjct 191 AATTGATTAGTTCCTTTAATATTAGGAGCCCCTGATATAGCTTTTCCTCGAATAAATAAT 250

Query 245 ATAAGTTTTTGAATACTTCCTCCCTCTTTAACTCTTTTAATTTCTAGATCTATAGTAGAA 304

||||||||||||||||||||||| ||||||||||||||| ||||||||||||||||||||

Sbjct 251 ATAAGTTTTTGAATACTTCCTCCTTCTTTAACTCTTTTACTTTCTAGATCTATAGTAGAA 310

Query 305 AATGGAGCAGGAACAGGTTGAACTGTATACCCTCCTCTTTCTTCTGGAACTGCTCATGCA 364

|||||||||||||| ||||||||||| || |||||||||||||||||||| ||||||||

Sbjct 311 AATGGAGCAGGAACTGGTTGAACTGTTTATCCTCCTCTTTCTTCTGGAACCGCTCATGCT 370

Query 365 GGAGCTTCTGTAGATTTAGCTATTTTTTCTCTTCATTTAGCAGGAATTTCTTCTATTTTA 424

|||||||| || |||||||||||||||||| | ||||||||||| ||||| |||||||||

Sbjct 371 GGAGCTTCAGTTGATTTAGCTATTTTTTCTTTACATTTAGCAGGTATTTCATCTATTTTA 430

Query 425 GGAGCAGTAAATTTTATTACAACTGTAATTAATATACGATCAACTGGAATTACACTTGAT 484

||||||||||||||||||||||||||||||||||| |||||| |||||||||||||||||

Sbjct 431 GGAGCAGTAAATTTTATTACAACTGTAATTAATATGCGATCAGCTGGAATTACACTTGAT 490

Query 485 CGTTTACCTTTATTTGTCTGATCTGTAATTATTACAGCTATTTTATTACTTCTTTCATTA 544

|| |||||||||||||| ||||| ||| | |||||||| ||||||||||||||||||||

Sbjct 491 CGATTACCTTTATTTGTTTGATCAGTATTAATTACAGCAATTTTATTACTTCTTTCATTG 550

Query 545 CCAGTTTTAGCAGGAGCTATTACTATATTATTAACTGATCGAAATTTTAATACATCATTT 604

|| |||||||||||||| || ||||| |||||||||||||||||||||||||||||||||

Sbjct 551 CCTGTTTTAGCAGGAGCAATCACTATGTTATTAACTGATCGAAATTTTAATACATCATTT 610

Query 605 TTTGACCCAATTGGAGGAGGTGACCCTATTCTTTATCAACATC 647

||||||||||||||||||||||||||||||||||| |||||||

Sbjct 611 TTTGACCCAATTGGAGGAGGTGACCCTATTCTTTACCAACATC 653

>Culicidae sp. sc_00010 cytochrome oxidase subunit 1 (COI) gene, partial cds; mitochondrial

Sequence ID: KX052496.1 Length: 647

Range 1: 13 to 645

Score:933 bits(505), Expect:0.0,

Identities:592/635(93%), Gaps:3/635(0%), Strand: Plus/Plus

Query 6 TATTTTTGGAGTATGATCGGGGA-AGTCGGAACTTCATTAAGAGTTTTAATTCGAACTGA 64

||||||||| || ||||| || | ||| |||||||| |||||||||||||||||||||||

Sbjct 13 TATTTTTGGRGTTTGATCTGGAATAGTAGGAACTTCTTTAAGAGTTTTAATTCGAACTGA 72

Query 65 ACTTAGTCACCCTGGAATATTTATCGGAAATGATCAAATTTATAATGTAATTCGTTACTA 124

||||||||||||||| |||||||| |||||||| |||||||||||||||||| ||||||

Sbjct 73 ACTTAGTCACCCTGGTATATTTATTGGAAATGACCAAATTTATAATGTAATT-GTTACT- 130

Query 125 GCTCATGCATTTATTATAAttttttttATAGTAATACCTATTATAATTGGAGGATTTGGA 184

||||||||||||||||||||||||||||||||||||||||||||||||||||||||||||

Sbjct 131 GCTCATGCATTTATTATAATTTTTTTTATAGTAATACCTATTATAATTGGAGGATTTGGA 190

Query 185 AATTGGTTAGTCCCATTAATATTAGGTGCTCCTGATATAGCTTTCCCTCGAATAAATAAT 244

||||| ||||| || ||||||||||| || |||||||||||||| |||||||||||||||

Sbjct 191 AATTGATTAGTTCCTTTAATATTAGGAGCCCCTGATATAGCTTTTCCTCGAATAAATAAT 250

Query 245 ATAAGTTTTTGAATACTTCCTCCCTCTTTAACTCTTTTAATTTCTAGATCTATAGTAGAA 304

||||||||||||||||||||||| ||||||||||||||| ||||||||||||||||||||

Sbjct 251 ATAAGTTTTTGAATACTTCCTCCTTCTTTAACTCTTTTACTTTCTAGATCTATAGTAGAA 310

Query 305 AATGGAGCAGGAACAGGTTGAACTGTATACCCTCCTCTTTCTTCTGGAACTGCTCATGCA 364

|||||||||||||| ||||||||||| || |||||||||||||||||||| ||||||||

Sbjct 311 AATGGAGCAGGAACTGGTTGAACTGTTTATCCTCCTCTTTCTTCTGGAACCGCTCATGCT 370

Query 365 GGAGCTTCTGTAGATTTAGCTATTTTTTCTCTTCATTTAGCAGGAATTTCTTCTATTTTA 424

|||||||| || |||||||||||||||||| | ||||||||||| ||||| |||||||||

Sbjct 371 GGAGCTTCAGTTGATTTAGCTATTTTTTCTTTACATTTAGCAGGTATTTCATCTATTTTA 430

Query 425 GGAGCAGTAAATTTTATTACAACTGTAATTAATATACGATCAACTGGAATTACACTTGAT 484

||||||||||||||||||||||||||||||||||| |||||| |||||||||||||||||

Sbjct 431 GGAGCAGTAAATTTTATTACAACTGTAATTAATATGCGATCAGCTGGAATTACACTTGAT 490

Query 485 CGTTTACCTTTATTTGTCTGATCTGTAATTATTACAGCTATTTTATTACTTCTTTCATTA 544

|| |||||||||||||| ||||| ||| | |||||||| |||||||||||||||||||||

Sbjct 491 CGATTACCTTTATTTGTTTGATCAGTATTAATTACAGCAATTTTATTACTTCTTTCATTA 550

Query 545 CCAGTTTTAGCAGGAGCTATTACTATATTATTAACTGATCGAAATTTTAATACATCATTT 604

|| |||||||||||||| || ||||| |||||||||||||||||||||||||||||||||

Sbjct 551 CCTGTTTTAGCAGGAGCAATCACTATGTTATTAACTGATCGAAATTTTAATACATCATTT 610

Query 605 TTTGACCCAATTGGAGGAGGTGACCCTATTCTTTA 639

|||||||||||||||||||||||||||||||||||

Sbjct 611 TTTGACCCAATTGGAGGAGGTGACCCTATTCTTTA 645

>Aedes polynesiensis voucher WA068 cytochrome c oxidase subunit I (COX1) gene, partial cds; mitochondrial

Sequence ID: MW664787.1 Length: 618

Range 1: 1 to 618

Score:920 bits(498), Expect:0.0,

Identities:580/620(94%), Gaps:3/620(0%), Strand: Plus/Plus

Query 19 TGATCGGGGA-AGTCGGAACTTCATTAAGAGTTTTAATTCGAACTGAACTTAGTCACCCT 77

||||| || | ||| |||||||| ||||||||||||||||||||||||||||||||||||

Sbjct 1 TGATCTGGAATAGTAGGAACTTCTTTAAGAGTTTTAATTCGAACTGAACTTAGTCACCCT 60

Query 78 GGAATATTTATCGGAAATGATCAAATTTATAATGTAATTCGTTACTAGCTCATGCATTTA 137

|| |||||||| |||||||| |||||||||||||||||| |||||| |||||||||||||

Sbjct 61 GGTATATTTATTGGAAATGACCAAATTTATAATGTAATT-GTTACT-GCTCATGCATTTA 118

Query 138 TTATAAttttttttATAGTAATACCTATTATAATTGGAGGATTTGGAAATTGGTTAGTCC 197

|||||||||||||||||||||||||||||||||||||||||||||||||||| ||||| |

Sbjct 119 TTATAATTTTTTTTATAGTAATACCTATTATAATTGGAGGATTTGGAAATTGATTAGTTC 178

Query 198 CATTAATATTAGGTGCTCCTGATATAGCTTTCCCTCGAATAAATAATATAAGTTTTTGAA 257

| ||||||||||| || |||||||||||||| ||||||||||||||||||||||||||||

Sbjct 179 CTTTAATATTAGGAGCCCCTGATATAGCTTTTCCTCGAATAAATAATATAAGTTTTTGAA 238

Query 258 TACTTCCTCCCTCTTTAACTCTTTTAATTTCTAGATCTATAGTAGAAAATGGAGCAGGAA 317

|||||||||| ||||||||||||||| |||||||||||||||||||||||||||||||||

Sbjct 239 TACTTCCTCCTTCTTTAACTCTTTTACTTTCTAGATCTATAGTAGAAAATGGAGCAGGAA 298

Query 318 CAGGTTGAACTGTATACCCTCCTCTTTCTTCTGGAACTGCTCATGCAGGAGCTTCTGTAG 377

| ||||||||||| || |||||||||||||||||||| |||||||| |||||||| || |

Sbjct 299 CTGGTTGAACTGTTTATCCTCCTCTTTCTTCTGGAACCGCTCATGCTGGAGCTTCAGTTG 358

Query 378 ATTTAGCTATTTTTTCTCTTCATTTAGCAGGAATTTCTTCTATTTTAGGAGCAGTAAATT 437

||||||||||||||||| | ||||||||||| ||||| ||||||||||||||||||||||

Sbjct 359 ATTTAGCTATTTTTTCTTTACATTTAGCAGGTATTTCATCTATTTTAGGAGCAGTAAATT 418

Query 438 TTATTACAACTGTAATTAATATACGATCAACTGGAATTACACTTGATCGTTTACCTTTAT 497

||||||||||||||||||||||||||||| ||||||||||||||||||| ||||||||||

Sbjct 419 TTATTACAACTGTAATTAATATACGATCAGCTGGAATTACACTTGATCGATTACCTTTAT 478

Query 498 TTGTCTGATCTGTAATTATTACAGCTATTTTATTACTTCTTTCATTACCAGTTTTAGCAG 557

|||| ||||| ||| | |||||||| ||||||||||||||||||||||| ||||||||||

Sbjct 479 TTGTTTGATCAGTAGTAATTACAGCAATTTTATTACTTCTTTCATTACCTGTTTTAGCAG 538

Query 558 GAGCTATTACTATATTATTAACTGATCGAAATTTTAATACATCATTTTTTGACCCAATTG 617

|||| || ||||||||||||||||||||||||||||||||||||||||||||||||||||

Sbjct 539 GAGCAATCACTATATTATTAACTGATCGAAATTTTAATACATCATTTTTTGACCCAATTG 598

Query 618 GAGGAGGTGACCCTATTCTT 637

|||||||||| |||||||||

Sbjct 599 GAGGAGGTGATCCTATTCTT 618

>Aedes polynesiensis voucher WA063 cytochrome c oxidase subunit I (COX1) gene, partial cds; mitochondrial

Sequence ID: MW664783.1 Length: 618

Range 1: 1 to 618

Score:920 bits(498), Expect:0.0,

Identities:580/620(94%), Gaps:3/620(0%), Strand: Plus/Plus

Query 19 TGATCGGGGA-AGTCGGAACTTCATTAAGAGTTTTAATTCGAACTGAACTTAGTCACCCT 77

||||| || | ||| |||||||| ||||||||||||||||||||||||||||||||||||

Sbjct 1 TGATCTGGAATAGTAGGAACTTCTTTAAGAGTTTTAATTCGAACTGAACTTAGTCACCCT 60

Query 78 GGAATATTTATCGGAAATGATCAAATTTATAATGTAATTCGTTACTAGCTCATGCATTTA 137

|| |||||||| |||||||| |||||||||||||||||| |||||| |||||||||||||

Sbjct 61 GGTATATTTATTGGAAATGACCAAATTTATAATGTAATT-GTTACT-GCTCATGCATTTA 118

Query 138 TTATAAttttttttATAGTAATACCTATTATAATTGGAGGATTTGGAAATTGGTTAGTCC 197

|||||||||||||||||||||||||||||||||||||||||||||||||||| ||||| |

Sbjct 119 TTATAATTTTTTTTATAGTAATACCTATTATAATTGGAGGATTTGGAAATTGATTAGTTC 178

Query 198 CATTAATATTAGGTGCTCCTGATATAGCTTTCCCTCGAATAAATAATATAAGTTTTTGAA 257

| ||||||||||| || |||||||||||||| ||||||||||||||||||||||||||||

Sbjct 179 CTTTAATATTAGGAGCCCCTGATATAGCTTTTCCTCGAATAAATAATATAAGTTTTTGAA 238

Query 258 TACTTCCTCCCTCTTTAACTCTTTTAATTTCTAGATCTATAGTAGAAAATGGAGCAGGAA 317

|||||||||| ||||||||||||||| |||||||||||||||||||||||||||||||||

Sbjct 239 TACTTCCTCCTTCTTTAACTCTTTTACTTTCTAGATCTATAGTAGAAAATGGAGCAGGAA 298

Query 318 CAGGTTGAACTGTATACCCTCCTCTTTCTTCTGGAACTGCTCATGCAGGAGCTTCTGTAG 377

| ||||||||||| || |||||||||||||||||||| |||||||| |||||||| || |

Sbjct 299 CTGGTTGAACTGTTTATCCTCCTCTTTCTTCTGGAACCGCTCATGCTGGAGCTTCAGTTG 358

Query 378 ATTTAGCTATTTTTTCTCTTCATTTAGCAGGAATTTCTTCTATTTTAGGAGCAGTAAATT 437

||||||||||||||||| | ||||||||||| ||||| ||||||||||||||||||||||

Sbjct 359 ATTTAGCTATTTTTTCTTTACATTTAGCAGGTATTTCATCTATTTTAGGAGCAGTAAATT 418

Query 438 TTATTACAACTGTAATTAATATACGATCAACTGGAATTACACTTGATCGTTTACCTTTAT 497

||||||||||||||||||||||||||||| ||||||||||||||||||| ||||||||||

Sbjct 419 TTATTACAACTGTAATTAATATACGATCAGCTGGAATTACACTTGATCGATTACCTTTAT 478

Query 498 TTGTCTGATCTGTAATTATTACAGCTATTTTATTACTTCTTTCATTACCAGTTTTAGCAG 557

|||| ||||| ||| | |||||||| ||||||||||||||||||||||| ||||||||||

Sbjct 479 TTGTTTGATCAGTAGTAATTACAGCAATTTTATTACTTCTTTCATTACCTGTTTTAGCAG 538

Query 558 GAGCTATTACTATATTATTAACTGATCGAAATTTTAATACATCATTTTTTGACCCAATTG 617

|||| || ||||||||||||||||||||||||||||||||||||||||||||||||||||

Sbjct 539 GAGCAATCACTATATTATTAACTGATCGAAATTTTAATACATCATTTTTTGACCCAATTG 598

Query 618 GAGGAGGTGACCCTATTCTT 637

|||||||||| |||||||||

Sbjct 599 GAGGAGGTGATCCTATTCTT 618

>Aedes polynesiensis voucher WA062 cytochrome c oxidase subunit I (COX1) gene, partial cds; mitochondrial

Sequence ID: MW664782.1 Length: 618

Range 1: 1 to 618

Score:920 bits(498), Expect:0.0,

Identities:580/620(94%), Gaps:3/620(0%), Strand: Plus/Plus

Query 19 TGATCGGGGA-AGTCGGAACTTCATTAAGAGTTTTAATTCGAACTGAACTTAGTCACCCT 77

||||| || | ||| |||||||| ||||||||||||||||||||||||||||||||||||

Sbjct 1 TGATCTGGAATAGTAGGAACTTCTTTAAGAGTTTTAATTCGAACTGAACTTAGTCACCCT 60

Query 78 GGAATATTTATCGGAAATGATCAAATTTATAATGTAATTCGTTACTAGCTCATGCATTTA 137

|| |||||||| |||||||| |||||||||||||||||| |||||| |||||||||||||

Sbjct 61 GGTATATTTATTGGAAATGACCAAATTTATAATGTAATT-GTTACT-GCTCATGCATTTA 118

Query 138 TTATAAttttttttATAGTAATACCTATTATAATTGGAGGATTTGGAAATTGGTTAGTCC 197

|||||||||||||||||||||||||||||||||||||||||||||||||||| ||||| |

Sbjct 119 TTATAATTTTTTTTATAGTAATACCTATTATAATTGGAGGATTTGGAAATTGATTAGTTC 178

Query 198 CATTAATATTAGGTGCTCCTGATATAGCTTTCCCTCGAATAAATAATATAAGTTTTTGAA 257

| ||||||||||| || |||||||||||||| ||||||||||||||||||||||||||||

Sbjct 179 CTTTAATATTAGGAGCCCCTGATATAGCTTTTCCTCGAATAAATAATATAAGTTTTTGAA 238

Query 258 TACTTCCTCCCTCTTTAACTCTTTTAATTTCTAGATCTATAGTAGAAAATGGAGCAGGAA 317

|||||||||| ||||||||||||||| |||||||||||||||||||||||||||||||||

Sbjct 239 TACTTCCTCCTTCTTTAACTCTTTTACTTTCTAGATCTATAGTAGAAAATGGAGCAGGAA 298

Query 318 CAGGTTGAACTGTATACCCTCCTCTTTCTTCTGGAACTGCTCATGCAGGAGCTTCTGTAG 377

| ||||||||||| || |||||||||||||||||||| |||||||| |||||||| || |

Sbjct 299 CTGGTTGAACTGTTTATCCTCCTCTTTCTTCTGGAACCGCTCATGCTGGAGCTTCAGTTG 358

Query 378 ATTTAGCTATTTTTTCTCTTCATTTAGCAGGAATTTCTTCTATTTTAGGAGCAGTAAATT 437

||||||||||||||||| | ||||||||||| ||||| ||||||||||||||||||||||

Sbjct 359 ATTTAGCTATTTTTTCTTTACATTTAGCAGGTATTTCATCTATTTTAGGAGCAGTAAATT 418

Query 438 TTATTACAACTGTAATTAATATACGATCAACTGGAATTACACTTGATCGTTTACCTTTAT 497

||||||||||||||||||||||||||||| ||||||||||||||||||| ||||||||||

Sbjct 419 TTATTACAACTGTAATTAATATACGATCAGCTGGAATTACACTTGATCGATTACCTTTAT 478

Query 498 TTGTCTGATCTGTAATTATTACAGCTATTTTATTACTTCTTTCATTACCAGTTTTAGCAG 557

|||| ||||| ||| | |||||||| ||||||||||||||||||||||| ||||||||||

Sbjct 479 TTGTTTGATCAGTAGTAATTACAGCAATTTTATTACTTCTTTCATTACCTGTTTTAGCAG 538

Query 558 GAGCTATTACTATATTATTAACTGATCGAAATTTTAATACATCATTTTTTGACCCAATTG 617

|||| || ||||||||||||||||||||||||||||||||||||||||||||||||||||

Sbjct 539 GAGCAATCACTATATTATTAACTGATCGAAATTTTAATACATCATTTTTTGACCCAATTG 598

Query 618 GAGGAGGTGACCCTATTCTT 637

|||||||||| |||||||||

Sbjct 599 GAGGAGGTGATCCTATTCTT 618

>Aedes polynesiensis voucher WA042 cytochrome c oxidase subunit I (COX1) gene, partial cds; mitochondrial

Sequence ID: MW664775.1 Length: 618

Range 1: 1 to 618

Score:920 bits(498), Expect:0.0,

Identities:580/620(94%), Gaps:3/620(0%), Strand: Plus/Plus

Query 19 TGATCGGGGA-AGTCGGAACTTCATTAAGAGTTTTAATTCGAACTGAACTTAGTCACCCT 77

||||| || | ||| |||||||| ||||||||||||||||||||||||||||||||||||

Sbjct 1 TGATCTGGAATAGTAGGAACTTCTTTAAGAGTTTTAATTCGAACTGAACTTAGTCACCCT 60

Query 78 GGAATATTTATCGGAAATGATCAAATTTATAATGTAATTCGTTACTAGCTCATGCATTTA 137

|| |||||||| |||||||| |||||||||||||||||| |||||| |||||||||||||

Sbjct 61 GGTATATTTATTGGAAATGACCAAATTTATAATGTAATT-GTTACT-GCTCATGCATTTA 118

Query 138 TTATAAttttttttATAGTAATACCTATTATAATTGGAGGATTTGGAAATTGGTTAGTCC 197

|||||||||||||||||||||||||||||||||||||||||||||||||||| ||||| |

Sbjct 119 TTATAATTTTTTTTATAGTAATACCTATTATAATTGGAGGATTTGGAAATTGATTAGTTC 178

Query 198 CATTAATATTAGGTGCTCCTGATATAGCTTTCCCTCGAATAAATAATATAAGTTTTTGAA 257

| ||||||||||| || |||||||||||||| ||||||||||||||||||||||||||||

Sbjct 179 CTTTAATATTAGGAGCCCCTGATATAGCTTTTCCTCGAATAAATAATATAAGTTTTTGAA 238

Query 258 TACTTCCTCCCTCTTTAACTCTTTTAATTTCTAGATCTATAGTAGAAAATGGAGCAGGAA 317

|||||||||| ||||||||||||||| |||||||||||||||||||||||||||||||||

Sbjct 239 TACTTCCTCCTTCTTTAACTCTTTTACTTTCTAGATCTATAGTAGAAAATGGAGCAGGAA 298

Query 318 CAGGTTGAACTGTATACCCTCCTCTTTCTTCTGGAACTGCTCATGCAGGAGCTTCTGTAG 377

| ||||||||||| || |||||||||||||||||||| |||||||| |||||||| || |

Sbjct 299 CTGGTTGAACTGTTTATCCTCCTCTTTCTTCTGGAACCGCTCATGCTGGAGCTTCAGTTG 358

Query 378 ATTTAGCTATTTTTTCTCTTCATTTAGCAGGAATTTCTTCTATTTTAGGAGCAGTAAATT 437

||||||||||||||||| | ||||||||||| ||||| ||||||||||||||||||||||

Sbjct 359 ATTTAGCTATTTTTTCTTTACATTTAGCAGGTATTTCATCTATTTTAGGAGCAGTAAATT 418

Query 438 TTATTACAACTGTAATTAATATACGATCAACTGGAATTACACTTGATCGTTTACCTTTAT 497

||||||||||||||||||||||||||||||||||||||||||||||||| ||||||||||

Sbjct 419 TTATTACAACTGTAATTAATATACGATCAACTGGAATTACACTTGATCGATTACCTTTAT 478

Query 498 TTGTCTGATCTGTAATTATTACAGCTATTTTATTACTTCTTTCATTACCAGTTTTAGCAG 557

|||| ||||| ||| | |||||||| ||||||||||||||||||||||| ||||||||||

Sbjct 479 TTGTTTGATCAGTAGTAATTACAGCAATTTTATTACTTCTTTCATTACCTGTTTTAGCAG 538

Query 558 GAGCTATTACTATATTATTAACTGATCGAAATTTTAATACATCATTTTTTGACCCAATTG 617

|||| || ||||| ||||||||||||||||||||||||||||||||||||||||||||||

Sbjct 539 GAGCAATCACTATGTTATTAACTGATCGAAATTTTAATACATCATTTTTTGACCCAATTG 598

Query 618 GAGGAGGTGACCCTATTCTT 637

|||||||||| |||||||||

Sbjct 599 GAGGAGGTGATCCTATTCTT 618

>Aedes polynesiensis voucher WA067 cytochrome c oxidase subunit I (COX1) gene, partial cds; mitochondrial

Sequence ID: MW664786.1 Length: 618

Range 1: 1 to 618

Score:915 bits(495), Expect:0.0,

Identities:579/620(93%), Gaps:3/620(0%), Strand: Plus/Plus

Query 19 TGATCGGGGA-AGTCGGAACTTCATTAAGAGTTTTAATTCGAACTGAACTTAGTCACCCT 77

||||| || | ||| |||||||| ||||||||||||||||||||||||||||||||||||

Sbjct 1 TGATCTGGAATAGTAGGAACTTCTTTAAGAGTTTTAATTCGAACTGAACTTAGTCACCCT 60

Query 78 GGAATATTTATCGGAAATGATCAAATTTATAATGTAATTCGTTACTAGCTCATGCATTTA 137

|| |||||||| |||||||| |||||||||||||||||| |||||| |||||||||||||

Sbjct 61 GGTATATTTATTGGAAATGACCAAATTTATAATGTAATT-GTTACT-GCTCATGCATTTA 118

Query 138 TTATAAttttttttATAGTAATACCTATTATAATTGGAGGATTTGGAAATTGGTTAGTCC 197

|||||||||||||||||||||||||||||||||||||||||||||||||||| ||||| |

Sbjct 119 TTATAATTTTTTTTATAGTAATACCTATTATAATTGGAGGATTTGGAAATTGATTAGTTC 178

Query 198 CATTAATATTAGGTGCTCCTGATATAGCTTTCCCTCGAATAAATAATATAAGTTTTTGAA 257

| ||||||||||| || |||||||||||||| ||||||||||||||||||||||||||||

Sbjct 179 CTTTAATATTAGGAGCCCCTGATATAGCTTTTCCTCGAATAAATAATATAAGTTTTTGAA 238

Query 258 TACTTCCTCCCTCTTTAACTCTTTTAATTTCTAGATCTATAGTAGAAAATGGAGCAGGAA 317

|||||||||| ||||||||||||||| |||||||||||||||||||||||||||||||||

Sbjct 239 TACTTCCTCCTTCTTTAACTCTTTTACTTTCTAGATCTATAGTAGAAAATGGAGCAGGAA 298

Query 318 CAGGTTGAACTGTATACCCTCCTCTTTCTTCTGGAACTGCTCATGCAGGAGCTTCTGTAG 377

| ||||||||||| || |||||||||||||||||||| |||||||| |||||||| || |

Sbjct 299 CTGGTTGAACTGTTTATCCTCCTCTTTCTTCTGGAACCGCTCATGCTGGAGCTTCAGTTG 358

Query 378 ATTTAGCTATTTTTTCTCTTCATTTAGCAGGAATTTCTTCTATTTTAGGAGCAGTAAATT 437

||||||||||||||||| | ||||||||||| ||||| ||||||||||||||||||||||

Sbjct 359 ATTTAGCTATTTTTTCTTTACATTTAGCAGGTATTTCATCTATTTTAGGAGCAGTAAATT 418

Query 438 TTATTACAACTGTAATTAATATACGATCAACTGGAATTACACTTGATCGTTTACCTTTAT 497

||||||||||||||||||||||||||||| ||||||||||||||||||| ||||||||||

Sbjct 419 TTATTACAACTGTAATTAATATACGATCAGCTGGAATTACACTTGATCGATTACCTTTAT 478

Query 498 TTGTCTGATCTGTAATTATTACAGCTATTTTATTACTTCTTTCATTACCAGTTTTAGCAG 557

|||| ||||| ||| | |||||||| ||||||||||||||||||||||| ||||||||||

Sbjct 479 TTGTTTGATCAGTAGTAATTACAGCAATTTTATTACTTCTTTCATTACCTGTTTTAGCAG 538

Query 558 GAGCTATTACTATATTATTAACTGATCGAAATTTTAATACATCATTTTTTGACCCAATTG 617

|||| || ||||| ||||||||||||||||||||||||||||||||||||||||||||||

Sbjct 539 GAGCAATCACTATGTTATTAACTGATCGAAATTTTAATACATCATTTTTTGACCCAATTG 598

Query 618 GAGGAGGTGACCCTATTCTT 637

|||||||||| |||||||||

Sbjct 599 GAGGAGGTGATCCTATTCTT 618

>Aedes polynesiensis voucher WA065 cytochrome c oxidase subunit I (COX1) gene, partial cds; mitochondrial

Sequence ID: MW664785.1 Length: 618

Range 1: 1 to 618

Score:915 bits(495), Expect:0.0,

Identities:579/620(93%), Gaps:3/620(0%), Strand: Plus/Plus

Query 19 TGATCGGGGA-AGTCGGAACTTCATTAAGAGTTTTAATTCGAACTGAACTTAGTCACCCT 77

||||| || | ||| |||||||| ||||||||||||||||||||||||||||||||||||

Sbjct 1 TGATCTGGAATAGTAGGAACTTCTTTAAGAGTTTTAATTCGAACTGAACTTAGTCACCCT 60

Query 78 GGAATATTTATCGGAAATGATCAAATTTATAATGTAATTCGTTACTAGCTCATGCATTTA 137

|| |||||||| |||||||| |||||||||||||||||| |||||| |||||||||||||

Sbjct 61 GGTATATTTATTGGAAATGACCAAATTTATAATGTAATT-GTTACT-GCTCATGCATTTA 118

Query 138 TTATAAttttttttATAGTAATACCTATTATAATTGGAGGATTTGGAAATTGGTTAGTCC 197

|||||||||||||||||||||||||||||||||||||||||||||||||||| ||||| |

Sbjct 119 TTATAATTTTTTTTATAGTAATACCTATTATAATTGGAGGATTTGGAAATTGATTAGTTC 178

Query 198 CATTAATATTAGGTGCTCCTGATATAGCTTTCCCTCGAATAAATAATATAAGTTTTTGAA 257

| ||||||||||| || |||||||||||||| ||||||||||||||||||||||||||||

Sbjct 179 CTTTAATATTAGGAGCCCCTGATATAGCTTTTCCTCGAATAAATAATATAAGTTTTTGAA 238

Query 258 TACTTCCTCCCTCTTTAACTCTTTTAATTTCTAGATCTATAGTAGAAAATGGAGCAGGAA 317

|||||||||| ||||||||||||||| |||||||||||||||||||||||||||||||||

Sbjct 239 TACTTCCTCCTTCTTTAACTCTTTTACTTTCTAGATCTATAGTAGAAAATGGAGCAGGAA 298

Query 318 CAGGTTGAACTGTATACCCTCCTCTTTCTTCTGGAACTGCTCATGCAGGAGCTTCTGTAG 377

| ||||||||||| || |||||||||||||||||||| |||||||| |||||||| || |

Sbjct 299 CTGGTTGAACTGTTTATCCTCCTCTTTCTTCTGGAACCGCTCATGCTGGAGCTTCAGTTG 358

Query 378 ATTTAGCTATTTTTTCTCTTCATTTAGCAGGAATTTCTTCTATTTTAGGAGCAGTAAATT 437

||||||||||||||||| | ||||||||||| ||||| ||||||||||||||||||||||

Sbjct 359 ATTTAGCTATTTTTTCTTTACATTTAGCAGGTATTTCATCTATTTTAGGAGCAGTAAATT 418

Query 438 TTATTACAACTGTAATTAATATACGATCAACTGGAATTACACTTGATCGTTTACCTTTAT 497

||||||||||||||||||||||||||||| ||||||||||||||||||| ||||||||||

Sbjct 419 TTATTACAACTGTAATTAATATACGATCAGCTGGAATTACACTTGATCGATTACCTTTAT 478

Query 498 TTGTCTGATCTGTAATTATTACAGCTATTTTATTACTTCTTTCATTACCAGTTTTAGCAG 557

|||| ||||| ||| | |||||||| ||||||||||||||||||||||| ||||||||||

Sbjct 479 TTGTTTGATCAGTAGTAATTACAGCAATTTTATTACTTCTTTCATTACCTGTTTTAGCAG 538

Query 558 GAGCTATTACTATATTATTAACTGATCGAAATTTTAATACATCATTTTTTGACCCAATTG 617

|||| || ||||| ||||||||||||||||||||||||||||||||||||||||||||||

Sbjct 539 GAGCAATCACTATGTTATTAACTGATCGAAATTTTAATACATCATTTTTTGACCCAATTG 598

Query 618 GAGGAGGTGACCCTATTCTT 637

|||||||||| |||||||||

Sbjct 599 GAGGAGGTGATCCTATTCTT 618

>Aedes polynesiensis voucher WA055 cytochrome c oxidase subunit I (COX1) gene, partial cds; mitochondrial

Sequence ID: MW664780.1 Length: 618

Range 1: 1 to 618

Score:915 bits(495), Expect:0.0,

Identities:579/620(93%), Gaps:3/620(0%), Strand: Plus/Plus

Query 19 TGATCGGGGA-AGTCGGAACTTCATTAAGAGTTTTAATTCGAACTGAACTTAGTCACCCT 77

||||| || | ||| |||||||| ||||||||||||||||||||||||||||||||||||

Sbjct 1 TGATCTGGAATAGTAGGAACTTCTTTAAGAGTTTTAATTCGAACTGAACTTAGTCACCCT 60

Query 78 GGAATATTTATCGGAAATGATCAAATTTATAATGTAATTCGTTACTAGCTCATGCATTTA 137

|| |||||||| |||||||| |||||||||||||||||| |||||| |||||||||||||

Sbjct 61 GGTATATTTATTGGAAATGACCAAATTTATAATGTAATT-GTTACT-GCTCATGCATTTA 118

Query 138 TTATAAttttttttATAGTAATACCTATTATAATTGGAGGATTTGGAAATTGGTTAGTCC 197

|||||||||||||||||||||||||||||||||||||||||||||||||||| ||||| |

Sbjct 119 TTATAATTTTTTTTATAGTAATACCTATTATAATTGGAGGATTTGGAAATTGATTAGTTC 178

Query 198 CATTAATATTAGGTGCTCCTGATATAGCTTTCCCTCGAATAAATAATATAAGTTTTTGAA 257

| ||||||||||| || |||||||||||||| ||||||||||||||||||||||||||||

Sbjct 179 CTTTAATATTAGGAGCCCCTGATATAGCTTTTCCTCGAATAAATAATATAAGTTTTTGAA 238

Query 258 TACTTCCTCCCTCTTTAACTCTTTTAATTTCTAGATCTATAGTAGAAAATGGAGCAGGAA 317

|||||||||| ||||||||||||||| |||||||||||||||||||||||||||||||||

Sbjct 239 TACTTCCTCCTTCTTTAACTCTTTTACTTTCTAGATCTATAGTAGAAAATGGAGCAGGAA 298

Query 318 CAGGTTGAACTGTATACCCTCCTCTTTCTTCTGGAACTGCTCATGCAGGAGCTTCTGTAG 377

| ||||||||||| || |||||||||||||||||||| |||||||| |||||||| || |

Sbjct 299 CTGGTTGAACTGTTTATCCTCCTCTTTCTTCTGGAACCGCTCATGCTGGAGCTTCAGTTG 358

Query 378 ATTTAGCTATTTTTTCTCTTCATTTAGCAGGAATTTCTTCTATTTTAGGAGCAGTAAATT 437

||||||||||||||||| | ||||||||||| ||||| ||||||||||||||||||||||

Sbjct 359 ATTTAGCTATTTTTTCTTTACATTTAGCAGGTATTTCATCTATTTTAGGAGCAGTAAATT 418

Query 438 TTATTACAACTGTAATTAATATACGATCAACTGGAATTACACTTGATCGTTTACCTTTAT 497

||||||||||||||||||||||||||||| ||||||||||||||||||| ||||||||||

Sbjct 419 TTATTACAACTGTAATTAATATACGATCAGCTGGAATTACACTTGATCGATTACCTTTAT 478

Query 498 TTGTCTGATCTGTAATTATTACAGCTATTTTATTACTTCTTTCATTACCAGTTTTAGCAG 557

|||| ||||| ||| | |||||||| ||||||||||||||||||||||| ||||||||||

Sbjct 479 TTGTTTGATCAGTAGTAATTACAGCAATTTTATTACTTCTTTCATTACCTGTTTTAGCAG 538

Query 558 GAGCTATTACTATATTATTAACTGATCGAAATTTTAATACATCATTTTTTGACCCAATTG 617

|||| || ||||| ||||||||||||||||||||||||||||||||||||||||||||||

Sbjct 539 GAGCAATCACTATGTTATTAACTGATCGAAATTTTAATACATCATTTTTTGACCCAATTG 598

Query 618 GAGGAGGTGACCCTATTCTT 637

|||||||||| |||||||||

Sbjct 599 GAGGAGGTGATCCTATTCTT 618

>Aedes polynesiensis voucher WA041 cytochrome c oxidase subunit I (COX1) gene, partial cds; mitochondrial

Sequence ID: MW664774.1 Length: 618

Range 1: 1 to 618

Score:915 bits(495), Expect:0.0,

Identities:579/620(93%), Gaps:3/620(0%), Strand: Plus/Plus

Query 19 TGATCGGGGA-AGTCGGAACTTCATTAAGAGTTTTAATTCGAACTGAACTTAGTCACCCT 77

||||| || | ||| |||||||| ||||||||||||||||||||||||||||||||||||

Sbjct 1 TGATCTGGAATAGTAGGAACTTCTTTAAGAGTTTTAATTCGAACTGAACTTAGTCACCCT 60

Query 78 GGAATATTTATCGGAAATGATCAAATTTATAATGTAATTCGTTACTAGCTCATGCATTTA 137

|| |||||||| |||||||| |||||||||||||||||| |||||| |||||||||||||

Sbjct 61 GGTATATTTATTGGAAATGACCAAATTTATAATGTAATT-GTTACT-GCTCATGCATTTA 118

Query 138 TTATAAttttttttATAGTAATACCTATTATAATTGGAGGATTTGGAAATTGGTTAGTCC 197

|||||||||||||||||||||||||||||||||||||||||||||||||||| ||||| |

Sbjct 119 TTATAATTTTTTTTATAGTAATACCTATTATAATTGGAGGATTTGGAAATTGATTAGTTC 178

Query 198 CATTAATATTAGGTGCTCCTGATATAGCTTTCCCTCGAATAAATAATATAAGTTTTTGAA 257

| ||||||||||| || |||||||||||||| ||||||||||||||||||||||||||||

Sbjct 179 CTTTAATATTAGGAGCCCCTGATATAGCTTTTCCTCGAATAAATAATATAAGTTTTTGAA 238

Query 258 TACTTCCTCCCTCTTTAACTCTTTTAATTTCTAGATCTATAGTAGAAAATGGAGCAGGAA 317

|||||||||| ||||||||||||||| |||||||||||||||||||||||||||||||||

Sbjct 239 TACTTCCTCCTTCTTTAACTCTTTTACTTTCTAGATCTATAGTAGAAAATGGAGCAGGAA 298

Query 318 CAGGTTGAACTGTATACCCTCCTCTTTCTTCTGGAACTGCTCATGCAGGAGCTTCTGTAG 377

| ||||||||||| || |||||||||||||||||||| |||||||| |||||||| || |

Sbjct 299 CTGGTTGAACTGTTTATCCTCCTCTTTCTTCTGGAACCGCTCATGCTGGAGCTTCAGTTG 358

Query 378 ATTTAGCTATTTTTTCTCTTCATTTAGCAGGAATTTCTTCTATTTTAGGAGCAGTAAATT 437

||||||||||||||||| | ||||||||||| ||||| ||||||||||||||||||||||

Sbjct 359 ATTTAGCTATTTTTTCTTTACATTTAGCAGGTATTTCATCTATTTTAGGAGCAGTAAATT 418

Query 438 TTATTACAACTGTAATTAATATACGATCAACTGGAATTACACTTGATCGTTTACCTTTAT 497

||||||||||||||||||||||||||||| ||||||||||||||||||| ||||||||||

Sbjct 419 TTATTACAACTGTAATTAATATACGATCAGCTGGAATTACACTTGATCGATTACCTTTAT 478

Query 498 TTGTCTGATCTGTAATTATTACAGCTATTTTATTACTTCTTTCATTACCAGTTTTAGCAG 557

|||| ||||| ||| | |||||||| ||||||||||||||||||||||| ||||||||||

Sbjct 479 TTGTTTGATCAGTAGTAATTACAGCAATTTTATTACTTCTTTCATTACCTGTTTTAGCAG 538

Query 558 GAGCTATTACTATATTATTAACTGATCGAAATTTTAATACATCATTTTTTGACCCAATTG 617

|||| || ||||| ||||||||||||||||||||||||||||||||||||||||||||||

Sbjct 539 GAGCAATCACTATGTTATTAACTGATCGAAATTTTAATACATCATTTTTTGACCCAATTG 598

Query 618 GAGGAGGTGACCCTATTCTT 637

|||||||||| |||||||||

Sbjct 599 GAGGAGGTGATCCTATTCTT 618

>Aedes polynesiensis voucher WA038 cytochrome c oxidase subunit I (COX1) gene, partial cds; mitochondrial

Sequence ID: MW664773.1 Length: 618

Range 1: 1 to 618

Score:915 bits(495), Expect:0.0,

Identities:579/620(93%), Gaps:3/620(0%), Strand: Plus/Plus

Query 19 TGATCGGGGA-AGTCGGAACTTCATTAAGAGTTTTAATTCGAACTGAACTTAGTCACCCT 77

||||| || | ||| |||||||| ||||||||||||||||||||||||||||||||||||

Sbjct 1 TGATCTGGAATAGTAGGAACTTCTTTAAGAGTTTTAATTCGAACTGAACTTAGTCACCCT 60

Query 78 GGAATATTTATCGGAAATGATCAAATTTATAATGTAATTCGTTACTAGCTCATGCATTTA 137

|| |||||||| |||||||| |||||||||||||||||| |||||| |||||||||||||

Sbjct 61 GGTATATTTATTGGAAATGACCAAATTTATAATGTAATT-GTTACT-GCTCATGCATTTA 118

Query 138 TTATAAttttttttATAGTAATACCTATTATAATTGGAGGATTTGGAAATTGGTTAGTCC 197

|||||||||||||||||||||||||||||||||||||||||||||||||||| ||||| |

Sbjct 119 TTATAATTTTTTTTATAGTAATACCTATTATAATTGGAGGATTTGGAAATTGATTAGTTC 178

Query 198 CATTAATATTAGGTGCTCCTGATATAGCTTTCCCTCGAATAAATAATATAAGTTTTTGAA 257

| ||||||||||| || |||||||||||||| ||||||||||||||||||||||||||||

Sbjct 179 CTTTAATATTAGGAGCCCCTGATATAGCTTTTCCTCGAATAAATAATATAAGTTTTTGAA 238

Query 258 TACTTCCTCCCTCTTTAACTCTTTTAATTTCTAGATCTATAGTAGAAAATGGAGCAGGAA 317

|||||||||| ||||||||||||||| |||||||||||||||||||||||||||||||||

Sbjct 239 TACTTCCTCCTTCTTTAACTCTTTTACTTTCTAGATCTATAGTAGAAAATGGAGCAGGAA 298

Query 318 CAGGTTGAACTGTATACCCTCCTCTTTCTTCTGGAACTGCTCATGCAGGAGCTTCTGTAG 377

| ||||||||||| || |||||||||||||||||||| |||||||| |||||||| || |

Sbjct 299 CTGGTTGAACTGTTTATCCTCCTCTTTCTTCTGGAACCGCTCATGCTGGAGCTTCAGTTG 358

Query 378 ATTTAGCTATTTTTTCTCTTCATTTAGCAGGAATTTCTTCTATTTTAGGAGCAGTAAATT 437

||||||||||||||||| | ||||||||||| ||||| ||||||||||||||||||||||

Sbjct 359 ATTTAGCTATTTTTTCTTTACATTTAGCAGGTATTTCATCTATTTTAGGAGCAGTAAATT 418

Query 438 TTATTACAACTGTAATTAATATACGATCAACTGGAATTACACTTGATCGTTTACCTTTAT 497

||||||||||||||||||||||||||||| ||||||||||||||||||| ||||||||||

Sbjct 419 TTATTACAACTGTAATTAATATACGATCAGCTGGAATTACACTTGATCGATTACCTTTAT 478

Query 498 TTGTCTGATCTGTAATTATTACAGCTATTTTATTACTTCTTTCATTACCAGTTTTAGCAG 557

|||| ||||| ||| | |||||||| ||||||||||||||||||||||| ||||||||||

Sbjct 479 TTGTTTGATCAGTAGTAATTACAGCAATTTTATTACTTCTTTCATTACCTGTTTTAGCAG 538

Query 558 GAGCTATTACTATATTATTAACTGATCGAAATTTTAATACATCATTTTTTGACCCAATTG 617

|||| || ||||| ||||||||||||||||||||||||||||||||||||||||||||||

Sbjct 539 GAGCAATCACTATGTTATTAACTGATCGAAATTTTAATACATCATTTTTTGACCCAATTG 598

Query 618 GAGGAGGTGACCCTATTCTT 637

|||||||||| |||||||||

Sbjct 599 GAGGAGGTGATCCTATTCTT 618

>Aedes polynesiensis voucher WA037 cytochrome c oxidase subunit I (COX1) gene, partial cds; mitochondrial

Sequence ID: MW664772.1 Length: 618

Range 1: 1 to 618

Score:915 bits(495), Expect:0.0,

Identities:579/620(93%), Gaps:3/620(0%), Strand: Plus/Plus

Query 19 TGATCGGGGA-AGTCGGAACTTCATTAAGAGTTTTAATTCGAACTGAACTTAGTCACCCT 77

||||| || | ||| |||||||| ||||||||||||||||||||||||||||||||||||

Sbjct 1 TGATCTGGAATAGTAGGAACTTCTTTAAGAGTTTTAATTCGAACTGAACTTAGTCACCCT 60

Query 78 GGAATATTTATCGGAAATGATCAAATTTATAATGTAATTCGTTACTAGCTCATGCATTTA 137

|| |||||||| |||||||| |||||||||||||||||| |||||| |||||||||||||

Sbjct 61 GGTATATTTATTGGAAATGACCAAATTTATAATGTAATT-GTTACT-GCTCATGCATTTA 118

Query 138 TTATAAttttttttATAGTAATACCTATTATAATTGGAGGATTTGGAAATTGGTTAGTCC 197

|||||||||||||||||||||||||||||||||||||||||||||||||||| ||||| |

Sbjct 119 TTATAATTTTTTTTATAGTAATACCTATTATAATTGGAGGATTTGGAAATTGATTAGTTC 178

Query 198 CATTAATATTAGGTGCTCCTGATATAGCTTTCCCTCGAATAAATAATATAAGTTTTTGAA 257

| ||||||||||| || |||||||||||||| ||||||||||||||||||||||||||||

Sbjct 179 CTTTAATATTAGGAGCCCCTGATATAGCTTTTCCTCGAATAAATAATATAAGTTTTTGAA 238

Query 258 TACTTCCTCCCTCTTTAACTCTTTTAATTTCTAGATCTATAGTAGAAAATGGAGCAGGAA 317

|||||||||| ||||||||||||||| |||||||||||||||||||||||||||||||||

Sbjct 239 TACTTCCTCCTTCTTTAACTCTTTTACTTTCTAGATCTATAGTAGAAAATGGAGCAGGAA 298

Query 318 CAGGTTGAACTGTATACCCTCCTCTTTCTTCTGGAACTGCTCATGCAGGAGCTTCTGTAG 377

| ||||||||||| || |||||||||||||||||||| |||||||| |||||||| || |

Sbjct 299 CTGGTTGAACTGTTTATCCTCCTCTTTCTTCTGGAACCGCTCATGCTGGAGCTTCAGTTG 358

Query 378 ATTTAGCTATTTTTTCTCTTCATTTAGCAGGAATTTCTTCTATTTTAGGAGCAGTAAATT 437

||||||||||||||||| | ||||||||||| ||||| ||||||||||||||||||||||

Sbjct 359 ATTTAGCTATTTTTTCTTTACATTTAGCAGGTATTTCATCTATTTTAGGAGCAGTAAATT 418

Query 438 TTATTACAACTGTAATTAATATACGATCAACTGGAATTACACTTGATCGTTTACCTTTAT 497

||||||||||||||||||||||||||||| ||||||||||||||||||| ||||||||||

Sbjct 419 TTATTACAACTGTAATTAATATACGATCAGCTGGAATTACACTTGATCGATTACCTTTAT 478

Query 498 TTGTCTGATCTGTAATTATTACAGCTATTTTATTACTTCTTTCATTACCAGTTTTAGCAG 557

|||| ||||| ||| | |||||||| ||||||||||||||||||||||| ||||||||||

Sbjct 479 TTGTTTGATCAGTAGTAATTACAGCAATTTTATTACTTCTTTCATTACCTGTTTTAGCAG 538

Query 558 GAGCTATTACTATATTATTAACTGATCGAAATTTTAATACATCATTTTTTGACCCAATTG 617

|||| || ||||| ||||||||||||||||||||||||||||||||||||||||||||||

Sbjct 539 GAGCAATCACTATGTTATTAACTGATCGAAATTTTAATACATCATTTTTTGACCCAATTG 598

Query 618 GAGGAGGTGACCCTATTCTT 637

|||||||||| |||||||||

Sbjct 599 GAGGAGGTGATCCTATTCTT 618

>Aedes polynesiensis voucher WA036 cytochrome c oxidase subunit I (COX1) gene, partial cds; mitochondrial

Sequence ID: MW664771.1 Length: 618

Range 1: 1 to 618

Score:915 bits(495), Expect:0.0,

Identities:579/620(93%), Gaps:3/620(0%), Strand: Plus/Plus

Query 19 TGATCGGGGA-AGTCGGAACTTCATTAAGAGTTTTAATTCGAACTGAACTTAGTCACCCT 77

||||| || | ||| |||||||| ||||||||||||||||||||||||||||||||||||

Sbjct 1 TGATCTGGAATAGTAGGAACTTCTTTAAGAGTTTTAATTCGAACTGAACTTAGTCACCCT 60

Query 78 GGAATATTTATCGGAAATGATCAAATTTATAATGTAATTCGTTACTAGCTCATGCATTTA 137

|| |||||||| |||||||| |||||||||||||||||| |||||| |||||||||||||

Sbjct 61 GGTATATTTATTGGAAATGACCAAATTTATAATGTAATT-GTTACT-GCTCATGCATTTA 118

Query 138 TTATAAttttttttATAGTAATACCTATTATAATTGGAGGATTTGGAAATTGGTTAGTCC 197

|||||||||||||||||||||||||||||||||||||||||||||||||||| ||||| |

Sbjct 119 TTATAATTTTTTTTATAGTAATACCTATTATAATTGGAGGATTTGGAAATTGATTAGTTC 178

Query 198 CATTAATATTAGGTGCTCCTGATATAGCTTTCCCTCGAATAAATAATATAAGTTTTTGAA 257

| ||||||||||| || |||||||||||||| ||||||||||||||||||||||||||||

Sbjct 179 CTTTAATATTAGGAGCCCCTGATATAGCTTTTCCTCGAATAAATAATATAAGTTTTTGAA 238

Query 258 TACTTCCTCCCTCTTTAACTCTTTTAATTTCTAGATCTATAGTAGAAAATGGAGCAGGAA 317

|||||||||| ||||||||||||||| |||||||||||||||||||||||||||||||||

Sbjct 239 TACTTCCTCCTTCTTTAACTCTTTTACTTTCTAGATCTATAGTAGAAAATGGAGCAGGAA 298

Query 318 CAGGTTGAACTGTATACCCTCCTCTTTCTTCTGGAACTGCTCATGCAGGAGCTTCTGTAG 377

| ||||||||||| || |||||||||||||||||||| |||||||| |||||||| || |

Sbjct 299 CTGGTTGAACTGTTTATCCTCCTCTTTCTTCTGGAACCGCTCATGCTGGAGCTTCAGTTG 358

Query 378 ATTTAGCTATTTTTTCTCTTCATTTAGCAGGAATTTCTTCTATTTTAGGAGCAGTAAATT 437

||||||||||||||||| | ||||||||||| ||||| ||||||||||||||||||||||

Sbjct 359 ATTTAGCTATTTTTTCTTTACATTTAGCAGGTATTTCATCTATTTTAGGAGCAGTAAATT 418

Query 438 TTATTACAACTGTAATTAATATACGATCAACTGGAATTACACTTGATCGTTTACCTTTAT 497

|||||||||||||||||||||| |||||| ||||||||||||||||||| ||||||||||

Sbjct 419 TTATTACAACTGTAATTAATATGCGATCAGCTGGAATTACACTTGATCGATTACCTTTAT 478

Query 498 TTGTCTGATCTGTAATTATTACAGCTATTTTATTACTTCTTTCATTACCAGTTTTAGCAG 557

|||| ||||| ||| | |||||||| ||||||||||||||||||||||| ||||||||||

Sbjct 479 TTGTTTGATCAGTAGTAATTACAGCAATTTTATTACTTCTTTCATTACCTGTTTTAGCAG 538

Query 558 GAGCTATTACTATATTATTAACTGATCGAAATTTTAATACATCATTTTTTGACCCAATTG 617

|||| || ||||||||||||||||||||||||||||||||||||||||||||||||||||

Sbjct 539 GAGCAATCACTATATTATTAACTGATCGAAATTTTAATACATCATTTTTTGACCCAATTG 598

Query 618 GAGGAGGTGACCCTATTCTT 637

|||||||||| |||||||||

Sbjct 599 GAGGAGGTGATCCTATTCTT 618

>Aedes pseudoscutellaris voucher FI328 cytochrome c oxidase subunit I (COX1) gene, partial cds; mitochondrial

Sequence ID: MW664801.1 Length: 618

Range 1: 1 to 618

Score:909 bits(492), Expect:0.0,

Identities:578/620(93%), Gaps:3/620(0%), Strand: Plus/Plus

Query 19 TGATCGGGGA-AGTCGGAACTTCATTAAGAGTTTTAATTCGAACTGAACTTAGTCACCCT 77

||||| || | ||| |||||||| ||||||||||||||||||||||||||||||||||||

Sbjct 1 TGATCTGGAATAGTAGGAACTTCTTTAAGAGTTTTAATTCGAACTGAACTTAGTCACCCT 60

Query 78 GGAATATTTATCGGAAATGATCAAATTTATAATGTAATTCGTTACTAGCTCATGCATTTA 137

|| |||||||| |||||||| |||||||||||||||||| |||||| |||||||||||||

Sbjct 61 GGTATATTTATTGGAAATGACCAAATTTATAATGTAATT-GTTACT-GCTCATGCATTTA 118

Query 138 TTATAAttttttttATAGTAATACCTATTATAATTGGAGGATTTGGAAATTGGTTAGTCC 197

|||||||||||||||||||||||||||||||||||||||||||||||||||| ||||| |

Sbjct 119 TTATAATTTTTTTTATAGTAATACCTATTATAATTGGAGGATTTGGAAATTGATTAGTTC 178

Query 198 CATTAATATTAGGTGCTCCTGATATAGCTTTCCCTCGAATAAATAATATAAGTTTTTGAA 257

| ||||||||||| || |||||||||||||| ||||||||||||||||||||||||||||

Sbjct 179 CTTTAATATTAGGAGCCCCTGATATAGCTTTTCCTCGAATAAATAATATAAGTTTTTGAA 238

Query 258 TACTTCCTCCCTCTTTAACTCTTTTAATTTCTAGATCTATAGTAGAAAATGGAGCAGGAA 317

|||||||||| ||||||||||||||| |||||||||||||||||||||||||||||||||

Sbjct 239 TACTTCCTCCTTCTTTAACTCTTTTACTTTCTAGATCTATAGTAGAAAATGGAGCAGGAA 298

Query 318 CAGGTTGAACTGTATACCCTCCTCTTTCTTCTGGAACTGCTCATGCAGGAGCTTCTGTAG 377

| ||||||||||| || |||||||||||||||||||| |||||||| |||||||| || |

Sbjct 299 CTGGTTGAACTGTTTATCCTCCTCTTTCTTCTGGAACCGCTCATGCTGGAGCTTCAGTTG 358

Query 378 ATTTAGCTATTTTTTCTCTTCATTTAGCAGGAATTTCTTCTATTTTAGGAGCAGTAAATT 437

||||||||||||||||| | ||||||||||| ||||| ||||||||||||||||||||||

Sbjct 359 ATTTAGCTATTTTTTCTTTACATTTAGCAGGTATTTCATCTATTTTAGGAGCAGTAAATT 418

Query 438 TTATTACAACTGTAATTAATATACGATCAACTGGAATTACACTTGATCGTTTACCTTTAT 497

||||||||||||||||||||||||||||| |||||||||| |||||||| ||||||||||

Sbjct 419 TTATTACAACTGTAATTAATATACGATCAGCTGGAATTACGCTTGATCGATTACCTTTAT 478

Query 498 TTGTCTGATCTGTAATTATTACAGCTATTTTATTACTTCTTTCATTACCAGTTTTAGCAG 557

|||| ||||| ||||| |||||||| ||||||||||||||||||||||| ||||||||||

Sbjct 479 TTGTTTGATCAGTAATAATTACAGCAATTTTATTACTTCTTTCATTACCTGTTTTAGCAG 538

Query 558 GAGCTATTACTATATTATTAACTGATCGAAATTTTAATACATCATTTTTTGACCCAATTG 617

|||| || ||||| |||||||||||||||||||||||||||||||||||||| |||||||

Sbjct 539 GAGCAATCACTATGTTATTAACTGATCGAAATTTTAATACATCATTTTTTGATCCAATTG 598

Query 618 GAGGAGGTGACCCTATTCTT 637

|||||||||| |||||||||

Sbjct 599 GAGGAGGTGATCCTATTCTT 618

>Aedes pseudoscutellaris voucher FI006 cytochrome c oxidase subunit I (COX1) gene, partial cds; mitochondrial

Sequence ID: MW664798.1 Length: 618

Range 1: 1 to 618

Score:909 bits(492), Expect:0.0,

Identities:578/620(93%), Gaps:3/620(0%), Strand: Plus/Plus

Query 19 TGATCGGGGA-AGTCGGAACTTCATTAAGAGTTTTAATTCGAACTGAACTTAGTCACCCT 77

||||| || | ||| |||||||| ||||||||||||||||||||||||||||||||||||

Sbjct 1 TGATCTGGAATAGTAGGAACTTCTTTAAGAGTTTTAATTCGAACTGAACTTAGTCACCCT 60

Query 78 GGAATATTTATCGGAAATGATCAAATTTATAATGTAATTCGTTACTAGCTCATGCATTTA 137

|| |||||||| |||||||| |||||||||||||||||| |||||| |||||||||||||

Sbjct 61 GGTATATTTATTGGAAATGACCAAATTTATAATGTAATT-GTTACT-GCTCATGCATTTA 118

Query 138 TTATAAttttttttATAGTAATACCTATTATAATTGGAGGATTTGGAAATTGGTTAGTCC 197

|||||||||||||||||||||||||||||||||||||||||||||||||||| ||||| |

Sbjct 119 TTATAATTTTTTTTATAGTAATACCTATTATAATTGGAGGATTTGGAAATTGATTAGTTC 178

Query 198 CATTAATATTAGGTGCTCCTGATATAGCTTTCCCTCGAATAAATAATATAAGTTTTTGAA 257

| ||||||||||| || |||||||||||||| ||||||||||||||||||||||||||||

Sbjct 179 CTTTAATATTAGGAGCCCCTGATATAGCTTTTCCTCGAATAAATAATATAAGTTTTTGAA 238

Query 258 TACTTCCTCCCTCTTTAACTCTTTTAATTTCTAGATCTATAGTAGAAAATGGAGCAGGAA 317

|||||||||| ||||||||||||||| |||||||||||||||||||||||||||||||||

Sbjct 239 TACTTCCTCCTTCTTTAACTCTTTTACTTTCTAGATCTATAGTAGAAAATGGAGCAGGAA 298

Query 318 CAGGTTGAACTGTATACCCTCCTCTTTCTTCTGGAACTGCTCATGCAGGAGCTTCTGTAG 377

| ||||||||||| || |||||||||||||||||||| |||||||| |||||||| || |

Sbjct 299 CTGGTTGAACTGTTTATCCTCCTCTTTCTTCTGGAACCGCTCATGCTGGAGCTTCAGTTG 358

Query 378 ATTTAGCTATTTTTTCTCTTCATTTAGCAGGAATTTCTTCTATTTTAGGAGCAGTAAATT 437

||||||||||||||||| | ||||||||||| ||||| ||||||||||||||||||||||

Sbjct 359 ATTTAGCTATTTTTTCTTTACATTTAGCAGGTATTTCATCTATTTTAGGAGCAGTAAATT 418

Query 438 TTATTACAACTGTAATTAATATACGATCAACTGGAATTACACTTGATCGTTTACCTTTAT 497

||||||||||||||||||||||||||||| |||||||||| |||||||| ||||||||||

Sbjct 419 TTATTACAACTGTAATTAATATACGATCAGCTGGAATTACGCTTGATCGATTACCTTTAT 478

Query 498 TTGTCTGATCTGTAATTATTACAGCTATTTTATTACTTCTTTCATTACCAGTTTTAGCAG 557

|||| ||||| ||||| |||||||| ||||||||||||||||||||||| ||||||||||

Sbjct 479 TTGTTTGATCAGTAATAATTACAGCAATTTTATTACTTCTTTCATTACCTGTTTTAGCAG 538

Query 558 GAGCTATTACTATATTATTAACTGATCGAAATTTTAATACATCATTTTTTGACCCAATTG 617

|||| || ||||| |||||||||||||||||||||||||||||||||||||| |||||||

Sbjct 539 GAGCAATCACTATGTTATTAACTGATCGAAATTTTAATACATCATTTTTTGATCCAATTG 598

Query 618 GAGGAGGTGACCCTATTCTT 637

|||||||||| |||||||||

Sbjct 599 GAGGAGGTGATCCTATTCTT 618

>Aedes polynesiensis voucher WA069 cytochrome c oxidase subunit I (COX1) gene, partial cds; mitochondrial

Sequence ID: MW664788.1 Length: 618

Range 1: 1 to 618

Score:909 bits(492), Expect:0.0,

Identities:578/620(93%), Gaps:3/620(0%), Strand: Plus/Plus

Query 19 TGATCGGGGA-AGTCGGAACTTCATTAAGAGTTTTAATTCGAACTGAACTTAGTCACCCT 77

||||| || | ||| |||||||| ||||||||||||||||||||||||||||||||||||

Sbjct 1 TGATCTGGAATAGTAGGAACTTCTTTAAGAGTTTTAATTCGAACTGAACTTAGTCACCCT 60

Query 78 GGAATATTTATCGGAAATGATCAAATTTATAATGTAATTCGTTACTAGCTCATGCATTTA 137

|| |||||||| |||||||| |||||||||||||||||| |||||| |||||||||||||

Sbjct 61 GGTATATTTATTGGAAATGACCAAATTTATAATGTAATT-GTTACT-GCTCATGCATTTA 118

Query 138 TTATAAttttttttATAGTAATACCTATTATAATTGGAGGATTTGGAAATTGGTTAGTCC 197

|||||||||||||||||||||||||||||||||||||||||||||||||||| ||||| |

Sbjct 119 TTATAATTTTTTTTATAGTAATACCTATTATAATTGGAGGATTTGGAAATTGATTAGTTC 178

Query 198 CATTAATATTAGGTGCTCCTGATATAGCTTTCCCTCGAATAAATAATATAAGTTTTTGAA 257

| ||||||||||| || |||||||||||||| ||||||||||||||||||||||||||||

Sbjct 179 CTTTAATATTAGGAGCCCCTGATATAGCTTTTCCTCGAATAAATAATATAAGTTTTTGAA 238

Query 258 TACTTCCTCCCTCTTTAACTCTTTTAATTTCTAGATCTATAGTAGAAAATGGAGCAGGAA 317

|||||||||| ||||||||||||||| |||||||||||||||||||||||||||||||||

Sbjct 239 TACTTCCTCCTTCTTTAACTCTTTTACTTTCTAGATCTATAGTAGAAAATGGAGCAGGAA 298

Query 318 CAGGTTGAACTGTATACCCTCCTCTTTCTTCTGGAACTGCTCATGCAGGAGCTTCTGTAG 377

| ||||||||||| || |||||||||||||||||||| |||||||| || ||||| || |

Sbjct 299 CTGGTTGAACTGTTTATCCTCCTCTTTCTTCTGGAACCGCTCATGCTGGGGCTTCAGTTG 358

Query 378 ATTTAGCTATTTTTTCTCTTCATTTAGCAGGAATTTCTTCTATTTTAGGAGCAGTAAATT 437

||||||||||||||||| | ||||||||||| ||||| ||||||||||||||||||||||

Sbjct 359 ATTTAGCTATTTTTTCTTTACATTTAGCAGGTATTTCATCTATTTTAGGAGCAGTAAATT 418

Query 438 TTATTACAACTGTAATTAATATACGATCAACTGGAATTACACTTGATCGTTTACCTTTAT 497

||||||||||||||||||||||||||||| ||||||||||||||||||| ||||||||||

Sbjct 419 TTATTACAACTGTAATTAATATACGATCAGCTGGAATTACACTTGATCGATTACCTTTAT 478

Query 498 TTGTCTGATCTGTAATTATTACAGCTATTTTATTACTTCTTTCATTACCAGTTTTAGCAG 557

|||| ||||| ||| | |||||||| ||||||||||||||||||||||| ||||||||||

Sbjct 479 TTGTTTGATCAGTAGTAATTACAGCAATTTTATTACTTCTTTCATTACCTGTTTTAGCAG 538

Query 558 GAGCTATTACTATATTATTAACTGATCGAAATTTTAATACATCATTTTTTGACCCAATTG 617

|||| || ||||| ||||||||||||||||||||||||||||||||||||||||||||||

Sbjct 539 GAGCAATCACTATGTTATTAACTGATCGAAATTTTAATACATCATTTTTTGACCCAATTG 598

Query 618 GAGGAGGTGACCCTATTCTT 637

|||||||||| |||||||||

Sbjct 599 GAGGAGGTGATCCTATTCTT 618

>Aedes polynesiensis voucher WA064 cytochrome c oxidase subunit I (COX1) gene, partial cds; mitochondrial

Sequence ID: MW664784.1 Length: 618

Range 1: 1 to 618

Score:909 bits(492), Expect:0.0,

Identities:578/620(93%), Gaps:3/620(0%), Strand: Plus/Plus

Query 19 TGATCGGGGA-AGTCGGAACTTCATTAAGAGTTTTAATTCGAACTGAACTTAGTCACCCT 77

||||| || | ||| |||||||| ||||||||||||||||||||||||||||||||||||

Sbjct 1 TGATCTGGAATAGTAGGAACTTCTTTAAGAGTTTTAATTCGAACTGAACTTAGTCACCCT 60

Query 78 GGAATATTTATCGGAAATGATCAAATTTATAATGTAATTCGTTACTAGCTCATGCATTTA 137

|| |||||||| |||||||| |||||||||||||||||| |||||| |||||||||||||

Sbjct 61 GGTATATTTATTGGAAATGACCAAATTTATAATGTAATT-GTTACT-GCTCATGCATTTA 118

Query 138 TTATAAttttttttATAGTAATACCTATTATAATTGGAGGATTTGGAAATTGGTTAGTCC 197

|||||||||||||||||||||||||||||||||||||||||||||||||||| ||||| |

Sbjct 119 TTATAATTTTTTTTATAGTAATACCTATTATAATTGGAGGATTTGGAAATTGATTAGTTC 178

Query 198 CATTAATATTAGGTGCTCCTGATATAGCTTTCCCTCGAATAAATAATATAAGTTTTTGAA 257

| ||||||||||| || |||||||||||||| ||||||||||||||||||||||||||||

Sbjct 179 CTTTAATATTAGGAGCCCCTGATATAGCTTTTCCTCGAATAAATAATATAAGTTTTTGAA 238

Query 258 TACTTCCTCCCTCTTTAACTCTTTTAATTTCTAGATCTATAGTAGAAAATGGAGCAGGAA 317

|||||||||| ||||||||||||||| |||||||||||||||||||||||||||||||||

Sbjct 239 TACTTCCTCCTTCTTTAACTCTTTTACTTTCTAGATCTATAGTAGAAAATGGAGCAGGAA 298

Query 318 CAGGTTGAACTGTATACCCTCCTCTTTCTTCTGGAACTGCTCATGCAGGAGCTTCTGTAG 377

| ||||||||||| || |||||||||||||||||||| |||||||| || ||||| || |

Sbjct 299 CTGGTTGAACTGTTTATCCTCCTCTTTCTTCTGGAACCGCTCATGCTGGGGCTTCAGTTG 358

Query 378 ATTTAGCTATTTTTTCTCTTCATTTAGCAGGAATTTCTTCTATTTTAGGAGCAGTAAATT 437

||||||||||||||||| | ||||||||||| ||||| ||||||||||||||||||||||

Sbjct 359 ATTTAGCTATTTTTTCTTTACATTTAGCAGGTATTTCATCTATTTTAGGAGCAGTAAATT 418

Query 438 TTATTACAACTGTAATTAATATACGATCAACTGGAATTACACTTGATCGTTTACCTTTAT 497

||||||||||||||||||||||||||||| ||||||||||||||||||| ||||||||||

Sbjct 419 TTATTACAACTGTAATTAATATACGATCAGCTGGAATTACACTTGATCGATTACCTTTAT 478

Query 498 TTGTCTGATCTGTAATTATTACAGCTATTTTATTACTTCTTTCATTACCAGTTTTAGCAG 557

|||| ||||| ||| | |||||||| ||||||||||||||||||||||| ||||||||||

Sbjct 479 TTGTTTGATCAGTAGTAATTACAGCAATTTTATTACTTCTTTCATTACCTGTTTTAGCAG 538

Query 558 GAGCTATTACTATATTATTAACTGATCGAAATTTTAATACATCATTTTTTGACCCAATTG 617

|||| || ||||| ||||||||||||||||||||||||||||||||||||||||||||||

Sbjct 539 GAGCAATCACTATGTTATTAACTGATCGAAATTTTAATACATCATTTTTTGACCCAATTG 598

Query 618 GAGGAGGTGACCCTATTCTT 637

|||||||||| |||||||||

Sbjct 599 GAGGAGGTGATCCTATTCTT 618

>Aedes polynesiensis voucher WA060 cytochrome c oxidase subunit I (COX1) gene, partial cds; mitochondrial

Sequence ID: MW664781.1 Length: 618

Range 1: 1 to 618

Score:909 bits(492), Expect:0.0,

Identities:578/620(93%), Gaps:3/620(0%), Strand: Plus/Plus

Query 19 TGATCGGGGA-AGTCGGAACTTCATTAAGAGTTTTAATTCGAACTGAACTTAGTCACCCT 77

||||| || | ||| |||||||| ||||||||||||||||||||||||||||||||||||

Sbjct 1 TGATCTGGAATAGTAGGAACTTCTTTAAGAGTTTTAATTCGAACTGAACTTAGTCACCCT 60

Query 78 GGAATATTTATCGGAAATGATCAAATTTATAATGTAATTCGTTACTAGCTCATGCATTTA 137

|| |||||||| |||||||| |||||||||||||||||| |||||| |||||||||||||

Sbjct 61 GGTATATTTATTGGAAATGACCAAATTTATAATGTAATT-GTTACT-GCTCATGCATTTA 118

Query 138 TTATAAttttttttATAGTAATACCTATTATAATTGGAGGATTTGGAAATTGGTTAGTCC 197

|||||||||||||||||||||||||||||||||||||||||||||||||||| ||||| |

Sbjct 119 TTATAATTTTTTTTATAGTAATACCTATTATAATTGGAGGATTTGGAAATTGATTAGTTC 178

Query 198 CATTAATATTAGGTGCTCCTGATATAGCTTTCCCTCGAATAAATAATATAAGTTTTTGAA 257

| ||||||||||| || |||||||||||||| ||||||||||||||||||||||||||||

Sbjct 179 CTTTAATATTAGGAGCCCCTGATATAGCTTTTCCTCGAATAAATAATATAAGTTTTTGAA 238

Query 258 TACTTCCTCCCTCTTTAACTCTTTTAATTTCTAGATCTATAGTAGAAAATGGAGCAGGAA 317

|||||||||| ||||||||||||||| |||||||||||||||||||||||||||||||||

Sbjct 239 TACTTCCTCCTTCTTTAACTCTTTTACTTTCTAGATCTATAGTAGAAAATGGAGCAGGAA 298

Query 318 CAGGTTGAACTGTATACCCTCCTCTTTCTTCTGGAACTGCTCATGCAGGAGCTTCTGTAG 377

| ||||||||||| || |||||||||||||||||||| |||||||| |||||||| || |

Sbjct 299 CTGGTTGAACTGTTTATCCTCCTCTTTCTTCTGGAACCGCTCATGCTGGAGCTTCAGTTG 358

Query 378 ATTTAGCTATTTTTTCTCTTCATTTAGCAGGAATTTCTTCTATTTTAGGAGCAGTAAATT 437

||||||||||||||||| | ||||||||||| ||||| ||||||||||||||||||||||

Sbjct 359 ATTTAGCTATTTTTTCTTTACATTTAGCAGGTATTTCATCTATTTTAGGAGCAGTAAATT 418

Query 438 TTATTACAACTGTAATTAATATACGATCAACTGGAATTACACTTGATCGTTTACCTTTAT 497

||||||||||||||||||||||||||||| ||||||||||||||||||| ||||||||||

Sbjct 419 TTATTACAACTGTAATTAATATACGATCAGCTGGAATTACACTTGATCGATTACCTTTAT 478

Query 498 TTGTCTGATCTGTAATTATTACAGCTATTTTATTACTTCTTTCATTACCAGTTTTAGCAG 557

|||| ||||| ||| | |||||||| ||||||||||||||||||||||| ||||||||||

Sbjct 479 TTGTTTGATCAGTAGTAATTACAGCAATTTTATTACTTCTTTCATTACCTGTTTTAGCAG 538

Query 558 GAGCTATTACTATATTATTAACTGATCGAAATTTTAATACATCATTTTTTGACCCAATTG 617

|||| || ||||| ||||||||||||||||||||||||||||||| ||||||||||||||

Sbjct 539 GAGCAATCACTATGTTATTAACTGATCGAAATTTTAATACATCATCTTTTGACCCAATTG 598

Query 618 GAGGAGGTGACCCTATTCTT 637

|||||||||| |||||||||

Sbjct 599 GAGGAGGTGATCCTATTCTT 618

>Aedes polynesiensis voucher WA054 cytochrome c oxidase subunit I (COX1) gene, partial cds; mitochondrial

Sequence ID: MW664779.1 Length: 618

Range 1: 1 to 618

Score:909 bits(492), Expect:0.0,

Identities:578/620(93%), Gaps:3/620(0%), Strand: Plus/Plus

Query 19 TGATCGGGGA-AGTCGGAACTTCATTAAGAGTTTTAATTCGAACTGAACTTAGTCACCCT 77

||||| || | ||| |||||||| ||||||||||||||||||||||||||||||||||||

Sbjct 1 TGATCTGGAATAGTAGGAACTTCTTTAAGAGTTTTAATTCGAACTGAACTTAGTCACCCT 60

Query 78 GGAATATTTATCGGAAATGATCAAATTTATAATGTAATTCGTTACTAGCTCATGCATTTA 137

|| |||||||| |||||||| |||||||||||||||||| |||||| |||||||||||||

Sbjct 61 GGTATATTTATTGGAAATGACCAAATTTATAATGTAATT-GTTACT-GCTCATGCATTTA 118

Query 138 TTATAAttttttttATAGTAATACCTATTATAATTGGAGGATTTGGAAATTGGTTAGTCC 197

|||||||||||||||||||||||||||||||||||||||||||||||||||| ||||| |

Sbjct 119 TTATAATTTTTTTTATAGTAATACCTATTATAATTGGAGGATTTGGAAATTGATTAGTTC 178

Query 198 CATTAATATTAGGTGCTCCTGATATAGCTTTCCCTCGAATAAATAATATAAGTTTTTGAA 257

| ||||||||||| || |||||||||||||| ||||||||||||||||||||||||||||

Sbjct 179 CTTTAATATTAGGAGCCCCTGATATAGCTTTTCCTCGAATAAATAATATAAGTTTTTGAA 238

Query 258 TACTTCCTCCCTCTTTAACTCTTTTAATTTCTAGATCTATAGTAGAAAATGGAGCAGGAA 317

|||||||||| ||||||||||||||| |||||||||||||||||||||||||||||||||

Sbjct 239 TACTTCCTCCTTCTTTAACTCTTTTACTTTCTAGATCTATAGTAGAAAATGGAGCAGGAA 298

Query 318 CAGGTTGAACTGTATACCCTCCTCTTTCTTCTGGAACTGCTCATGCAGGAGCTTCTGTAG 377

| ||||||||||| || |||||||||||||||||||| |||||||| |||||||| || |

Sbjct 299 CTGGTTGAACTGTTTATCCTCCTCTTTCTTCTGGAACCGCTCATGCTGGAGCTTCAGTTG 358

Query 378 ATTTAGCTATTTTTTCTCTTCATTTAGCAGGAATTTCTTCTATTTTAGGAGCAGTAAATT 437

||||||||||||||||| | ||||||||||| || || ||||||||||||||||||||||

Sbjct 359 ATTTAGCTATTTTTTCTTTACATTTAGCAGGTATCTCATCTATTTTAGGAGCAGTAAATT 418

Query 438 TTATTACAACTGTAATTAATATACGATCAACTGGAATTACACTTGATCGTTTACCTTTAT 497

||||||||||||||||||||||||||||| ||||||||||||||||||| ||||||||||

Sbjct 419 TTATTACAACTGTAATTAATATACGATCAGCTGGAATTACACTTGATCGATTACCTTTAT 478

Query 498 TTGTCTGATCTGTAATTATTACAGCTATTTTATTACTTCTTTCATTACCAGTTTTAGCAG 557

|||| ||||| ||| | |||||||| ||||||||||||||||||||||| ||||||||||

Sbjct 479 TTGTTTGATCAGTAGTAATTACAGCAATTTTATTACTTCTTTCATTACCTGTTTTAGCAG 538

Query 558 GAGCTATTACTATATTATTAACTGATCGAAATTTTAATACATCATTTTTTGACCCAATTG 617

|||| || ||||| ||||||||||||||||||||||||||||||||||||||||||||||

Sbjct 539 GAGCAATCACTATGTTATTAACTGATCGAAATTTTAATACATCATTTTTTGACCCAATTG 598

Query 618 GAGGAGGTGACCCTATTCTT 637

|||||||||| |||||||||

Sbjct 599 GAGGAGGTGATCCTATTCTT 618

>Aedes polynesiensis voucher WA053 cytochrome c oxidase subunit I (COX1) gene, partial cds; mitochondrial

Sequence ID: MW664778.1 Length: 618

Range 1: 1 to 618

Score:909 bits(492), Expect:0.0,

Identities:578/620(93%), Gaps:3/620(0%), Strand: Plus/Plus

Query 19 TGATCGGGGA-AGTCGGAACTTCATTAAGAGTTTTAATTCGAACTGAACTTAGTCACCCT 77

||||| || | ||| |||||||| ||||||||||||||||||||||||||||||||||||

Sbjct 1 TGATCTGGAATAGTAGGAACTTCTTTAAGAGTTTTAATTCGAACTGAACTTAGTCACCCT 60

Query 78 GGAATATTTATCGGAAATGATCAAATTTATAATGTAATTCGTTACTAGCTCATGCATTTA 137

|| |||||||| |||||||| |||||||||||||||||| |||||| |||||||||||||

Sbjct 61 GGTATATTTATTGGAAATGACCAAATTTATAATGTAATT-GTTACT-GCTCATGCATTTA 118

Query 138 TTATAAttttttttATAGTAATACCTATTATAATTGGAGGATTTGGAAATTGGTTAGTCC 197

|||||||||||||||||||||||||||||||||||||||||||||||||||| ||||| |

Sbjct 119 TTATAATTTTTTTTATAGTAATACCTATTATAATTGGAGGATTTGGAAATTGATTAGTTC 178

Query 198 CATTAATATTAGGTGCTCCTGATATAGCTTTCCCTCGAATAAATAATATAAGTTTTTGAA 257

| ||||||||||| || |||||||||||||| ||||||||||||||||||||||||||||

Sbjct 179 CTTTAATATTAGGAGCCCCTGATATAGCTTTTCCTCGAATAAATAATATAAGTTTTTGAA 238

Query 258 TACTTCCTCCCTCTTTAACTCTTTTAATTTCTAGATCTATAGTAGAAAATGGAGCAGGAA 317

|||||||||| ||||||||||||||| |||||||||||||||||||||||||||||||||

Sbjct 239 TACTTCCTCCTTCTTTAACTCTTTTACTTTCTAGATCTATAGTAGAAAATGGAGCAGGAA 298

Query 318 CAGGTTGAACTGTATACCCTCCTCTTTCTTCTGGAACTGCTCATGCAGGAGCTTCTGTAG 377

| ||||||||||| || |||||||||||||||||||| |||||||| || ||||| || |

Sbjct 299 CTGGTTGAACTGTTTATCCTCCTCTTTCTTCTGGAACCGCTCATGCTGGGGCTTCAGTTG 358

Query 378 ATTTAGCTATTTTTTCTCTTCATTTAGCAGGAATTTCTTCTATTTTAGGAGCAGTAAATT 437

||||||||||||||||| | ||||||||||| ||||| ||||||||||||||||||||||

Sbjct 359 ATTTAGCTATTTTTTCTTTACATTTAGCAGGTATTTCATCTATTTTAGGAGCAGTAAATT 418

Query 438 TTATTACAACTGTAATTAATATACGATCAACTGGAATTACACTTGATCGTTTACCTTTAT 497

||||||||||||||||||||||||||||| ||||||||||||||||||| ||||||||||

Sbjct 419 TTATTACAACTGTAATTAATATACGATCAGCTGGAATTACACTTGATCGATTACCTTTAT 478

Query 498 TTGTCTGATCTGTAATTATTACAGCTATTTTATTACTTCTTTCATTACCAGTTTTAGCAG 557

|||| ||||| ||| | |||||||| ||||||||||||||||||||||| ||||||||||

Sbjct 479 TTGTTTGATCAGTAGTAATTACAGCAATTTTATTACTTCTTTCATTACCTGTTTTAGCAG 538

Query 558 GAGCTATTACTATATTATTAACTGATCGAAATTTTAATACATCATTTTTTGACCCAATTG 617

|||| || ||||| ||||||||||||||||||||||||||||||||||||||||||||||

Sbjct 539 GAGCAATCACTATGTTATTAACTGATCGAAATTTTAATACATCATTTTTTGACCCAATTG 598

Query 618 GAGGAGGTGACCCTATTCTT 637

|||||||||| |||||||||

Sbjct 599 GAGGAGGTGATCCTATTCTT 618

>Aedes polynesiensis voucher WA048 cytochrome c oxidase subunit I (COX1) gene, partial cds; mitochondrial

Sequence ID: MW664777.1 Length: 618

Range 1: 1 to 618

Score:909 bits(492), Expect:0.0,

Identities:578/620(93%), Gaps:3/620(0%), Strand: Plus/Plus

Query 19 TGATCGGGGA-AGTCGGAACTTCATTAAGAGTTTTAATTCGAACTGAACTTAGTCACCCT 77

||||| || | ||| |||||||| ||||||||||||||||||||||||||||||||||||

Sbjct 1 TGATCTGGAATAGTAGGAACTTCTTTAAGAGTTTTAATTCGAACTGAACTTAGTCACCCT 60

Query 78 GGAATATTTATCGGAAATGATCAAATTTATAATGTAATTCGTTACTAGCTCATGCATTTA 137

|| |||||||| |||||||| |||||||||||||||||| |||||| |||||||||||||

Sbjct 61 GGTATATTTATTGGAAATGACCAAATTTATAATGTAATT-GTTACT-GCTCATGCATTTA 118

Query 138 TTATAAttttttttATAGTAATACCTATTATAATTGGAGGATTTGGAAATTGGTTAGTCC 197

|||||||||||||||||||||||||||||||||||||||||||||||||||| ||||| |

Sbjct 119 TTATAATTTTTTTTATAGTAATACCTATTATAATTGGAGGATTTGGAAATTGATTAGTTC 178

Query 198 CATTAATATTAGGTGCTCCTGATATAGCTTTCCCTCGAATAAATAATATAAGTTTTTGAA 257

| ||||||||||| || |||||||||||||| ||||||||||||||||||||||||||||

Sbjct 179 CTTTAATATTAGGAGCCCCTGATATAGCTTTTCCTCGAATAAATAATATAAGTTTTTGAA 238

Query 258 TACTTCCTCCCTCTTTAACTCTTTTAATTTCTAGATCTATAGTAGAAAATGGAGCAGGAA 317

|||||||||| ||||||||||||||| |||||||||||||||||||||||||||||||||

Sbjct 239 TACTTCCTCCTTCTTTAACTCTTTTACTTTCTAGATCTATAGTAGAAAATGGAGCAGGAA 298

Query 318 CAGGTTGAACTGTATACCCTCCTCTTTCTTCTGGAACTGCTCATGCAGGAGCTTCTGTAG 377

| ||||||||||| || |||||||||||||||||||| |||||||| |||||||| || |

Sbjct 299 CTGGTTGAACTGTTTATCCTCCTCTTTCTTCTGGAACCGCTCATGCTGGAGCTTCAGTTG 358

Query 378 ATTTAGCTATTTTTTCTCTTCATTTAGCAGGAATTTCTTCTATTTTAGGAGCAGTAAATT 437

||||||||||||||||| | ||||||||||| || || ||||||||||||||||||||||

Sbjct 359 ATTTAGCTATTTTTTCTTTACATTTAGCAGGTATCTCATCTATTTTAGGAGCAGTAAATT 418

Query 438 TTATTACAACTGTAATTAATATACGATCAACTGGAATTACACTTGATCGTTTACCTTTAT 497

||||||||||||||||||||||||||||| ||||||||||||||||||| ||||||||||

Sbjct 419 TTATTACAACTGTAATTAATATACGATCAGCTGGAATTACACTTGATCGATTACCTTTAT 478

Query 498 TTGTCTGATCTGTAATTATTACAGCTATTTTATTACTTCTTTCATTACCAGTTTTAGCAG 557

|||| ||||| ||| | |||||||| ||||||||||||||||||||||| ||||||||||

Sbjct 479 TTGTTTGATCAGTAGTAATTACAGCAATTTTATTACTTCTTTCATTACCTGTTTTAGCAG 538

Query 558 GAGCTATTACTATATTATTAACTGATCGAAATTTTAATACATCATTTTTTGACCCAATTG 617

|||| || ||||| ||||||||||||||||||||||||||||||||||||||||||||||

Sbjct 539 GAGCAATCACTATGTTATTAACTGATCGAAATTTTAATACATCATTTTTTGACCCAATTG 598

Query 618 GAGGAGGTGACCCTATTCTT 637

|||||||||| |||||||||

Sbjct 599 GAGGAGGTGATCCTATTCTT 618

>Aedes polynesiensis voucher WA046 cytochrome c oxidase subunit I (COX1) gene, partial cds; mitochondrial

Sequence ID: MW664776.1 Length: 618

Range 1: 1 to 618

Score:909 bits(492), Expect:0.0,

Identities:578/620(93%), Gaps:3/620(0%), Strand: Plus/Plus

Query 19 TGATCGGGGA-AGTCGGAACTTCATTAAGAGTTTTAATTCGAACTGAACTTAGTCACCCT 77

||||| || | ||| |||||||| ||||||||||||||||||||||||||||||||||||

Sbjct 1 TGATCTGGAATAGTAGGAACTTCTTTAAGAGTTTTAATTCGAACTGAACTTAGTCACCCT 60

Query 78 GGAATATTTATCGGAAATGATCAAATTTATAATGTAATTCGTTACTAGCTCATGCATTTA 137

|| |||||||| |||||||| |||||||||||||||||| |||||| |||||||||||||

Sbjct 61 GGTATATTTATTGGAAATGACCAAATTTATAATGTAATT-GTTACT-GCTCATGCATTTA 118

Query 138 TTATAAttttttttATAGTAATACCTATTATAATTGGAGGATTTGGAAATTGGTTAGTCC 197

|||||||||||||||||||||||||||||||||||||||||||||||||||| ||||| |

Sbjct 119 TTATAATTTTTTTTATAGTAATACCTATTATAATTGGAGGATTTGGAAATTGATTAGTTC 178

Query 198 CATTAATATTAGGTGCTCCTGATATAGCTTTCCCTCGAATAAATAATATAAGTTTTTGAA 257

| ||||||||||| || |||||||||||||| ||||||||||||||||||||||||||||

Sbjct 179 CTTTAATATTAGGAGCCCCTGATATAGCTTTTCCTCGAATAAATAATATAAGTTTTTGAA 238

Query 258 TACTTCCTCCCTCTTTAACTCTTTTAATTTCTAGATCTATAGTAGAAAATGGAGCAGGAA 317

|||||||||| ||||||||||||||| |||||||||||||||||||||||||||||||||

Sbjct 239 TACTTCCTCCTTCTTTAACTCTTTTACTTTCTAGATCTATAGTAGAAAATGGAGCAGGAA 298

Query 318 CAGGTTGAACTGTATACCCTCCTCTTTCTTCTGGAACTGCTCATGCAGGAGCTTCTGTAG 377

| ||||||||||| || |||||||||||||||||||| |||||||| || ||||| || |

Sbjct 299 CTGGTTGAACTGTTTATCCTCCTCTTTCTTCTGGAACCGCTCATGCTGGGGCTTCAGTTG 358

Query 378 ATTTAGCTATTTTTTCTCTTCATTTAGCAGGAATTTCTTCTATTTTAGGAGCAGTAAATT 437

||||||||||||||||| | ||||||||||| ||||| ||||||||||||||||||||||

Sbjct 359 ATTTAGCTATTTTTTCTTTACATTTAGCAGGTATTTCATCTATTTTAGGAGCAGTAAATT 418

Query 438 TTATTACAACTGTAATTAATATACGATCAACTGGAATTACACTTGATCGTTTACCTTTAT 497

||||||||||||||||||||||||||||| ||||||||||||||||||| ||||||||||

Sbjct 419 TTATTACAACTGTAATTAATATACGATCAGCTGGAATTACACTTGATCGATTACCTTTAT 478

Query 498 TTGTCTGATCTGTAATTATTACAGCTATTTTATTACTTCTTTCATTACCAGTTTTAGCAG 557

|||| ||||| ||| | |||||||| ||||||||||||||||||||||| ||||||||||

Sbjct 479 TTGTTTGATCAGTAGTAATTACAGCAATTTTATTACTTCTTTCATTACCTGTTTTAGCAG 538

Query 558 GAGCTATTACTATATTATTAACTGATCGAAATTTTAATACATCATTTTTTGACCCAATTG 617

|||| || ||||| ||||||||||||||||||||||||||||||||||||||||||||||

Sbjct 539 GAGCAATCACTATGTTATTAACTGATCGAAATTTTAATACATCATTTTTTGACCCAATTG 598

Query 618 GAGGAGGTGACCCTATTCTT 637

|||||||||| |||||||||

Sbjct 599 GAGGAGGTGATCCTATTCTT 618

>Aedes polynesiensis voucher WA034 cytochrome c oxidase subunit I (COX1) gene, partial cds; mitochondrial

Sequence ID: MW664770.1 Length: 618

Range 1: 1 to 618

Score:909 bits(492), Expect:0.0,

Identities:578/620(93%), Gaps:3/620(0%), Strand: Plus/Plus

Query 19 TGATCGGGGA-AGTCGGAACTTCATTAAGAGTTTTAATTCGAACTGAACTTAGTCACCCT 77

||||| || | ||| |||||||| ||||||||||||||||||||||||||||||||||||

Sbjct 1 TGATCTGGAATAGTAGGAACTTCTTTAAGAGTTTTAATTCGAACTGAACTTAGTCACCCT 60

Query 78 GGAATATTTATCGGAAATGATCAAATTTATAATGTAATTCGTTACTAGCTCATGCATTTA 137

|| |||||||| |||||||| |||||||||||||||||| |||||| |||||||||||||

Sbjct 61 GGTATATTTATTGGAAATGACCAAATTTATAATGTAATT-GTTACT-GCTCATGCATTTA 118

Query 138 TTATAAttttttttATAGTAATACCTATTATAATTGGAGGATTTGGAAATTGGTTAGTCC 197

|||||||||||||||||||||||||||||||||||||||||||||||||||| ||||| |

Sbjct 119 TTATAATTTTTTTTATAGTAATACCTATTATAATTGGAGGATTTGGAAATTGATTAGTTC 178

Query 198 CATTAATATTAGGTGCTCCTGATATAGCTTTCCCTCGAATAAATAATATAAGTTTTTGAA 257

| ||||||||||| || |||||||||||||| ||||||||||||||||||||||||||||

Sbjct 179 CTTTAATATTAGGAGCCCCTGATATAGCTTTTCCTCGAATAAATAATATAAGTTTTTGAA 238

Query 258 TACTTCCTCCCTCTTTAACTCTTTTAATTTCTAGATCTATAGTAGAAAATGGAGCAGGAA 317

|||||||||| ||||||||||||||| |||||||||||||||||||||||||||||||||

Sbjct 239 TACTTCCTCCTTCTTTAACTCTTTTACTTTCTAGATCTATAGTAGAAAATGGAGCAGGAA 298

Query 318 CAGGTTGAACTGTATACCCTCCTCTTTCTTCTGGAACTGCTCATGCAGGAGCTTCTGTAG 377

| ||||||||||| || |||||||||||||||||||| |||||||| |||||||| || |

Sbjct 299 CTGGTTGAACTGTTTATCCTCCTCTTTCTTCTGGAACCGCTCATGCTGGAGCTTCAGTTG 358

Query 378 ATTTAGCTATTTTTTCTCTTCATTTAGCAGGAATTTCTTCTATTTTAGGAGCAGTAAATT 437

||||||||||||||||| | ||||||||||| ||||| ||||||||||||||||||||||

Sbjct 359 ATTTAGCTATTTTTTCTTTACATTTAGCAGGTATTTCATCTATTTTAGGAGCAGTAAATT 418

Query 438 TTATTACAACTGTAATTAATATACGATCAACTGGAATTACACTTGATCGTTTACCTTTAT 497

|||||||||||||||||||||| |||||| ||||||||||||||||||| ||||||||||

Sbjct 419 TTATTACAACTGTAATTAATATGCGATCAGCTGGAATTACACTTGATCGATTACCTTTAT 478

Query 498 TTGTCTGATCTGTAATTATTACAGCTATTTTATTACTTCTTTCATTACCAGTTTTAGCAG 557

|||| ||||| ||| | |||||||| ||||||||||||||||||||||| ||||||||||

Sbjct 479 TTGTTTGATCAGTAGTAATTACAGCAATTTTATTACTTCTTTCATTACCTGTTTTAGCAG 538

Query 558 GAGCTATTACTATATTATTAACTGATCGAAATTTTAATACATCATTTTTTGACCCAATTG 617

|||| || ||||| ||||||||||||||||||||||||||||||||||||||||||||||

Sbjct 539 GAGCAATCACTATGTTATTAACTGATCGAAATTTTAATACATCATTTTTTGACCCAATTG 598

Query 618 GAGGAGGTGACCCTATTCTT 637

|||||||||| |||||||||

Sbjct 599 GAGGAGGTGATCCTATTCTT 618

>Aedes polynesiensis voucher WA033 cytochrome c oxidase subunit I (COX1) gene, partial cds; mitochondrial

Sequence ID: MW664769.1 Length: 618

Range 1: 1 to 618

Score:909 bits(492), Expect:0.0,

Identities:578/620(93%), Gaps:3/620(0%), Strand: Plus/Plus

Query 19 TGATCGGGGA-AGTCGGAACTTCATTAAGAGTTTTAATTCGAACTGAACTTAGTCACCCT 77

||||| || | ||| |||||||| ||||||||||||||||||||||||||||||||||||

Sbjct 1 TGATCTGGAATAGTAGGAACTTCTTTAAGAGTTTTAATTCGAACTGAACTTAGTCACCCT 60

Query 78 GGAATATTTATCGGAAATGATCAAATTTATAATGTAATTCGTTACTAGCTCATGCATTTA 137

|| |||||||| |||||||| |||||||||||||||||| |||||| |||||||||||||

Sbjct 61 GGTATATTTATTGGAAATGACCAAATTTATAATGTAATT-GTTACT-GCTCATGCATTTA 118

Query 138 TTATAAttttttttATAGTAATACCTATTATAATTGGAGGATTTGGAAATTGGTTAGTCC 197

|||||||||||||||||||||||||||||||||||||||||||||||||||| ||||| |

Sbjct 119 TTATAATTTTTTTTATAGTAATACCTATTATAATTGGAGGATTTGGAAATTGATTAGTTC 178

Query 198 CATTAATATTAGGTGCTCCTGATATAGCTTTCCCTCGAATAAATAATATAAGTTTTTGAA 257

| ||||||||||| || |||||||||||||| ||||||||||||||||||||||||||||

Sbjct 179 CTTTAATATTAGGAGCCCCTGATATAGCTTTTCCTCGAATAAATAATATAAGTTTTTGAA 238

Query 258 TACTTCCTCCCTCTTTAACTCTTTTAATTTCTAGATCTATAGTAGAAAATGGAGCAGGAA 317

|||||||||| ||||||||||||||| |||||||||||||||||||||||||||||||||

Sbjct 239 TACTTCCTCCTTCTTTAACTCTTTTACTTTCTAGATCTATAGTAGAAAATGGAGCAGGAA 298

Query 318 CAGGTTGAACTGTATACCCTCCTCTTTCTTCTGGAACTGCTCATGCAGGAGCTTCTGTAG 377

| ||||||||||| || |||||||||||||||||||| |||||||| |||||||| || |

Sbjct 299 CTGGTTGAACTGTTTATCCTCCTCTTTCTTCTGGAACCGCTCATGCTGGAGCTTCAGTTG 358

Query 378 ATTTAGCTATTTTTTCTCTTCATTTAGCAGGAATTTCTTCTATTTTAGGAGCAGTAAATT 437

||||||||||||||||| | ||||||||||| ||||| ||||||||||||||||||||||

Sbjct 359 ATTTAGCTATTTTTTCTTTACATTTAGCAGGTATTTCATCTATTTTAGGAGCAGTAAATT 418

Query 438 TTATTACAACTGTAATTAATATACGATCAACTGGAATTACACTTGATCGTTTACCTTTAT 497

|||||||||||||||||||||| |||||| ||||||||||||||||||| ||||||||||

Sbjct 419 TTATTACAACTGTAATTAATATGCGATCAGCTGGAATTACACTTGATCGATTACCTTTAT 478

Query 498 TTGTCTGATCTGTAATTATTACAGCTATTTTATTACTTCTTTCATTACCAGTTTTAGCAG 557

|||| ||||| ||| | |||||||| ||||||||||||||||||||||| ||||||||||

Sbjct 479 TTGTTTGATCAGTAGTAATTACAGCAATTTTATTACTTCTTTCATTACCTGTTTTAGCAG 538

Query 558 GAGCTATTACTATATTATTAACTGATCGAAATTTTAATACATCATTTTTTGACCCAATTG 617

|||| || ||||| ||||||||||||||||||||||||||||||||||||||||||||||

Sbjct 539 GAGCAATCACTATGTTATTAACTGATCGAAATTTTAATACATCATTTTTTGACCCAATTG 598

Query 618 GAGGAGGTGACCCTATTCTT 637

|||||||||| |||||||||

Sbjct 599 GAGGAGGTGATCCTATTCTT 618

>Aedes polynesiensis voucher WA029 cytochrome c oxidase subunit I (COX1) gene, partial cds; mitochondrial

Sequence ID: MW664768.1 Length: 618

Range 1: 1 to 618

Score:909 bits(492), Expect:0.0,

Identities:578/620(93%), Gaps:3/620(0%), Strand: Plus/Plus

Query 19 TGATCGGGGA-AGTCGGAACTTCATTAAGAGTTTTAATTCGAACTGAACTTAGTCACCCT 77

||||| || | ||| |||||||| ||||||||||||||||||||||||||||||||||||

Sbjct 1 TGATCTGGAATAGTAGGAACTTCTTTAAGAGTTTTAATTCGAACTGAACTTAGTCACCCT 60

Query 78 GGAATATTTATCGGAAATGATCAAATTTATAATGTAATTCGTTACTAGCTCATGCATTTA 137

|| |||||||| |||||||| |||||||||||||||||| |||||| |||||||||||||

Sbjct 61 GGTATATTTATTGGAAATGACCAAATTTATAATGTAATT-GTTACT-GCTCATGCATTTA 118

Query 138 TTATAAttttttttATAGTAATACCTATTATAATTGGAGGATTTGGAAATTGGTTAGTCC 197

|||||||||||||||||||||||||||||||||||||||||||||||||||| ||||| |

Sbjct 119 TTATAATTTTTTTTATAGTAATACCTATTATAATTGGAGGATTTGGAAATTGATTAGTTC 178

Query 198 CATTAATATTAGGTGCTCCTGATATAGCTTTCCCTCGAATAAATAATATAAGTTTTTGAA 257

| ||||||||||| || |||||||||||||| ||||||||||||||||||||||||||||

Sbjct 179 CTTTAATATTAGGAGCCCCTGATATAGCTTTTCCTCGAATAAATAATATAAGTTTTTGAA 238

Query 258 TACTTCCTCCCTCTTTAACTCTTTTAATTTCTAGATCTATAGTAGAAAATGGAGCAGGAA 317

|||||||||| ||||||||||||||| |||||||||||||||||||||||||||||||||

Sbjct 239 TACTTCCTCCTTCTTTAACTCTTTTACTTTCTAGATCTATAGTAGAAAATGGAGCAGGAA 298

Query 318 CAGGTTGAACTGTATACCCTCCTCTTTCTTCTGGAACTGCTCATGCAGGAGCTTCTGTAG 377

| ||||||||||| || |||||||||||||||||||| |||||||| |||||||| || |

Sbjct 299 CTGGTTGAACTGTTTATCCTCCTCTTTCTTCTGGAACCGCTCATGCTGGAGCTTCAGTTG 358

Query 378 ATTTAGCTATTTTTTCTCTTCATTTAGCAGGAATTTCTTCTATTTTAGGAGCAGTAAATT 437

||||||||||||||||| | ||||||||||| ||||| ||||||||||||||||||||||

Sbjct 359 ATTTAGCTATTTTTTCTTTACATTTAGCAGGTATTTCATCTATTTTAGGAGCAGTAAATT 418

Query 438 TTATTACAACTGTAATTAATATACGATCAACTGGAATTACACTTGATCGTTTACCTTTAT 497

|||||||||||||||||||||| |||||| ||||||||||||||||||| ||||||||||

Sbjct 419 TTATTACAACTGTAATTAATATGCGATCAGCTGGAATTACACTTGATCGATTACCTTTAT 478

Query 498 TTGTCTGATCTGTAATTATTACAGCTATTTTATTACTTCTTTCATTACCAGTTTTAGCAG 557

|||| ||||| ||| | |||||||| ||||||||||||||||||||||| ||||||||||

Sbjct 479 TTGTTTGATCAGTAGTAATTACAGCAATTTTATTACTTCTTTCATTACCTGTTTTAGCAG 538

Query 558 GAGCTATTACTATATTATTAACTGATCGAAATTTTAATACATCATTTTTTGACCCAATTG 617

|||| || ||||| ||||||||||||||||||||||||||||||||||||||||||||||

Sbjct 539 GAGCAATCACTATGTTATTAACTGATCGAAATTTTAATACATCATTTTTTGACCCAATTG 598

Query 618 GAGGAGGTGACCCTATTCTT 637

|||||||||| |||||||||

Sbjct 599 GAGGAGGTGATCCTATTCTT 618

>Aedes polynesiensis voucher WA027 cytochrome c oxidase subunit I (COX1) gene, partial cds; mitochondrial

Sequence ID: MW664767.1 Length: 618

Range 1: 1 to 618

Score:909 bits(492), Expect:0.0,

Identities:578/620(93%), Gaps:3/620(0%), Strand: Plus/Plus

Query 19 TGATCGGGGA-AGTCGGAACTTCATTAAGAGTTTTAATTCGAACTGAACTTAGTCACCCT 77

||||| || | ||| |||||||| ||||||||||||||||||||||||||||||||||||

Sbjct 1 TGATCTGGAATAGTAGGAACTTCTTTAAGAGTTTTAATTCGAACTGAACTTAGTCACCCT 60

Query 78 GGAATATTTATCGGAAATGATCAAATTTATAATGTAATTCGTTACTAGCTCATGCATTTA 137

|| |||||||| |||||||| |||||||||||||||||| |||||| |||||||||||||

Sbjct 61 GGTATATTTATTGGAAATGACCAAATTTATAATGTAATT-GTTACT-GCTCATGCATTTA 118

Query 138 TTATAAttttttttATAGTAATACCTATTATAATTGGAGGATTTGGAAATTGGTTAGTCC 197

|||||||||||||||||||||||||||||||||||||||||||||||||||| ||||| |

Sbjct 119 TTATAATTTTTTTTATAGTAATACCTATTATAATTGGAGGATTTGGAAATTGATTAGTTC 178

Query 198 CATTAATATTAGGTGCTCCTGATATAGCTTTCCCTCGAATAAATAATATAAGTTTTTGAA 257

| ||||||||||| || |||||||||||||| ||||||||||||||||||||||||||||

Sbjct 179 CTTTAATATTAGGAGCCCCTGATATAGCTTTTCCTCGAATAAATAATATAAGTTTTTGAA 238

Query 258 TACTTCCTCCCTCTTTAACTCTTTTAATTTCTAGATCTATAGTAGAAAATGGAGCAGGAA 317

|||||||||| ||||||||||||||| |||||||||||||||||||||||||||||||||

Sbjct 239 TACTTCCTCCTTCTTTAACTCTTTTACTTTCTAGATCTATAGTAGAAAATGGAGCAGGAA 298

Query 318 CAGGTTGAACTGTATACCCTCCTCTTTCTTCTGGAACTGCTCATGCAGGAGCTTCTGTAG 377

| ||||||||||| || |||||||||||||||||||| |||||||| |||||||| || |

Sbjct 299 CTGGTTGAACTGTTTATCCTCCTCTTTCTTCTGGAACCGCTCATGCTGGAGCTTCAGTTG 358

Query 378 ATTTAGCTATTTTTTCTCTTCATTTAGCAGGAATTTCTTCTATTTTAGGAGCAGTAAATT 437

||||||||||||||||| | ||||||||||| ||||| ||||||||||||||||||||||

Sbjct 359 ATTTAGCTATTTTTTCTTTACATTTAGCAGGTATTTCATCTATTTTAGGAGCAGTAAATT 418

Query 438 TTATTACAACTGTAATTAATATACGATCAACTGGAATTACACTTGATCGTTTACCTTTAT 497

|||||||||||||||||||||| |||||| ||||||||||||||||||| ||||||||||

Sbjct 419 TTATTACAACTGTAATTAATATGCGATCAGCTGGAATTACACTTGATCGATTACCTTTAT 478

Query 498 TTGTCTGATCTGTAATTATTACAGCTATTTTATTACTTCTTTCATTACCAGTTTTAGCAG 557

|||| ||||| ||| | |||||||| ||||||||||||||||||||||| ||||||||||

Sbjct 479 TTGTTTGATCAGTAGTAATTACAGCAATTTTATTACTTCTTTCATTACCTGTTTTAGCAG 538

Query 558 GAGCTATTACTATATTATTAACTGATCGAAATTTTAATACATCATTTTTTGACCCAATTG 617

|||| || ||||| ||||||||||||||||||||||||||||||||||||||||||||||

Sbjct 539 GAGCAATCACTATGTTATTAACTGATCGAAATTTTAATACATCATTTTTTGACCCAATTG 598

Query 618 GAGGAGGTGACCCTATTCTT 637

|||||||||| |||||||||

Sbjct 599 GAGGAGGTGATCCTATTCTT 618

>Aedes polynesiensis voucher WA026 cytochrome c oxidase subunit I (COX1) gene, partial cds; mitochondrial

Sequence ID: MW664766.1 Length: 618

Range 1: 1 to 618

Score:909 bits(492), Expect:0.0,

Identities:578/620(93%), Gaps:3/620(0%), Strand: Plus/Plus

Query 19 TGATCGGGGA-AGTCGGAACTTCATTAAGAGTTTTAATTCGAACTGAACTTAGTCACCCT 77

||||| || | ||| |||||||| ||||||||||||||||||||||||||||||||||||

Sbjct 1 TGATCTGGAATAGTAGGAACTTCTTTAAGAGTTTTAATTCGAACTGAACTTAGTCACCCT 60

Query 78 GGAATATTTATCGGAAATGATCAAATTTATAATGTAATTCGTTACTAGCTCATGCATTTA 137

|| |||||||| |||||||| |||||||||||||||||| |||||| |||||||||||||

Sbjct 61 GGTATATTTATTGGAAATGACCAAATTTATAATGTAATT-GTTACT-GCTCATGCATTTA 118

Query 138 TTATAAttttttttATAGTAATACCTATTATAATTGGAGGATTTGGAAATTGGTTAGTCC 197

|||||||||||||||||||||||||||||||||||||||||||||||||||| ||||| |

Sbjct 119 TTATAATTTTTTTTATAGTAATACCTATTATAATTGGAGGATTTGGAAATTGATTAGTTC 178

Query 198 CATTAATATTAGGTGCTCCTGATATAGCTTTCCCTCGAATAAATAATATAAGTTTTTGAA 257

| ||||||||||| || |||||||||||||| ||||||||||||||||||||||||||||

Sbjct 179 CTTTAATATTAGGAGCCCCTGATATAGCTTTTCCTCGAATAAATAATATAAGTTTTTGAA 238

Query 258 TACTTCCTCCCTCTTTAACTCTTTTAATTTCTAGATCTATAGTAGAAAATGGAGCAGGAA 317

|||||||||| ||||||||||||||| |||||||||||||||||||||||||||||||||

Sbjct 239 TACTTCCTCCTTCTTTAACTCTTTTACTTTCTAGATCTATAGTAGAAAATGGAGCAGGAA 298

Query 318 CAGGTTGAACTGTATACCCTCCTCTTTCTTCTGGAACTGCTCATGCAGGAGCTTCTGTAG 377

| ||||||||||| || |||||||||||||||||||| |||||||| |||||||| || |

Sbjct 299 CTGGTTGAACTGTTTATCCTCCTCTTTCTTCTGGAACCGCTCATGCTGGAGCTTCAGTTG 358

Query 378 ATTTAGCTATTTTTTCTCTTCATTTAGCAGGAATTTCTTCTATTTTAGGAGCAGTAAATT 437

||||||||||||||||| | ||||||||||| ||||| ||||||||||||||||||||||

Sbjct 359 ATTTAGCTATTTTTTCTTTACATTTAGCAGGTATTTCATCTATTTTAGGAGCAGTAAATT 418

Query 438 TTATTACAACTGTAATTAATATACGATCAACTGGAATTACACTTGATCGTTTACCTTTAT 497

|||||||||||||||||||||| |||||| ||||||||||||||||||| ||||||||||

Sbjct 419 TTATTACAACTGTAATTAATATGCGATCAGCTGGAATTACACTTGATCGATTACCTTTAT 478

Query 498 TTGTCTGATCTGTAATTATTACAGCTATTTTATTACTTCTTTCATTACCAGTTTTAGCAG 557

|||| ||||| ||| | |||||||| ||||||||||||||||||||||| ||||||||||

Sbjct 479 TTGTTTGATCAGTAGTAATTACAGCAATTTTATTACTTCTTTCATTACCTGTTTTAGCAG 538

Query 558 GAGCTATTACTATATTATTAACTGATCGAAATTTTAATACATCATTTTTTGACCCAATTG 617

|||| || ||||| ||||||||||||||||||||||||||||||||||||||||||||||

Sbjct 539 GAGCAATCACTATGTTATTAACTGATCGAAATTTTAATACATCATTTTTTGACCCAATTG 598

Query 618 GAGGAGGTGACCCTATTCTT 637

|||||||||| |||||||||

Sbjct 599 GAGGAGGTGATCCTATTCTT 618

>Aedes polynesiensis voucher WA025 cytochrome c oxidase subunit I (COX1) gene, partial cds; mitochondrial

Sequence ID: MW664765.1 Length: 618

Range 1: 1 to 618

Score:909 bits(492), Expect:0.0,

Identities:578/620(93%), Gaps:3/620(0%), Strand: Plus/Plus

Query 19 TGATCGGGGA-AGTCGGAACTTCATTAAGAGTTTTAATTCGAACTGAACTTAGTCACCCT 77

||||| || | ||| |||||||| ||||||||||||||||||||||||||||||||||||

Sbjct 1 TGATCTGGAATAGTAGGAACTTCTTTAAGAGTTTTAATTCGAACTGAACTTAGTCACCCT 60

Query 78 GGAATATTTATCGGAAATGATCAAATTTATAATGTAATTCGTTACTAGCTCATGCATTTA 137

|| |||||||| |||||||| |||||||||||||||||| |||||| |||||||||||||

Sbjct 61 GGTATATTTATTGGAAATGACCAAATTTATAATGTAATT-GTTACT-GCTCATGCATTTA 118

Query 138 TTATAAttttttttATAGTAATACCTATTATAATTGGAGGATTTGGAAATTGGTTAGTCC 197

|||||||||||||||||||||||||||||||||||||||||||||||||||| ||||| |

Sbjct 119 TTATAATTTTTTTTATAGTAATACCTATTATAATTGGAGGATTTGGAAATTGATTAGTTC 178

Query 198 CATTAATATTAGGTGCTCCTGATATAGCTTTCCCTCGAATAAATAATATAAGTTTTTGAA 257

| ||||||||||| || |||||||||||||| ||||||||||||||||||||||||||||

Sbjct 179 CTTTAATATTAGGAGCCCCTGATATAGCTTTTCCTCGAATAAATAATATAAGTTTTTGAA 238

Query 258 TACTTCCTCCCTCTTTAACTCTTTTAATTTCTAGATCTATAGTAGAAAATGGAGCAGGAA 317

|||||||||| ||||||||||||||| |||||||||||||||||||||||||||||||||

Sbjct 239 TACTTCCTCCTTCTTTAACTCTTTTACTTTCTAGATCTATAGTAGAAAATGGAGCAGGAA 298

Query 318 CAGGTTGAACTGTATACCCTCCTCTTTCTTCTGGAACTGCTCATGCAGGAGCTTCTGTAG 377

| ||||||||||| || |||||||||||||||||||| |||||||| |||||||| || |

Sbjct 299 CTGGTTGAACTGTTTATCCTCCTCTTTCTTCTGGAACCGCTCATGCTGGAGCTTCAGTTG 358

Query 378 ATTTAGCTATTTTTTCTCTTCATTTAGCAGGAATTTCTTCTATTTTAGGAGCAGTAAATT 437

||||||||||||||||| | ||||||||||| ||||| ||||||||||||||||||||||

Sbjct 359 ATTTAGCTATTTTTTCTTTACATTTAGCAGGTATTTCATCTATTTTAGGAGCAGTAAATT 418

Query 438 TTATTACAACTGTAATTAATATACGATCAACTGGAATTACACTTGATCGTTTACCTTTAT 497

|||||||||||||||||||||| |||||| ||||||||||||||||||| ||||||||||

Sbjct 419 TTATTACAACTGTAATTAATATGCGATCAGCTGGAATTACACTTGATCGATTACCTTTAT 478

Query 498 TTGTCTGATCTGTAATTATTACAGCTATTTTATTACTTCTTTCATTACCAGTTTTAGCAG 557

|||| ||||| ||| | |||||||| ||||||||||||||||||||||| ||||||||||

Sbjct 479 TTGTTTGATCAGTAGTAATTACAGCAATTTTATTACTTCTTTCATTACCTGTTTTAGCAG 538

Query 558 GAGCTATTACTATATTATTAACTGATCGAAATTTTAATACATCATTTTTTGACCCAATTG 617

|||| || ||||| ||||||||||||||||||||||||||||||||||||||||||||||

Sbjct 539 GAGCAATCACTATGTTATTAACTGATCGAAATTTTAATACATCATTTTTTGACCCAATTG 598

Query 618 GAGGAGGTGACCCTATTCTT 637

|||||||||| |||||||||

Sbjct 599 GAGGAGGTGATCCTATTCTT 618

>Aedes polynesiensis voucher WA024 cytochrome c oxidase subunit I (COX1) gene, partial cds; mitochondrial

Sequence ID: MW664764.1 Length: 618

Range 1: 1 to 618

Score:909 bits(492), Expect:0.0,

Identities:578/620(93%), Gaps:3/620(0%), Strand: Plus/Plus

Query 19 TGATCGGGGA-AGTCGGAACTTCATTAAGAGTTTTAATTCGAACTGAACTTAGTCACCCT 77

||||| || | ||| |||||||| ||||||||||||||||||||||||||||||||||||

Sbjct 1 TGATCTGGAATAGTAGGAACTTCTTTAAGAGTTTTAATTCGAACTGAACTTAGTCACCCT 60

Query 78 GGAATATTTATCGGAAATGATCAAATTTATAATGTAATTCGTTACTAGCTCATGCATTTA 137

|| |||||||| |||||||| |||||||||||||||||| |||||| |||||||||||||

Sbjct 61 GGTATATTTATTGGAAATGACCAAATTTATAATGTAATT-GTTACT-GCTCATGCATTTA 118

Query 138 TTATAAttttttttATAGTAATACCTATTATAATTGGAGGATTTGGAAATTGGTTAGTCC 197

|||||||||||||||||||||||||||||||||||||||||||||||||||| ||||| |

Sbjct 119 TTATAATTTTTTTTATAGTAATACCTATTATAATTGGAGGATTTGGAAATTGATTAGTTC 178

Query 198 CATTAATATTAGGTGCTCCTGATATAGCTTTCCCTCGAATAAATAATATAAGTTTTTGAA 257

| ||||||||||| || |||||||||||||| ||||||||||||||||||||||||||||

Sbjct 179 CTTTAATATTAGGAGCCCCTGATATAGCTTTTCCTCGAATAAATAATATAAGTTTTTGAA 238

Query 258 TACTTCCTCCCTCTTTAACTCTTTTAATTTCTAGATCTATAGTAGAAAATGGAGCAGGAA 317

|||||||||| ||||||||||||||| |||||||||||||||||||||||||||||||||

Sbjct 239 TACTTCCTCCTTCTTTAACTCTTTTACTTTCTAGATCTATAGTAGAAAATGGAGCAGGAA 298

Query 318 CAGGTTGAACTGTATACCCTCCTCTTTCTTCTGGAACTGCTCATGCAGGAGCTTCTGTAG 377

| ||||||||||| || |||||||||||||||||||| |||||||| |||||||| || |

Sbjct 299 CTGGTTGAACTGTTTATCCTCCTCTTTCTTCTGGAACCGCTCATGCTGGAGCTTCAGTTG 358

Query 378 ATTTAGCTATTTTTTCTCTTCATTTAGCAGGAATTTCTTCTATTTTAGGAGCAGTAAATT 437

||||||||||||||||| | ||||||||||| ||||| ||||||||||||||||||||||

Sbjct 359 ATTTAGCTATTTTTTCTTTACATTTAGCAGGTATTTCATCTATTTTAGGAGCAGTAAATT 418

Query 438 TTATTACAACTGTAATTAATATACGATCAACTGGAATTACACTTGATCGTTTACCTTTAT 497

|||||||||||||||||||||| |||||| ||||||||||||||||||| ||||||||||

Sbjct 419 TTATTACAACTGTAATTAATATGCGATCAGCTGGAATTACACTTGATCGATTACCTTTAT 478

Query 498 TTGTCTGATCTGTAATTATTACAGCTATTTTATTACTTCTTTCATTACCAGTTTTAGCAG 557

|||| ||||| ||| | |||||||| ||||||||||||||||||||||| ||||||||||

Sbjct 479 TTGTTTGATCAGTAGTAATTACAGCAATTTTATTACTTCTTTCATTACCTGTTTTAGCAG 538

Query 558 GAGCTATTACTATATTATTAACTGATCGAAATTTTAATACATCATTTTTTGACCCAATTG 617

|||| || ||||| ||||||||||||||||||||||||||||||||||||||||||||||

Sbjct 539 GAGCAATCACTATGTTATTAACTGATCGAAATTTTAATACATCATTTTTTGACCCAATTG 598

Query 618 GAGGAGGTGACCCTATTCTT 637

|||||||||| |||||||||

Sbjct 599 GAGGAGGTGATCCTATTCTT 618

>Aedes pseudoscutellaris voucher FI272 cytochrome c oxidase subunit I (COX1) gene, partial cds; mitochondrial

Sequence ID: MW664812.1 Length: 618

Range 1: 1 to 618

Score:904 bits(489), Expect:0.0,

Identities:577/620(93%), Gaps:3/620(0%), Strand: Plus/Plus

Query 19 TGATCGGGGA-AGTCGGAACTTCATTAAGAGTTTTAATTCGAACTGAACTTAGTCACCCT 77

||||| || | ||| |||||||| ||||||||||||||||||||||||||||||||||||

Sbjct 1 TGATCTGGAATAGTAGGAACTTCTTTAAGAGTTTTAATTCGAACTGAACTTAGTCACCCT 60

Query 78 GGAATATTTATCGGAAATGATCAAATTTATAATGTAATTCGTTACTAGCTCATGCATTTA 137

|| |||||||| |||||||| |||||||||||||||||| |||||| |||||||||||||

Sbjct 61 GGTATATTTATTGGAAATGACCAAATTTATAATGTAATT-GTTACT-GCTCATGCATTTA 118

Query 138 TTATAAttttttttATAGTAATACCTATTATAATTGGAGGATTTGGAAATTGGTTAGTCC 197

|||||||||||||||||||||||||||||||||||||||||||||||||||| ||||| |

Sbjct 119 TTATAATTTTTTTTATAGTAATACCTATTATAATTGGAGGATTTGGAAATTGATTAGTTC 178

Query 198 CATTAATATTAGGTGCTCCTGATATAGCTTTCCCTCGAATAAATAATATAAGTTTTTGAA 257

| ||||||||||| || |||||||||||||| ||||||||||||||||||||||||||||

Sbjct 179 CTTTAATATTAGGAGCCCCTGATATAGCTTTTCCTCGAATAAATAATATAAGTTTTTGAA 238

Query 258 TACTTCCTCCCTCTTTAACTCTTTTAATTTCTAGATCTATAGTAGAAAATGGAGCAGGAA 317

|||||||||| ||||||||||||||| |||||||||||||||||||||||||||||||||

Sbjct 239 TACTTCCTCCTTCTTTAACTCTTTTACTTTCTAGATCTATAGTAGAAAATGGAGCAGGAA 298

Query 318 CAGGTTGAACTGTATACCCTCCTCTTTCTTCTGGAACTGCTCATGCAGGAGCTTCTGTAG 377

| ||||||||||| || |||||||||||||||||||| |||||||| |||||||| || |

Sbjct 299 CTGGTTGAACTGTTTATCCTCCTCTTTCTTCTGGAACCGCTCATGCTGGAGCTTCAGTTG 358

Query 378 ATTTAGCTATTTTTTCTCTTCATTTAGCAGGAATTTCTTCTATTTTAGGAGCAGTAAATT 437

||||||||||||||||| | ||||||||||| ||||| ||||||||||||||||||||||

Sbjct 359 ATTTAGCTATTTTTTCTTTACATTTAGCAGGTATTTCATCTATTTTAGGAGCAGTAAATT 418

Query 438 TTATTACAACTGTAATTAATATACGATCAACTGGAATTACACTTGATCGTTTACCTTTAT 497

||||||||||||||||||||||||||||| |||||||||| |||||||| ||||||||||

Sbjct 419 TTATTACAACTGTAATTAATATACGATCAGCTGGAATTACGCTTGATCGATTACCTTTAT 478

Query 498 TTGTCTGATCTGTAATTATTACAGCTATTTTATTACTTCTTTCATTACCAGTTTTAGCAG 557

|||| ||||| ||| | |||||||| ||||||||||||||||||||||| ||||||||||

Sbjct 479 TTGTTTGATCAGTAGTAATTACAGCAATTTTATTACTTCTTTCATTACCTGTTTTAGCAG 538

Query 558 GAGCTATTACTATATTATTAACTGATCGAAATTTTAATACATCATTTTTTGACCCAATTG 617

|||| || ||||| |||||||||||||||||||||||||||||||||||||| |||||||

Sbjct 539 GAGCAATCACTATGTTATTAACTGATCGAAATTTTAATACATCATTTTTTGATCCAATTG 598

Query 618 GAGGAGGTGACCCTATTCTT 637

|||||||||| |||||||||

Sbjct 599 GAGGAGGTGATCCTATTCTT 618

>Aedes pseudoscutellaris voucher FI271 cytochrome c oxidase subunit I (COX1) gene, partial cds; mitochondrial

Sequence ID: MW664811.1 Length: 618

Range 1: 1 to 618

Score:904 bits(489), Expect:0.0,

Identities:577/620(93%), Gaps:3/620(0%), Strand: Plus/Plus

Query 19 TGATCGGGGA-AGTCGGAACTTCATTAAGAGTTTTAATTCGAACTGAACTTAGTCACCCT 77

||||| || | ||| |||||||| ||||||||||||||||||||||||||||||||||||

Sbjct 1 TGATCTGGAATAGTAGGAACTTCTTTAAGAGTTTTAATTCGAACTGAACTTAGTCACCCT 60

Query 78 GGAATATTTATCGGAAATGATCAAATTTATAATGTAATTCGTTACTAGCTCATGCATTTA 137

|| |||||||| |||||||| |||||||||||||||||| |||||| |||||||||||||

Sbjct 61 GGTATATTTATTGGAAATGACCAAATTTATAATGTAATT-GTTACT-GCTCATGCATTTA 118

Query 138 TTATAAttttttttATAGTAATACCTATTATAATTGGAGGATTTGGAAATTGGTTAGTCC 197

|||||||||||||||||||||||||||||||||||||||||||||||||||| ||||| |

Sbjct 119 TTATAATTTTTTTTATAGTAATACCTATTATAATTGGAGGATTTGGAAATTGATTAGTTC 178

Query 198 CATTAATATTAGGTGCTCCTGATATAGCTTTCCCTCGAATAAATAATATAAGTTTTTGAA 257

| ||||||||||| || |||||||||||||| ||||||||||||||||||||||||||||

Sbjct 179 CTTTAATATTAGGAGCCCCTGATATAGCTTTTCCTCGAATAAATAATATAAGTTTTTGAA 238

Query 258 TACTTCCTCCCTCTTTAACTCTTTTAATTTCTAGATCTATAGTAGAAAATGGAGCAGGAA 317

|||||||||| ||||||||||||||| |||||||||||||||||||||||||||||||||

Sbjct 239 TACTTCCTCCTTCTTTAACTCTTTTACTTTCTAGATCTATAGTAGAAAATGGAGCAGGAA 298

Query 318 CAGGTTGAACTGTATACCCTCCTCTTTCTTCTGGAACTGCTCATGCAGGAGCTTCTGTAG 377

| ||||||||||| || |||||||||||||||||||| |||||||| |||||||| || |

Sbjct 299 CTGGTTGAACTGTTTATCCTCCTCTTTCTTCTGGAACCGCTCATGCTGGAGCTTCAGTTG 358

Query 378 ATTTAGCTATTTTTTCTCTTCATTTAGCAGGAATTTCTTCTATTTTAGGAGCAGTAAATT 437

||||||||||||||||| | ||||||||||| ||||| ||||||||||||||||||||||

Sbjct 359 ATTTAGCTATTTTTTCTTTACATTTAGCAGGTATTTCATCTATTTTAGGAGCAGTAAATT 418

Query 438 TTATTACAACTGTAATTAATATACGATCAACTGGAATTACACTTGATCGTTTACCTTTAT 497

||||||||||||||||||||||||||||| |||||||||| |||||||| ||||||||||

Sbjct 419 TTATTACAACTGTAATTAATATACGATCAGCTGGAATTACGCTTGATCGATTACCTTTAT 478

Query 498 TTGTCTGATCTGTAATTATTACAGCTATTTTATTACTTCTTTCATTACCAGTTTTAGCAG 557

|||| ||||| ||| | |||||||| ||||||||||||||||||||||| ||||||||||

Sbjct 479 TTGTTTGATCAGTAGTAATTACAGCAATTTTATTACTTCTTTCATTACCTGTTTTAGCAG 538

Query 558 GAGCTATTACTATATTATTAACTGATCGAAATTTTAATACATCATTTTTTGACCCAATTG 617

|||| || ||||| |||||||||||||||||||||||||||||||||||||| |||||||

Sbjct 539 GAGCAATCACTATGTTATTAACTGATCGAAATTTTAATACATCATTTTTTGATCCAATTG 598

Query 618 GAGGAGGTGACCCTATTCTT 637

|||||||||| |||||||||

Sbjct 599 GAGGAGGTGATCCTATTCTT 618

>Aedes pseudoscutellaris voucher FI205 cytochrome c oxidase subunit I (COX1) gene, partial cds; mitochondrial

Sequence ID: MW664810.1 Length: 618

Range 1: 1 to 618

Score:904 bits(489), Expect:0.0,

Identities:577/620(93%), Gaps:3/620(0%), Strand: Plus/Plus

Query 19 TGATCGGGGA-AGTCGGAACTTCATTAAGAGTTTTAATTCGAACTGAACTTAGTCACCCT 77

||||| || | ||| |||||||| ||||||||||||||||||||||||||||||||||||

Sbjct 1 TGATCTGGAATAGTAGGAACTTCTTTAAGAGTTTTAATTCGAACTGAACTTAGTCACCCT 60

Query 78 GGAATATTTATCGGAAATGATCAAATTTATAATGTAATTCGTTACTAGCTCATGCATTTA 137

|| |||||||| |||||||| |||||||||||||||||| |||||| |||||||||||||

Sbjct 61 GGTATATTTATTGGAAATGACCAAATTTATAATGTAATT-GTTACT-GCTCATGCATTTA 118

Query 138 TTATAAttttttttATAGTAATACCTATTATAATTGGAGGATTTGGAAATTGGTTAGTCC 197

|||||||||||||||||||||||||||||||||||||||||||||||||||| ||||| |

Sbjct 119 TTATAATTTTTTTTATAGTAATACCTATTATAATTGGAGGATTTGGAAATTGATTAGTTC 178

Query 198 CATTAATATTAGGTGCTCCTGATATAGCTTTCCCTCGAATAAATAATATAAGTTTTTGAA 257

| ||||||||||| || |||||||||||||| ||||||||||||||||||||||||||||

Sbjct 179 CTTTAATATTAGGAGCCCCTGATATAGCTTTTCCTCGAATAAATAATATAAGTTTTTGAA 238

Query 258 TACTTCCTCCCTCTTTAACTCTTTTAATTTCTAGATCTATAGTAGAAAATGGAGCAGGAA 317

|||||||||| ||||||||||||||| |||||||||||||||||||||||||||||||||

Sbjct 239 TACTTCCTCCTTCTTTAACTCTTTTACTTTCTAGATCTATAGTAGAAAATGGAGCAGGAA 298

Query 318 CAGGTTGAACTGTATACCCTCCTCTTTCTTCTGGAACTGCTCATGCAGGAGCTTCTGTAG 377

| ||||||||||| || |||||||||||||||||||| |||||||| |||||||| || |

Sbjct 299 CTGGTTGAACTGTTTATCCTCCTCTTTCTTCTGGAACCGCTCATGCTGGAGCTTCAGTTG 358

Query 378 ATTTAGCTATTTTTTCTCTTCATTTAGCAGGAATTTCTTCTATTTTAGGAGCAGTAAATT 437

||||||||||||||||| | ||||||||||| ||||| ||||||||||||||||||||||

Sbjct 359 ATTTAGCTATTTTTTCTTTACATTTAGCAGGTATTTCATCTATTTTAGGAGCAGTAAATT 418

Query 438 TTATTACAACTGTAATTAATATACGATCAACTGGAATTACACTTGATCGTTTACCTTTAT 497

||||||||||||||||||||||||||||| |||||||||| |||||||| ||||||||||

Sbjct 419 TTATTACAACTGTAATTAATATACGATCAGCTGGAATTACGCTTGATCGATTACCTTTAT 478

Query 498 TTGTCTGATCTGTAATTATTACAGCTATTTTATTACTTCTTTCATTACCAGTTTTAGCAG 557

|||| ||||| ||| | |||||||| ||||||||||||||||||||||| ||||||||||

Sbjct 479 TTGTTTGATCAGTAGTAATTACAGCAATTTTATTACTTCTTTCATTACCTGTTTTAGCAG 538

Query 558 GAGCTATTACTATATTATTAACTGATCGAAATTTTAATACATCATTTTTTGACCCAATTG 617

|||| || ||||| |||||||||||||||||||||||||||||||||||||| |||||||

Sbjct 539 GAGCAATCACTATGTTATTAACTGATCGAAATTTTAATACATCATTTTTTGATCCAATTG 598

Query 618 GAGGAGGTGACCCTATTCTT 637

|||||||||| |||||||||

Sbjct 599 GAGGAGGTGATCCTATTCTT 618

>Aedes pseudoscutellaris voucher FI314 cytochrome c oxidase subunit I (COX1) gene, partial cds; mitochondrial

Sequence ID: MW664809.1 Length: 618

Range 1: 1 to 618

Score:904 bits(489), Expect:0.0,

Identities:577/620(93%), Gaps:3/620(0%), Strand: Plus/Plus

Query 19 TGATCGGGGA-AGTCGGAACTTCATTAAGAGTTTTAATTCGAACTGAACTTAGTCACCCT 77

||||| || | ||| |||||||| ||||||||||||||||||||||||||||||||||||

Sbjct 1 TGATCTGGAATAGTAGGAACTTCTTTAAGAGTTTTAATTCGAACTGAACTTAGTCACCCT 60

Query 78 GGAATATTTATCGGAAATGATCAAATTTATAATGTAATTCGTTACTAGCTCATGCATTTA 137

|| |||||||| |||||||| |||||||||||||||||| |||||| |||||||||||||

Sbjct 61 GGTATATTTATTGGAAATGACCAAATTTATAATGTAATT-GTTACT-GCTCATGCATTTA 118

Query 138 TTATAAttttttttATAGTAATACCTATTATAATTGGAGGATTTGGAAATTGGTTAGTCC 197

|||||||||||||||||||||||||||||||||||||||||||||||||||| ||||| |

Sbjct 119 TTATAATTTTTTTTATAGTAATACCTATTATAATTGGAGGATTTGGAAATTGATTAGTTC 178

Query 198 CATTAATATTAGGTGCTCCTGATATAGCTTTCCCTCGAATAAATAATATAAGTTTTTGAA 257

| ||||||||||| || |||||||||||||| ||||||||||||||||||||||||||||

Sbjct 179 CTTTAATATTAGGAGCCCCTGATATAGCTTTTCCTCGAATAAATAATATAAGTTTTTGAA 238

Query 258 TACTTCCTCCCTCTTTAACTCTTTTAATTTCTAGATCTATAGTAGAAAATGGAGCAGGAA 317

|||||||||| ||||||||||||||| |||||||||||||||||||||||||||||||||

Sbjct 239 TACTTCCTCCTTCTTTAACTCTTTTACTTTCTAGATCTATAGTAGAAAATGGAGCAGGAA 298

Query 318 CAGGTTGAACTGTATACCCTCCTCTTTCTTCTGGAACTGCTCATGCAGGAGCTTCTGTAG 377

| ||||||||||| || |||||||||||||||||||| |||||||| |||||||| || |

Sbjct 299 CTGGTTGAACTGTTTATCCTCCTCTTTCTTCTGGAACCGCTCATGCTGGAGCTTCAGTTG 358

Query 378 ATTTAGCTATTTTTTCTCTTCATTTAGCAGGAATTTCTTCTATTTTAGGAGCAGTAAATT 437

||||||||||||||||| | ||||||||||| ||||| ||||||||||||||||||||||

Sbjct 359 ATTTAGCTATTTTTTCTTTACATTTAGCAGGTATTTCATCTATTTTAGGAGCAGTAAATT 418

Query 438 TTATTACAACTGTAATTAATATACGATCAACTGGAATTACACTTGATCGTTTACCTTTAT 497

||||||||||||||||||||||||||||| |||||||||| |||||||| ||||||||||

Sbjct 419 TTATTACAACTGTAATTAATATACGATCAGCTGGAATTACGCTTGATCGATTACCTTTAT 478

Query 498 TTGTCTGATCTGTAATTATTACAGCTATTTTATTACTTCTTTCATTACCAGTTTTAGCAG 557

|||| ||||| ||| | |||||||| ||||||||||||||||||||||| ||||||||||

Sbjct 479 TTGTTTGATCAGTAGTAATTACAGCAATTTTATTACTTCTTTCATTACCTGTTTTAGCAG 538

Query 558 GAGCTATTACTATATTATTAACTGATCGAAATTTTAATACATCATTTTTTGACCCAATTG 617

|||| || ||||| |||||||||||||||||||||||||||||||||||||| |||||||

Sbjct 539 GAGCAATCACTATGTTATTAACTGATCGAAATTTTAATACATCATTTTTTGATCCAATTG 598

Query 618 GAGGAGGTGACCCTATTCTT 637

|||||||||| |||||||||

Sbjct 599 GAGGAGGTGATCCTATTCTT 618

>Aedes pseudoscutellaris voucher FI163 cytochrome c oxidase subunit I (COX1) gene, partial cds; mitochondrial

Sequence ID: MW664808.1 Length: 618

Range 1: 1 to 618

Score:904 bits(489), Expect:0.0,

Identities:577/620(93%), Gaps:3/620(0%), Strand: Plus/Plus

Query 19 TGATCGGGGA-AGTCGGAACTTCATTAAGAGTTTTAATTCGAACTGAACTTAGTCACCCT 77

||||| || | ||| |||||||| ||||||||||||||||||||||||||||||||||||

Sbjct 1 TGATCTGGAATAGTAGGAACTTCTTTAAGAGTTTTAATTCGAACTGAACTTAGTCACCCT 60

Query 78 GGAATATTTATCGGAAATGATCAAATTTATAATGTAATTCGTTACTAGCTCATGCATTTA 137

|| |||||||| |||||||| |||||||||||||||||| |||||| |||||||||||||

Sbjct 61 GGTATATTTATTGGAAATGACCAAATTTATAATGTAATT-GTTACT-GCTCATGCATTTA 118

Query 138 TTATAAttttttttATAGTAATACCTATTATAATTGGAGGATTTGGAAATTGGTTAGTCC 197

|||||||||||||||||||||||||||||||||||||||||||||||||||| ||||| |

Sbjct 119 TTATAATTTTTTTTATAGTAATACCTATTATAATTGGAGGATTTGGAAATTGATTAGTTC 178

Query 198 CATTAATATTAGGTGCTCCTGATATAGCTTTCCCTCGAATAAATAATATAAGTTTTTGAA 257

| ||||||||||| || |||||||||||||| ||||||||||||||||||||||||||||

Sbjct 179 CTTTAATATTAGGAGCCCCTGATATAGCTTTTCCTCGAATAAATAATATAAGTTTTTGAA 238

Query 258 TACTTCCTCCCTCTTTAACTCTTTTAATTTCTAGATCTATAGTAGAAAATGGAGCAGGAA 317

|||||||||| ||||||||||||||| |||||||||||||||||||||||||||||||||

Sbjct 239 TACTTCCTCCTTCTTTAACTCTTTTACTTTCTAGATCTATAGTAGAAAATGGAGCAGGAA 298

Query 318 CAGGTTGAACTGTATACCCTCCTCTTTCTTCTGGAACTGCTCATGCAGGAGCTTCTGTAG 377

| ||||||||||| || |||||||||||||||||||| |||||||| |||||||| || |

Sbjct 299 CTGGTTGAACTGTTTATCCTCCTCTTTCTTCTGGAACCGCTCATGCTGGAGCTTCAGTTG 358

Query 378 ATTTAGCTATTTTTTCTCTTCATTTAGCAGGAATTTCTTCTATTTTAGGAGCAGTAAATT 437

||||||||||||||||| | ||||||||||| ||||| ||||||||||||||||||||||

Sbjct 359 ATTTAGCTATTTTTTCTTTACATTTAGCAGGTATTTCATCTATTTTAGGAGCAGTAAATT 418

Query 438 TTATTACAACTGTAATTAATATACGATCAACTGGAATTACACTTGATCGTTTACCTTTAT 497

||||||||||||||||||||||||||||| |||||||||| |||||||| ||||||||||

Sbjct 419 TTATTACAACTGTAATTAATATACGATCAGCTGGAATTACGCTTGATCGATTACCTTTAT 478

Query 498 TTGTCTGATCTGTAATTATTACAGCTATTTTATTACTTCTTTCATTACCAGTTTTAGCAG 557

|||| ||||| ||| | |||||||| ||||||||||||||||||||||| ||||||||||

Sbjct 479 TTGTTTGATCAGTAGTAATTACAGCAATTTTATTACTTCTTTCATTACCTGTTTTAGCAG 538

Query 558 GAGCTATTACTATATTATTAACTGATCGAAATTTTAATACATCATTTTTTGACCCAATTG 617

|||| || ||||| |||||||||||||||||||||||||||||||||||||| |||||||

Sbjct 539 GAGCAATCACTATGTTATTAACTGATCGAAATTTTAATACATCATTTTTTGATCCAATTG 598

Query 618 GAGGAGGTGACCCTATTCTT 637

|||||||||| |||||||||

Sbjct 599 GAGGAGGTGATCCTATTCTT 618

>Aedes pseudoscutellaris voucher FI013 cytochrome c oxidase subunit I (COX1) gene, partial cds; mitochondrial

Sequence ID: MW664807.1 Length: 618

Range 1: 1 to 618

Score:904 bits(489), Expect:0.0,

Identities:577/620(93%), Gaps:3/620(0%), Strand: Plus/Plus

Query 19 TGATCGGGGA-AGTCGGAACTTCATTAAGAGTTTTAATTCGAACTGAACTTAGTCACCCT 77

||||| || | ||| |||||||| ||||||||||||||||||||||||||||||||||||

Sbjct 1 TGATCTGGAATAGTAGGAACTTCTTTAAGAGTTTTAATTCGAACTGAACTTAGTCACCCT 60

Query 78 GGAATATTTATCGGAAATGATCAAATTTATAATGTAATTCGTTACTAGCTCATGCATTTA 137

|| |||||||| |||||||| |||||||||||||||||| |||||| |||||||||||||

Sbjct 61 GGTATATTTATTGGAAATGACCAAATTTATAATGTAATT-GTTACT-GCTCATGCATTTA 118

Query 138 TTATAAttttttttATAGTAATACCTATTATAATTGGAGGATTTGGAAATTGGTTAGTCC 197

|||||||||||||||||||||||||||||||||||||||||||||||||||| ||||| |

Sbjct 119 TTATAATTTTTTTTATAGTAATACCTATTATAATTGGAGGATTTGGAAATTGATTAGTTC 178

Query 198 CATTAATATTAGGTGCTCCTGATATAGCTTTCCCTCGAATAAATAATATAAGTTTTTGAA 257

| ||||||||||| || |||||||||||||| ||||||||||||||||||||||||||||

Sbjct 179 CTTTAATATTAGGAGCCCCTGATATAGCTTTTCCTCGAATAAATAATATAAGTTTTTGAA 238

Query 258 TACTTCCTCCCTCTTTAACTCTTTTAATTTCTAGATCTATAGTAGAAAATGGAGCAGGAA 317

|||||||||| ||||||||||||||| |||||||||||||||||||||||||||||||||

Sbjct 239 TACTTCCTCCTTCTTTAACTCTTTTACTTTCTAGATCTATAGTAGAAAATGGAGCAGGAA 298

Query 318 CAGGTTGAACTGTATACCCTCCTCTTTCTTCTGGAACTGCTCATGCAGGAGCTTCTGTAG 377

| ||||||||||| || |||||||||||||||||||| |||||||| |||||||| || |

Sbjct 299 CTGGTTGAACTGTTTATCCTCCTCTTTCTTCTGGAACCGCTCATGCTGGAGCTTCAGTTG 358

Query 378 ATTTAGCTATTTTTTCTCTTCATTTAGCAGGAATTTCTTCTATTTTAGGAGCAGTAAATT 437

||||||||||||||||| | ||||||||||| ||||| ||||||||||||||||||||||

Sbjct 359 ATTTAGCTATTTTTTCTTTACATTTAGCAGGTATTTCATCTATTTTAGGAGCAGTAAATT 418

Query 438 TTATTACAACTGTAATTAATATACGATCAACTGGAATTACACTTGATCGTTTACCTTTAT 497

||||||||||||||||||||||||||||| |||||||||| |||||||| ||||||||||

Sbjct 419 TTATTACAACTGTAATTAATATACGATCAGCTGGAATTACGCTTGATCGATTACCTTTAT 478

Query 498 TTGTCTGATCTGTAATTATTACAGCTATTTTATTACTTCTTTCATTACCAGTTTTAGCAG 557

|||| ||||| ||| | |||||||| ||||||||||||||||||||||| ||||||||||

Sbjct 479 TTGTTTGATCAGTAGTAATTACAGCAATTTTATTACTTCTTTCATTACCTGTTTTAGCAG 538

Query 558 GAGCTATTACTATATTATTAACTGATCGAAATTTTAATACATCATTTTTTGACCCAATTG 617

|||| || ||||| |||||||||||||||||||||||||||||||||||||| |||||||

Sbjct 539 GAGCAATCACTATGTTATTAACTGATCGAAATTTTAATACATCATTTTTTGATCCAATTG 598

Query 618 GAGGAGGTGACCCTATTCTT 637

|||||||||| |||||||||

Sbjct 599 GAGGAGGTGATCCTATTCTT 618

>Aedes pseudoscutellaris voucher FI221 cytochrome c oxidase subunit I (COX1) gene, partial cds; mitochondrial

Sequence ID: MW664806.1 Length: 618

Range 1: 1 to 618

Score:904 bits(489), Expect:0.0,

Identities:577/620(93%), Gaps:3/620(0%), Strand: Plus/Plus

Query 19 TGATCGGGGA-AGTCGGAACTTCATTAAGAGTTTTAATTCGAACTGAACTTAGTCACCCT 77

||||| || | ||| |||||||| ||||||||||||||||||||||||||||||||||||

Sbjct 1 TGATCTGGAATAGTAGGAACTTCTTTAAGAGTTTTAATTCGAACTGAACTTAGTCACCCT 60

Query 78 GGAATATTTATCGGAAATGATCAAATTTATAATGTAATTCGTTACTAGCTCATGCATTTA 137

|| |||||||| |||||||| |||||||||||||||||| |||||| |||||||||||||

Sbjct 61 GGTATATTTATTGGAAATGACCAAATTTATAATGTAATT-GTTACT-GCTCATGCATTTA 118

Query 138 TTATAAttttttttATAGTAATACCTATTATAATTGGAGGATTTGGAAATTGGTTAGTCC 197

|||||||||||||||||||||||||||||||||||||||||||||||||||| ||||| |

Sbjct 119 TTATAATTTTTTTTATAGTAATACCTATTATAATTGGAGGATTTGGAAATTGATTAGTTC 178

Query 198 CATTAATATTAGGTGCTCCTGATATAGCTTTCCCTCGAATAAATAATATAAGTTTTTGAA 257

| ||||||||||| || |||||||||||||| ||||||||||||||||||||||||||||

Sbjct 179 CTTTAATATTAGGAGCCCCTGATATAGCTTTTCCTCGAATAAATAATATAAGTTTTTGAA 238

Query 258 TACTTCCTCCCTCTTTAACTCTTTTAATTTCTAGATCTATAGTAGAAAATGGAGCAGGAA 317

|||||||||| ||||||||||||||| |||||||||||||||||||||||||||||||||

Sbjct 239 TACTTCCTCCTTCTTTAACTCTTTTACTTTCTAGATCTATAGTAGAAAATGGAGCAGGAA 298

Query 318 CAGGTTGAACTGTATACCCTCCTCTTTCTTCTGGAACTGCTCATGCAGGAGCTTCTGTAG 377

| ||||||||||| || |||||||||||||||||||| |||||||| |||||||| || |

Sbjct 299 CTGGTTGAACTGTTTATCCTCCTCTTTCTTCTGGAACCGCTCATGCTGGAGCTTCAGTTG 358

Query 378 ATTTAGCTATTTTTTCTCTTCATTTAGCAGGAATTTCTTCTATTTTAGGAGCAGTAAATT 437

||||||||||||||||| | ||||||||||| ||||| ||||||||||||||||||||||

Sbjct 359 ATTTAGCTATTTTTTCTTTACATTTAGCAGGTATTTCATCTATTTTAGGAGCAGTAAATT 418

Query 438 TTATTACAACTGTAATTAATATACGATCAACTGGAATTACACTTGATCGTTTACCTTTAT 497

||||||||||||||||||||||||||||| |||||||||| |||||||| ||||||||||

Sbjct 419 TTATTACAACTGTAATTAATATACGATCAGCTGGAATTACGCTTGATCGATTACCTTTAT 478

Query 498 TTGTCTGATCTGTAATTATTACAGCTATTTTATTACTTCTTTCATTACCAGTTTTAGCAG 557

|||| ||||| ||| | |||||||| ||||||||||||||||||||||| ||||||||||

Sbjct 479 TTGTTTGATCAGTAGTAATTACAGCAATTTTATTACTTCTTTCATTACCTGTTTTAGCAG 538

Query 558 GAGCTATTACTATATTATTAACTGATCGAAATTTTAATACATCATTTTTTGACCCAATTG 617

|||| || ||||| |||||||||||||||||||||||||||||||||||||| |||||||

Sbjct 539 GAGCAATCACTATGTTATTAACTGATCGAAATTTTAATACATCATTTTTTGATCCAATTG 598

Query 618 GAGGAGGTGACCCTATTCTT 637

|||||||||| |||||||||

Sbjct 599 GAGGAGGTGATCCTATTCTT 618

>Aedes pseudoscutellaris voucher FI220 cytochrome c oxidase subunit I (COX1) gene, partial cds; mitochondrial

Sequence ID: MW664805.1 Length: 618

Range 1: 1 to 618

Score:904 bits(489), Expect:0.0,

Identities:577/620(93%), Gaps:3/620(0%), Strand: Plus/Plus

Query 19 TGATCGGGGA-AGTCGGAACTTCATTAAGAGTTTTAATTCGAACTGAACTTAGTCACCCT 77

||||| || | ||| |||||||| ||||||||||||||||||||||||||||||||||||

Sbjct 1 TGATCTGGAATAGTAGGAACTTCTTTAAGAGTTTTAATTCGAACTGAACTTAGTCACCCT 60

Query 78 GGAATATTTATCGGAAATGATCAAATTTATAATGTAATTCGTTACTAGCTCATGCATTTA 137

|| |||||||| |||||||| |||||||||||||||||| |||||| |||||||||||||

Sbjct 61 GGTATATTTATTGGAAATGACCAAATTTATAATGTAATT-GTTACT-GCTCATGCATTTA 118

Query 138 TTATAAttttttttATAGTAATACCTATTATAATTGGAGGATTTGGAAATTGGTTAGTCC 197

|||||||||||||||||||||||||||||||||||||||||||||||||||| ||||| |

Sbjct 119 TTATAATTTTTTTTATAGTAATACCTATTATAATTGGAGGATTTGGAAATTGATTAGTTC 178

Query 198 CATTAATATTAGGTGCTCCTGATATAGCTTTCCCTCGAATAAATAATATAAGTTTTTGAA 257

| ||||||||||| || |||||||||||||| ||||||||||||||||||||||||||||

Sbjct 179 CTTTAATATTAGGAGCCCCTGATATAGCTTTTCCTCGAATAAATAATATAAGTTTTTGAA 238

Query 258 TACTTCCTCCCTCTTTAACTCTTTTAATTTCTAGATCTATAGTAGAAAATGGAGCAGGAA 317

|||||||||| ||||||||||||||| |||||||||||||||||||||||||||||||||

Sbjct 239 TACTTCCTCCTTCTTTAACTCTTTTACTTTCTAGATCTATAGTAGAAAATGGAGCAGGAA 298

Query 318 CAGGTTGAACTGTATACCCTCCTCTTTCTTCTGGAACTGCTCATGCAGGAGCTTCTGTAG 377

| ||||||||||| || |||||||||||||||||||| |||||||| |||||||| || |

Sbjct 299 CTGGTTGAACTGTTTATCCTCCTCTTTCTTCTGGAACCGCTCATGCTGGAGCTTCAGTTG 358

Query 378 ATTTAGCTATTTTTTCTCTTCATTTAGCAGGAATTTCTTCTATTTTAGGAGCAGTAAATT 437

||||||||||||||||| | ||||||||||| ||||| ||||||||||||||||||||||

Sbjct 359 ATTTAGCTATTTTTTCTTTACATTTAGCAGGTATTTCATCTATTTTAGGAGCAGTAAATT 418

Query 438 TTATTACAACTGTAATTAATATACGATCAACTGGAATTACACTTGATCGTTTACCTTTAT 497

||||||||||||||||||||||||||||| |||||||||| |||||||| ||||||||||

Sbjct 419 TTATTACAACTGTAATTAATATACGATCAGCTGGAATTACGCTTGATCGATTACCTTTAT 478

Query 498 TTGTCTGATCTGTAATTATTACAGCTATTTTATTACTTCTTTCATTACCAGTTTTAGCAG 557

|||| ||||| ||| | |||||||| ||||||||||||||||||||||| ||||||||||

Sbjct 479 TTGTTTGATCAGTAGTAATTACAGCAATTTTATTACTTCTTTCATTACCTGTTTTAGCAG 538

Query 558 GAGCTATTACTATATTATTAACTGATCGAAATTTTAATACATCATTTTTTGACCCAATTG 617

|||| || ||||| |||||||||||||||||||||||||||||||||||||| |||||||

Sbjct 539 GAGCAATCACTATGTTATTAACTGATCGAAATTTTAATACATCATTTTTTGATCCAATTG 598

Query 618 GAGGAGGTGACCCTATTCTT 637

|||||||||| |||||||||

Sbjct 599 GAGGAGGTGATCCTATTCTT 618

>Aedes pseudoscutellaris voucher FI012 cytochrome c oxidase subunit I (COX1) gene, partial cds; mitochondrial

Sequence ID: MW664804.1 Length: 618

Range 1: 1 to 618

Score:904 bits(489), Expect:0.0,

Identities:577/620(93%), Gaps:3/620(0%), Strand: Plus/Plus

Query 19 TGATCGGGGA-AGTCGGAACTTCATTAAGAGTTTTAATTCGAACTGAACTTAGTCACCCT 77

||||| || | ||| |||||||| ||||||||||||||||||||||||||||||||||||

Sbjct 1 TGATCTGGAATAGTAGGAACTTCTTTAAGAGTTTTAATTCGAACTGAACTTAGTCACCCT 60

Query 78 GGAATATTTATCGGAAATGATCAAATTTATAATGTAATTCGTTACTAGCTCATGCATTTA 137

|| |||||||| |||||||| |||||||||||||||||| |||||| |||||||||||||

Sbjct 61 GGTATATTTATTGGAAATGACCAAATTTATAATGTAATT-GTTACT-GCTCATGCATTTA 118

Query 138 TTATAAttttttttATAGTAATACCTATTATAATTGGAGGATTTGGAAATTGGTTAGTCC 197

|||||||||||||||||||||||||||||||||||||||||||||||||||| ||||| |

Sbjct 119 TTATAATTTTTTTTATAGTAATACCTATTATAATTGGAGGATTTGGAAATTGATTAGTTC 178

Query 198 CATTAATATTAGGTGCTCCTGATATAGCTTTCCCTCGAATAAATAATATAAGTTTTTGAA 257

| ||||||||||| || |||||||||||||| ||||||||||||||||||||||||||||

Sbjct 179 CTTTAATATTAGGAGCCCCTGATATAGCTTTTCCTCGAATAAATAATATAAGTTTTTGAA 238

Query 258 TACTTCCTCCCTCTTTAACTCTTTTAATTTCTAGATCTATAGTAGAAAATGGAGCAGGAA 317

|||||||||| ||||||||||||||| |||||||||||||||||||||||||||||||||

Sbjct 239 TACTTCCTCCTTCTTTAACTCTTTTACTTTCTAGATCTATAGTAGAAAATGGAGCAGGAA 298

Query 318 CAGGTTGAACTGTATACCCTCCTCTTTCTTCTGGAACTGCTCATGCAGGAGCTTCTGTAG 377

| ||||||||||| || |||||||||||||||||||| |||||||| |||||||| || |

Sbjct 299 CTGGTTGAACTGTTTATCCTCCTCTTTCTTCTGGAACCGCTCATGCTGGAGCTTCAGTTG 358

Query 378 ATTTAGCTATTTTTTCTCTTCATTTAGCAGGAATTTCTTCTATTTTAGGAGCAGTAAATT 437

||||||||||||||||| | ||||||||||| ||||| ||||||||||||||||||||||

Sbjct 359 ATTTAGCTATTTTTTCTTTACATTTAGCAGGTATTTCATCTATTTTAGGAGCAGTAAATT 418

Query 438 TTATTACAACTGTAATTAATATACGATCAACTGGAATTACACTTGATCGTTTACCTTTAT 497

||||||||||||||||||||||||||||| |||||||||| |||||||| ||||||||||

Sbjct 419 TTATTACAACTGTAATTAATATACGATCAGCTGGAATTACGCTTGATCGATTACCTTTAT 478

Query 498 TTGTCTGATCTGTAATTATTACAGCTATTTTATTACTTCTTTCATTACCAGTTTTAGCAG 557

|||| ||||| ||| | |||||||| ||||||||||||||||||||||| ||||||||||

Sbjct 479 TTGTTTGATCAGTAGTAATTACAGCAATTTTATTACTTCTTTCATTACCTGTTTTAGCAG 538

Query 558 GAGCTATTACTATATTATTAACTGATCGAAATTTTAATACATCATTTTTTGACCCAATTG 617

|||| || ||||| |||||||||||||||||||||||||||||||||||||| |||||||

Sbjct 539 GAGCAATCACTATGTTATTAACTGATCGAAATTTTAATACATCATTTTTTGATCCAATTG 598

Query 618 GAGGAGGTGACCCTATTCTT 637

|||||||||| |||||||||

Sbjct 599 GAGGAGGTGATCCTATTCTT 618

>Aedes pseudoscutellaris voucher FI010 cytochrome c oxidase subunit I (COX1) gene, partial cds; mitochondrial

Sequence ID: MW664803.1 Length: 618

Range 1: 1 to 618

Score:904 bits(489), Expect:0.0,

Identities:577/620(93%), Gaps:3/620(0%), Strand: Plus/Plus

Query 19 TGATCGGGGA-AGTCGGAACTTCATTAAGAGTTTTAATTCGAACTGAACTTAGTCACCCT 77

||||| || | ||| |||||||| ||||||||||||||||||||||||||||||||||||

Sbjct 1 TGATCTGGAATAGTAGGAACTTCTTTAAGAGTTTTAATTCGAACTGAACTTAGTCACCCT 60

Query 78 GGAATATTTATCGGAAATGATCAAATTTATAATGTAATTCGTTACTAGCTCATGCATTTA 137

|| |||||||| |||||||| |||||||||||||||||| |||||| |||||||||||||

Sbjct 61 GGTATATTTATTGGAAATGACCAAATTTATAATGTAATT-GTTACT-GCTCATGCATTTA 118

Query 138 TTATAAttttttttATAGTAATACCTATTATAATTGGAGGATTTGGAAATTGGTTAGTCC 197

|||||||||||||||||||||||||||||||||||||||||||||||||||| ||||| |

Sbjct 119 TTATAATTTTTTTTATAGTAATACCTATTATAATTGGAGGATTTGGAAATTGATTAGTTC 178

Query 198 CATTAATATTAGGTGCTCCTGATATAGCTTTCCCTCGAATAAATAATATAAGTTTTTGAA 257

| ||||||||||| || |||||||||||||| ||||||||||||||||||||||||||||

Sbjct 179 CTTTAATATTAGGAGCCCCTGATATAGCTTTTCCTCGAATAAATAATATAAGTTTTTGAA 238

Query 258 TACTTCCTCCCTCTTTAACTCTTTTAATTTCTAGATCTATAGTAGAAAATGGAGCAGGAA 317

|||||||||| ||||||||||||||| |||||||||||||||||||||||||||||||||

Sbjct 239 TACTTCCTCCTTCTTTAACTCTTTTACTTTCTAGATCTATAGTAGAAAATGGAGCAGGAA 298

Query 318 CAGGTTGAACTGTATACCCTCCTCTTTCTTCTGGAACTGCTCATGCAGGAGCTTCTGTAG 377

| ||||||||||| || |||||||||||||||||||| |||||||| |||||||| || |

Sbjct 299 CTGGTTGAACTGTTTATCCTCCTCTTTCTTCTGGAACCGCTCATGCTGGAGCTTCAGTTG 358

Query 378 ATTTAGCTATTTTTTCTCTTCATTTAGCAGGAATTTCTTCTATTTTAGGAGCAGTAAATT 437

||||||||||||||||| | ||||||||||| ||||| ||||||||||||||||||||||

Sbjct 359 ATTTAGCTATTTTTTCTTTACATTTAGCAGGTATTTCATCTATTTTAGGAGCAGTAAATT 418

Query 438 TTATTACAACTGTAATTAATATACGATCAACTGGAATTACACTTGATCGTTTACCTTTAT 497

||||||||||||||||||||||||||||| |||||||||| |||||||| ||||||||||

Sbjct 419 TTATTACAACTGTAATTAATATACGATCAGCTGGAATTACGCTTGATCGATTACCTTTAT 478

Query 498 TTGTCTGATCTGTAATTATTACAGCTATTTTATTACTTCTTTCATTACCAGTTTTAGCAG 557

|||| ||||| ||| | |||||||| ||||||||||||||||||||||| ||||||||||

Sbjct 479 TTGTTTGATCAGTAGTAATTACAGCAATTTTATTACTTCTTTCATTACCTGTTTTAGCAG 538

Query 558 GAGCTATTACTATATTATTAACTGATCGAAATTTTAATACATCATTTTTTGACCCAATTG 617

|||| || ||||| |||||||||||||||||||||||||||||||||||||| |||||||

Sbjct 539 GAGCAATCACTATGTTATTAACTGATCGAAATTTTAATACATCATTTTTTGATCCAATTG 598

Query 618 GAGGAGGTGACCCTATTCTT 637

|||||||||| |||||||||

Sbjct 599 GAGGAGGTGATCCTATTCTT 618

>Aedes pseudoscutellaris voucher FI219 cytochrome c oxidase subunit I (COX1) gene, partial cds; mitochondrial

Sequence ID: MW664802.1 Length: 618

Range 1: 1 to 618

Score:904 bits(489), Expect:0.0,

Identities:577/620(93%), Gaps:3/620(0%), Strand: Plus/Plus

Query 19 TGATCGGGGA-AGTCGGAACTTCATTAAGAGTTTTAATTCGAACTGAACTTAGTCACCCT 77

||||| || | ||| |||||||| ||||||||||||||||||||||||||||||||||||

Sbjct 1 TGATCTGGAATAGTAGGAACTTCTTTAAGAGTTTTAATTCGAACTGAACTTAGTCACCCT 60

Query 78 GGAATATTTATCGGAAATGATCAAATTTATAATGTAATTCGTTACTAGCTCATGCATTTA 137

|| |||||||| |||||||| |||||||||||||||||| |||||| |||||||||||||

Sbjct 61 GGTATATTTATTGGAAATGACCAAATTTATAATGTAATT-GTTACT-GCTCATGCATTTA 118

Query 138 TTATAAttttttttATAGTAATACCTATTATAATTGGAGGATTTGGAAATTGGTTAGTCC 197

|||||||||||||||||||||||||||||||||||||||||||||||||||| ||||| |

Sbjct 119 TTATAATTTTTTTTATAGTAATACCTATTATAATTGGAGGATTTGGAAATTGATTAGTTC 178

Query 198 CATTAATATTAGGTGCTCCTGATATAGCTTTCCCTCGAATAAATAATATAAGTTTTTGAA 257

| ||||||||||| || |||||||||||||| ||||||||||||||||||||||||||||

Sbjct 179 CTTTAATATTAGGAGCCCCTGATATAGCTTTTCCTCGAATAAATAATATAAGTTTTTGAA 238

Query 258 TACTTCCTCCCTCTTTAACTCTTTTAATTTCTAGATCTATAGTAGAAAATGGAGCAGGAA 317

|||||||||| ||||||||||||||| |||||||||||||||||||||||||||||||||

Sbjct 239 TACTTCCTCCTTCTTTAACTCTTTTACTTTCTAGATCTATAGTAGAAAATGGAGCAGGAA 298

Query 318 CAGGTTGAACTGTATACCCTCCTCTTTCTTCTGGAACTGCTCATGCAGGAGCTTCTGTAG 377

| ||||||||||| || |||||||||||||||||||| |||||||| |||||||| || |

Sbjct 299 CTGGTTGAACTGTTTATCCTCCTCTTTCTTCTGGAACCGCTCATGCTGGAGCTTCAGTTG 358

Query 378 ATTTAGCTATTTTTTCTCTTCATTTAGCAGGAATTTCTTCTATTTTAGGAGCAGTAAATT 437

||||||||||||||||| | ||||||||||| ||||| ||||||||||||||||||||||

Sbjct 359 ATTTAGCTATTTTTTCTTTACATTTAGCAGGTATTTCATCTATTTTAGGAGCAGTAAATT 418

Query 438 TTATTACAACTGTAATTAATATACGATCAACTGGAATTACACTTGATCGTTTACCTTTAT 497

||||||||||||||||||||||||||||| |||||||||| |||||||| ||||||||||

Sbjct 419 TTATTACAACTGTAATTAATATACGATCAGCTGGAATTACGCTTGATCGATTACCTTTAT 478

Query 498 TTGTCTGATCTGTAATTATTACAGCTATTTTATTACTTCTTTCATTACCAGTTTTAGCAG 557

|||| ||||| ||| | |||||||| ||||||||||||||||||||||| ||||||||||

Sbjct 479 TTGTTTGATCAGTAGTAATTACAGCAATTTTATTACTTCTTTCATTACCTGTTTTAGCAG 538

Query 558 GAGCTATTACTATATTATTAACTGATCGAAATTTTAATACATCATTTTTTGACCCAATTG 617

|||| || ||||| |||||||||||||||||||||||||||||||||||||| |||||||

Sbjct 539 GAGCAATCACTATGTTATTAACTGATCGAAATTTTAATACATCATTTTTTGATCCAATTG 598

Query 618 GAGGAGGTGACCCTATTCTT 637

|||||||||| |||||||||

Sbjct 599 GAGGAGGTGATCCTATTCTT 618
