# Supplementary figures and images for: Comprehensive intra-host infection kinetics reveals high arbo-orthoflavivirus transmission potential by neglected vector species, Aedes scutellaris
Source: PLoS Negl Trop Dis. 2025 May 6;19(5):e0012530. doi: 10.1371/journal.pntd.0012530 (PMC12080922; doi:10.1371/journal.pntd.0012530)

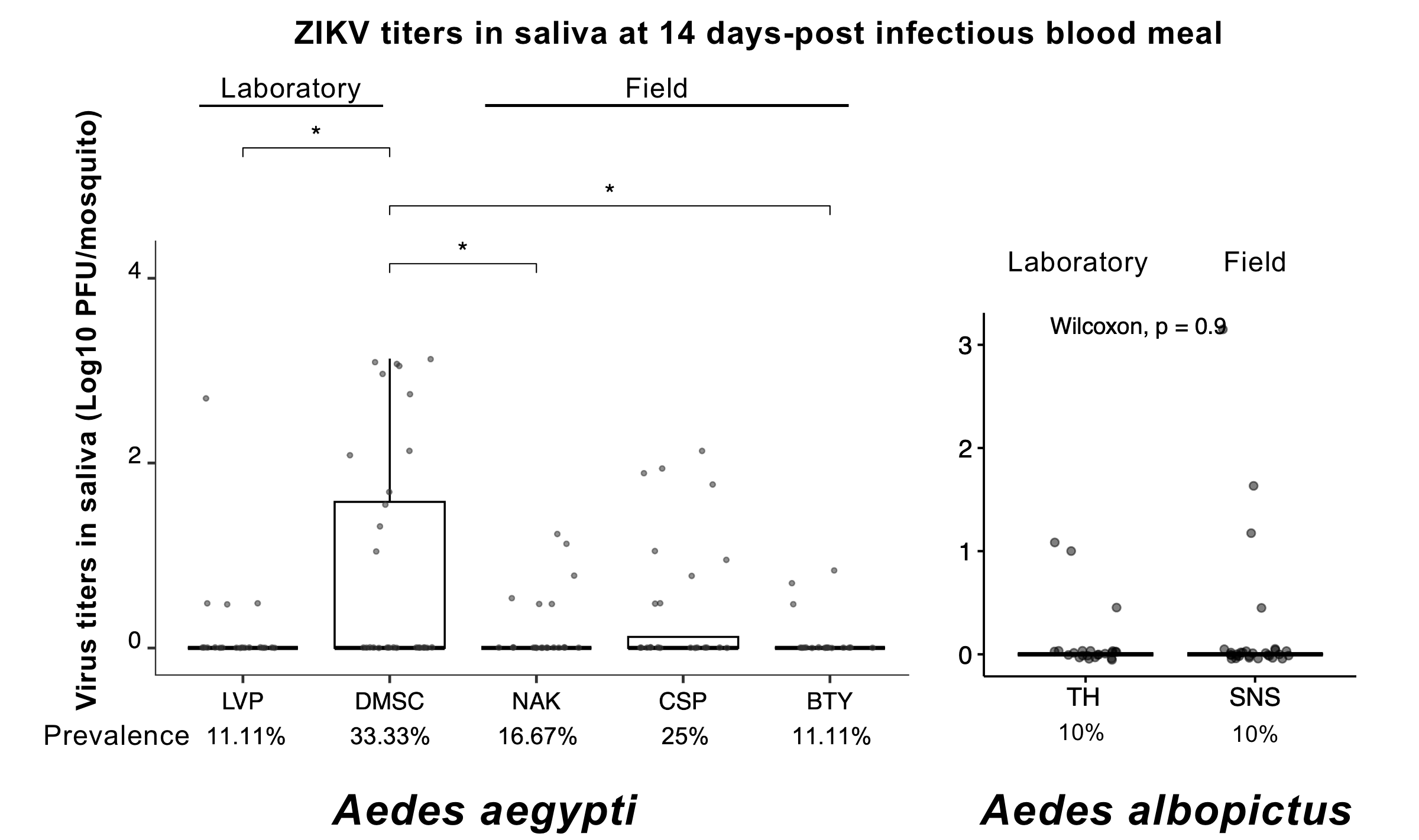

Supplement: S1 Fig — Box and scatter plot comparing virus titers in saliva at 14 dpibm. Each dot represents titers in saliva of each individual mosquito. The boxes in the plots represent the interquartile range, and the whiskers indicate the range of maximum and minimum values, excluding outliers (less than Q1 - 1.5 X IQR, or more than Q3 + 1.5 X IQR). The Ae. aegypti strains consist of two laboratory strains: DMSC and LVP and three field strains: NAK, CSP and BTY. The Ae. albopictus strains consist of one laboratory strain TH and one field strain SNS. The data were summarized from one blood feeding experiment with at least 30 mosquitoes per group. Statistical analysis comparing infection level was conducted using Kruskal-Wallis followed by Dunn’s post-hoc test for Ae. aegypti and Wilcoxon rank sum test for Ae. albopictus. *: p < 0.05. (TIFF) [file pntd.0012530.s006.tiff]

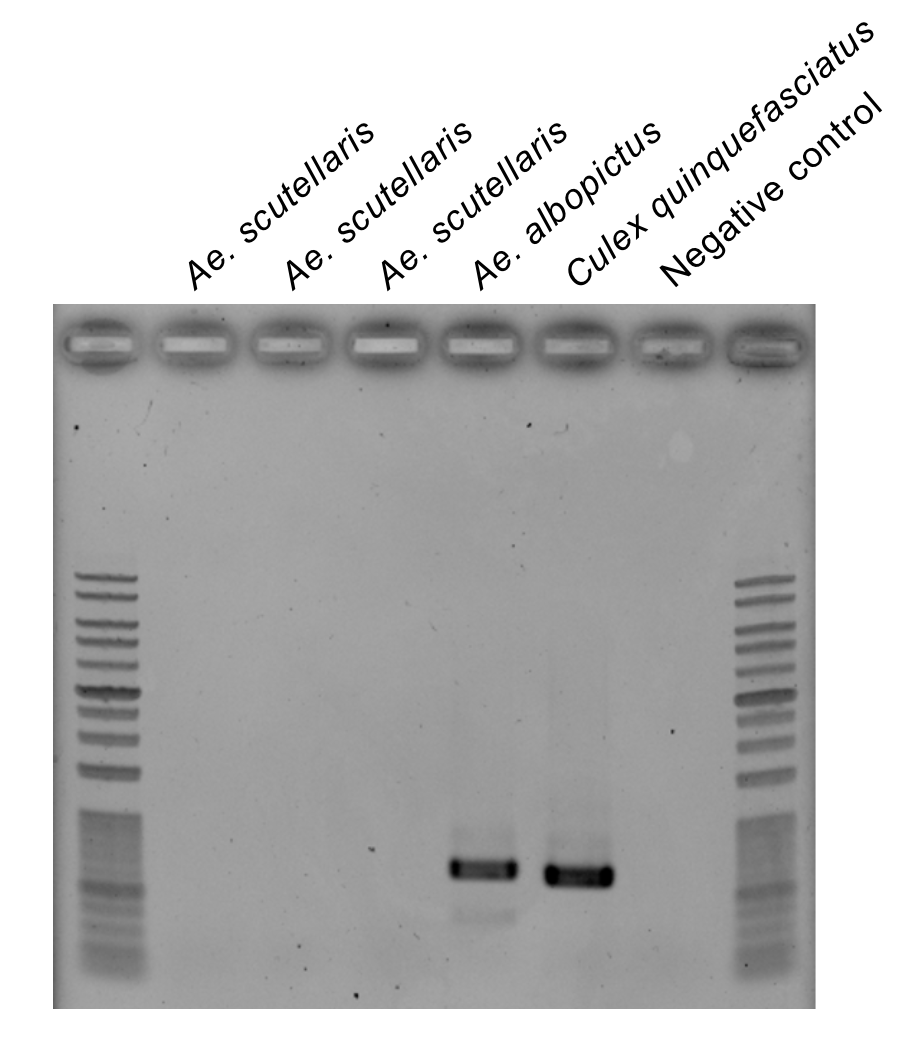

Supplement: S2 Fig — The presence of Wolbachia was detected by PCR of wsp gene. None of the Ae. scutellaris samples were infected by Wolbachia while the wsp gene was detected in Ae. albopictus and Culex quinquefasciatus used as positive controls. (TIFF) [file pntd.0012530.s007.tiff]
